# Controlling Solid-State Mechanochemical Pathways in Triarylmethane Mechanophores via Molecular and Matrix Design

**DOI:** 10.1021/acs.macromol.6c00789

**Published:** 2026-07-13

**Authors:** Dnyaneshwar Ajinath Mache, Eduardo García-Padilla, Federico Frateloreto, Gaia Egizzo, James R. Hemmer, Javier G. Valverde-Bastidas, Silvia De la Flor, Feliu Maseras, José Augusto Berrocal

**Affiliations:** † Institute of Chemical Research of Catalonia (ICIQ), 202569 Barcelona Institute of Science and Technology (BIST), Tarragona 43007, Spain; ‡ Departament de Química Orgánica, 16777 Universitat Rovira i Virgili, Tarragona 43307, Spain; § 311305 Adolphe Merkle Institute, Chemin des Verdiers 4, Fribourg CH-1700, Switzerland; ∥ Department of Mechanical Engineering, Universitat Rovira i Virgili, Tarragona 43307, Spain; ⊥ ICREA, Pg. Lluís Companys 23, Barcelona 08010, Spain

## Abstract

Controlling reaction pathways under mechanical force is a challenge in polymer mechanochemistry. Most mechanophores exhibit a single dissociation route, and examples of mechanistic multiplicity are exceedingly rare—particularly in the solid state. Here, we report triarylmethane mechanophores that enable predominant homolytic or heterolytic pathways in polymer networks through combined molecular and matrix design. Two variants were synthesized: **Tr-1**, bearing a benzyl ether leaving group connected through a weak C–O bond, and **Tr-2**, incorporating a cyanoacetate leaving group linked via a weak C–C bond. Embedded in poly­(methyl acrylate) (PMA) and poly­(methyl acrylate-*co*-2-hydroxylethyl acrylate) (P­(MA-*co*-HEA)) networks, these mechanophores display mechanochromic responses that report distinct cleavage mechanisms. **Tr-1** undergoes homolysis in PMA, while hydrogen-bond donors in P­(MA-*co*-HEA) promote heterolysis. **Tr-2** favors heterolytic dissociation regardless of matrix composition, instead. Optical measurements, EPR spectroscopy, and DFT calculations support these assignments and further establish triarylmethane ethers as mechanochemical probes of hydrogen bond donors.

## Graphical abstract

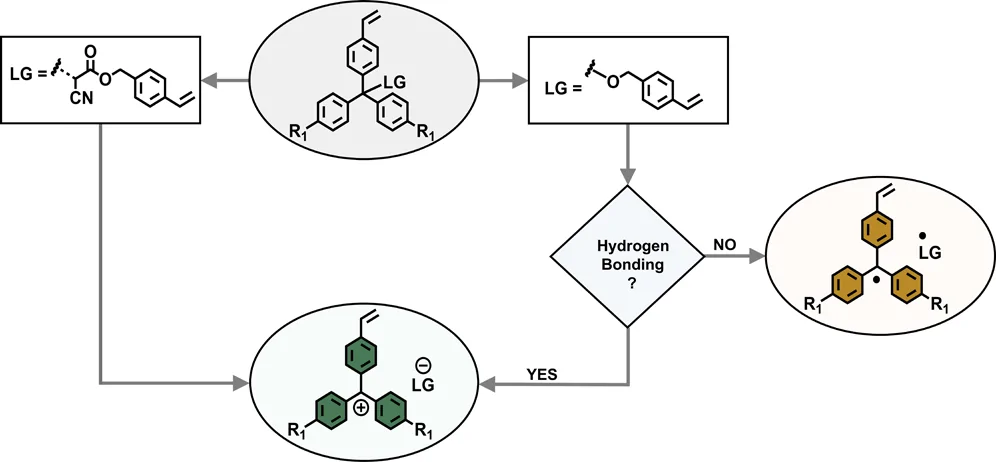

## Introduction

Chemical reactions often exhibit a strong intrinsic bias toward a single mechanistic regime—ionic or radical—set by the relative stability of intermediates and the surrounding medium.^1,2^ Overcoming this bias to selectively access alternative pathways is a longstanding goal across synthetic and physical chemistry,^1,2^ with broad implications for reactivity control and materials design. In this context, mechanical force can provide an orthogonal input to traditional thermodynamic and kinetic controls by coupling directional work to specific coordinates.^3–7^ Such an approach has been shown to reshape potential energy landscapes in ways that complement those from “more conventional” strategies.^3–7^

Polymer mechanochemistry explores this concept through mechanophores—molecular units embedded in polymer systems that undergo well-defined chemical transformations under load.^8–13^ By localizing force vectors along scissile bonds, mechanophores enable “productive”^14^ bond activation, which has found application in mechanocatalysis, mechanochromism, and stress/strain-sensing soft materials, among others.^15–18^ Nevertheless, achieving precise mechanistic control remains challenging: most mechanophores follow a single dissociation pathway under force, and deliberate mechanistic control, such as switching between homolytic and heterolytic scission, for example, remains rare.^10,19,20^

To our best knowledge, mechanistic multiplicity—the coexistence or switchability among distinct force-coupled pathways—has been observed only with a few examples in solution, thus far. De Bo and co-workers reported a mechanophore that undergoes three concomitant C–C dissociation pathways under ultrasound and further demonstrated that subtle changes, i.e., isotopic substitution, can redirect the preferred route.^21,22^ These studies highlight how small structural variations and force transduction can shift outcomes among homolytic, heterolytic, and concerted pathways.^21,22^ However, they rely on solvating media that stabilize polar (ionic) intermediates and provide tunable dielectric environments, which are conditions fundamentally distinct from cross-linked polymers.

By contrast, in solid-state polymer networks, the absence of bulk solvation and the generally apolar character of common elastomeric matrices typically favor homolytic scission, making heterolytic cleavage significantly less frequent to encounter.^10,19,21^ Triarylmethane-based mechanophores represent one of the few exceptions in which force-coupled heterolysis can be induced in a polymer matrix doped with hydrogen-bonding donors, such as poly­(methyl acrylate-*co*-2-hydroxyethyl acrylate) (P­(MA-*co*-HEA)) (Figure 1a).^23,24^ Yet, a tunable strategy to bias solid-state mechanophores predominantly toward radical and ionic pathways, in analogy with solvent-controlled selectivity in solution, remains elusive.

**Figure 1:**
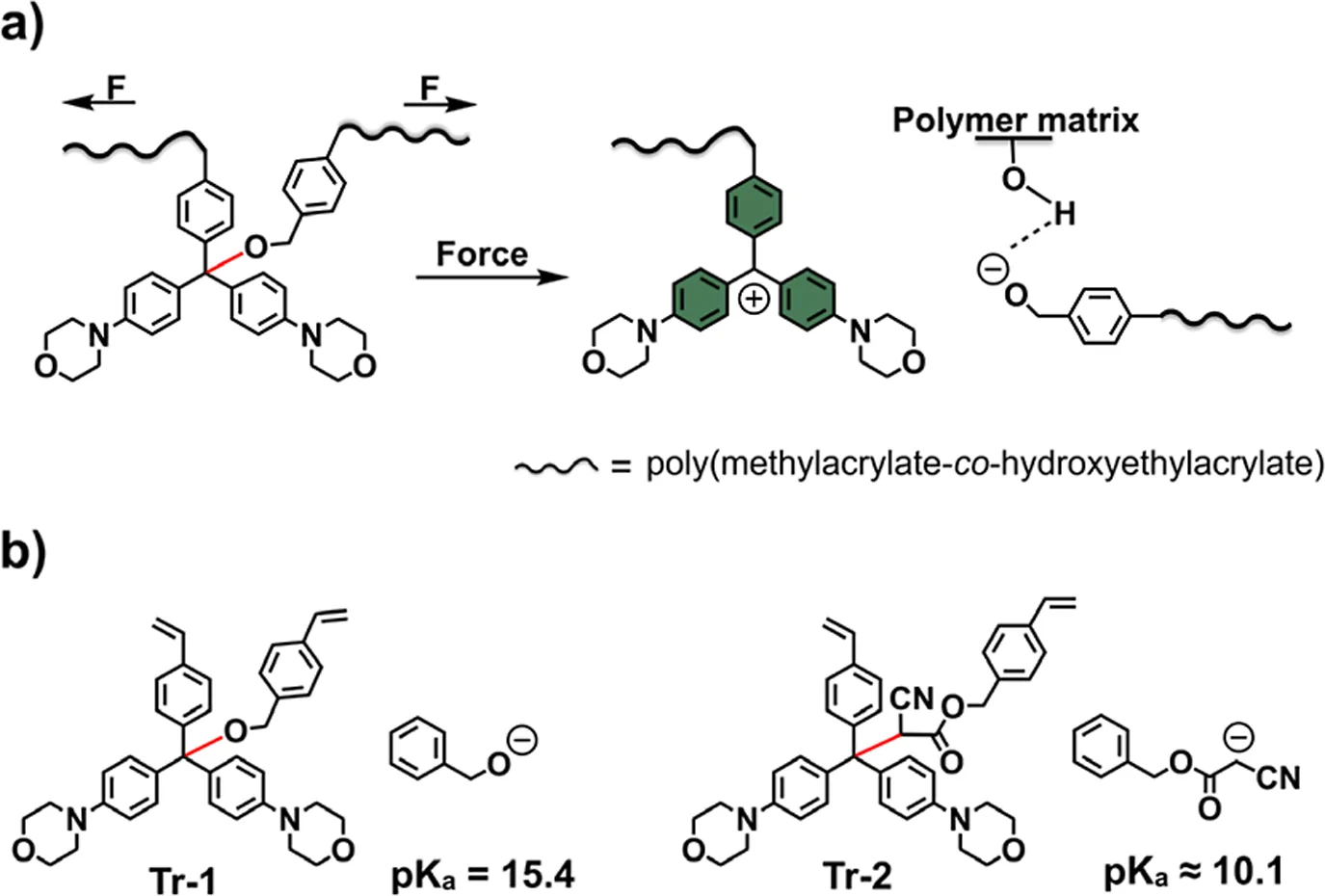
(a) Force-induced C–O heterolytic bond cleavage in triarylmethane benzyl ether mechanophores assisted by hydrogen-bonding. (b) Chemical structures of **Tr-1** and **Tr-2**, with their associated leaving groups and the p*K*_a_ values of the corresponding conjugate acids.

Here, we address this challenge by bringing molecular design into dialogue with matrix engineering. Triarylmethanes are particularly attractive because their radical- and cationic-derived skeletons are both stabilized by resonance, making them sensitive to subtle electronic and environmental cues.^25,26^ We reasoned that varying the leaving group (LG) basicity and the nature of the scissile bond (C–O vs C–C) would tune the thermodynamic driving forces for heterolytic versus homolytic cleavage under force, while hydrogen-bond donors in the polymer would provide local stabilization of anionic counterparts formed upon heterolysis. Building on this rationale, we compare triarylmethane derivatives **Tr-1** ^23^ and **Tr-2** (Figure 1b) in poly­(methyl acrylate) (PMA) and P­(MA-*co*-HEA) networks incorporating 5 or 10 wt % of HEA. The two compounds possess the same triarylmethane core substituted with morpholine moieties in two aromatic rings, so each is expected to generate radical species of comparable stability to one another, and the same is expected for the corresponding carbocations. However, the LGs of **Tr-1** and **Tr-2** are different. **Tr-1** features a benzyl-ether weak C–O bond that, in the case of heterolytic cleavage, produces a benzyl alcoholate anion with a p*K*_a_ (of the conjugate acid) of 15.4 (Figure 1b).^27^ The p*K*_a_ of the conjugate acid of the cyanoacetate anionic fragment of **Tr-2** is ca. 10.1,^28^ instead (Figure 1b). Thus, **Tr-1** and **Tr-2** should provide complementary handles to bias ion-pair formation without resorting to additives.

In summary, with the **Tr-1**–**2** pair of compounds, we ask whether a mechanophore can be driven into different mechanochemical mechanisms in the solid state by modulating local hydrogen-bonding, and whether orthogonal molecular design (nature of leaving-group and scissile bond) can bias the mechanochemical pathway. To our best knowledge, these questions have not been addressed for solid polymer networks, and define the novelty of this study.

## Results and Discussion

To evaluate how molecular design and matrix polarity govern the mechanochemical reactivity of triarylmethane mechanophores, we first examine their intrinsic thermodynamic preferences using density functional theory (DFT). These calculations could provide insights into how leaving-group identity and scissile-bond character bias homolytic versus heterolytic cleavage in environments of different polarity (i.e., dielectric constant) and hydrogen-bonding capability. Guided by these predictions, we next investigate the mechanochemical behavior of **Tr-1** and **Tr-2** incorporated into cross-linked polymer networks with systematically varied hydrogen-bond donor content (0, 5, and 10 wt % HEA). To further decouple polarity and hydrogen-bonding effects, additional copolymer systems incorporating 2-methoxyethyl acrylate (MEA), i.e., a methylated analog of HEA, were also examined. Their force-induced activation is followed by in situ mechanochromic analysis under tensile deformation, providing wavelength-resolved signatures of radical or carbocation formation. Finally, electron paramagnetic resonance (EPR) measurements directly probe the spin-generating pathways and validate the mechanistic assignments inferred from optical data. Together, these experimental and computational results establish the principles by which local polymer microenvironments and mechanophore structure jointly dictate mechanochemical outcomes in the solid state.

### Theoretical Studies on the C–C and C–O Bond Cleavage

We first performed DFT calculations on simplified model compounds **1** and **2**, representing the scissile C–O bond of **Tr-1** and the scissile C–C bond of **Tr-2**, respectively (Figure 2), to establish the intrinsic thermodynamic factors governing homolytic versus heterolytic scission in the triarylmethane mechanophores. In these models, the styrenyl polymer handles were replaced with methyl groups to reduce computational cost while preserving the key electronic and structural features of the mechanophores. The calculations were carried out at the B3LYP-D3/6-311+G­(d,p) level of theory using the polarizable continuum model (PCM) to approximate a range of dielectric environments relevant to polymer matrices. The computational study aimed at (i) determining how the nature of the leaving-group and weak bond influence the intrinsic preference for radical versus ionic cleavage, and (ii) evaluating how local polarity and hydrogen bonding—two features expected to vary in PMA and P­(MA-*co*-HEA) matrices—modulate these preferences.

**Figure 2:**
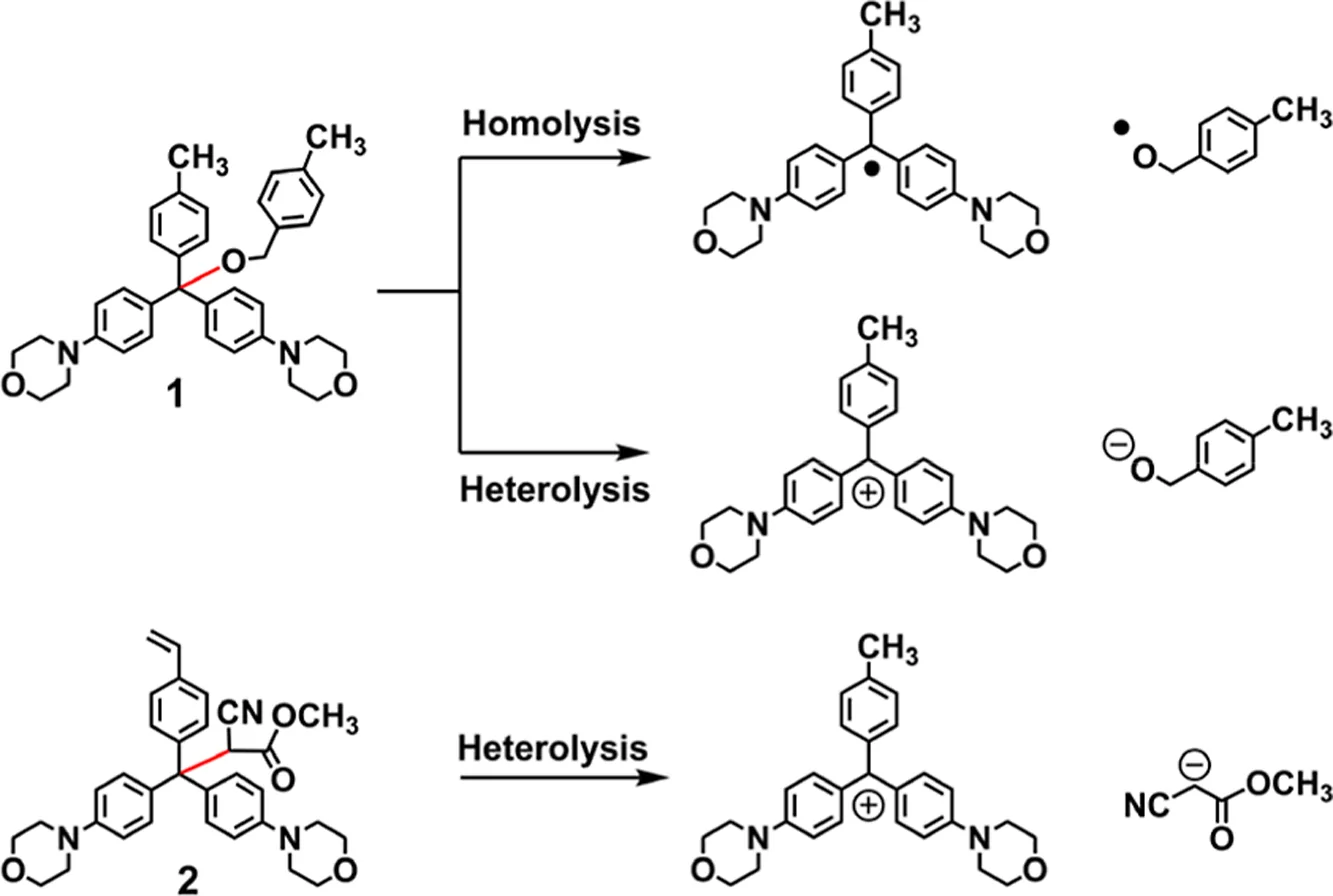
Chemical structures of model compounds **1** and **2**, and those of the products derived from the homolytic and heterolytic pathways studied computationally.

We selected four media spanning a broad range of dielectric constants: ethanol (ε = 24.85; polar medium), methyl propanoate and methyl butanoate (ε = 5.07 and 5.56, respectively; intermediate polarity), and poly­(methyl methacrylate) (PMMA) (ε = 3.90; low polarity).^29,30^ The last medium represents a solid polymer environment similar to PMA. It is important to stress that, within the PCM framework, these environments provide only bulk polarity, i.e., the model does not encode specific hydrogen-bonding interactions. Thus, this initial set of calculations probes how polarity alone affects the thermodynamics of homolytic versus heterolytic cleavage in the absence of explicit donor–acceptor interactions. This was determined by comparing the calculated free energy of the radical and ionic fragments (Table 1). The free energy of both homolytic and heterolytic bond cleavages (Δ*G*_hom_ and Δ*G*_het_, respectively) of model compounds **1** and **2** was calculated in the four different media, and compared against each other (ΔΔ*G*) to examine the dependence of the thermodynamics bias on the polymer environment (Table 1), as the dielectric environments are known to strongly alter the outcomes in mechanochemical reactions.^31–33^ Some of the absolute values for dissociation energy are quite large, but it must be taken into account that the cleavage takes place under mechanical stress. This is why we focus on the comparison between the two paths. Positive values of ΔΔ*G* highlight a preference for heterolysis, while negative ΔΔ*G* implies a thermodynamic tendency for homolysis (Table 1). Significantly large absolute values of ΔΔ*G* would suggest a strong thermodynamic bias, with practically full selectivity for one of the product pairs beyond ±3 kcal/mol.

**Table 1:** Calculated (B3LYP-D3/6-311+G­(d, p), PCM; 1 M, 298 K) Free Energy (kcal/mol) for the Homolysis and Heterolysis of the C–O Bond of **1** (Δ*G*_hom_ and Δ*G*_het_, Respectively), Difference between the Calculated Free Energy for the Homolysis and Heterolysis of the C–O Bond of **1** (ΔΔ*G*), and the Calculated Free Energy for the Heterolysis of the C–O Bond of **1** in the Presence of an Explicit Molecule of Methanol (Δ*G*_het_ MeOH) in Ethanol, Methyl Propanoate, Methyl Butanoate, and poly­(methyl Methacrylate) (PMMA); Calculated Free Energy (kcal/mol) for the Homolysis and Heterolysis of the C–C Bond of **2** (Δ*G*_hom_ and Δ*G*_het_, Respectively) and Difference between the Calculated Free Energy for the Homolysis and Heterolysis of the C–C Bond of **2** (ΔΔ*G*).^a^

solvent	**1**				**2**		
	Δ*G*_hom_ (kcal/mol)	Δ*G*_het_ (kcal/mol)	ΔΔ*G* (kcal/mol)	Δ*G*_het_ MeOH (kcal/mol)	Δ*G*_hom_ (kcal/mol)	Δ*G*_het_ (kcal/mol)	ΔΔ*G* (kcal/mol)
ethanol	39.0	25.9	+13.1	+19.8	24.7	2.0	+22.7
methyl propanoate	39.4	38.2	+1.2	+30.8^a^	24.8	12.3	+12.5
methyl butanoate	39.5	39.7	–0.2	(+32.1)^a^	24.8	13.5	+11.3
PMMA	39.9	47.1	–7.2	(+38.8)^a^	24.6	19.3	+5.3

a For polymer matrices with no copolymerized alcohol groups, no hydrogen bonding moieties are expected to be available.

For model **1**, representing the weak C–O bond of **Tr-1**, homolytic cleavage produces a triarylmethyl radical and a *p*-methyl-benzyloxy radical, whereas heterolytic cleavage produces a triarylmethyl cation and a *p*-methyl benzyloxide anion (Figure 2). In a polar environment such as ethanol, the heterolytic pathway is strongly favored (ΔΔ*G* = +13.1 kcal/mol; Table 1), reflecting efficient stabilization of charge-separated species by a high dielectric medium. As the dielectric constant decreases, the thermodynamic advantage of heterolysis diminishes: model **1** shows nearly degenerate pathways in methyl butanoate, and a clear preference for homolysis in the PMMA-like environment (Table 1). These trends directly mirror what one would expect for ionic versus radical pathways in media of decreasing polarity.

We next assessed whether specific hydrogen-bonding interactions, which are absent in PCM, could alter this balance in the four media. Because the anion formed upon heterolysis of **1** has a non-negligible basicity (p*K*_a_ ≈ 15.4), it is reasonable to expect stabilization by hydrogen-bond donors in polymer matrices containing HEA. To model this effect, we introduced one explicit methanol (MeOH) molecule in several orientations around the benzyloxide anion. Even a single hydrogen-bonding interaction was sufficient to favor heterolysis in otherwise apolar environments, suggesting that modest local interactions can compensate for the lack of bulk polarity and reshape the preferred dissociation pathway (see the column “Δ*G*_het_ MeOH” in Table 1).

Model **2**, representing the weak C–C bond of **Tr-2**, displayed a strikingly different profile. Across all media—from ethanol to PMMA—the heterolytic cleavage pathway remained consistently preferred, with ΔΔ*G* values ranging from +22.7 kcal/mol in ethanol to +5.3 kcal/mol in the PMMA-like environment (Table 1). This observation strongly suggests that **Tr-2** should be intrinsically biased toward heterolysis, regardless of environmental polarity, likely due to the substantially lower basicity of the cyanoacetate leaving group relative to a benzyloxide. As the PCM framework suggested that no additional hydrogen-bonding stabilization was required to bias **Tr-2** toward heterolysis, additional calculations with an explicit MeOH molecule were not performed.

Taken together, these calculations yield two clear predictions. Compound **1** (or **Tr-1**) lies near a mechanistic boundary in which its homolysis/heterolysis is sensitive to local polarity and hydrogen bonding. Thus, matrix composition (e.g., varying HEA content) might modulate its mechanochemical outcome. On the other hand, **2** (or **Tr-2**) possesses a strong intrinsic heterolytic bias and, therefore, should produce carbocations under mechanical load regardless of matrix environment.

### Synthesis of Small Molecules and their Incorporation in a Polymer Matrix

Motivated by the thermodynamic insights obtained from the DFT calculations, we synthesized triarylmethane derivatives **Tr-1** and **Tr-2** for experimental evaluation. The preparation of **Tr-1** has been reported previously;^23^ hence, synthetic procedures were simply repeated. In contrast, **Tr-2** required a new synthetic route that enabled installation of a cyanoacetate leaving group linked via a weak C–C bond.

The synthesis of **Tr-2** began with the generation of its triarylmethyl carbocation precursor (Scheme 1a). Treatment of triarylcarbinol **3** ^23^ with boron trifluoride etherate (BF_3_·OEt_2_) produced the corresponding air- and bench-stable salt **4**. In parallel, cyanoacetic acid **5** was coupled with 4-vinylbenzyl chloride **6** to furnish the styrenyl cyanoacetate derivative **7** after chromatographic purification. Deprotonation of **7** with potassium hydroxide (KOH) generated the cyanoacetate nucleophile, to which **4** was added to form **Tr-2** via C–C bond formation. Purification by column chromatography afforded **Tr-2** in analytically pure form. All intermediates and final products were characterized by ^1^H and ^13^C NMR, FT-IR spectroscopy, and high-resolution mass spectrometry. Full description of the synthetic procedures and characterization data are provided in the Supporting Information (SI, pages S5–S24).

**Scheme 1:**
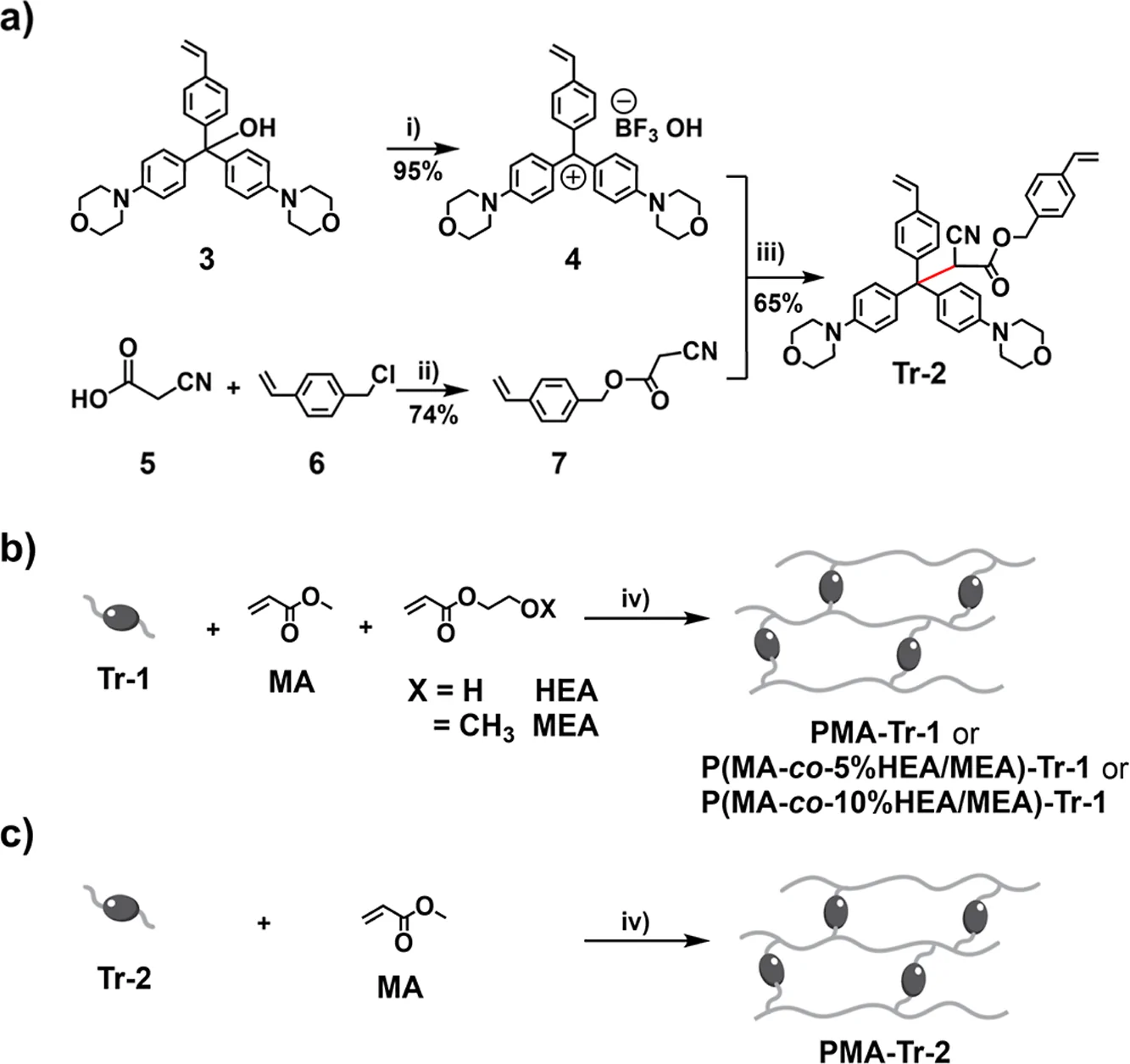
(a) Synthesis of **Tr-2**. (b) Preparation of **PMA-Tr-1**, **P­(MA-** *co* **-5%HEA/MEA)-Tr-1**, and **P­(MA-** *co* **-10%HEA/MEA)-Tr-1**. (c) Preparation of **PMA-Tr-2** ^a^

a Reaction Conditions: (i) BF_3_•OEt_2_, DCM, rt, 1 h; (ii) KOH, DMF, 70 °C, 24 h; (iii) *t*-BuOK, DMF, rt, 30 min; (iv) Irgacure 2959, λ = 365 nm, rt, 10 min.

To investigate the influence of hydrogen bonding and leaving-group identity on mechanophore activation, **Tr-1** and **Tr-2** were incorporated as cross-linkers into poly­(methyl acrylate)-based networks with a loading of 1 mol %. Films were fabricated via free-radical polymerization of methyl acrylate (**MA**) and, when necessary, 2-hydroxyethyl acrylate (**HEA**), in the presence of the photoinitiator Irgacure^©^ 2959 (Scheme 1b,c). By using HEA contents of 0, 5, and 10 wt %, we systematically modulated the hydrogen-bond donor density within the polymer matrix, producing a homopolymer network (**PMA-Tr-1**) and two copolymers containing 5 or 10 wt % HEA, namely **P­(MA-** *co* **-5%HEA)-Tr-1** and **P­(MA-** *co* **-10%HEA)-Tr-1**. To further probe the possible influence of hydrogen bonding on the mechanochemical activation of **Tr-1**, we prepared additional polymer networks comprising **MA** and 5 and 10 wt % 2-methoxyethyl acrylate (**MEA**), i.e., **P­(MA-** *co* **-5%MEA)–Tr-1** and **P­(MA-** *co* **-10%MEA)–Tr-1** (Scheme 1b). The **MEA** monomer is a methylated analog of **HEA** that is incapable of serving as a hydrogen bond donor. This choice maintains a similar polymer backbone and does not alter bulk polarity (vide infra). Finally, **PMA-Tr-2** was obtained by incorporating **Tr-2** into a poly­(methyl acrylate) network under analogous conditions to those for **PMA-Tr**-**1** (Scheme 1c). The detailed description of the synthetic procedures is available in the Supporting Information (pages S8–S10).

The polymer films (∼200 μm thickness) were prepared by curing monomer mixtures between glass slides under irradiation with 365 nm light. All films were obtained as transparent elastomers, enabling direct optical analysis during mechanical deformation. Thermal characterization of the polymer films carried out with thermogravimetric analysis (TGA) and differential scanning calorimetry (DSC) revealed comparable thermal stabilities (degradation starting at ca. 380 °C) and glass transition temperatures (*T*_g_ ≈ 18–20 °C) for all networks (pages S25–S31), pointing to the fact that **HEA**/**MEA** incorporation did not substantially alter bulk thermal properties. The consistency of the gel content (approximately 90–95%) for the polymers incorporating **Tr-1** and **Tr-2** suggested a comparable density of cross-links across the entire series (Supporting Information, page S32). This was also confirmed by dynamic mechanical analysis (DMA), which showed consistent storage modulus values above *T*_g_ for all polymers (pages S33–S34). These compositionally controlled materials provide an ideal platform to evaluate how local polarity and hydrogen-bonding capability govern force-coupled bond scission in **Tr-1** and **Tr-2**.

### Mechanochromic Behavior

To quantitatively probe the mechanochromic response, uniaxial tensile tests were performed on rectangular specimens (length ≈3 cm, thickness ≈200 μm, width ≈5 mm). A microtensile tester coupled with a spectrophotometer enabled simultaneous recording of the stress–strain response and optical transmission during deformation (strain rate of 2.20% s^–1^ = 375 μm s^–1^).

We first examined the mechanochemical response of **Tr-1** embedded in PMA and P­(MA-*co*-HEA) networks under uniaxial elongation. Manual stretching of all films produced localized coloration in the gauge section of the films, hinting at force-induced activation of **Tr-1** (Figure 3a, d, and g; Supporting Information Movie 1, Movie 2, and Movie 3). The nature of the color change, however, depended strongly on the presence and amount of hydrogen-bond donors in the matrix.

**Figure 3:**
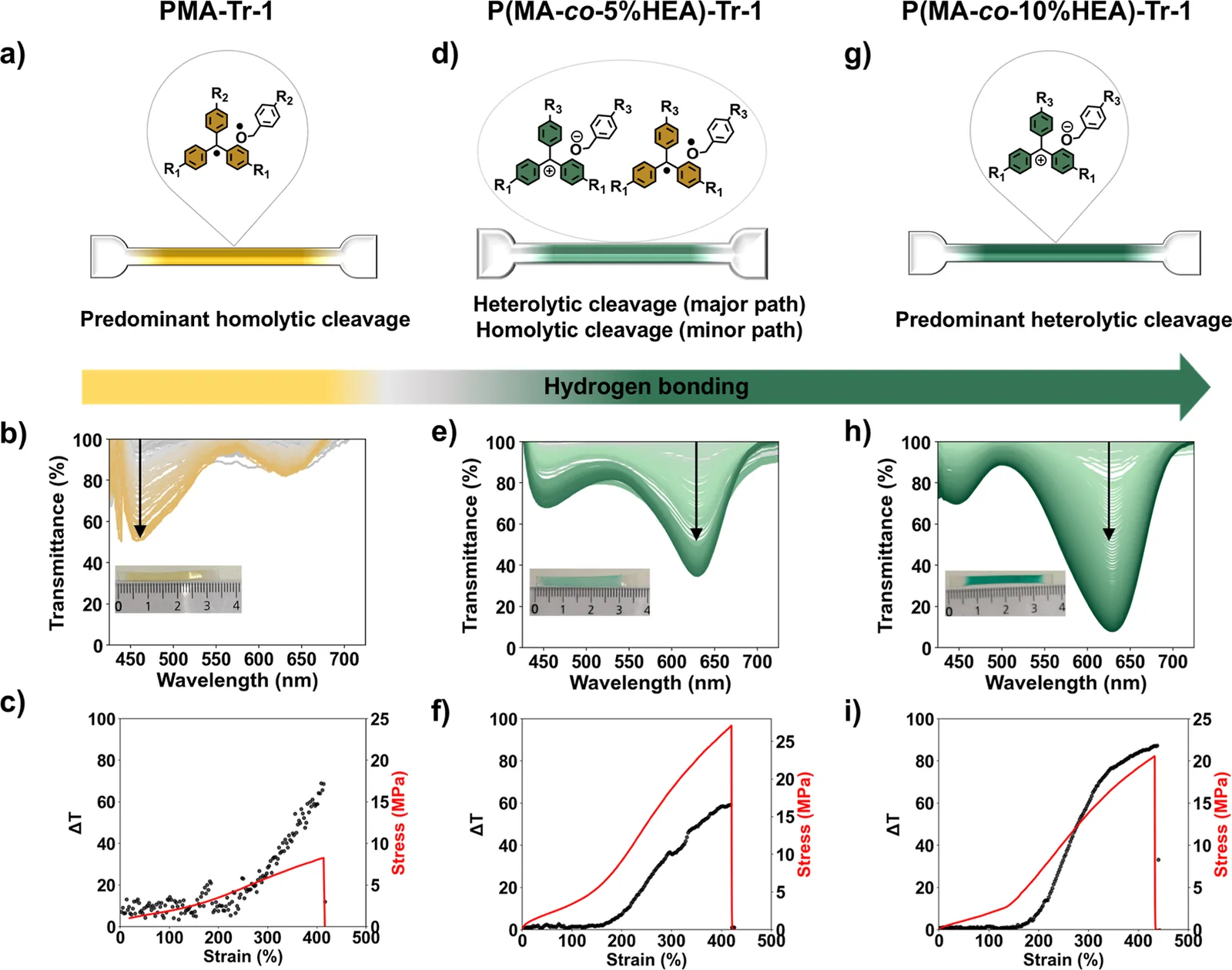
Schematic illustration of force-triggered dissociation of **Tr-1** following (a) homolytic, (d) heterolytic (major) and homolytic (minor), and (g) heterolytic dissociation pathways. Transmittance spectra recorded during stretching of (b) **PMA-Tr-1**, (e) **P­(MA-** *co* **-5%HEA)-Tr-1**, and (h) **P­(MA-** *co* **-10%HEA)-Tr-1** films. The black arrow shows the direction of increasing applied strain (top → bottom). The insets show photographs of polymer films taken after tensile deformation, next to a ruler (dimensions in cm). Stress–strain curves (red traces) and simultaneously measured relative transmittance changes (black traces) for (c) **PMA-Tr-1**, where ΔTransmittance = *T*(700 nm) – *T*(444 nm), (f) **P­(MA-** *co* **-5%HEA)-Tr-1**, with ΔTransmittance = *T*(725 nm) – *T*(630 nm), and (i) **P­(MA-** *co* **-10%HEA)-Tr-1**, with ΔTransmittance = *T*(710 nm) – *T*(630 nm).

In the absence of **HEA**, **PMA-Tr-1** films developed an orange coloration upon deformation (Supporting Information Movie 1). In situ transmittance spectroscopy during tensile testing revealed two bands: a dominant feature centered at 444 nm and a weaker band near 630 nm (Figure 3b). In line with our previous investigations,^24^ we quantified the color evolution via the delta transmittance (Δ*T*) mitigation, which compensates for the shift in baseline across the series of optical measurements due to sample thinning (Δ*T* = Transmittance (700 nm) – Transmittance (444 nm) for **PMA-Tr-1**). The Δ*T* vs elongation (strain %) profile for **PMA-Tr-1** shows that mechanochemical activation begins at strains above ∼200% and increases monotonically with strain (Figure 3c). The spectral profile of the mechanically generated species, and in particular the stronger absorption in the 400–450 nm region, is consistent with triarylmethyl radical species, as suggested by previous literature on malachite-green radicals,^34,35^ which are structurally and electronically similar to the triarylmethane core of **Tr-1**—the only difference being the replacement of the morpholine moieties with dimethylamino groups. The possible mechanochemical generation of radical species in **PMA-Tr-1** aligns well with the DFT prediction that homolytic cleavage of **Tr-1** is favored in an apolar environment lacking hydrogen bond donors. The stretched films became more intensely colored upon exposure to air, suggesting that the formed radical likely reacts with oxygen, forming a stable adduct.^36–38^

Incorporation of 5 wt % HEA into the polymer network (i.e., **P­(MA-** *co* **-5%HEA)-Tr-1**) dramatically altered the mechanochemical signature of **Tr-1**. These films developed a light bluish-green coloration upon stretching (Supporting Information Movie 2), and their transmittance spectra featured two bands whose relative intensities were inverted compared to those observed with **PMA-Tr-1**, namely a stronger band at 630 nm and a weaker one at 444 nm (Figure 3e). These two bands qualitatively match those present in the independently measured absorption spectrum of salt **4** in acetonitrile, whose absorption in the visible part of the spectrum is ascribed to the triarylmethyl carbocation (Figure S40). This spectral resemblance provides strong evidence for the occurrence of the heterolytic pathway in **P­(MA-** *co* **-5%HEA)-Tr-1**. However, a close comparison between the transmittance spectra of **P­(MA-** *co* **-5%HEA)-Tr-1** measured right before film failure and the absorption spectrum of **4** reveals a significant difference between the intensity ratio of the two maxima (630 and 444 nm) (Figure S40). This strongly hints at the coexistence of radical and carbocation triarylmethane species, consistent with the DFT prediction that **Tr-1** lies near a mechanistic boundary in moderately polar environments. For **P­(MA-** *co* **-5%HEA)-Tr-1**, Δ*T* analysis (Δ*T* = Transmittance (725 nm) – Transmittance (630 nm)) indicated mechanochemical activation at slightly lower strains (∼180%) and a linear correlation with applied strain (Figure 3f). The stretched bluish-green films gradually turned orange over time, suggesting the occurrence of secondary processes leading to the formation of a triarylmethyl radical or its oxygen adduct.^36–38^

It is important to note that both triarylmethyl radicals and carbocations exhibit partially overlapping absorption features in the visible region, with maxima centered approximately at 450 nm and 630–640 nm, and with inverted relative intensities. As a result, our mechanistic assignment cannot rely solely on the presence of individual absorption bands; instead, it depends on their relative intensities, in combination with EPR measurements (vide infra). Quantitative partitioning of homolytic versus heterolytic contributions is further complicated by intrinsic limitations of in situ optical measurements in deforming solid polymer networks. During uniaxial stretching, continuous changes in optical path length, along with strain localization and scattering effects, preclude the application of Beer–Lambert-type relationships required to determine absolute concentrations. Accordingly, the analysis presented here relies on relative spectral metrics (Δ*T*), which enable consistent comparison across samples subjected to identical deformation histories.

Further increasing the HEA content to 10 wt % (i.e., **P­(MA-** *co* **-10%HEA)-Tr-1**) amplified the heterolytic character of the activation, in line with our previous experiments.^23^ **P­(MA-** *co* **-10%HEA)-Tr-1** films produced a more intense blue-green color upon stretching (Figure 3g, Supporting Information Movie 3). The in situ spectra showed a dominant 630 nm band with a substantially diminished 444 nm feature (Figure 3h), suggesting predominant carbocation formation. The Δ*T* vs strain % profile (Δ*T* defined as in the case of **P­(MA-** *co* **-5%HEA)-Tr-1**) indicated activation at strain levels above ∼160% and a linear correlation with applied strain (Figure 3i).

To directly test whether the mechanochemical behavior of **Tr-1** is governed by hydrogen-bond donation rather than bulk polarity, we performed a complementary set of experiments using **P­(MA-** *co* **-MEA)-Tr-1** networks (5 and 10 wt % MEA). The hydroxyl group of HEA is chemically blocked by methylation in MEA, meaning that MEA is incapable of acting as a hydrogen-bond donor. Under uniaxial tensile deformation, both **P­(MA-** *co* **-5%MEA)-Tr-1** and **P­(MA-** *co* **-10%MEA)-Tr-1** films displayed a mechanochromic response closely resembling that of **PMA-Tr-1**, i.e., the development of an orange coloration and a dominant absorption band centered near ∼450 nm (Figure 4 for **P­(MA-** *co* **-10%MEA)-Tr-1**, Figure S44 for **P­(MA-** *co* **-5%MEA)-Tr-1**, and Supporting Information Movies 4 and 5 for **P­(MA-** *co* **-10%MEA)-Tr-1** and **P­(MA-** *co* **-5%MEA)-Tr-1**, respectively). These features are consistent with the formation of triarylmethyl radical species and indicate that homolytic bond cleavage is the predominant mechanochemical pathway in the absence of hydrogen-bond donors. We previously saw that increasing hydrogen-bond donor content promotes heterolytic cleavage in the HEA-containing networks. In the MEA-containing systems, instead, no dominant absorption at ∼630 nm was observed upon increasing the MEA loading. Thus, removal of hydrogen-bond donation restores homolytic activation of **Tr-1**. These conclusions are further supported by EPR measurements, which confirm the presence of radical species in MEA-containing networks (vide infra).

**Figure 4:**
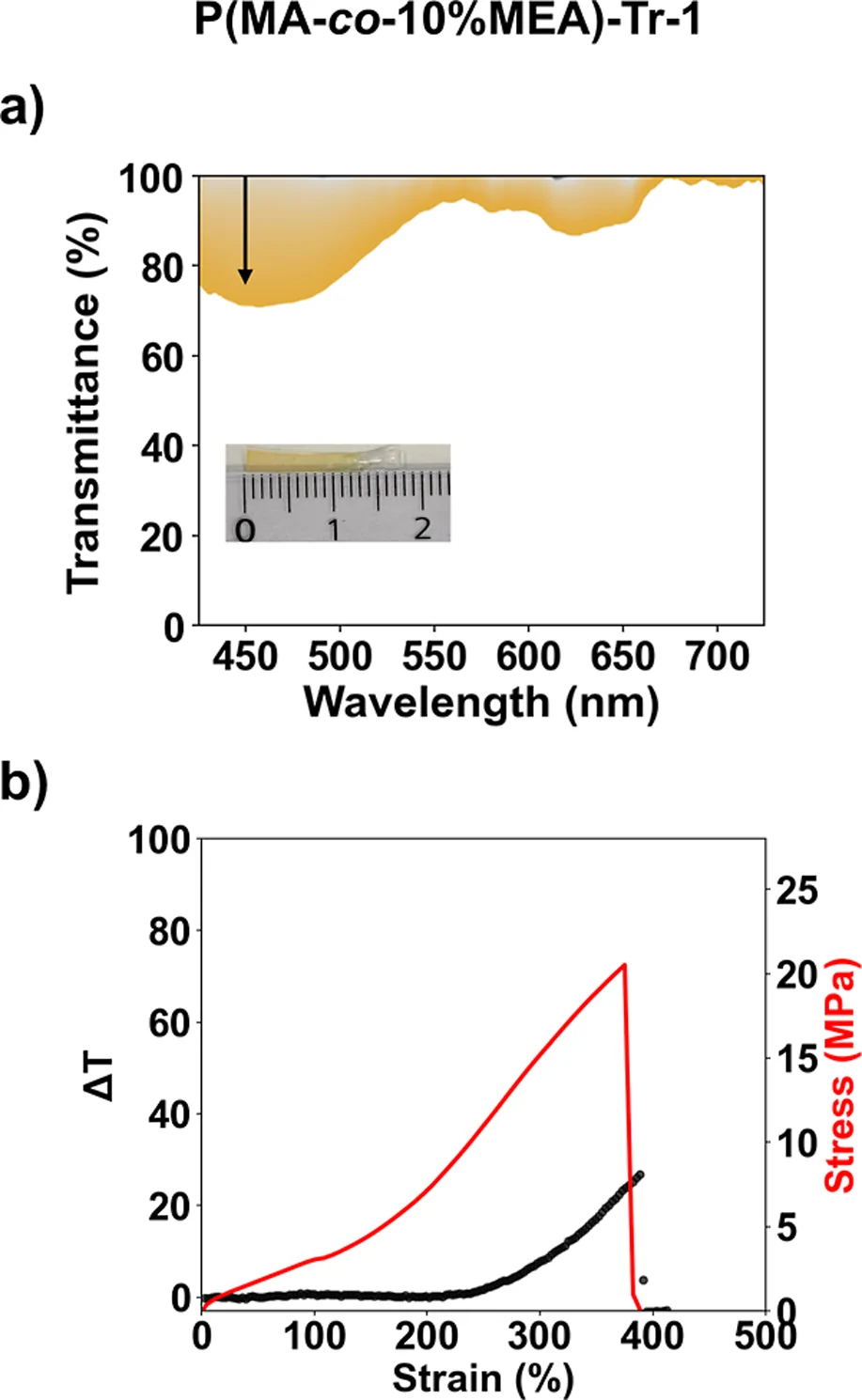
Force-triggered dissociation of **Tr-1** in **P­(MA-** *co* **-10%MEA)-Tr-1** films. (a) Transmittance spectra recorded during stretching. The black arrow shows spectra recorded at increasingly applied strain. The inset shows a photograph of a polymer film taken after tensile deformation, next to a ruler (dimensions in cm). (b) Stress–strain curve (red trace) and simultaneously measured relative transmittance changes (black trace), where ΔTransmittance = *T*(700 nm) – *T*(444 nm).

To further assess whether changes in bulk polarity could account for the different behavior of the **P­(MA-** *co* **-HEA)-Tr-1** and **P­(MA-** *co* **-MEA)-Tr-1** systems, we performed complementary solvatochromic experiments using Nile Red as a local polarity probe in PMA, P­(MA-*co*-HEA), and P­(MA-*co*-MEA) networks (Figure S41). The comparison between the emission profiles displayed only small shifts in emission maxima (ca. 7 nm) between PMA and P­(MA-*co*-HEA/MEA) networks investigated (λ_max_ = 584 nm for **PMA-Tr-1**, 587 nm for **P­(MA** *-co* **-5%HEA)-Tr-1**, 591 nm for **P­(MA-** *co* **-10%HEA)-Tr-1**, 585 nm for **P­(MA-** *co* **-5%MEA)-Tr-1**, and 586 nm for **P­(MA-** *co* **-10%MEA)-Tr-1**). This suggests that HEA- and MEA-based systems do not differ significantly in terms of the average polarity experienced by the fluorescent probe within the matrix, and that incorporating HEA or MEA at these loadings produces only modest changes in the polarity of the surrounding environment (Figure S41). In other words, the small differences in polarity across PMA, P­(MA-*co*-HEA), and P­(MA-*co*-MEA) networks are insufficient to rationalize the pronounced divergence in mechanochemical response.

Overall, the systematic study of **Tr-1** embedded in PMA and PMA-*co*-HEA/MEA networks supports the fact that local hydrogen-bonding interactions, rather than bulk dielectric constant, are key to modulating the mechanochromic response of the polymer films. These data reveal that the same mechanophore can be steered between radical and ionic outcomes in the solid state by microenvironmental hydrogen-bonding. These results also reveal a feature of **Tr-1** that was inaccessible in previous studies,^23^ which is the fact that **Tr-1** does not universally undergo heterolysis under force. Instead, it occupies a mechanistic boundary where the outcome—radical or ion formation—is dictated by the microenvironment. The earlier observation of heterolysis in **P­(MA-** *co*-**10%HEA**)^23^ thus represented only one point along a broader mechanochemical landscape, which we map here for the first time.

We next investigated the response of **Tr-2**, for which DFT predicted a preference for heterolytic cleavage even in apolar environments. The gauge section of **PMA-Tr-2** films turned blue-green upon stretching manually (Figure 5a,b, Supporting Information Movie 6), a color that matches that exhibited by mechanically activated **P­(MA-** *co* **-10%HEA)-Tr-1** films. The transmittance spectra collected during tensile deformation showed a dominant band at 630 nm, accompanied by a secondary absorption at 444 nm (Figure 5b). The Δ*T* analysis for **PMA-Tr-2** (definition of Δ*T* identical to that of **P­(MA-** *co* **-5%HEA)-Tr-1** and **P­(MA-** *co* **-10%HEA)**-Tr-1) reveals that **Tr-2** requires higher strains to activate (ca. 280%) (Figure 5c). Moreover, the extent of mechanochemical activation—inferred by the optical response—increased linearly with strain and stress (Figure 5c).

**Figure 5:**
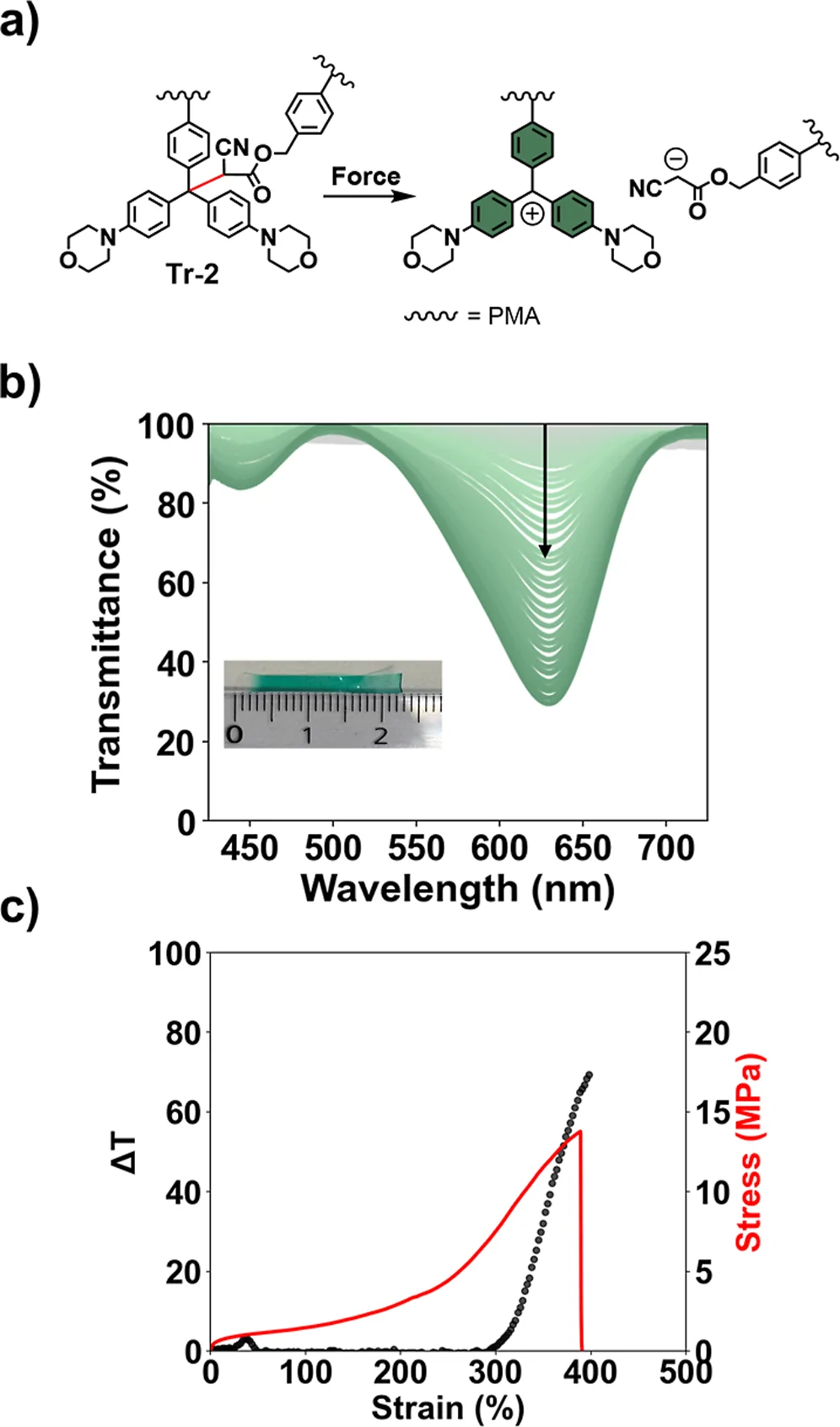
(a) Schematic representation of force-triggered heterolytic dissociation of **Tr-2** in PMA. (b) Transmittance spectra recorded during stretching **PMA-Tr-2** films. Increasing strains result in a more intense band, as highlighted by the black arrow. The insets show photographs of polymer films taken after tensile deformation, next to a ruler (dimensions in cm). (c) Stress–strain curves (red traces) and simultaneously measured relative transmittance changes (black traces) for **PMA-Tr-2**, with Δ*T* = *T*(710 nm) – *T*(630 nm).

The supposed heterolysis of **Tr-2** in **PMA-Tr-2** stands in sharp contrast to the environment-sensitive behavior of **Tr-1** and validates the computational prediction that the cyanoacetate leaving group ensures strong stabilization of charge-separated products irrespective of local hydrogen-bonding interactions.

Finally, we note that identical mechanochromic responses were observed when comparing as-prepared and extensively solvent-washed films, indicating that residual extractable species (^1^H NMR spectra of the extracts shown in Figure S43) do not contribute significantly to the observed behavior (see Figure S42).

### Electron Paramagnetic Resonance (EPR) Study

To unambiguously differentiate between homolytic and heterolytic dissociation pathways in the polymer networks, we performed electron paramagnetic resonance (EPR) spectroscopy on **PMA-Tr-1**, **P­(MA-** *co* **-5%HEA/MEA)-Tr-1**, **P­(MA-** *co* **-10%HEA/MEA)-Tr-1**, and **PMA-Tr-2** films. Each sample was analyzed under three conditions: (i) as-prepared, (ii) after tensile deformation, and (iii) after high-intensity UV irradiation for 90 min. For the latter, we used a powerful mercury lamp (200–2000 nm, irradiation power of 100 W, irradiation time of 90 min). EPR measurements were conducted at room temperature with identical sample positioning to ensure quantitative comparison.

As-prepared **PMA-Tr-1** films showed no detectable EPR signal, whereas the stretched films displayed a well-defined radical signature (Figure 6a). The observed spectrum corresponds to a carbon-centered radical with a *g*-value of ∼2.002–2.003, consistent with literature values for triarylmethyl radicals.^39,40^ Upon powerful photochemical irradiation, the same radical signal intensified significantly, suggesting that both mechanical force and light promote homolytic C–O bond cleavage in **Tr-1** when embedded in a non-hydrogen-bonding matrix. The behavior observed by EPR directly supports the optical analysis during the uniaxial deformation experiments, in which **PMA-Tr-1** exhibited a stronger absorption in the 430–450 nm region.

**Figure 6:**
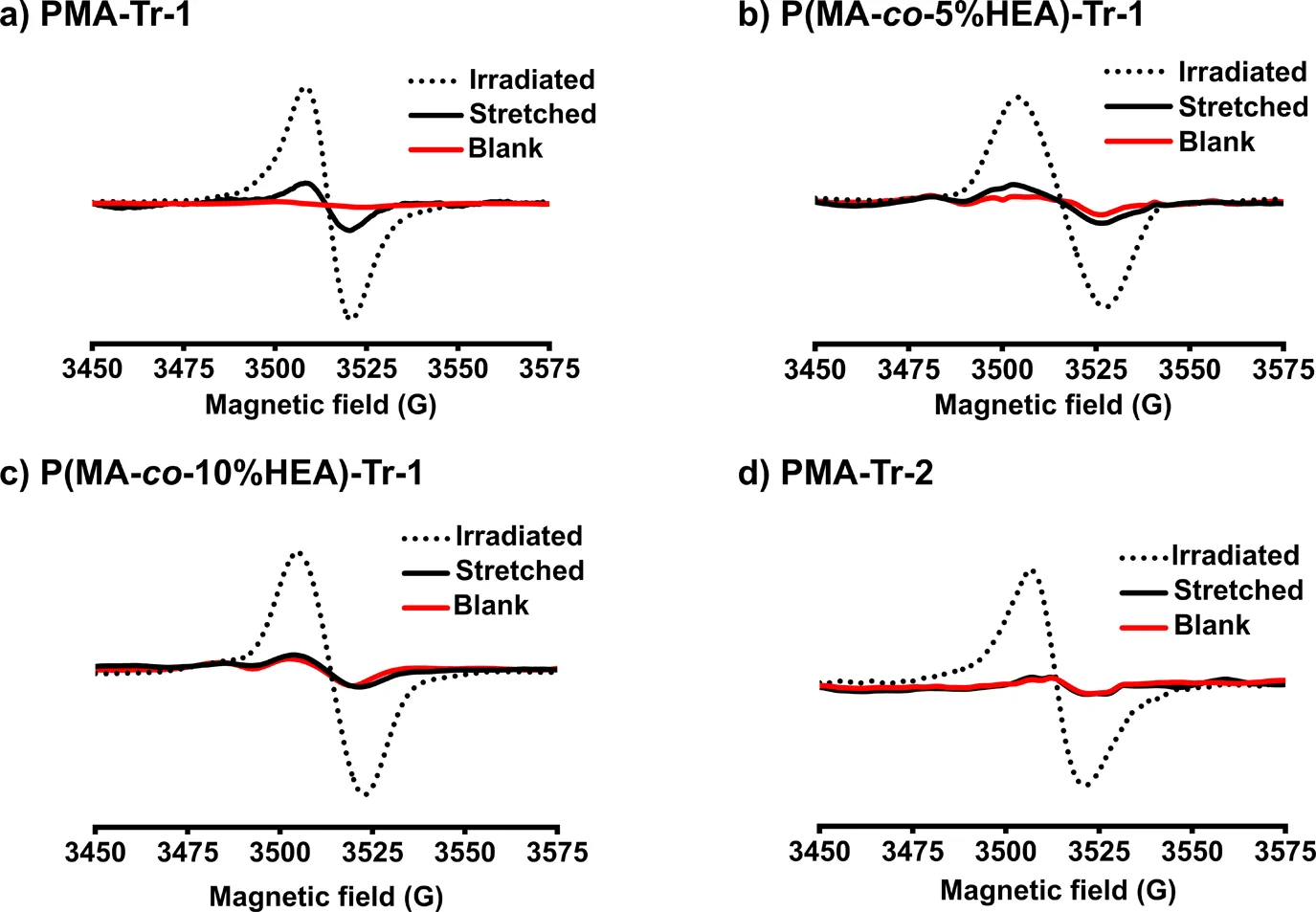
EPR spectra of polymer films (a) **PMA-Tr-1**, (b) **P**(**MA-** *co* **-5%HEA**)**-Tr-1**, (c) **P**(**MA-** *co* **-10%HEA**)**-Tr-1**, and (d) **PMA-Tr-2** before and after being manually pulled, and after irradiation with a Hg lamp (200–2000 nm, irradiation power of 100 W, irradiation time of 90 min).

In **P­(MA-** *co* **-5%HEA)-Tr-1**, mechanical stretching produced a much weaker radical signal compared to **PMA-Tr-1**, suggesting a significant suppression of the homolytic pathway (Figure 6b). The reduction in spin density also aligns with the transmittance spectra measured while pulling, which revealed a predominant carbocationic signature, although the transmittance spectra suggested the presence of additional (radical) species. Together, these observations suggest that both pathways operate concurrently at 5 wt % HEA, placing this composition near the mechanistic crossover.

Interestingly, powerful irradiation of the **P­(MA-** *co* **-5%HEA)-Tr-1** films restored a strong radical signal, demonstrating that photochemical activation proceeds via homolysis even when mechanical activation favors heterolysis. This is presumably linked to the open-shell excited states favoring homolytic cleavage to form the radical products.

No radical signal was detected before and after stretching the **P­(MA-** *co* **-10%HEA)-Tr-1** films, instead (Figure 6c). The absence of EPR activity and the observed mechanochromism (Figure 3h) confirm that mechanical force effectively induces heterolytic cleavage at this HEA content. Notably, irradiation of the same films generated a clear radical signal, revealing that powerful irradiation with light offers a complementary activation pathway that is not accessible under mechanical load in hydrogen-bond-rich environments. This divergence highlights the unique ability of mechanical force to sometimes yield distinct reaction products compared to those formed under photochemical excitation.

In line with the optical experiments, upon mechanical deformation, **P­(MA-** *co* **-MEA)-Tr-1** samples exhibit pronounced radical signals comparable to those observed in **PMA-Tr-1**, which are in stark contrast to the suppressed or absent radical signals in the corresponding HEA-containing networks. Finally, mechanical stretching of **PMA-Tr-2** produced no detectable radical signal in agreement with the heterolytic response predicted by DFT and observed experimentally (Figure 6d). Only upon irradiation did a radical signature appear, suggesting, also in this case, that the C–C bond in **Tr-2** can undergo homolytic cleavage photochemically but not mechanically. This behavior matches the DFT prediction that the cyanoacetate leaving group strongly stabilizes the heterolytic pathway, rendering the radical route inaccessible under force.

Together, the optical and EPR data establish that triarylmethanes constitute a mechanophore platform capable of tunable access to distinct mechanochemical pathways.^41^ These results significantly expand the scope of triarylmethane mechanochemistry beyond the single heterolytic pathway previously reported in PMA-*co*-10%HEA.^23^

## Conclusions

In summary, this investigation reveals a general strategy to control force-coupled reaction pathways in solid-state polymer mechanochemistry by integrating molecular design with matrix engineering. By integrating environmental effects with mechanophore rational design (e.g., **Tr-2**), we have transformed triarylmethanes from single-pathway systems into tunable platforms for controlling mechanochemical pathways in the condensed phase. Our findings reveal that subtle variations in local hydrogen-bonding environments, even within matrices of comparable bulk polarity, can bias mechanochemical outcomes, introducing a new dimension of environmental sensitivity in condensed-phase reactivity. To the best of our knowledge, this level of mechanistic control has been confined only to solution-phase systems.^21,22^

In addition to enabling pathway switching, our study uncovered two broader advances. First, we have demonstrated that triarylmethane radicals can be generated and stabilized in cross-linked polymer networks, providing rare experimental access to these reactive species in the solid state. Building on this first aspect, we have shown that triarylmethane ethers can also serve as mechanochemical molecular probes of microenvironmental polarity, as they are capable of reporting on hydrogen-bonding interactions through distinct mechanochromic signatures. These insights expand the conceptual framework of polymer mechanochemistry and suggest design principles for next-generation force-responsive materials that couple mechanical inputs to programmable chemical outputs.

## Supplementary Material















## Notes

Synthetic procedures, compound characterization (^1^H and ^13^C NMR spectra, FT-IR spectra, mass characterization, and UV–vis spectra), and additional discussion on thermal and mechanical characterization of polymer networks incorporating **Tr-1** and **Tr-2**. Swelling percentage and gel content data, and DMA characterization for all synthesized polymers. Atomic coordinates of all calculated structures. (PDF)
A collection of all computational data has been published in the^42^ ioChem-BD repository and can be accessed at DOI: 10.19061/iochem-bd-1-341 . Videos showing the mechanical activation of all polymers by pulling manually (Supporting Information Movies 1–6). Mechanochemical activation upon manual deformation of PMA-Tr-1 (MP4)
Mechanochemical activation upon manual deformation of P­(MA-co-5%HEA)-Tr-1 (MP4)
Mechanochemical activation upon manual deformation of P­(MA-co-10%HEA)-Tr-1 (MP4)
Mechanochemical activation upon manual deformation of P­(MA-co-10%MEA)-Tr-1 (MP4)
Mechanochemical activation upon manual deformation of P­(MA-co-5%MEA)-Tr-1 (MP4)
Mechanochemical activation upon manual deformation of PMA-Tr-2 (MP4)
