# Supplementary material for: Controlling Solid-State Mechanochemical Pathways in Triarylmethane Mechanophores via Molecular and Matrix Design
Source: Macromolecules. 2026 Jul 13;59(16):9065–75. doi: 10.1021/acs.macromol.6c00789 (PMC13557437; doi:10.1021/acs.macromol.6c00789)
Supplement: Supplementary file 1 [file ma6c00789_si_001.pdf]

Supporting information for

Controlling Solid-State Mechanochemical Pathways in Triarylmethane Mechanophores via Molecular and Matrix Design

Dnyaneshwar Ajinath Mache,[a,b] Eduardo Garcia-Padilla,[a,b] Federico Frateloreto,[a] Gaia Egizzo,[a,b] James R. Hemmer,[c] Javier G. Valverde-Bastidas,[d] Silvia De la Flor,[d] Feliu Maseras,[a] José Augusto Berrocal[a,c,e]*

[a] Institute of Chemical Research of Catalonia (ICIQ), Barcelona Institute of Science and Technology (BIST), Tarragona 43007, Spain

[b] Universitat Rovira i Virgili, Departament de Química Orgánica, Tarragona 43007, Spain

[c] Adolphe Merkle Institute, Chemin des Verdiers 4, CH-1700 Fribourg, Switzerland

[d] Universitat Rovira i Virgili, Department of Mechanical Engineering, Tarragona 43007, Spain

[e] ICREA, Pg. Lluís Companys 23, 08010 Barcelona, Spain

* To whom correspondence should be addressed: jberrocal@iciq.es

Contents

1. General Information S3

2. Experimental Section S5

2.1 Synthesis of bis(4-morpholinophenyl) methanone (**10**) S5

2.2 Synthesis of bis(4-morpholinophenyl) (4-vinylphenyl) methanol (**3**) S5

2.3 Synthesis of 4,4'-((((4-vinylbenzyl) oxy) (4-vinylphenyl) methylene) bis(4,1-phenylene)) dimorpholine (**Tr-1**) S6

2.4 Synthesis of bis(4-morpholinophenyl)(4-vinylphenyl)methylium trifluoro(hydroxy)borate salt (**4**).. S6

2.5 Synthesis of 4-vinylbenzyl 2-cyanoacetate (**7**) S7

2.6 Synthesis of 4-vinylbenzyl 2-cyano-3,3-bis(4-morpholinophenyl)-3-(4-vinylphenyl) propanoate (**Tr-2**) S7

2.7 Synthesis of crosslinked polymer films by incorporating **Tr-1** and **Tr-2** S8

2.7.1. Synthesis of **PMA-Tr-1** S8

2.7.2 Synthesis of **P(MA-*co*-5%HEA)-Tr-1** S8

2.7.3 Synthesis of **P(MA-*co*-10%HEA)-Tr-1** S9

2.7.4 Synthesis of **P(MA-*co*-5%MEA)-Tr-1** S9

2.7.5 Synthesis of **P(MA-*co*-10%MEA)-Tr-1** S10

2.7.6 Synthesis of **PMA-Tr-2** S10

3. Nuclear magnetic resonance (NMR) data S11

4. Fourier Transform Infrared (FT-IR) Spectroscopy S17

5. High-Resolution Mass Spectrometry (HR-MS) Characterization S21

6. UV–Visible Spectroscopy Analysis S24

7. Thermal characterization of PMA and PMA-*co*-HEA/MEA polymer networks S25

7.1 Thermogravimetric Analysis (TGA) S25

7.2 Differential Scanning Calorimetry (DSC) S28

8. Swelling Index (SI) and Gel Content (GC) Calculation: S32

9. Dynamic Mechanical Analysis (DMA) of cross-linked polymer films S33

10. Mechanical activation of crosslinked polymer films and their dissociation mechanism S35

11. UV–vis spectrum of **4**. S36

12. Solvatochromism of Nile Red in **PMA-Tr-1** and **P(MA-*co*-HEA/MEA)-Tr-1** networks S37

13. Evaluation of Residual Monomer and Mechanophore Content and the Mechanical Response of Washed Polymer Films S38

14. Mechanochromic behavior of **P(MA-*co*-5%MEA)-Tr-1** S40

15. Electron paramagnetic resonance characterization of P(MA-*co*-MEA) copolymers S41

16. References S42

17. Atomic coordinates of the calculated structures S43

1. General Information

Materials. All reagents were used as purchased. Anhydrous solvents were purified using a PureSolv™ system. TLC (thin layer chromatography) was performed on silica gel plates and visualized under UV light. Flash chromatography was carried out on silica gel or using a Biotage Selekt system with prepacked silica columns. Moisture-sensitive reactions were conducted in dried Schlenk glassware under an argon or nitrogen atmosphere. Yields refer to isolated, purified compounds.

Nuclear Magnetic Resonance (NMR) Spectroscopy. NMR spectra were recorded at 298 K on a Bruker Avance 300 and Bruker Avance 400 or 500 ultra-shield spectrometers. Data were processed using MestReNova (v 12.0). Chemical shifts (δ) are reported in parts per million (ppm) relative to the residual peak of the solvent, and coupling constants (*J*) are reported in hertz (Hz). Signal multiplicities are denoted as follows: s = singlet, br s = broad singlet, d = doublet, t = triplet, q = quartet, m = multiplet, app = apparent.

Fourier Transform Infrared (FT-IR) Spectroscopy. Infrared spectra were recorded using a Cary 630 FT-IR spectrometer, and the resulting data were analysed and plotted using ACD/Lab Specman software.

Mass spectrometry (MS). Mass spectra were recorded on a Waters LCT Premier spectrometer using electrospray ionization (ESI) with a time-of-flight (TOF) mass analyzer.

UV-Vis spectroscopy. UV-vis spectra were acquired on a Shimadzu Corporation UV-1800PC spectrophotometer. Data were plotted using Origin 2017.

Photopolymerization Conditions. UV irradiation was performed using a Dr. Hönle LED Cube 100 UV curing system operating at 365 nm. Samples were irradiated for a total duration of approximately 7 min. Photopolymerization was conducted using a two-step irradiation protocol: first, samples were exposed at 50% output power for a time of 2 min (12 bursts of 10 s each), with 20–30 s pauses between bursts; this was followed by irradiation at 20% output power for a time of 5 min (15 bursts of 20 s each), with 20–30 s pauses between bursts.

Electron Paramagnetic Resonance (EPR) Spectroscopy. EPR spectra were recorded on a Bruker EMX Micro X-band spectrometer operating at 9.85 GHz using a Bruker ER 1164 HS resonator. Polymer films (thickness < 0.5 mm, length 2.5 cm) were loaded into individual EPR tubes and positioned identically within the resonant cavity for all measurements. Spectra were acquired at room temperature under the following conditions: microwave power, 0.6402 mW; center field, 3516 G; sweep width, 600 G; sweep time, 30 s; modulation frequency, 100 kHz; modulation amplitude, 1 G; power attenuation, 25 dB; time constant, 0.01 ms; conversion time, 5 ms; and gain, 30 dB. UV irradiation was performed using a mercury lamp (λ = 200–2000 nm) at 100 W for 90 minutes.

Thermogravimetric analyses (TGA) were performed using a Mettler–Toledo TGA/SDTA851 instrument. Samples were heated from 30 to 600 °C at 10 °C min^{-1} under a nitrogen atmosphere with a flow rate of 40 mL min^{-1}. The resulting data were analysed using STARe Evaluation software.

Differential scanning calorimetry (DSC) was conducted under nitrogen using a Mettler–Toledo DSC822e instrument. Measurements were performed over a temperature range of −80 to 200 °C at heating and cooling rates of 10 °C min^{-1}. DSC data were analysed using the STARe Evaluation software.

Dynamic mechanical analysis (DMA) was performed using a Discovery DMA 850 (TA Instruments) equipped with a film tension clamp and with an ACS3+ cooling system. Prismatic rectangular samples with dimensions around 15 x 5 x 0.2 mm^3 were analyzed from -50 °C to 125 °C at 1 Hz, with 0.02 % of strain at a heating rate of 2 °C min^{-1}.

Tensile testing. Tensile tests were conducted on a Linkam MFS micro tensile stage equipped with a 200 N load cell and controlled by the accompanying Linksys32 software. Tests were carried out at room temperature with a strain rate of 2.20 % s^{-1} (= 375 µm s^{-1}) on rectangular films with a length of ca. 30 mm, a width of ca. 5 mm, and a thickness of ca. 200 µm.

In situ transmittance measurements. Optical characterization was performed using an optical microscope (Axioscope 5, Carl Zeiss Microscopy GmbH, Germany) equipped with a white-light LED source (nominal electrical power: 10 W). For in situ transmittance measurements, the light transmitted through the sample was collected using an optical fiber (P200-2-VIS-NIR, 200 µm core; Ocean Insight, Orlando, FL, USA) and analysed with a Maya 2000-Pro spectrometer (Ocean Insight, Orlando, FL, USA). The measurement spot size was approximately 200 µm, determined with a 20× objective with a numerical aperture of 0.22 (Zeiss EC Epiplan Neofluar 20×/0.22). Transmission spectra were recorded by the spectrometer and normalized to the spectrum of the unstretched sample.

Data Processing and Plotting. All transmittance and tensile data were processed and plotted using custom Python scripts executed in the Spyder software.

Computational details. All calculations were carried out using Gaussian09.[S1] Geometry optimizations were performed with B3LYP[S2–4] and Grimme's D3 dispersion correction,[S5] with the 6-31G(d) basis set.[S6] Accurate energies were obtained from single-point calculations with B3LYP-D3 and 6-311+G(d,p). The solvation environment was modelled with the implicit polarizable continuum model (PCM) in all calculations,[S7] using ethanol, methyl propanoate, methyl butanoate, or an approximation of poly(methyl methacrylate) using Eps=3.9 (static dielectric constant) and EpsInf=2.222 (optical dielectric constant) from the square of the refractive index.[S8] All stationary points were characterized by the absence of imaginary vibrations. The reported free energies were calculated at 298 K and 1 M.

2. Experimental Section

2.1 Synthesis of bis(4-morpholinophenyl) methanone (**10**)

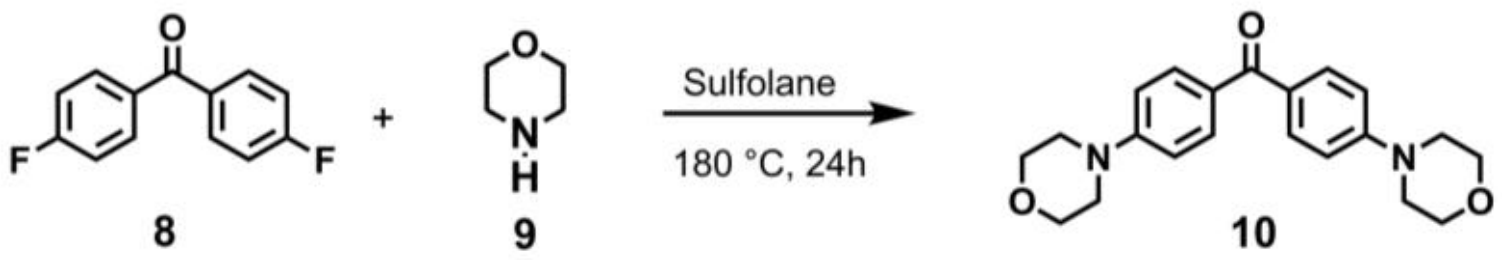

4,4'-difluorobenzophenone (**8**; 10 g; 46 mmol; 1 equivalent) and morpholine (**9**; 16 g; 184 mmol; 4 equivalents) were loaded into a round-bottom flask equipped with a stirring bar. Sulfolane (30 mL) was added. The reaction mixture was heated to reflux at 180 °C for 24 h, and its progress was monitored by TLC using ethyl acetate: *n*-hexane (4:1, v/v) as the eluent. The reaction mixture was cooled to room temperature and poured into ice-cold water (500 mL). A brown solid precipitated, which was collected by filtration. The crude product was dissolved in ethyl acetate (20 mL) and reprecipitated. This procedure was repeated twice. The solid was filtered and dried under high vacuum. Compound **10** was isolated as a yellow solid (13.4 g; 83% yield).

^{1}H NMR (500 MHz, $CDCl_3$) δ 7.81–7.72 (m, 4H), 6.95–6.86 (m, 4H), 3.92–3.84 (m, 8H), 3.35–3.26 (m, 8H).
^{13}C NMR (125 MHz, $CDCl_3$) δ 193.4, 153.0, 131.4, 128.3, 112.7, 66.0, 47.2.
FT-IR (cm^{-1}) 2966 (C-H stretch), 2829 (C-H stretch), 1630, 1593, 1513, 1375, 1315,1228, 1179, 1118, 920, 831, 765, 680.
HR-MS (*m/z*): $[M]+H^+$ calcd for $C_{21}H_{25}N_2O_3$ 353.1860; found 353.1870.

2.2 Synthesis of bis(4-morpholinophenyl) (4-vinylphenyl) methanol (**3**)

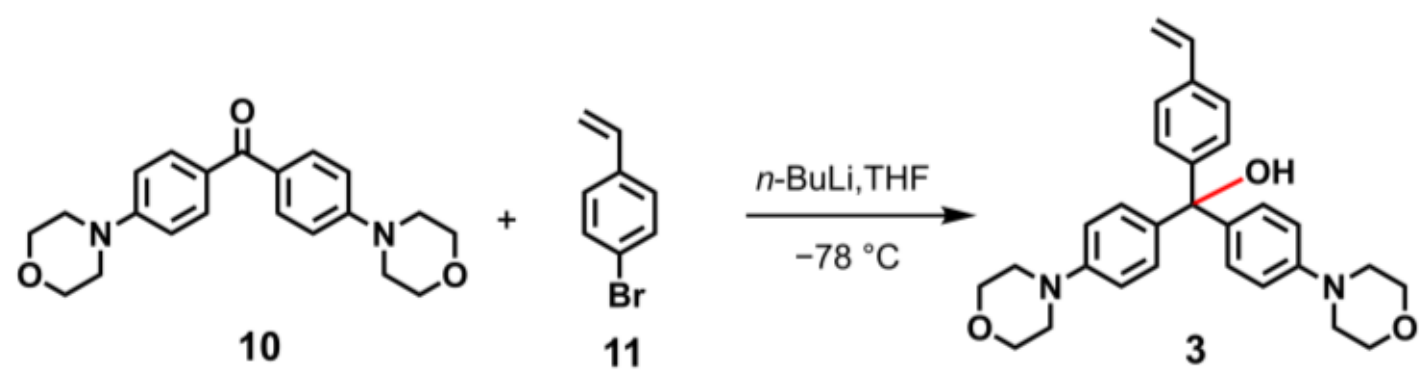

4-vinylbromobenzene (**11**; 0.67 mL; 5.1 mmol; 1.5 equivalent) and anhydrous THF (15 mL) were added to a two-necked round-bottom flask equipped with a stirring bar under a nitrogen atmosphere. The reaction mixture was cooled to −78 °C using an ethyl acetate/liquid nitrogen bath. A solution of *n*-BuLi in hexane (2.5 M; 2 mL; 5.1 mmol; 1.5 equivalent) was added dropwise, and the mixture was stirred for 5 min at −78 °C. A solution of compound **10** (1.2 g; 3.4 mmol, 1 equivalent) in dry THF (10 mL) was then added dropwise, and stirring was continued for an additional 10 min. The reaction was quenched with saturated aqueous sodium bicarbonate, followed by water. The mixture was extracted twice with ethyl acetate, and the combined organic layers were dried over anhydrous Na_2SO_4. Solvent removal under reduced pressure afforded a residue, which was dissolved in a minimal amount of ethyl acetate and reprecipitated by the slow addition of hexane until turbidity persisted. The suspension was left to stand overnight. The resulting solid was collected by filtration, washed with hexane, and dried under vacuum to afford compound **3** as white crystals (1.2 g; 77% yield).

^{1}H NMR (500 MHz, $CDCl_3$) δ 7.36–7.32 (m, 2H), 7.26–7.23 (m, 2H), 7.17–7.13 (m, 4H), 6.86–6.80 (m, 4H), 6.70 (dd, J = 17.6, 10.9 Hz, 1H), 5.73 (dd, J = 17.6, 1.0 Hz, 1H), 5.23 (dd, J = 10.9, 1.0 Hz, 1H), 3.87 – 3.82 (m, 8H), 3.19 – 3.11 (m, 8H), 2.69 (s, 1H).
^{13}C NMR (75 MHz, $CDCl_3$) δ 149.7, 146.6, 138.1, 136.0, 135.8, 128.4, 127.5, 125.2, 114.2, 113.4, 80.8, 66.4, 48.6.

FT-IR (**cm**$^{-1}$) 3395 (O-H stretch), 2961 (C-H stretch), 2806 (C-H stretch), 1606, 1507, 1444, 1233, 1110, 918, 818.
HR-MS (**m/z**): $[M+H]^+$ calcd for $C_{29}H_{33}N_2O_3$ 457.2486; found 457.2496.

2.3 Synthesis of 4,4'-((((4-vinylbenzyl) oxy) (4-vinylphenyl) methylene) bis(4,1-phenylene)) dimorpholine (**Tr-1**)

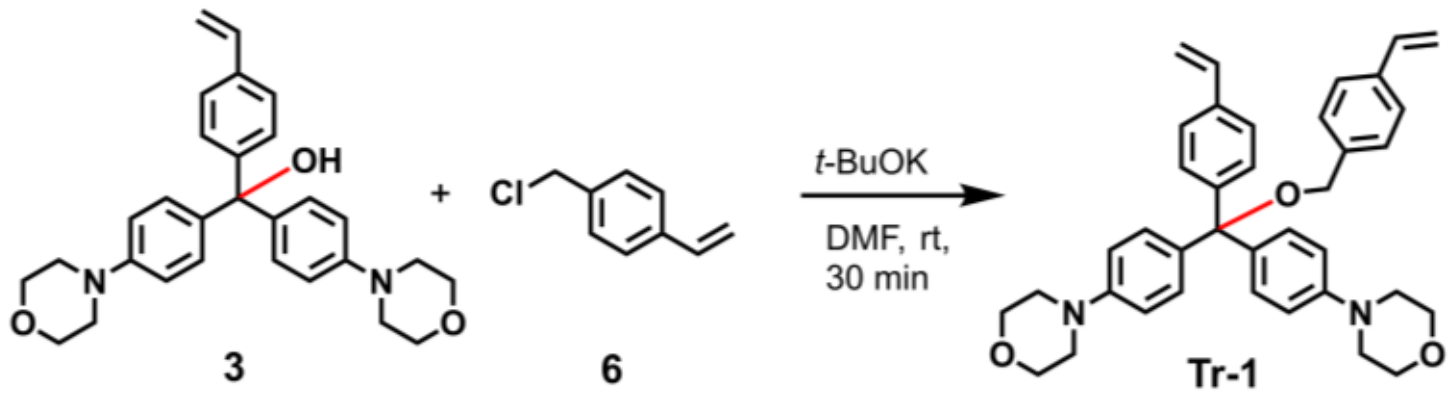

Compound **3** (300 mg; 657 µmol; 1 equivalent) was dissolved in dry DMF (4 mL) in a two-necked round-bottom flask equipped with a stirring bar under a nitrogen atmosphere. Potassium *tert*-butoxide (*t*-BuOK; 111 mg; 986 µmol; 1.5 equivalents) was added, followed by 4-vinylbenzyl chloride (**6**; 139 µL; 150 mg; 986 µmol; 1.5 equivalents; density = 1.083 g/mL at 25°C), and the reaction mixture was stirred at room temperature for 10 minutes. Reaction progress was monitored by TLC, which indicated the presence of unreacted starting material. Therefore, an additional 0.5 equivalents of *t*-BuOK (36.9 mg; 329 µmol) and 4-vinylbenzyl chloride (46 µL; 50.1mg; 329 µmol) were added. Upon completion, DMF was removed by rotatory evaporation, and the crude compound was purified by column chromatography on silica gel (gradient elution from 30% to 40% ethyl acetate in *n*-hexane). The product was dried under vacuum to afford **Tr-1** as a white foam (310 mg; 82% yield).

^{1}H NMR (400 MHz, $CDCl_3$) δ 7.47 (d, *J* = 8.3 Hz, 2H), 7.36 (ddt, *J* = 13.6, 8.3, 4.7 Hz, 10H), 6.85–6.82 (m, 4H), 6.69 (ddd, *J* = 17.7, 14.1, 10.9 Hz, 2H), 5.72 (ddd, *J* = 17.7, 11.1, 1.0 Hz, 2H), 5.21 (ddd, *J* = 10.8, 7.2, 1.0 Hz, 2H), 4.16 (s, 2H), 3.86–3.83 (m, 8H), 3.17–3.14 (m, 8H).
^{13}C NMR (101 MHz, $CDCl_3$) δ 149.7, 144.9, 138.9, 136.5, 136.3, 136.2, 135.7, 134.9, 129.6, 128.1, 126.9, 125.9, 125.9, 125.5, 114.3, 113.2, 86.1, 66.7, 65.1, 48.9.
FT-IR (cm^{-1}): 2952 (C-H stretch), 2925 (C-H stretch), 2851, 1576,1421, 1379, 1237, 1181, 1032, 924, 825, 706.
HR-MS (**m/z**): $[M+H]^+$ calcd for $C_{38}H_{41}N_2O_3$ 573.3112; found 573.3128.

2.4 Synthesis of bis(4-morpholinophenyl)(4-vinylphenyl)methylium trifluoro(hydroxy)borate salt (**4**)

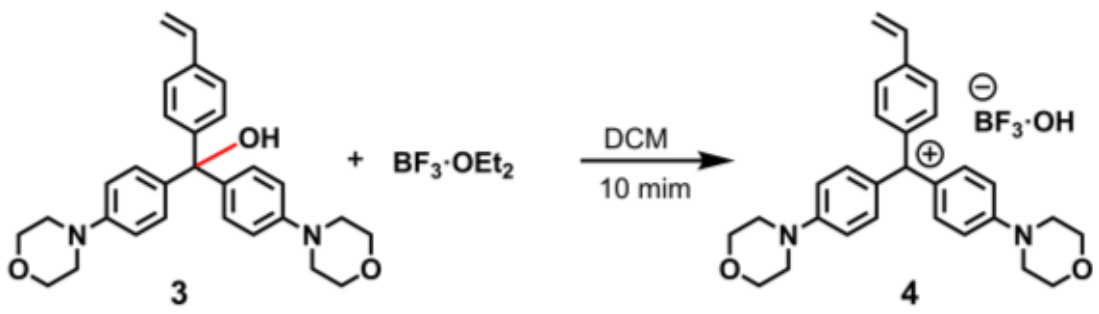

In two-neck round-bottom flask, compound **3** (300 mg; 657 µmol; 1 equivalent) was dissolved in dichloromethane (30 mL) at room temperature. $BF_3{\cdot}OEt_2$ (203 mg (46%); 657 µmol; 1 equivalent) was added dropwise, which immediately yielded a deep green solution. The reaction mixture was stirred for 20 min, after which diethyl ether (50 mL) was added dropwise, yielding a dark green precipitate. The solid was collected by filtration, washed with diethyl ether, and dried under vacuum to afford compound **4** as a dark-green powder (274 mg; 95% yield)

^{1}H NMR (300 MHz, CD_3CN) δ 7.70 (d, *J* = 8.2 Hz, 2H), 7.46 (d, *J* = 9.4 Hz, 4H), 7.37 (d, *J* = 8.3 Hz, 2H), 7.14 (d, *J* = 9.5 Hz, 4H), 6.94 (dd, *J* = 17.7, 11.0 Hz, 1H), 6.10 (dd, *J* = 17.7, 0.7 Hz, 1H), 5.54 (dd, *J* = 10.9, 0.7 Hz, 1H), 3.97 – 3.79 (m, 8H), 3.76 – 3.65 (m, 8H).
^{13}C NMR (75 MHz, CD_3CN) δ 178.5, 157.8, 143.5, 142.0, 139.8, 136.7, 136.5, 129.1, 127.2, 118.5, 115.0, 67.0, 48.2
FT-IR (cm^{-1}) 2966 (C-H stretch), 2855 (C-H stretch), 1574, 1379, 1239, 1181, 1032, 924, 821.
HR-MS (m/z): $[M+H]^+$ calcd for $C_{29}H_{31}N_2O_2$ 439.2380; found 439.2370

2.5 Synthesis of 4-vinylbenzyl 2-cyanoacetate (**7**)

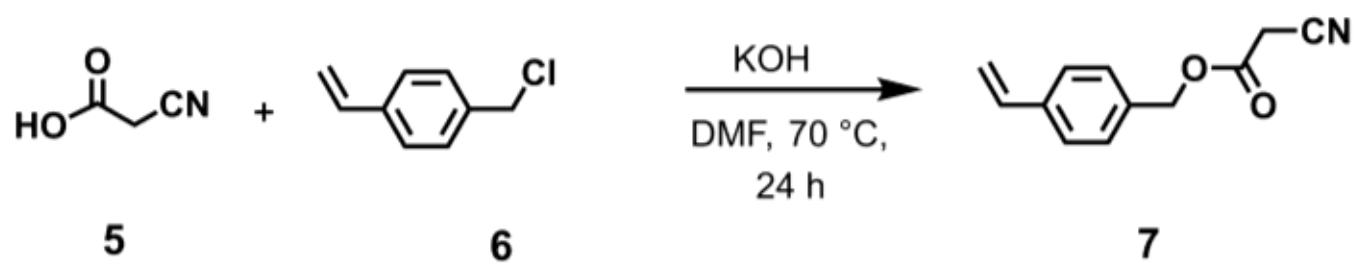

In a two-necked round-bottom flask, cyanoacetic acid **5** (3.0 g; 35 mmol; 1 equivalent) and DMF (30 mL) were combined. Potassium hydroxide (2.0 g; 35 mmol; 1 equivalent) was then added, and the reaction mixture was heated at 60 °C for 40 min. Subsequently, 4-vinylbenzyl chloride **6** (5 mL; 5.4 g; 35 mmol;1 equivalent; density = 1.083 g/mL at 25°C) was added, and the mixture was heated at 70 °C for 24 h. After cooling to room temperature, the reaction mixture was poured into ice-cold water and extracted with diethyl ether. The combined organic layers were dried over $MgSO_4$ and filtered, and the solvent was removed under reduced pressure. The crude product was purified by column chromatography on silica gel using dichloromethane/*n*-hexane (9:1, v/v) as the eluent to afford **7** as a white solid (5.25 g; 74% yield).

^{1}H NMR (500 MHz, $CDCl_3$) δ 7.45–7.40 (m, 2H), 7.36–7.30 (m, 2H), 6.72 (dd, *J* = 17.6, 10.9 Hz, 1H), 5.78 (dd, *J* = 17.7, 0.8 Hz, 1H), 5.29 (dd, *J* = 11.0, 0.8 Hz, 1H), 5.21 (s, 2H), 3.48 (s, 2H).
^{13}C NMR (125 MHz, $CDCl_3$) δ 162.2, 137.6, 135.5, 133.1, 128.3, 125.9, 114.2, 112.2, 67.7, 24.2.
FT-IR (cm^{-1}) 2961 (C-H stretch), 2924 (C-H stretch), 2260 (CN), 1736 (C=O), 1382, 1330, 1190, 987, 916, 825.
HR-MS (m/z): $[M+Na]^+$ calcd for $C_{12}H_{11}NO_2Na$ 224.0682; found 224.0677

2.6 Synthesis of 4-vinylbenzyl 2-cyano-3,3-bis(4-morpholinophenyl)-3-(4-vinylphenyl) propanoate (**Tr-2**)

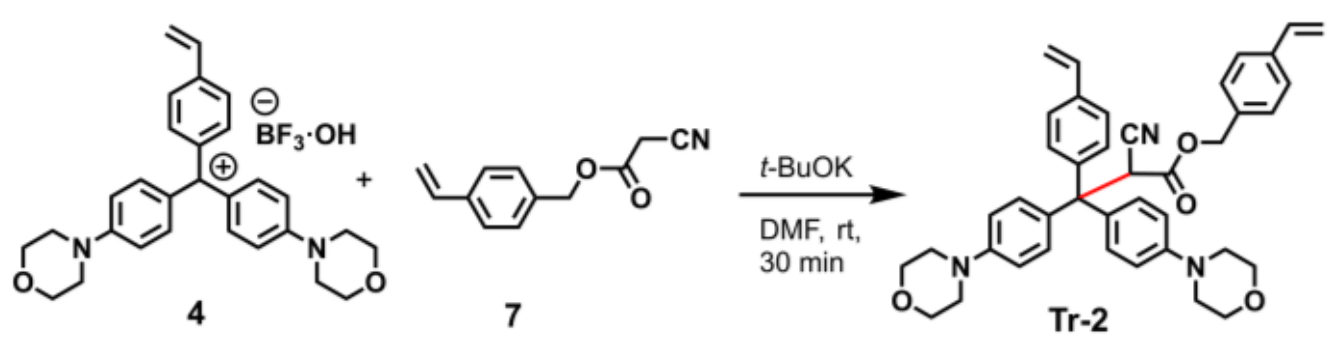

Compound **7** (119 mg; 591 µmol; 1.3 equivalent) was dissolved in dry DMF (10 mL) in a round-bottom flask equipped with a stirring bar under a nitrogen atmosphere. Potassium *tert*-butoxide (*t*-BuOK; 66.4 mg; 591 µmol; 1.3 equivalent) was added, and the mixture was stirred at room temperature for 15 min. In parallel, compound **4** (200 mg; 455 µmol; 1 equivalent) was dissolved in dry DMF (3 mL) in a separate round-bottom flask under a nitrogen atmosphere. This solution was then added dropwise to the reaction mixture via cannula with continuous stirring under nitrogen. After 30 minutes, the reaction was quenched with saturated aqueous sodium bicarbonate. The mixture was extracted with ethyl acetate. The combined organic layers were dried over anhydrous Na_2SO_4 and filtered, and the solvent was removed under reduced

pressure. The crude product was purified by column chromatography (Biotage) on silica gel using a gradient of 30–40% ethyl acetate in *n*-hexane as the eluent to afford **Tr-2** as a white solid (190 mg; 65% yield).

^{1}H NMR (400 MHz, $CDCl_3$) δ 7.31 (d, *J* = 8.0 Hz, 2H), 7.27 (s, 4H), 7.18 (dd, *J* = 9.1, 3.0 Hz, 4H), 7.03 (d, *J* = 8.2 Hz, 2H), 6.75 (d, *J* = 9.1 Hz, 4H), 6.71–6.61 (m, 2H), 5.91–5.63 (m, 2H), 5.26 (dd, *J* = 15.7, 10.4 Hz, 2H), 5.20 (s, 1H), 4.91 (d, *J* = 12.4 Hz, 1H), 4.80 (d, *J* = 12.3 Hz, 1H), 3.83 (t, *J* = 5.3 Hz, 8H), 3.12 (t, *J* = 4.9 Hz, 8H).
^{13}C NMR (101 MHz, $CDCl_3$) δ 164.6, 149.8, 143.4, 137.8, 136.4, 136.3, 134.3, 133.9, 130.3, 130.1, 129.3, 128.8, 126.4, 125.9, 116.6, 114.6, 114.5, 114.3, 68.0, 67.0, 59.0, 48.8, 48.0.
FT-IR (cm^{-1}) 2894 (C-H stretch), 2860 (C-H stretch), 2247 (CN), 1742 (C=O), 1608,1457,1120.
HR-MS (m/z): $[M+H]^+$ calcd for $C_{41}H_{42}N_3O_4$ 640.3170; found 640.3177
UV-Vis: λ_{max} = 255 nm, and molar extinction coefficient (ε) = 54622 L mol^{-1} cm^{-1}

2.7 Synthesis of crosslinked polymer films by incorporating **Tr-1** and **Tr-2**

2.7.1. Synthesis of **PMA-Tr-1**

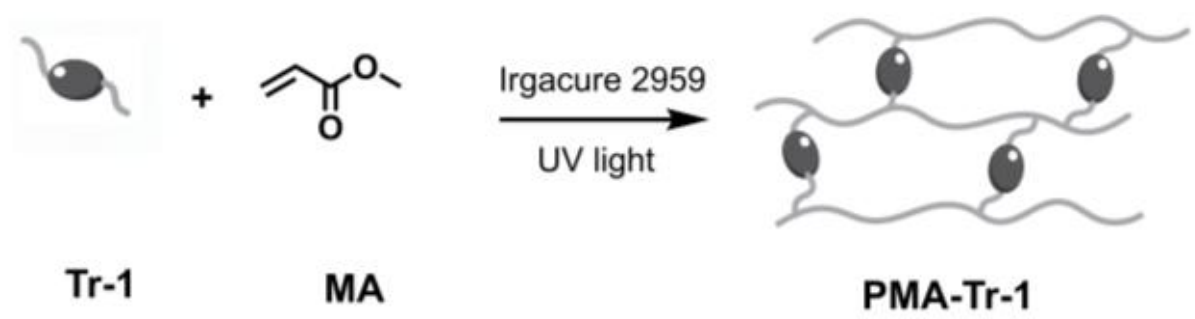

Irgacure 2959 (1 mg; 0.00446 mmol), **Tr-1** (68 mg; 0.118 mmol), and methyl acrylate (**MA**) (1.07 mL; 11.8 mmol; density = 0.956 g/mL at 25°C) were combined in a vial, and the mixture was purged with nitrogen for 5 min. Two glass microscope slides were covered with clear tape, and two pieces of folded parafilm were placed between the slides to act as 200 µm spacers. The slides were clamped together using office clamps, and the monomer solution was introduced into the gap with a pipette. Polymerization was carried out in a 365 nm LED light box (Dr. Hönle LED Cube). Samples were exposed at 50% output power for a time of 2 min (12 bursts of 10 s each), with 20–30 s pauses between bursts; this was followed by irradiation at 20% output power for a time of 5 min (15 bursts of 20 s each), with 20–30 s pauses between bursts. The slides were then carefully separated, and the resulting polymer film was dried in a vacuum oven at 60 °C overnight.

2.7.2 Synthesis of **P(MA-*co*-5%HEA)-Tr-1**

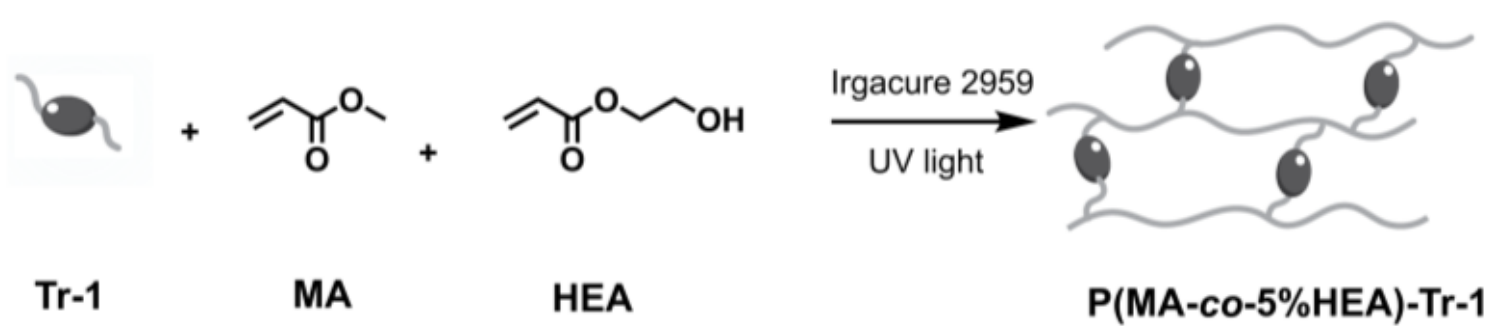

Irgacure 2959 (1 mg; 0.00446 mmol), **Tr-1** (68 mg; 0.118 mmol), methyl acrylate (**MA**; 1.04 mL; 11.04 mmol; density = 0.956 g/mL at 25°C), and hydroxyethyl acrylate (**HEA**; 47 µL; 0.448 mmol; density = 1.011 g/mL at 25°C) were combined in a vial, and the mixture was purged with nitrogen for 5 min. Two glass microscope slides were covered with clear tape, and two pieces of folded parafilm were placed between them as spacers. The slides were clamped together using office clamps, and the monomer solution was introduced into the gap with a pipette. Polymerization was carried out in a 365 nm LED light box (Dr. Hönle LED Cube). Samples were exposed at 50% output power for a time of 2 min (12 bursts of 10 s each), with 20–30 s pauses between bursts; this was followed by irradiation at 20% output power for a time of 5 min (15 bursts of 20 s each), with 20–30 s pauses between bursts. The slides were carefully separated, and the resulting polymer film was dried in a vacuum oven at 60 °C overnight.

2.7.3 Synthesis of **P(MA-*co*-10%HEA)-Tr-1**

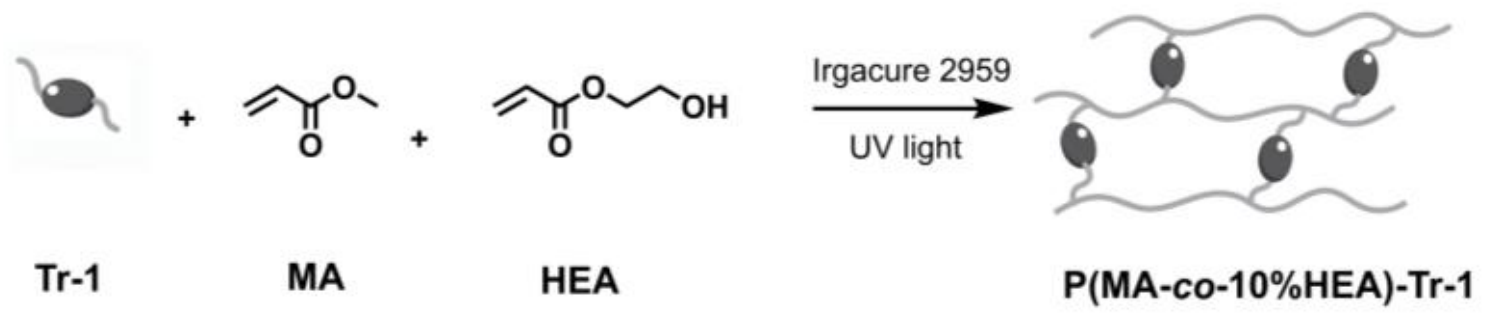

Synthesis of **P(MA-*co*-10%HEA)-Tr-1** was performed following the procedure previously described by Hemmer *et al* (2021).[S9]

Irgacure 2959 (1 mg; 0.00446 mmol), **Tr-1** (68 mg; 0.118 mmol), methyl acrylate (**MA**; 1 mL; 11.04 mmol; density = 0.956 g/mL at 25°C), and hydroxyethyl acrylate (**HEA**; 95 µL; 0.904 mmol; density = 1.011 g/mL at 25°C) were combined in a vial, and the mixture was purged with nitrogen for 5 min. Two glass microscope slides were covered with clear tape, and two pieces of folded parafilm were placed between them as spacers. The slides were clamped together using office clamps, and the monomer solution was introduced into the gap with a pipette. Polymerization was carried out in a 365 nm LED light box (Dr. Hönle LED Cube). Samples were exposed at 50% output power for a time of 2 min (12 bursts of 10 s each), with 20–30 s pauses between bursts; this was followed by irradiation at 20% output power for a time of 5 min (15 bursts of 20 s each), with 20–30 s pauses between bursts. The slides were carefully separated, and the resulting polymer film was dried in a vacuum oven at 60 °C overnight.

2.7.4 Synthesis of **P(MA-*co*-5%MEA)-Tr-1**

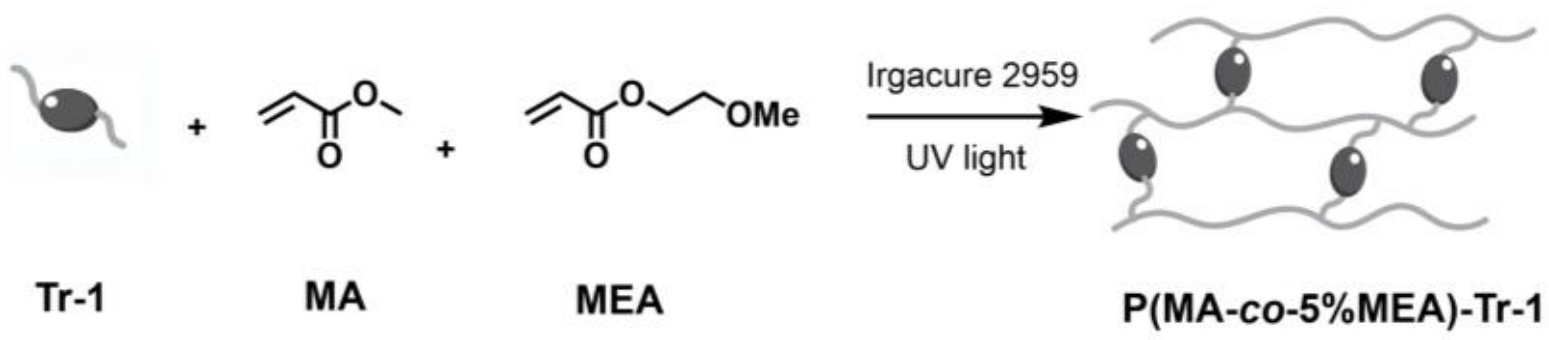

Irgacure 2959 (1 mg; 0.00446 mmol), **Tr-1** (68 mg; 0.118 mmol), methyl acrylate (**MA**; 1.04 mL; 11.04 mmol; density = 0.956 g/mL at 25°C), and 2-methoxyethyl acrylate (**MEA**; 48 µL; 0.448 mmol; density = 1.012 g/mL at 25°C) were combined in a vial, and the mixture was purged with nitrogen for 5 min. Two glass microscope slides were covered with clear tape, and two pieces of folded parafilm were placed between them as spacers. The slides were clamped together using office clamps, and the monomer solution was introduced into the gap with a pipette. Polymerization was carried out in a 365 nm LED light box (Dr. Hönle LED Cube). Samples were exposed at 50% output power for a time of 2 min (12 bursts of 10 s each), with 20–30 s pauses between bursts; this was followed by irradiation at 20% output power for a time of 5 min (15 bursts of 20 s each), with 20–30 s pauses between bursts. The slides were carefully separated, and the resulting polymer film was dried in a vacuum oven at 60 °C overnight.

2.7.5 Synthesis of **P(MA-*co*-10%MEA)-Tr-1**

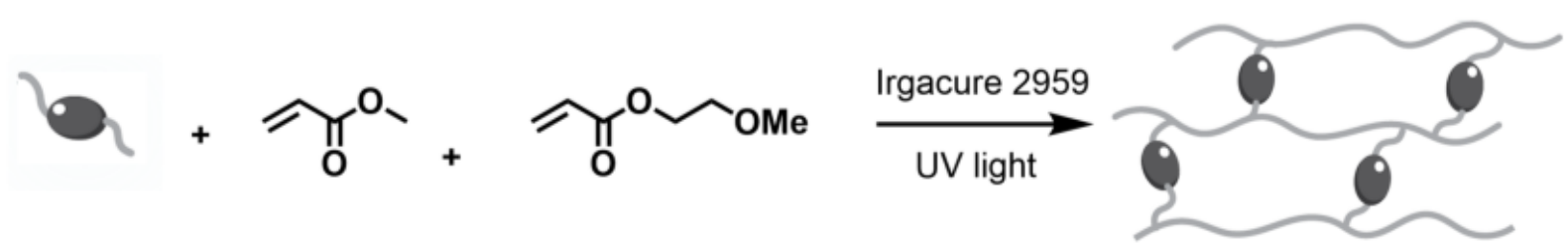

Tr-1 MA MEA P(MA-*co*-10%MEA)-Tr-1

Irgacure 2959 (1 mg; 0.00446 mmol), **Tr-1** (68 mg; 0.118 mmol), methyl acrylate (**MA**; 1 mL; 10.09 mmol; density = 0.956 g/mL at 25°C), and 2-methoxyethyl acrylate (**MEA**; 103 µL; 0.801 mmol; density = 1.012 g/mL at 25°C) were combined in a vial, and the mixture was purged with nitrogen for 5 min. Two glass microscope slides were covered with clear tape, and two pieces of folded parafilm were placed between them as spacers. The slides were clamped together using office clamps, and the monomer solution was introduced into the gap with a pipette. Polymerization was carried out in a 365 nm LED light box (Dr. Hönle LED Cube). Samples were exposed at 50% output power for a time of 2 min (12 bursts of 10 s each), with 20–30 s pauses between bursts; this was followed by irradiation at 20% output power for a time of 5 min (15 bursts of 20 s each), with 20–30 s pauses between bursts. The slides were carefully separated, and the resulting polymer film was dried in a vacuum oven at 60 °C overnight.

2.7.6 Synthesis of **PMA-Tr-2**

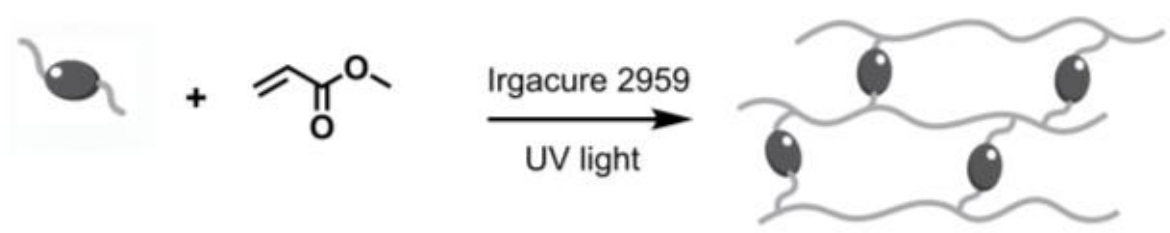

Tr-2 MA PMA-Tr-2

Irgacure 2959 (1 mg; 0.00446 mmol), **Tr-2** (75 mg; 0.118 mmol), and methyl acrylate (**MA**; 1.07 mL; 11.8 mmol; density = 0.956 g/mL at 25°C) were combined in a vial, and the mixture was purged with nitrogen for 5 min. Two glass microscope slides were covered with clear tape, and two pieces of folded parafilm were placed between them to act as 200 µm spacers. The slides were clamped together using office clamps, and the monomer solution was introduced into the gap with a pipette. Polymerization was carried out in a 365 nm LED light box (Dr. Hönle LED Cube). Samples were exposed at 50% output power for a time of 2 min (12 bursts of 10 s each), with 20–30 s pauses between bursts; this was followed by irradiation at 20% output power for a time of 5 min (15 bursts of 20 s each), with 20–30 s pauses between bursts. The slides were then carefully separated, and the resulting polymer film was dried in a vacuum oven at 60 °C overnight.

3. Nuclear magnetic resonance (NMR) data

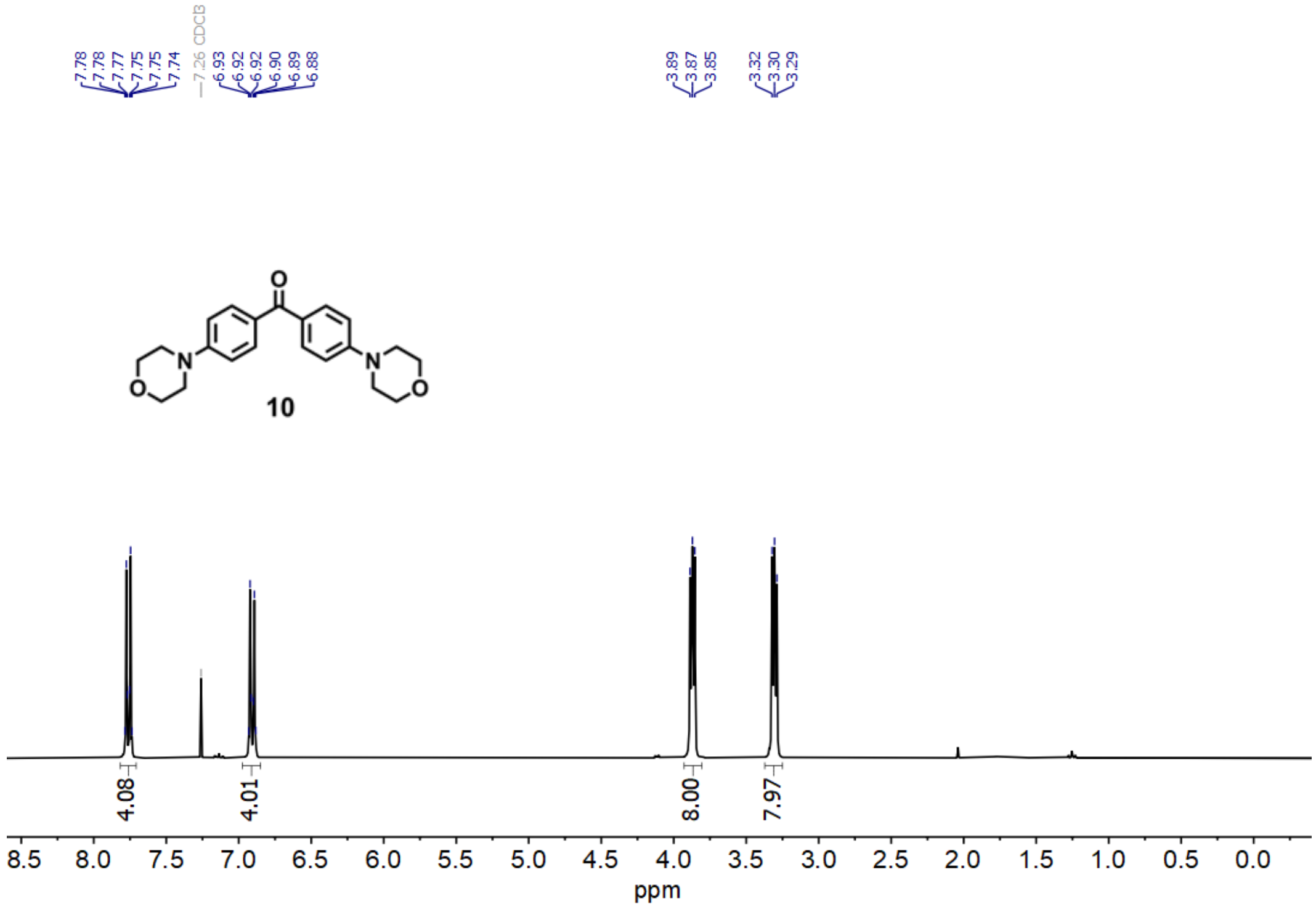

Figure S1. ^{1}H NMR spectrum of compound **10** (500 MHz, $CDCl_3$).

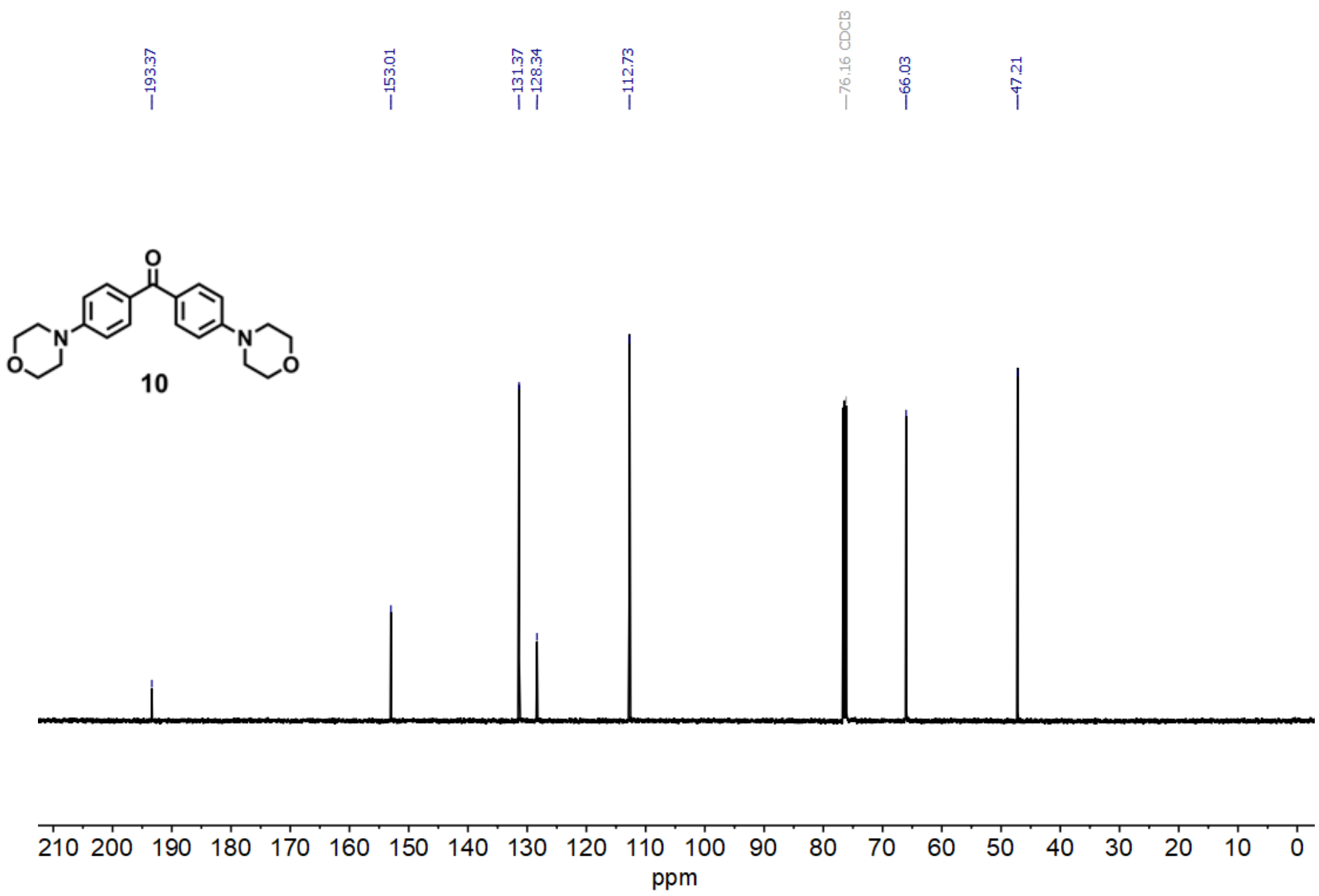

Figure S2.^{13}C NMR spectra of the compound **10** (125 MHz, $CDCl_3$).

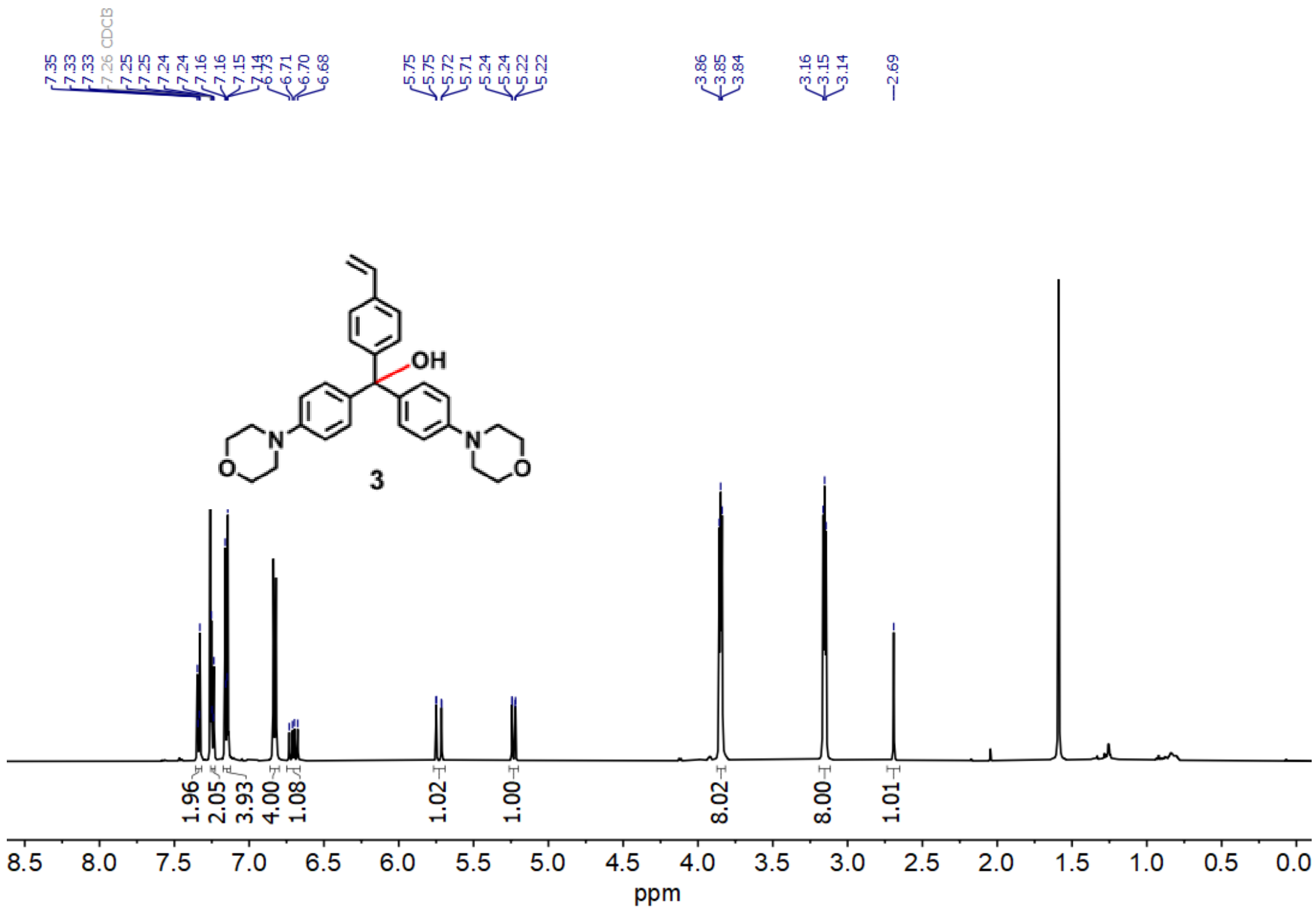

Figure S3. ^{1}H NMR spectrum of compound **3** (500 MHz, $CDCl_3$).

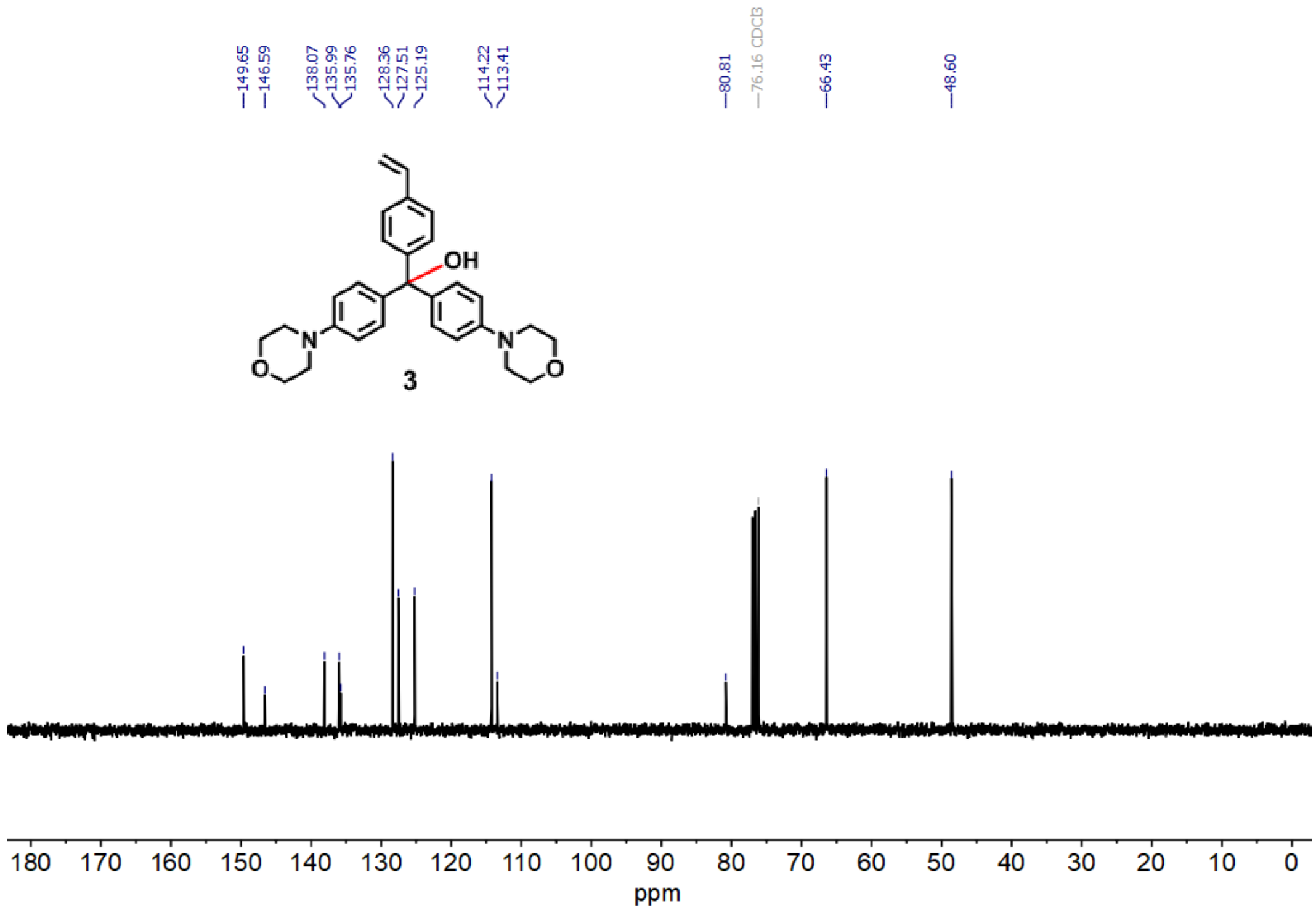

Figure S4. ^{13}C NMR spectrum of compound **3** (75 MHz, $CDCl_3$).

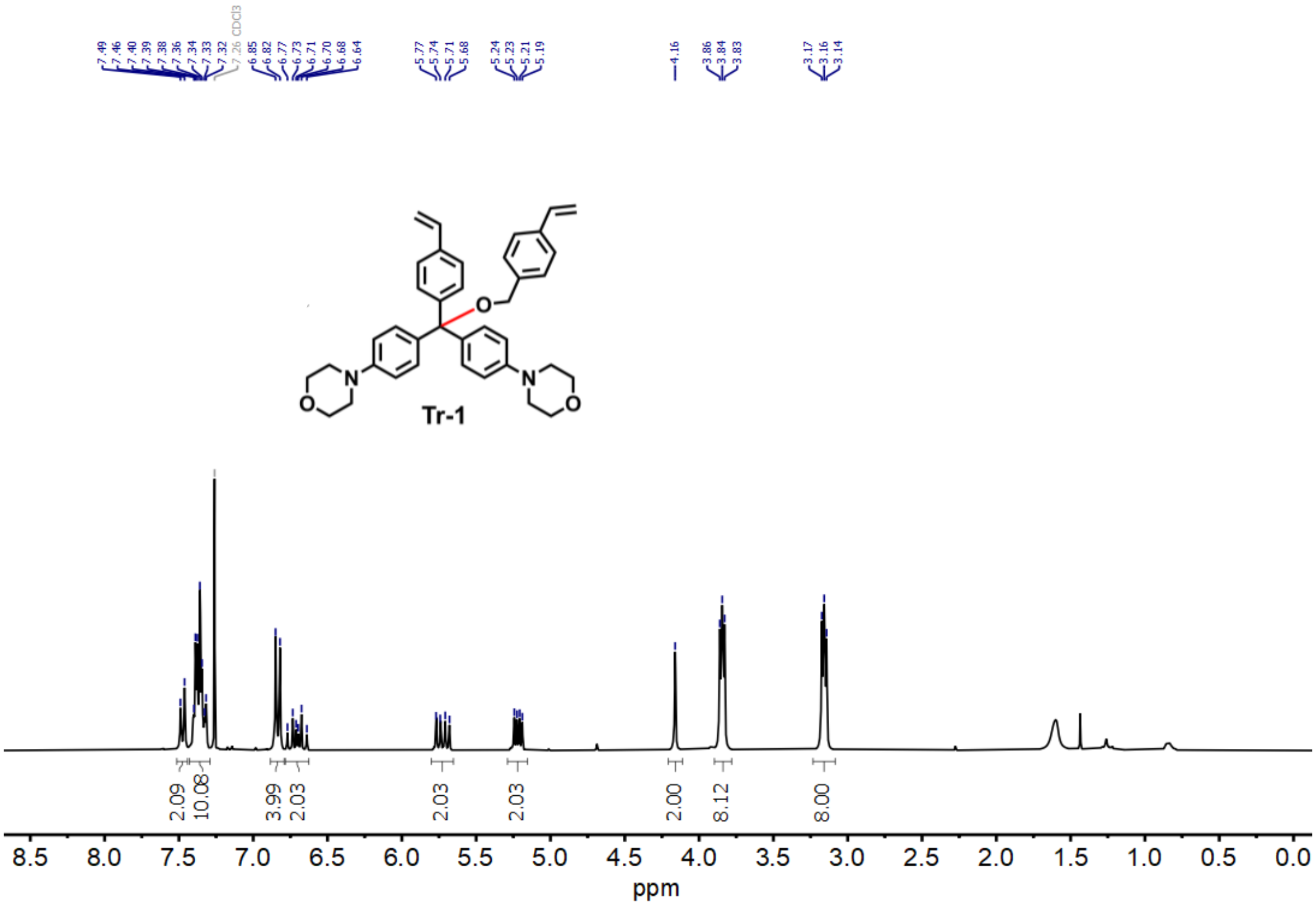

Figure S5. ^{1}H NMR spectrum of **Tr-1** (400 MHz, $CDCl_3$).

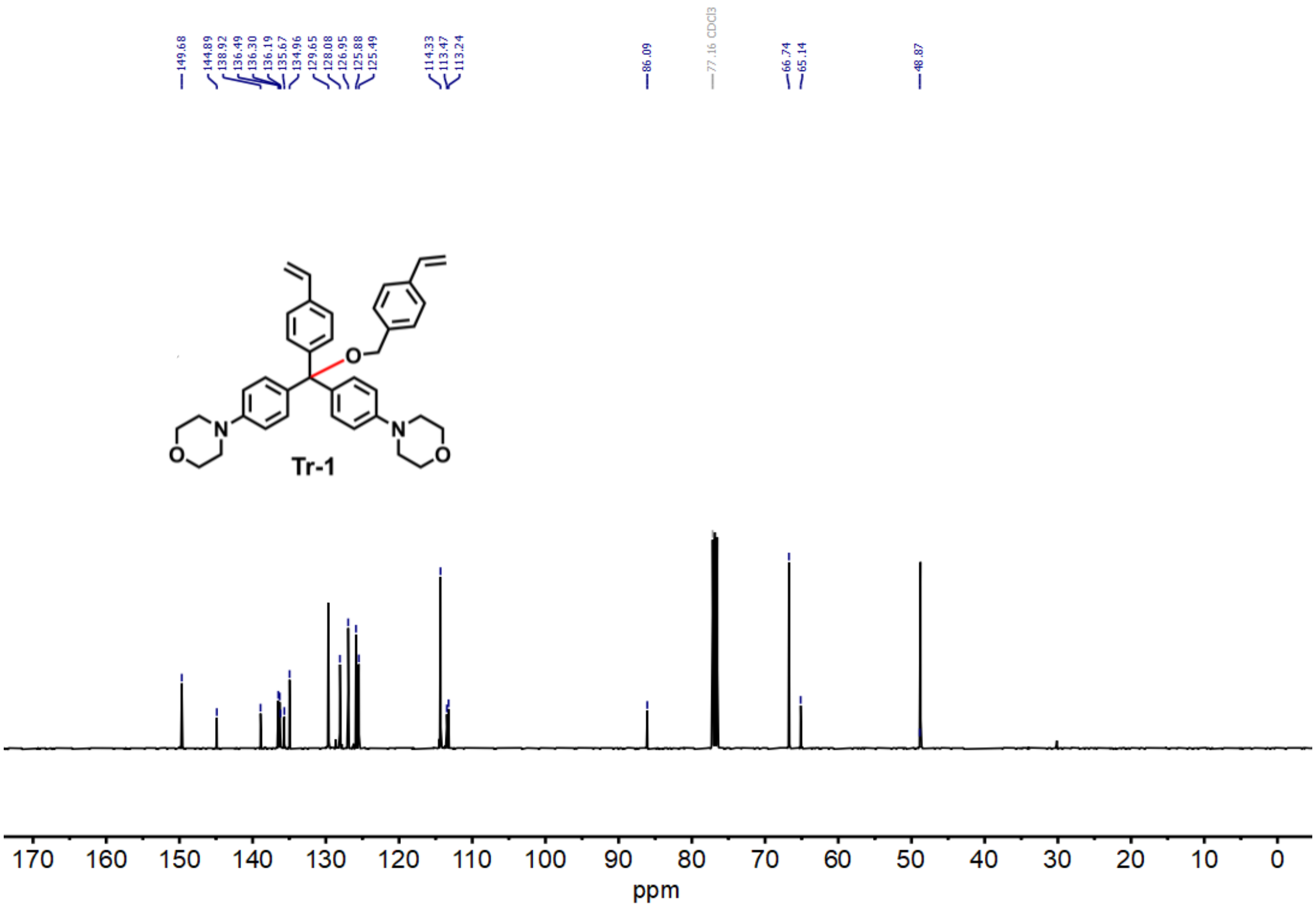

Figure S6. ^{13}C NMR spectrum of **Tr-1** (100 MHz, $CDCl_3$).

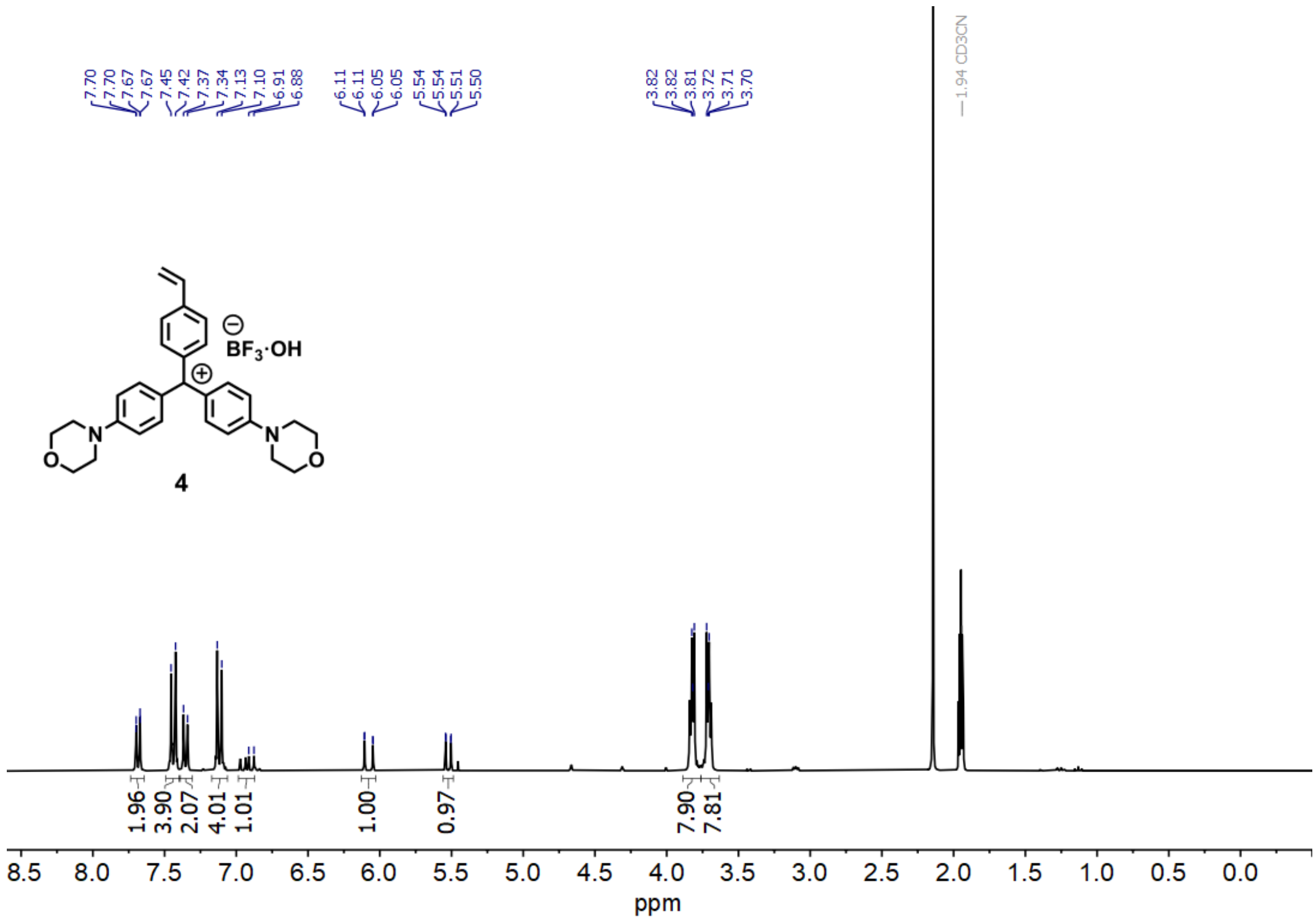

Figure S7. ^{1}H NMR spectrum of compound **4** (300 MHz, $CDCl_3$).

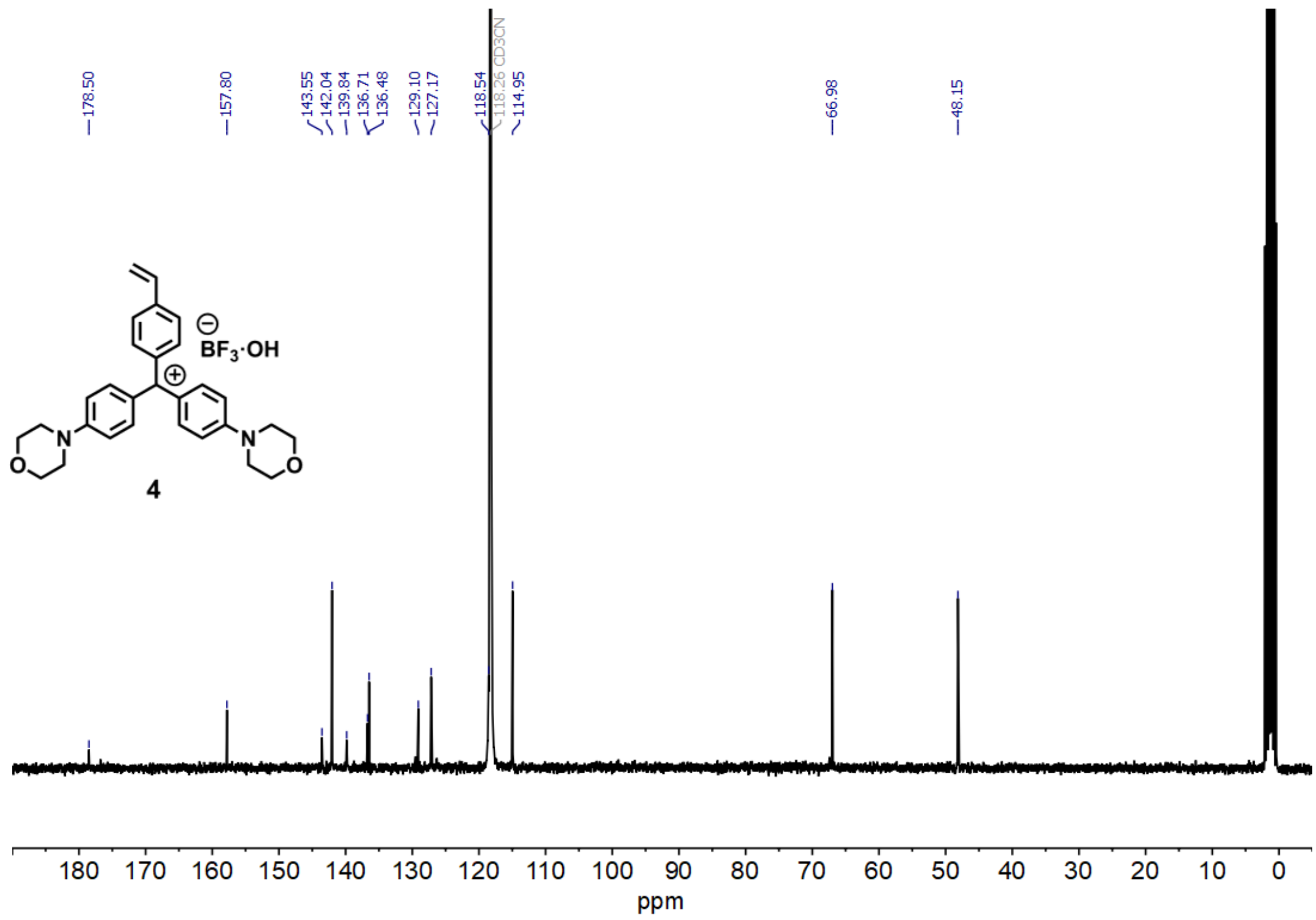

Figure S8. ^{13}C NMR spectrum of compound **4** (75 MHz, $CDCl_3$).

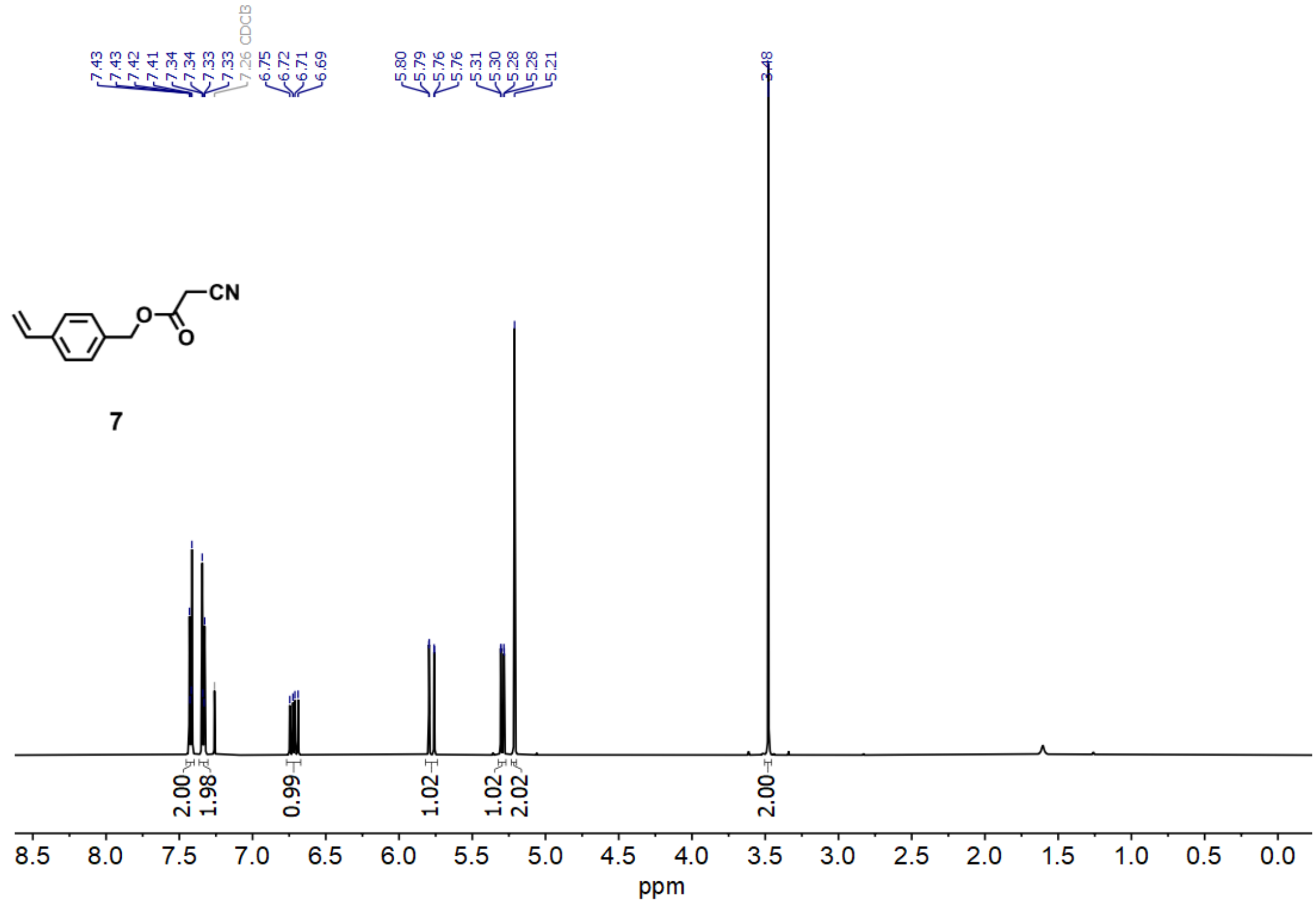

Figure S9. ^{1}H NMR spectrum of compound **7** (500 MHz, $CDCl_3$).

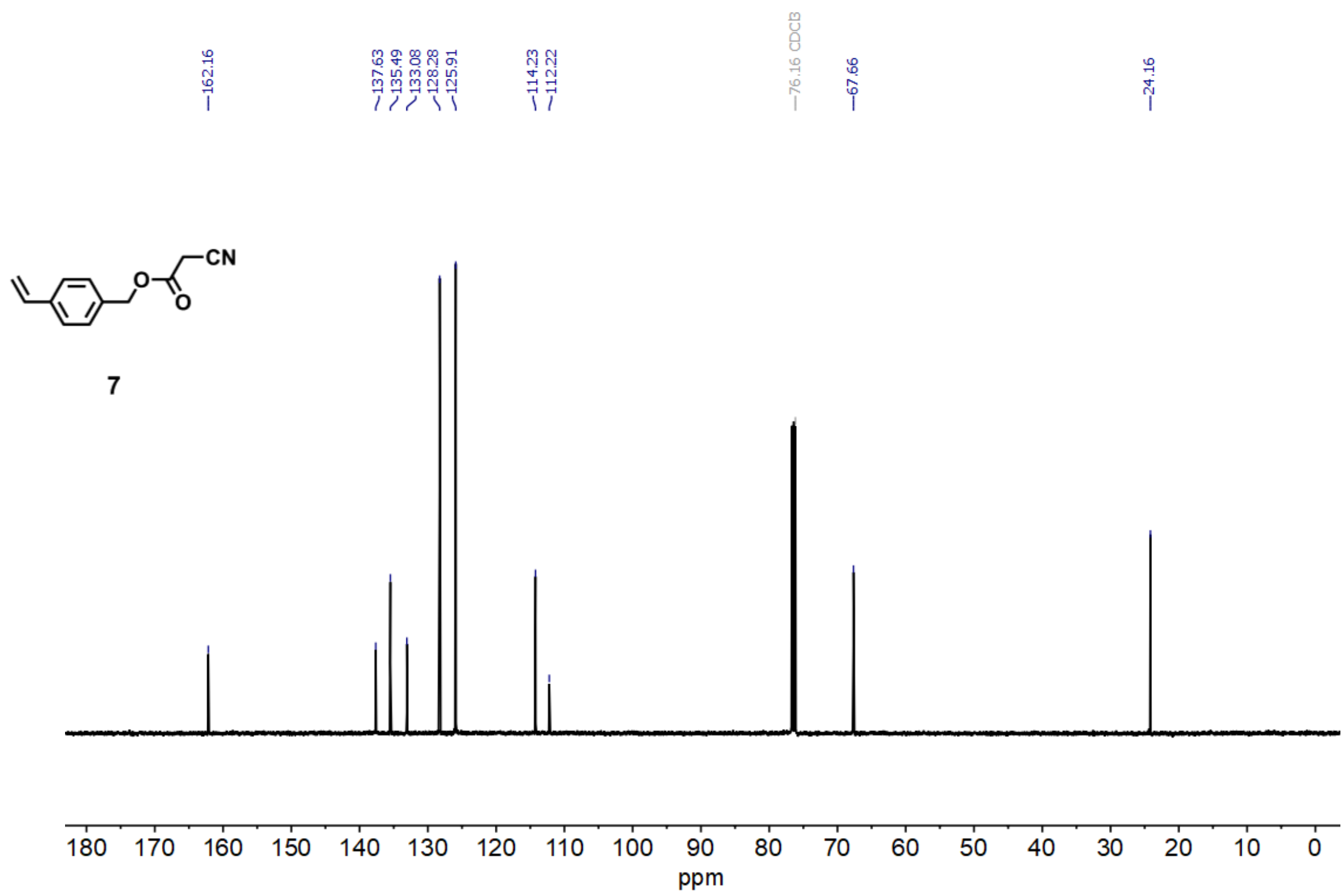

Figure S10. ^{13}C NMR spectrum of compound **9** (125 MHz, $CDCl_3$).

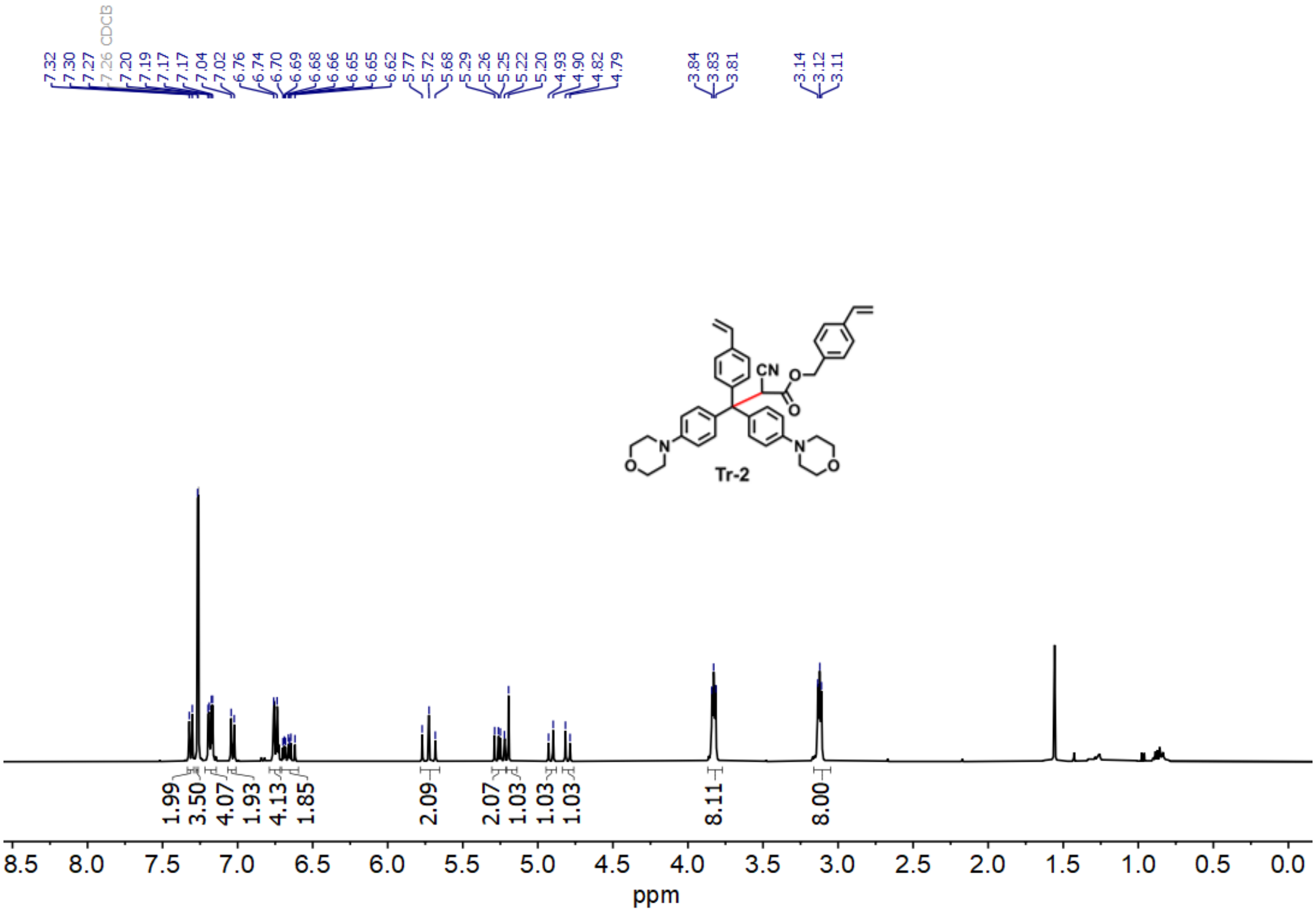

Figure S11. ^{1}H NMR spectrum of **Tr-2** (400 MHz, $CDCl_3$).

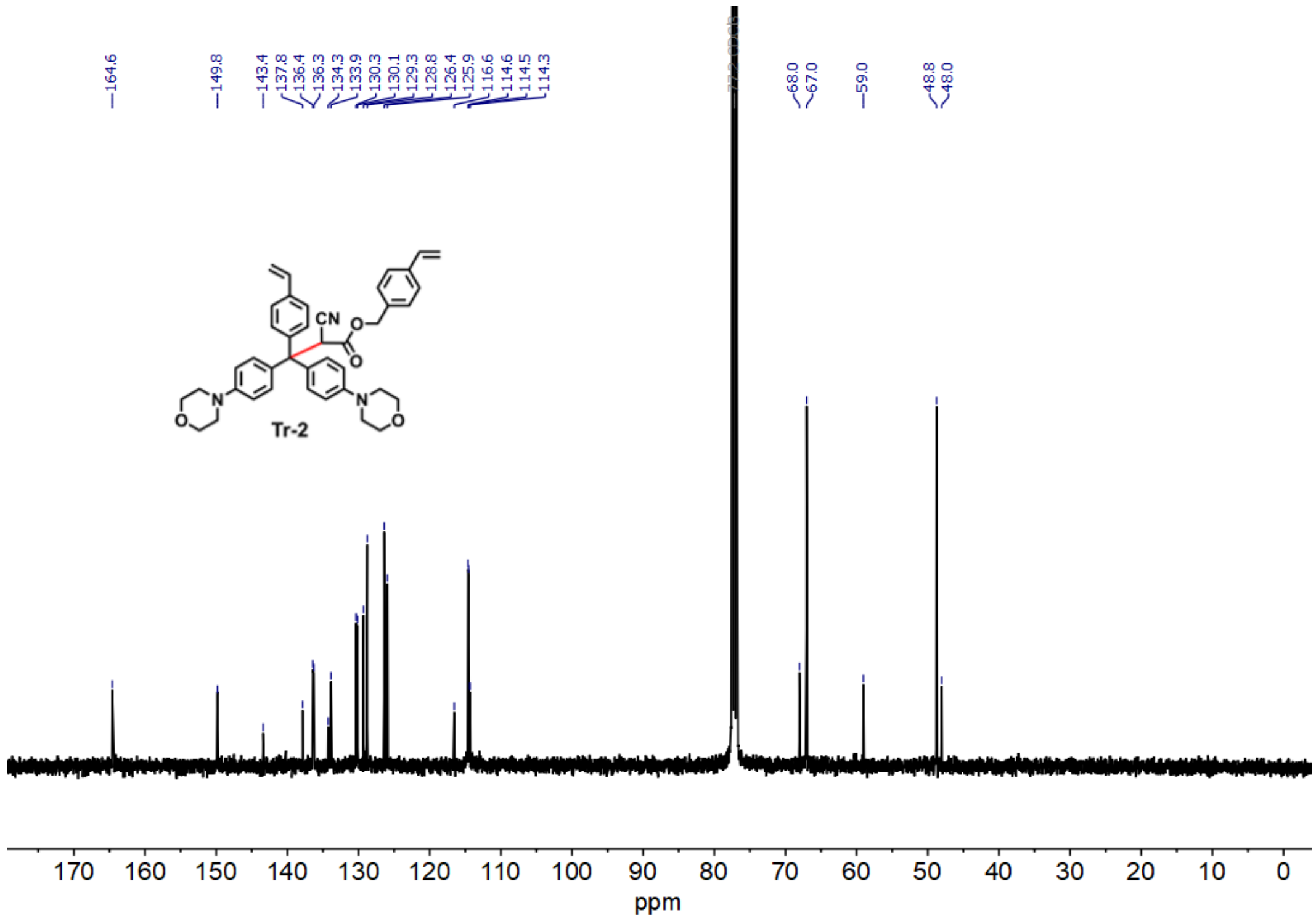

Figure S12 ^{13}C NMR spectrum of **Tr-2** (100 MHz, $CDCl_3$).

4. Fourier Transform Infrared (FT-IR) Spectroscopy

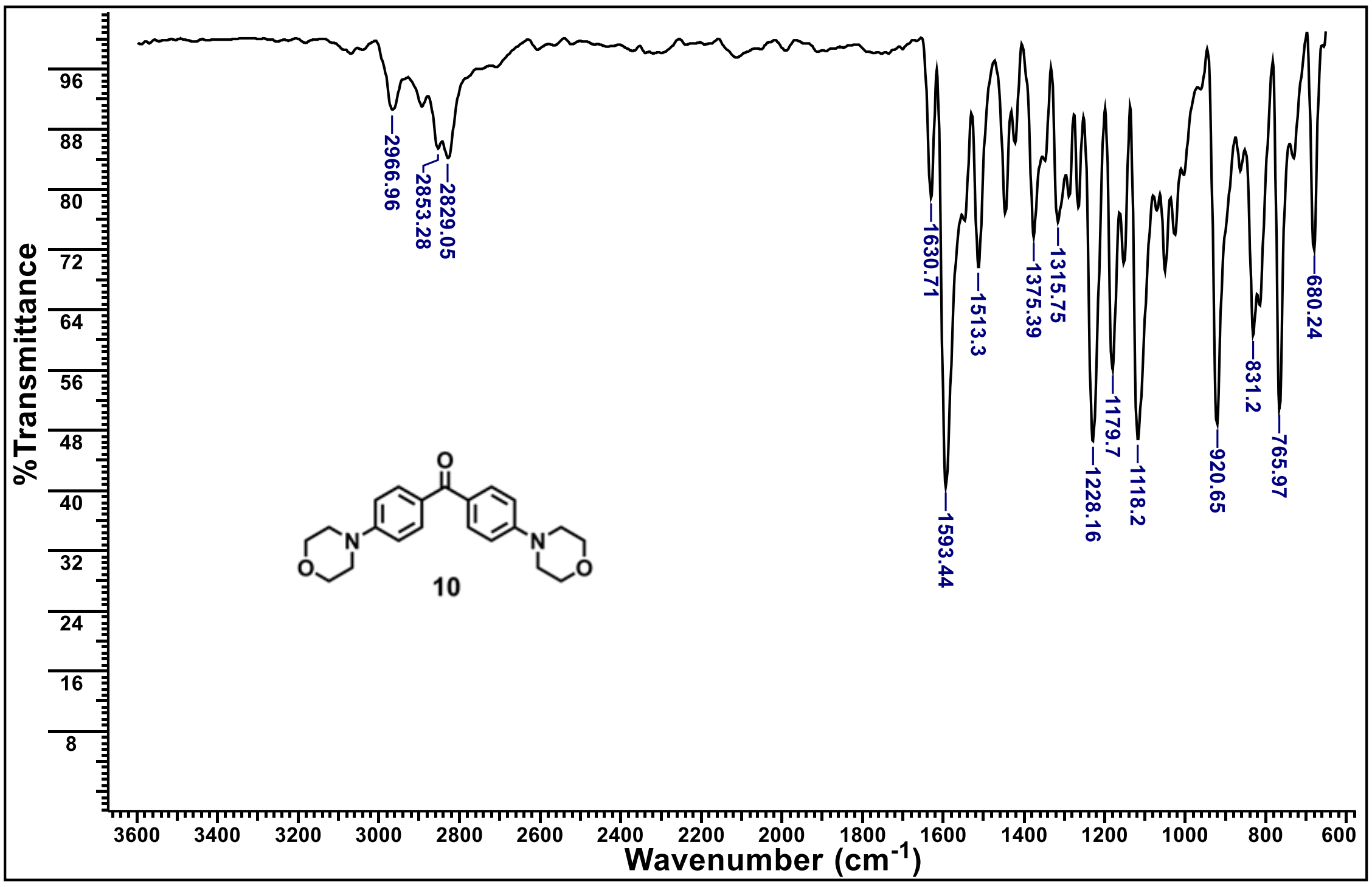

Figure S13. FT-IR spectrum of compound **10**

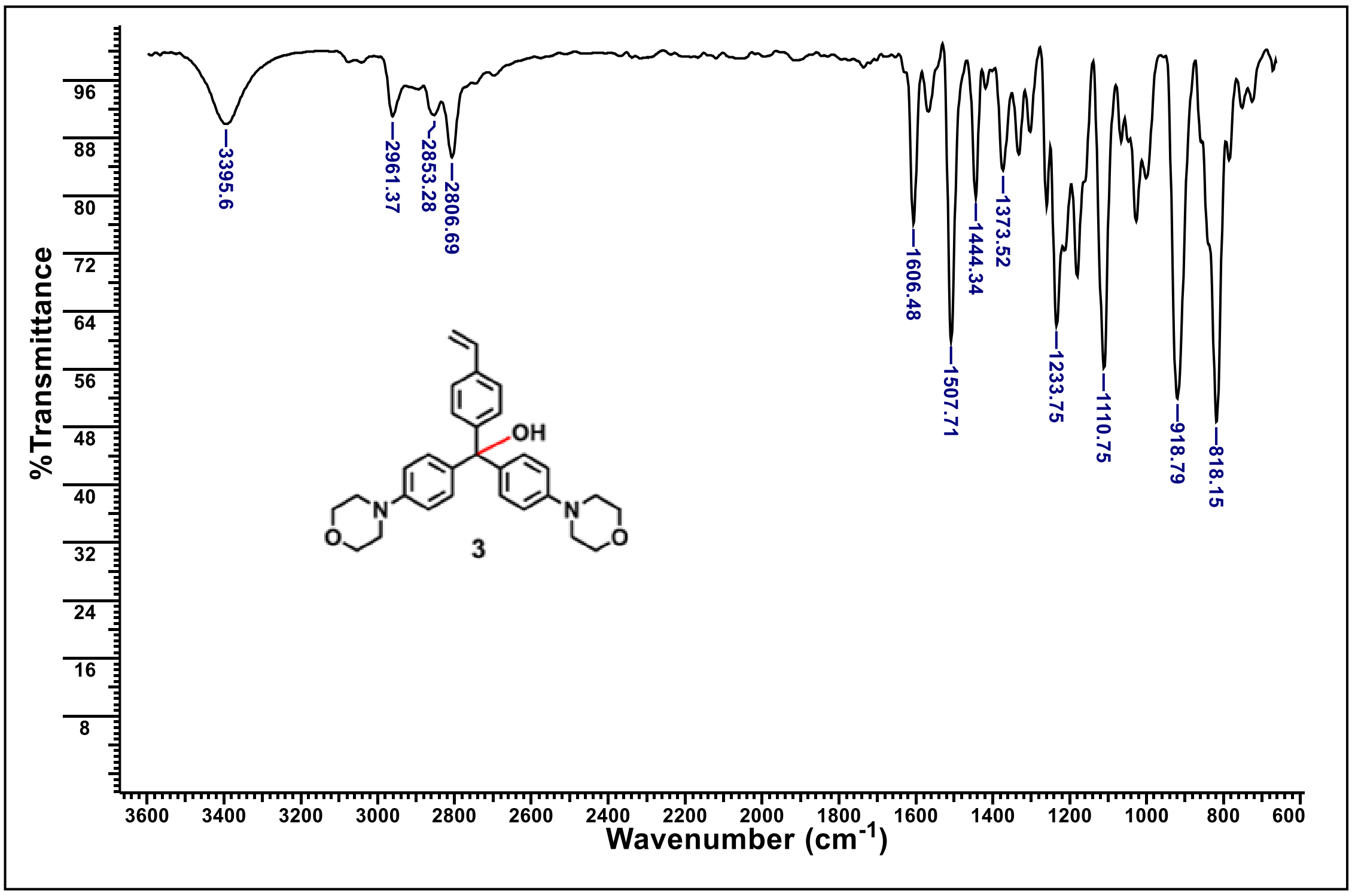

Figure S14. FT-IR spectrum of compound **3**

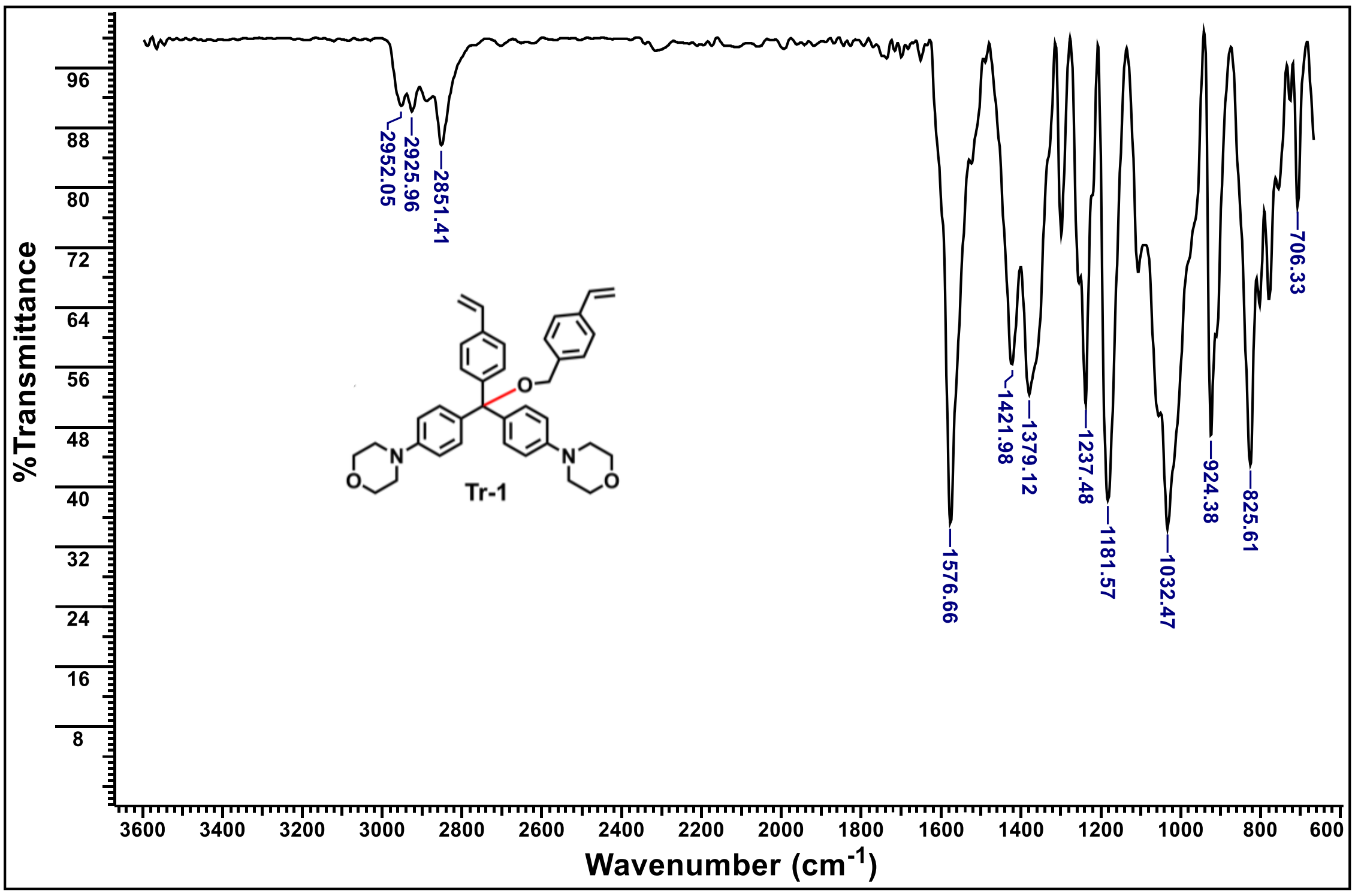

Figure S15. FT-IR spectrum of compound **Tr-1**

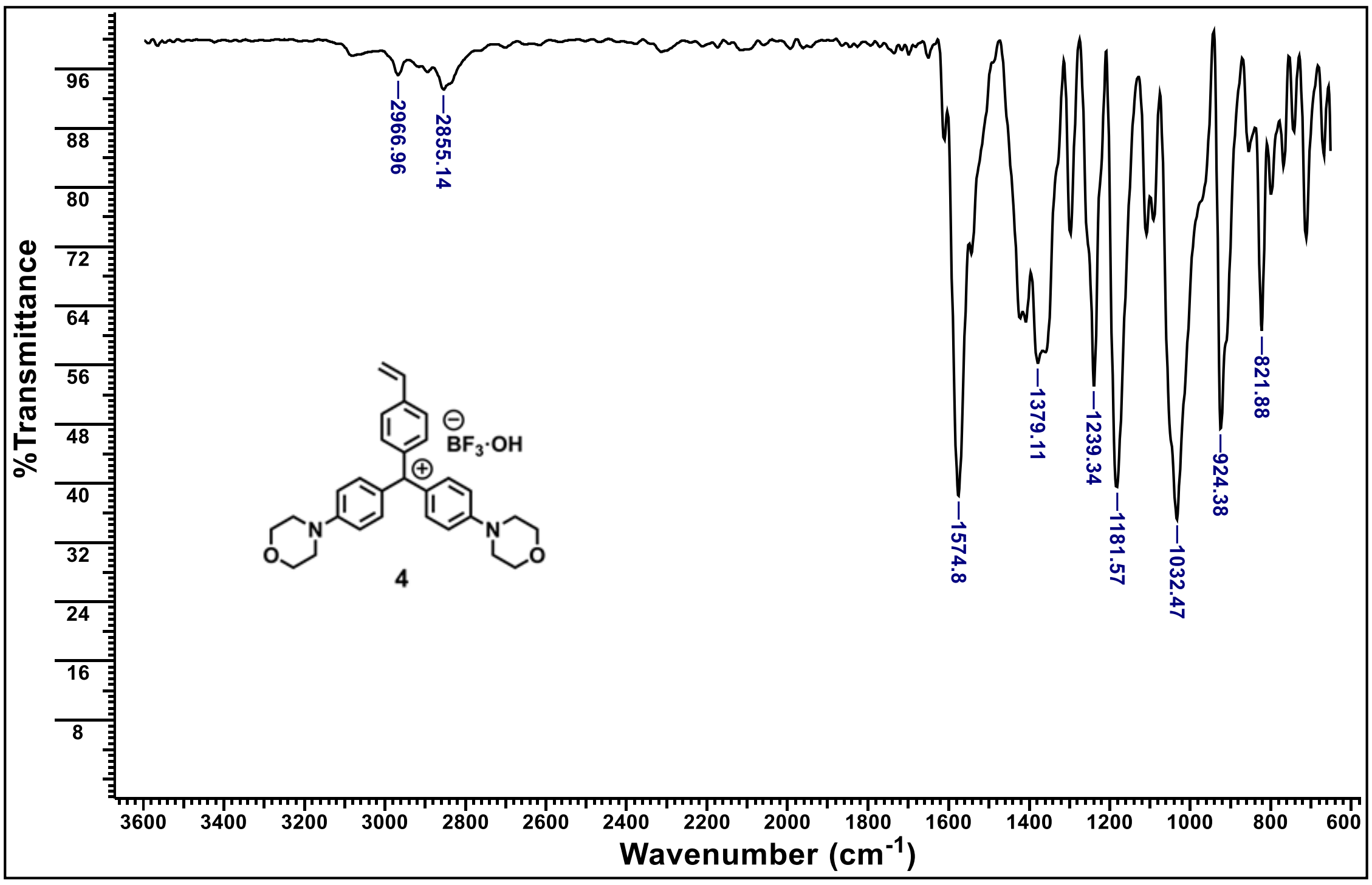

Figure S16. FT-IR spectrum of compound **4**

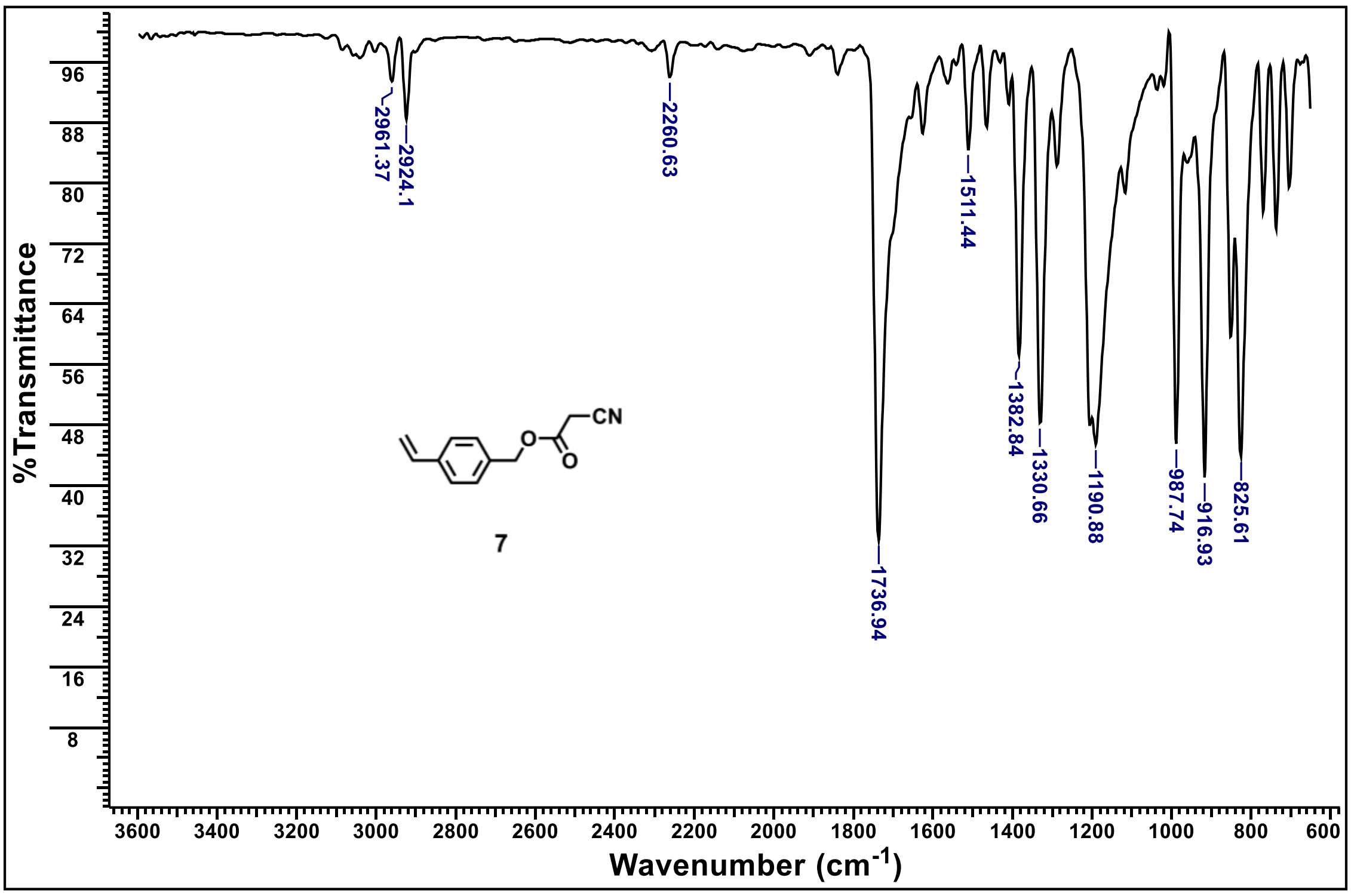

Figure S17. FT-IR spectrum of compound **7**

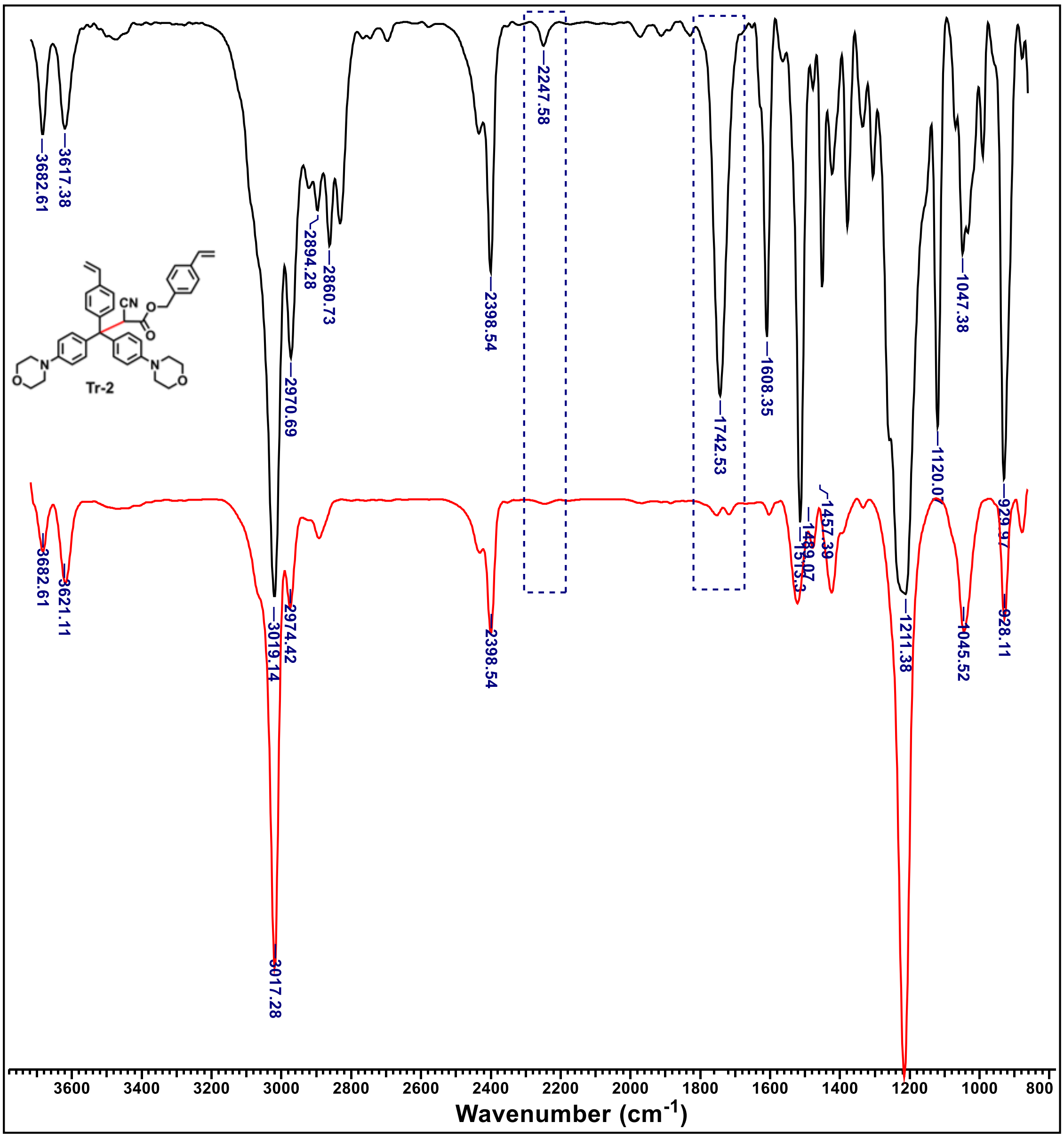

Figure S18. The FT-IR spectrum of **Tr-2** was recorded in solution (DCM), as the CN stretching band was very weak in the FT-IR spectrum of solid **Tr-2**. The red trace corresponds to the FT-IR spectrum of DCM, while the black trace corresponds to the spectrum of **Tr-2** in DCM. Overlaying the spectra clearly reveals the CN stretching band at 2247 cm^{-1}, confirming the presence of the CN bond.

5. High-Resolution Mass Spectrometry (HR-MS) Characterization

All newly synthesized compounds were characterized by high-resolution mass spectroscopy to support the structural analysis.

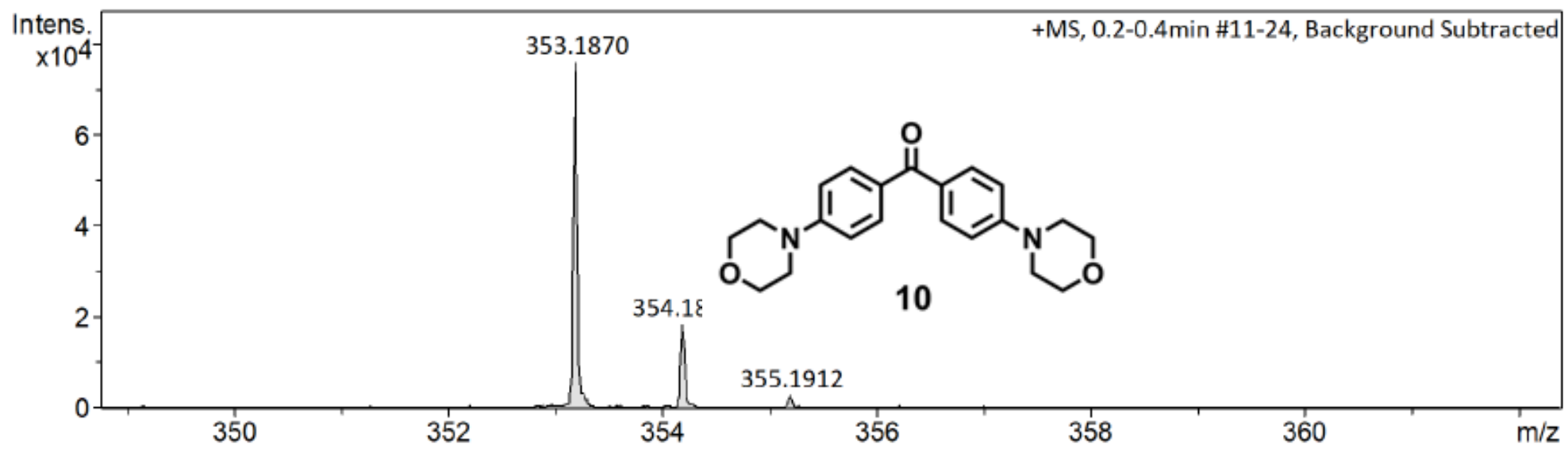

Meas. m/z	Ion Formula	m/z	|err| [ppm]	err [mDa]	mSigma	e¯ Conf	z
353.1870	C21H25N2O3	353.1860	2.9	-1.0	1.5	even	1+

Figure S19. HR-MS spectrum of **10**

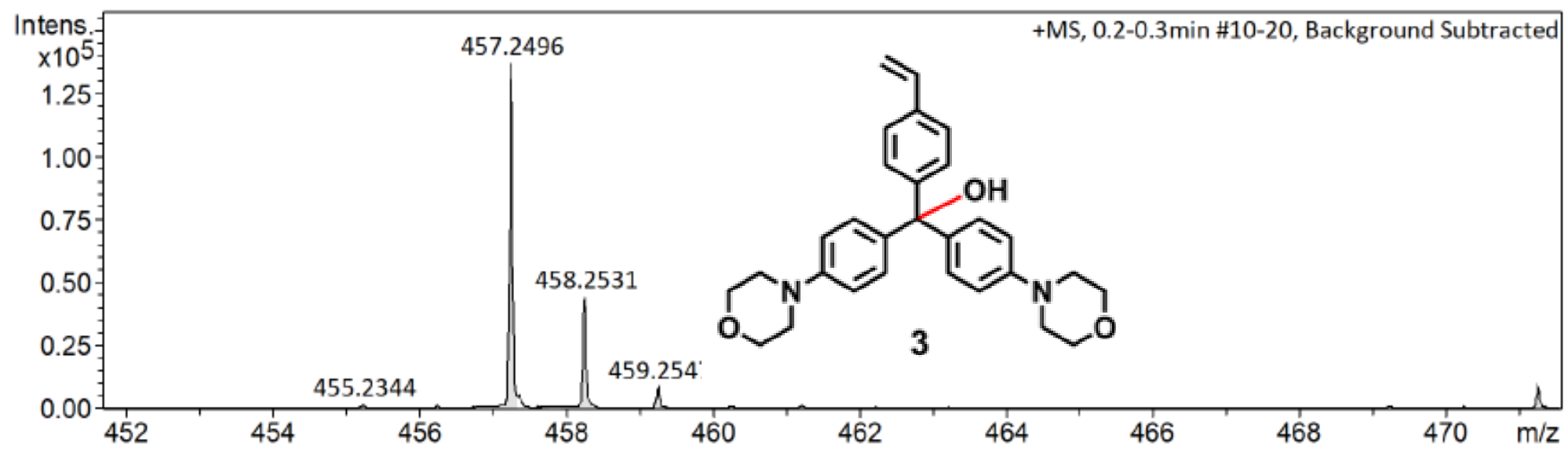

Meas. m/z	Ion Formula	m/z	|err| [ppm]	err [mDa]	mSigma	e¯ Conf	z
457.2496	C29H33N2O3	457.2486	2.4	1.1	2.1	even	1+

Figure S20. HR-MS spectrum of **3**

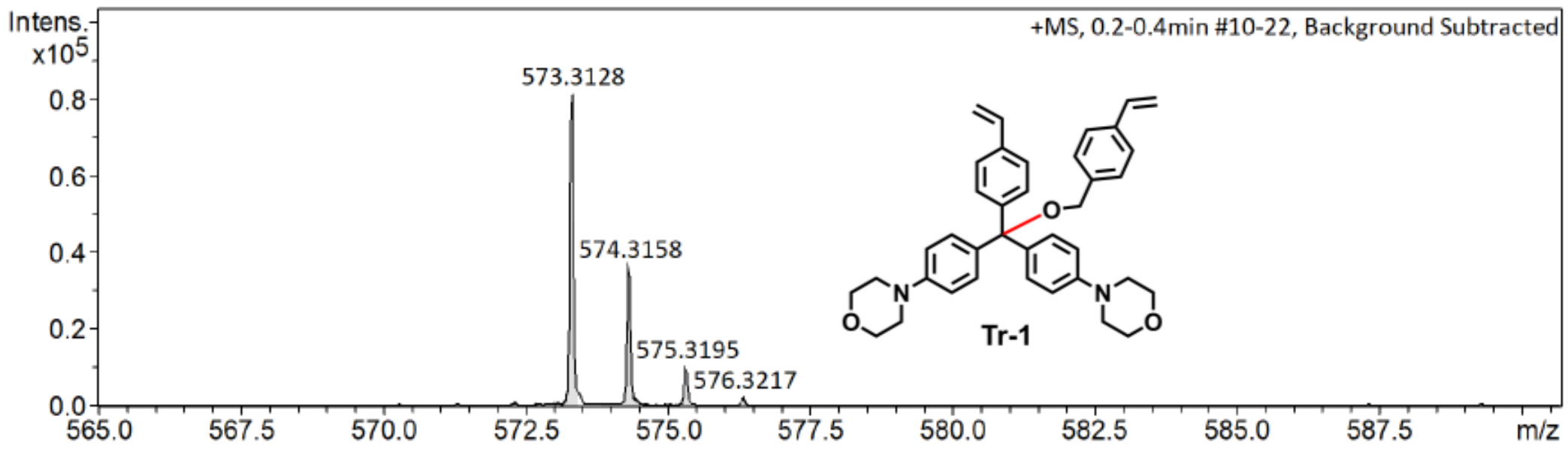

Meas. m/z	Ion Formula	m/z	|err| [ppm]	err [mDa]	mSigma	e⁻ Conf	z
573.3128	C38H41N2O3	573.3112	2.9	1.7	20.8	even	1+

Figure S21. HR-MS spectrum of **Tr-1**

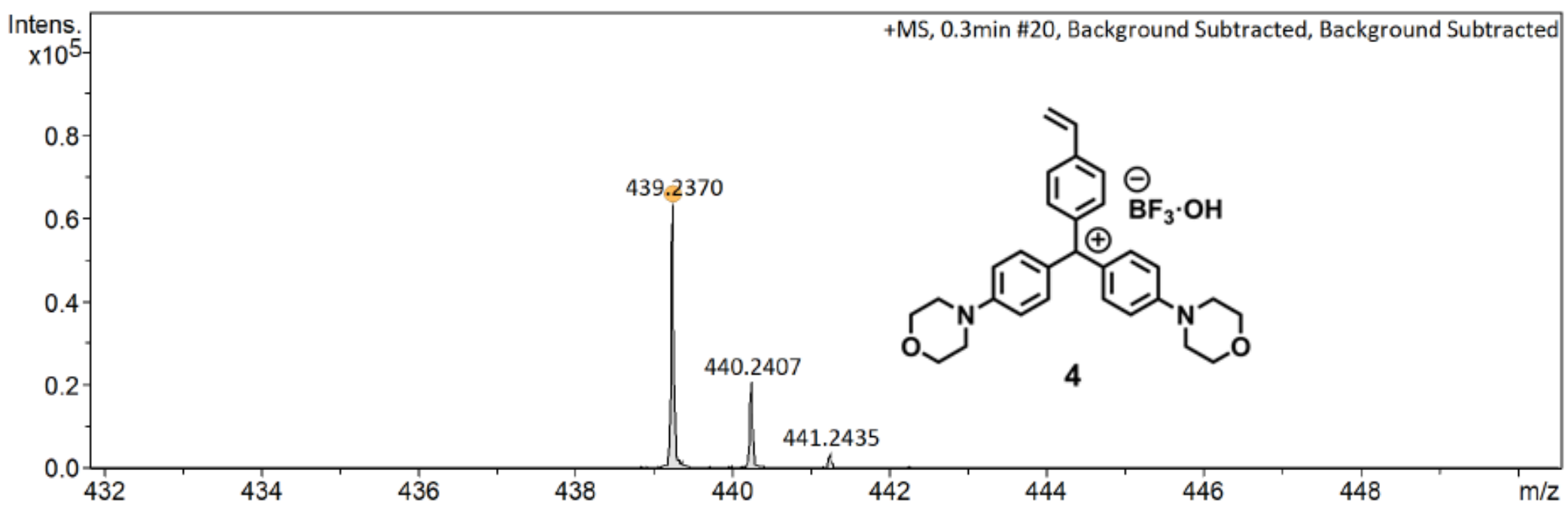

Meas. m/z	#	Ion Formula	m/z	|err| [ppm]	mSigma	Score	rdb	e⁻ Conf	z
439.2370	1	C29H31N2O2	439.2380	2.3	0.7	100.00	15.5	even	1+

Figure S22. HR-MS spectrum of **4**

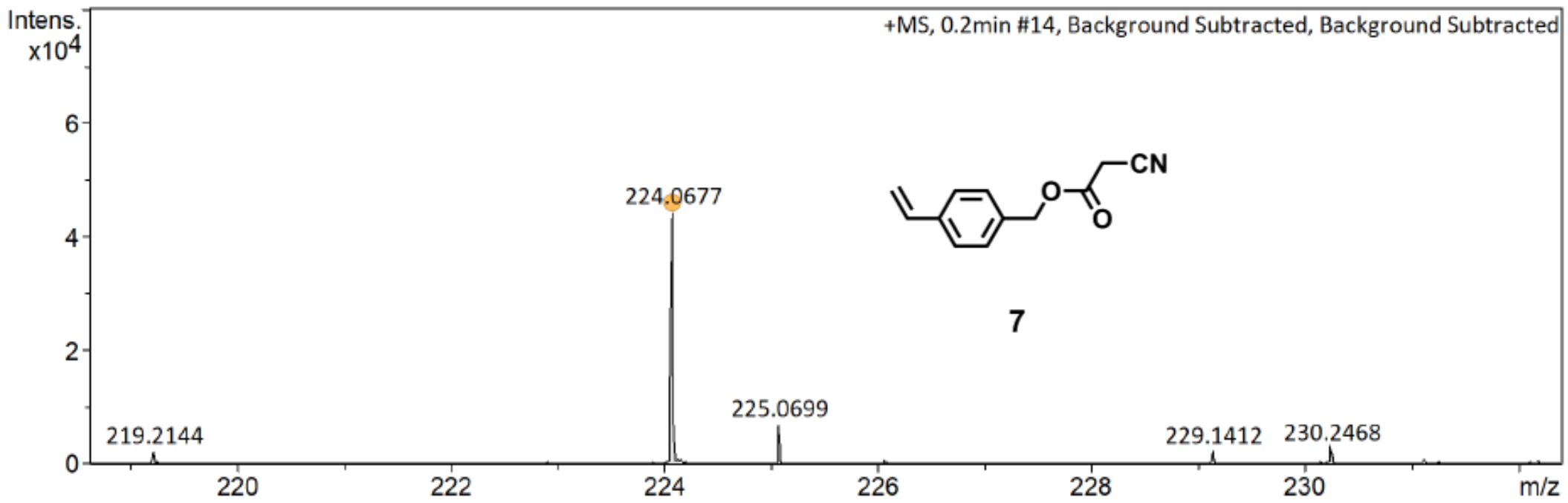

Meas. m/z	#	Ion Formula	m/z	|err| [ppm]	mSigma	Score	rdb	e⁻ Conf	z
224.0677	1	C12H11NNaO2	224.0682	2.2	12.0	100.00	7.5	even	1+

Figure S23. HR-MS spectrum of **7**

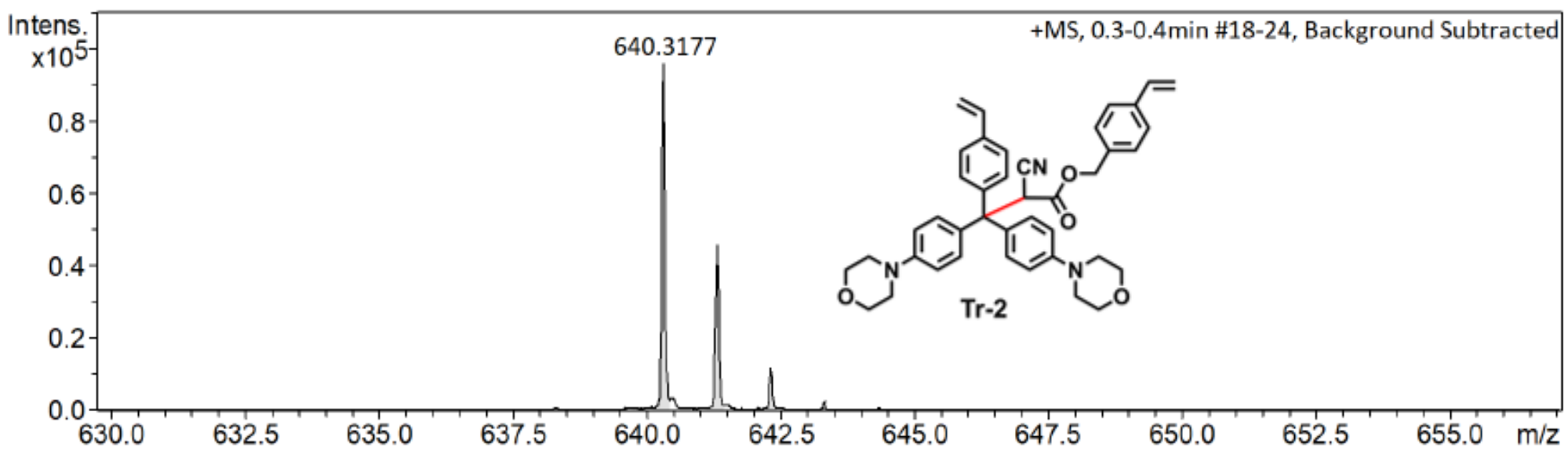

Meas. m/z	Ion Formula	m/z	|err| [ppm]	err [mDa]	mSigma	e⁻ Conf	z
640.3177	C41H42N3O4	640.3170	1.1	0.7	11.0	even	1+

Figure S24. HR-MS spectrum of **Tr-2**

6. UV–Visible Spectroscopy Analysis

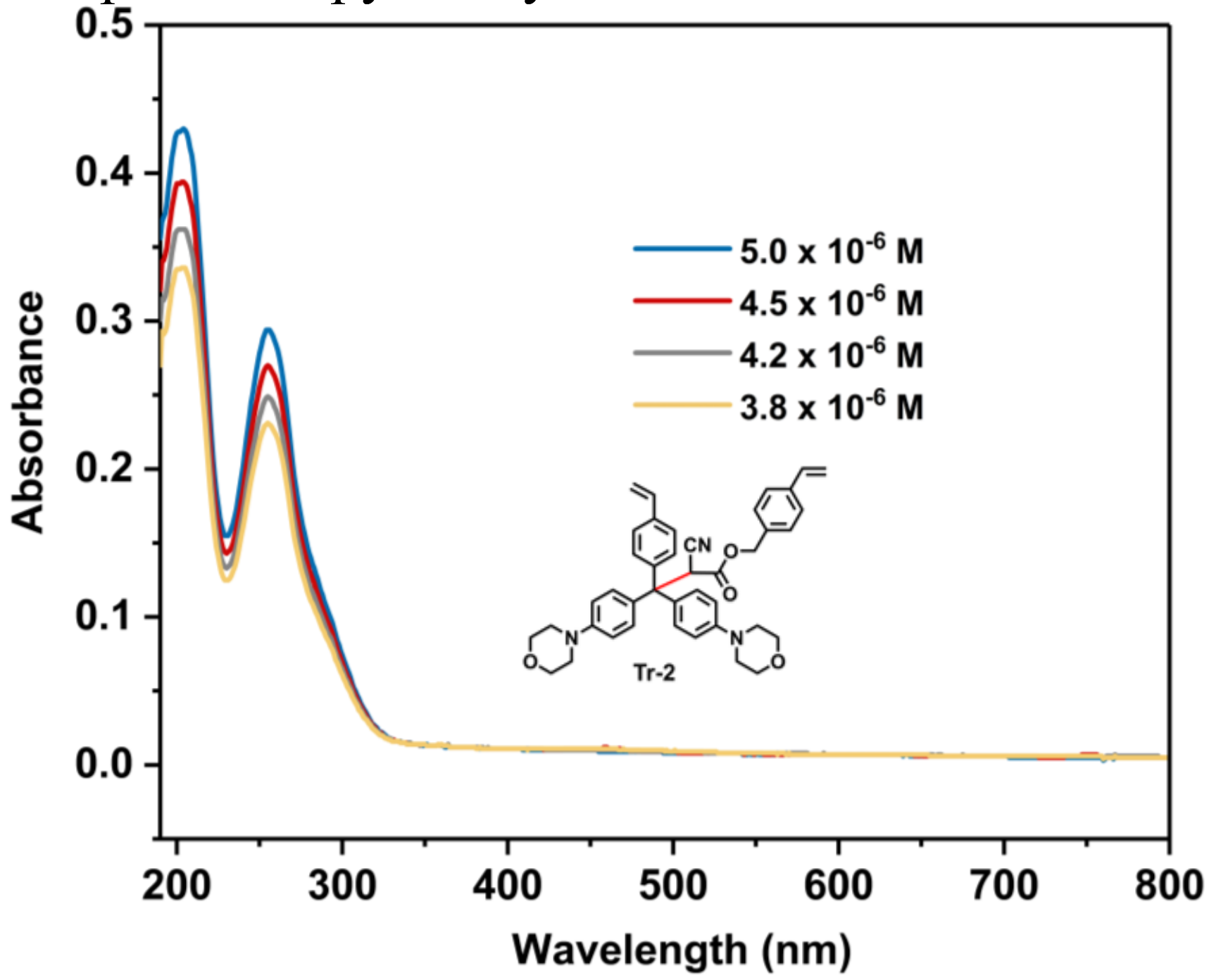

Figure S25. UV-vis spectrum of **Tr-2** recorded in acetonitrile (CH_3CN) at four different concentrations at 25 °C.

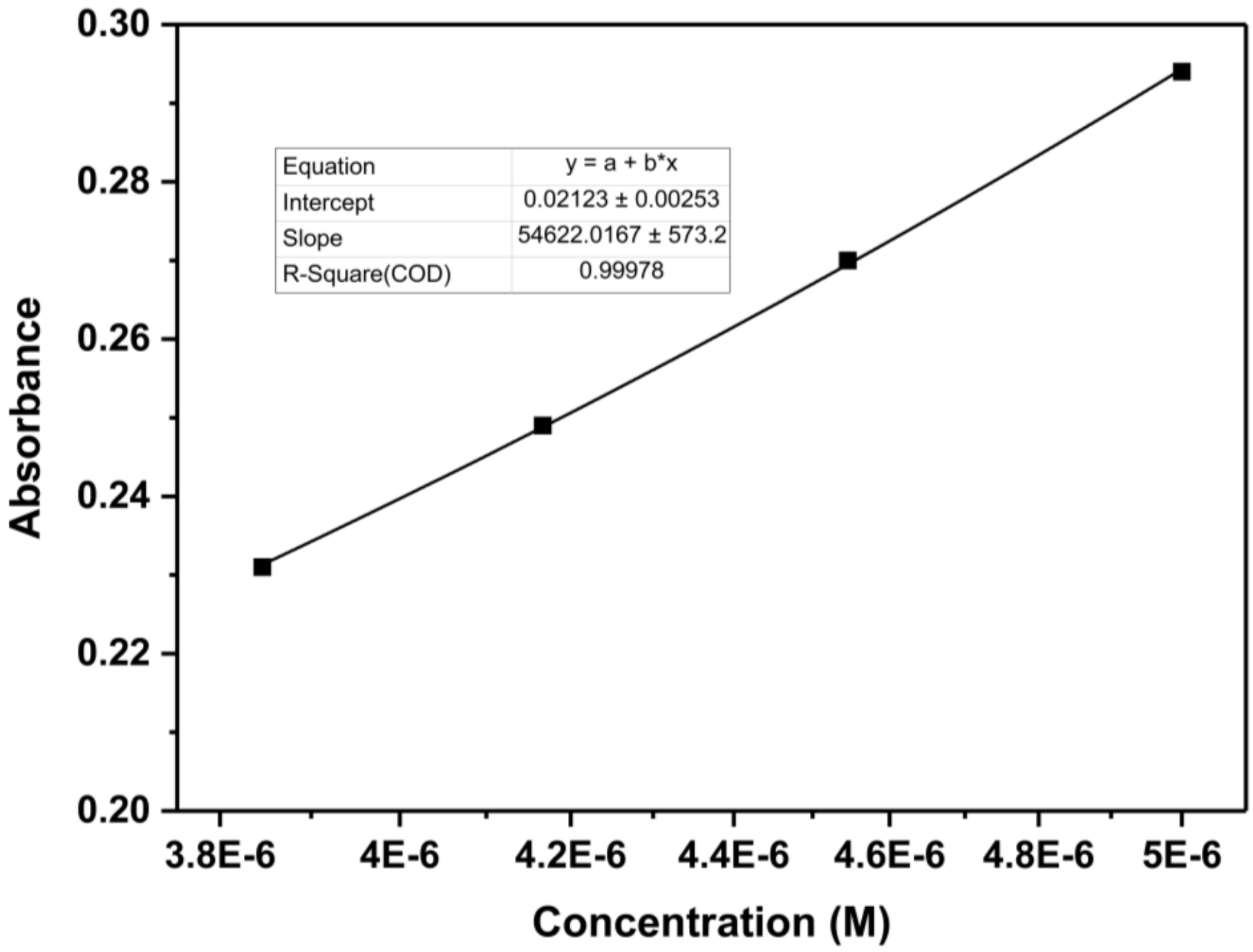

Figure S26 Absorbance versus concentration for **Tr-2** in acetonitrile (CH_3CN) at 25 °C. Linear regression was applied to determine the molar extinction coefficient (ε).

7. Thermal characterization of PMA and P(MA-*co*-HEA/MEA) polymer networks

We conducted the thermal characterization of the PMA and P(MA-co-HEA/MEA) networks using homogeneous polymer films prepared in glass molds with a controlled thickness of ca. 200 µm (synthetic procedures on page S8–S10). Thermal analysis was performed using thermogravimetric analysis (TGA) and differential scanning calorimetry (DSC). The following polymer samples were examined:

1. **PMA-Tr-1**
2. **P(MA-*co*-5%HEA)-Tr-1**
3. **P(MA-*co*-10%HEA)-Tr-1**
4. **P(MA-*co*-5%MEA)-Tr-1**
5. **P(MA-*co*-10%MEA)-Tr-1**
6. **PMA-Tr-2**

7.1 Thermogravimetric Analysis (TGA)

The TGA profile of **PMA-Tr-1** demonstrates excellent thermal stability, with virtually no mass loss observed up to approximately 350 °C. The sample exhibits a 5% weight-loss temperature ($T_{5\%}$) of 382 °C, indicating a high thermal-decomposition threshold (Figure S27). A rapid decrease in mass occurs between ~380–450 °C, followed by a small residual mass at higher temperatures.

All other samples show comparable thermal behavior, with negligible weight loss up to ~350 °C. The $T_{5\%}$ values are 391 °C for **P(MA-*co*-5%HEA)-Tr-1**, 387 °C for **P(MA-*co*-10%HEA)-Tr-1**, 380 °C for **P(MA-*co*-5%MEA)-Tr-1,** 382 °C for **P(MA-*co*-10%MEA)-Tr-1**, and 387 °C for **PMA-Tr-2**, which highlight the consistently high thermal stability across the entire series of tested polymers (Figures S27–S32).

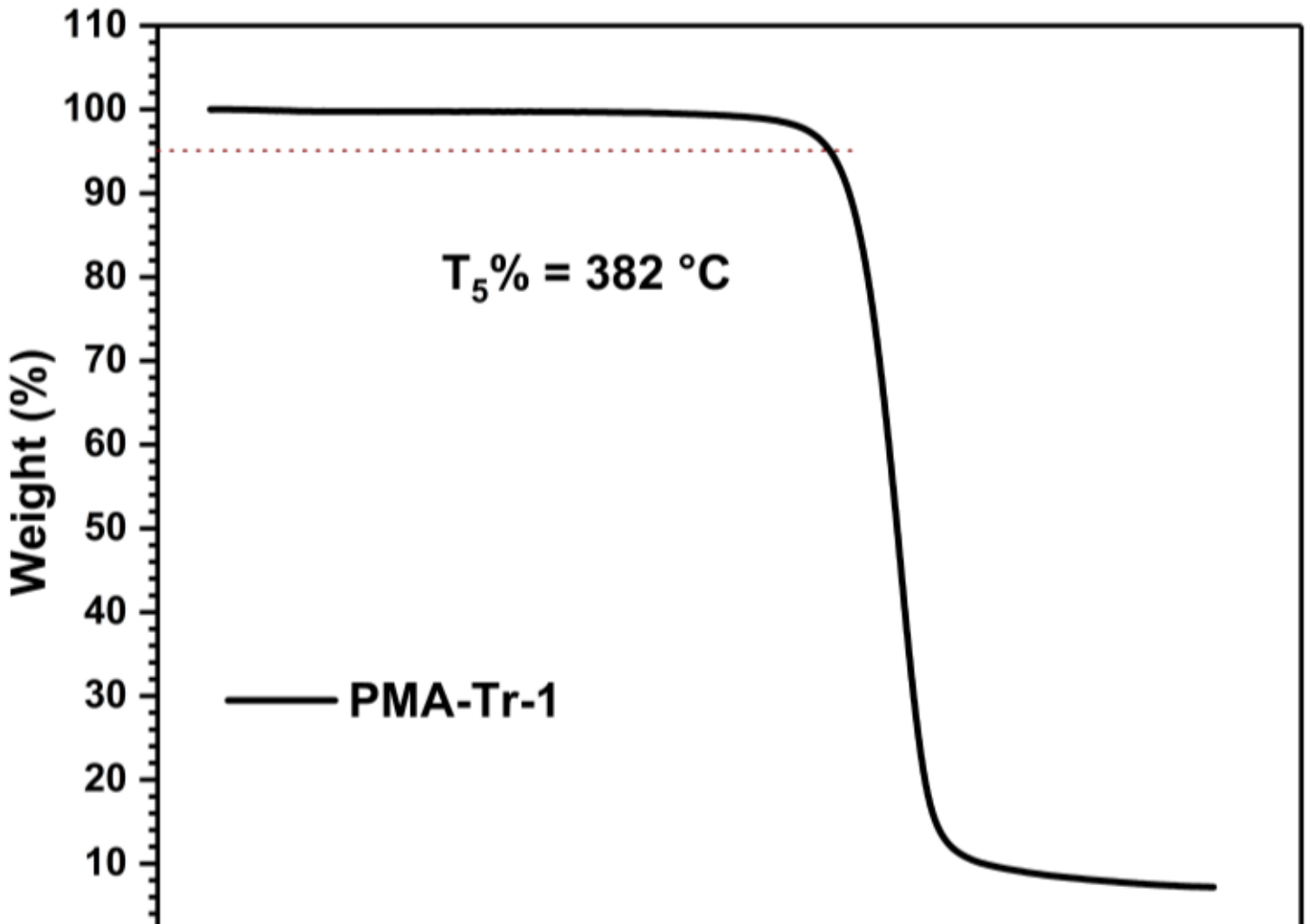

Figure S27. Thermogravimetric analysis curve of **PMA-Tr-1**. The red dashed line indicates the temperature at 5% weight loss ($T_{5\%}$), which occurs at 382 °C

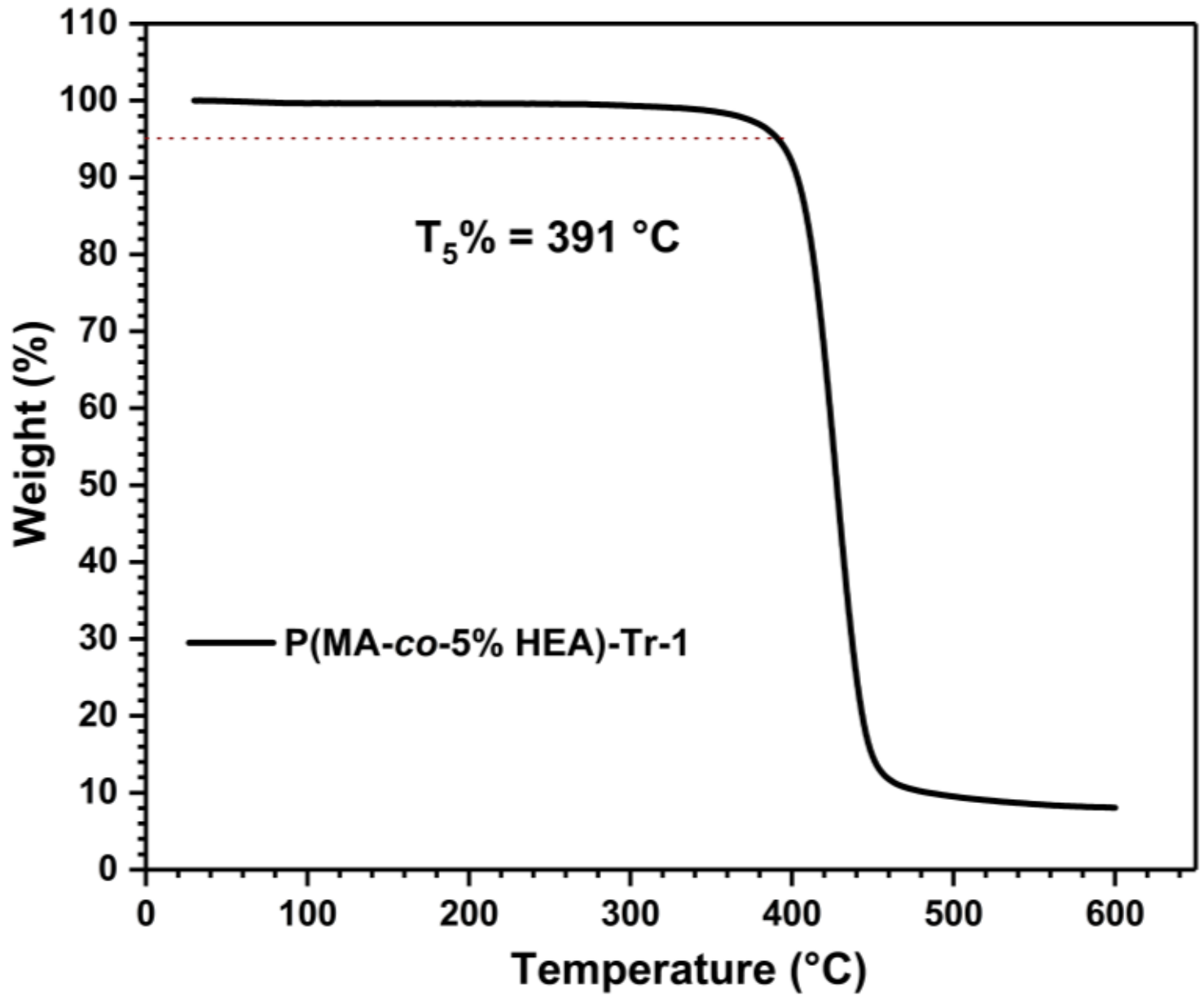

Figure S28. Thermogravimetric analysis curve of **P(MA-*co*-5%HEA)-Tr-1**. The red dashed line indicates the temperature at 5% weight loss (T_5%), which occurs at 391 °C

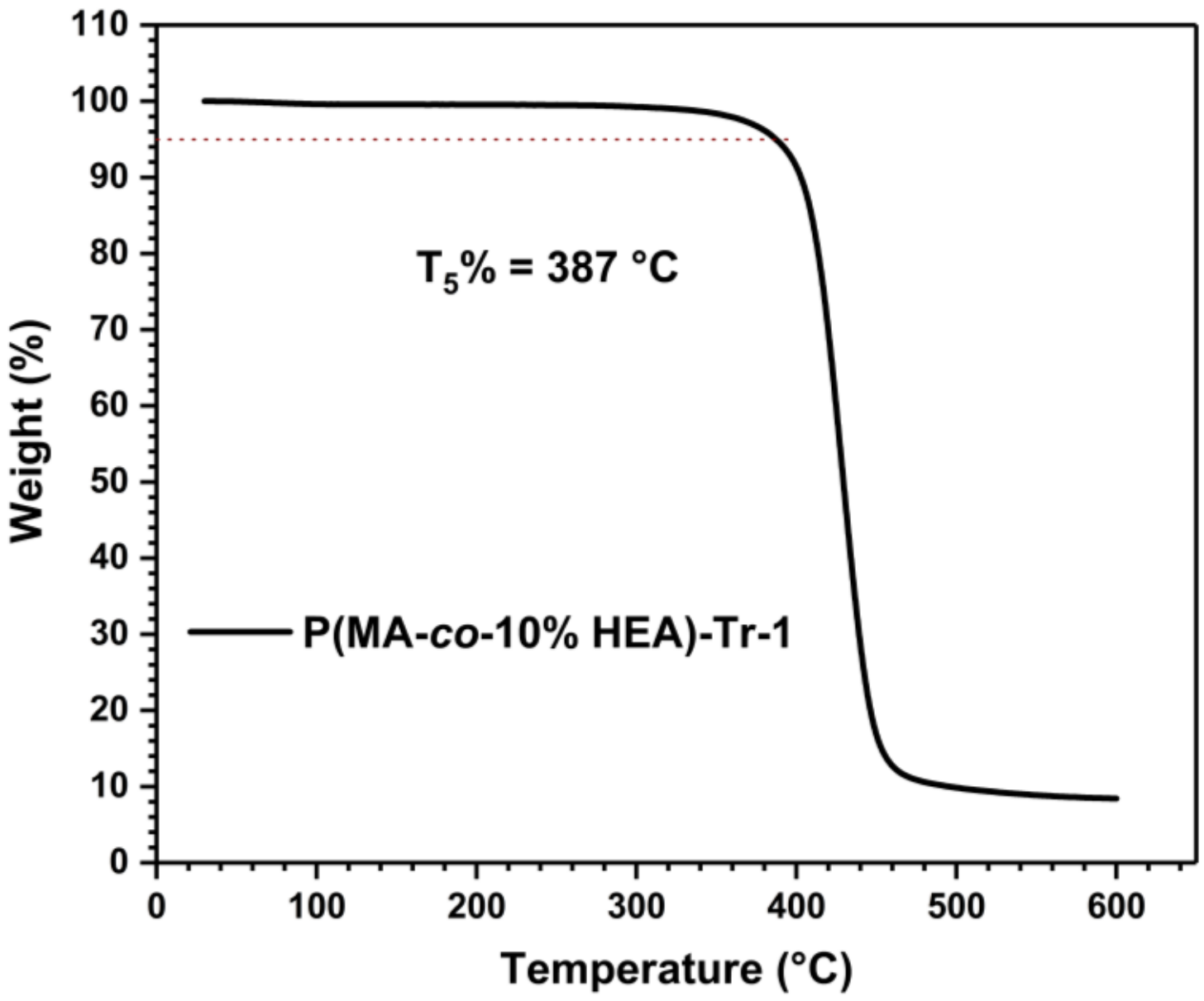

Figure S29. Thermogravimetric analysis curve of **P(MA-*co*-10%HEA)-Tr-1**. The red dashed line indicates the temperature at 5% weight loss (T_5%), which occurs at 387 °C

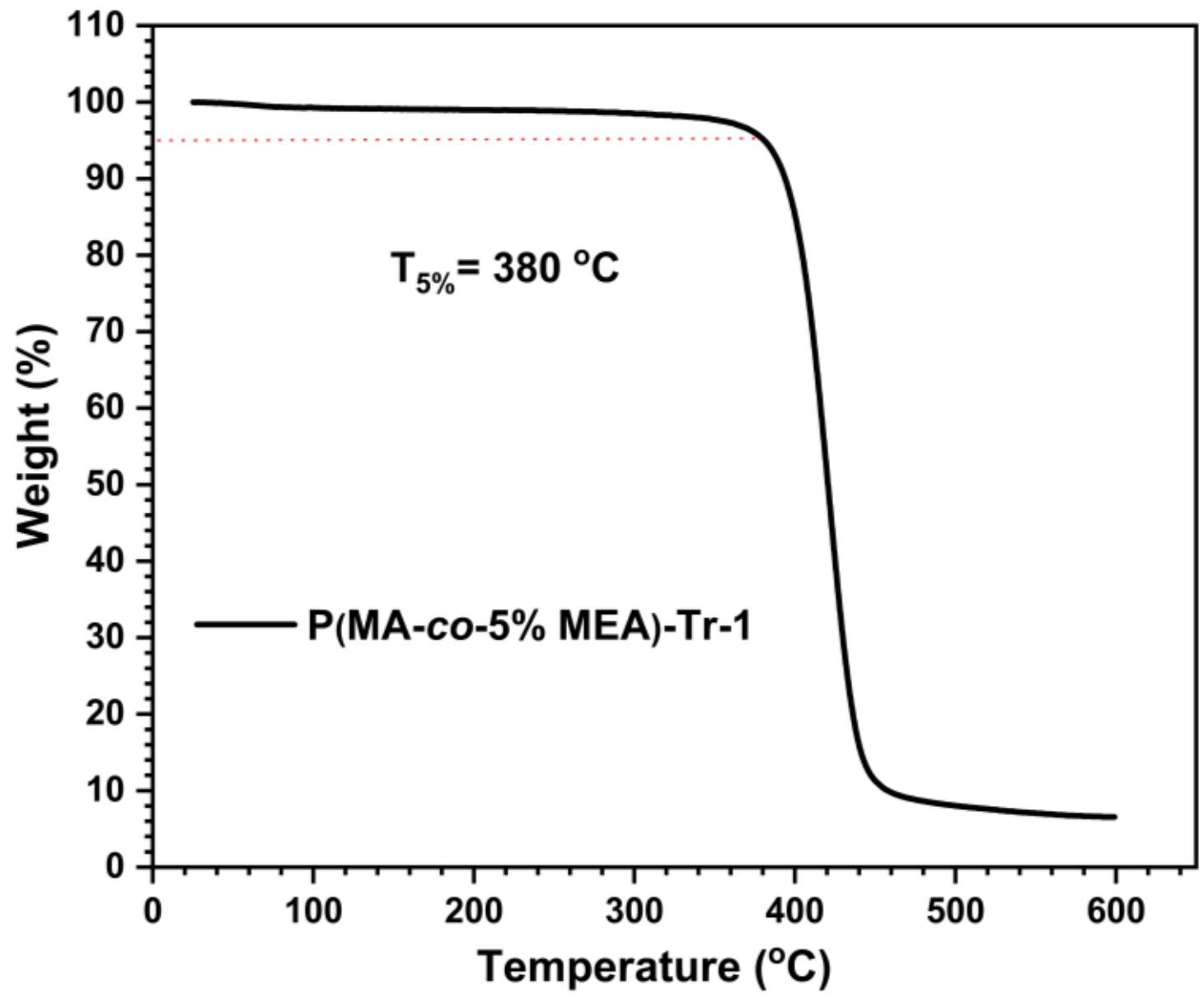

Figure S30. Thermogravimetric analysis curve of **P(MA-*co*-5%MEA)-Tr-1**. The red dashed line indicates the temperature at 5% weight loss (T_5%), which occurs at 380 °C.

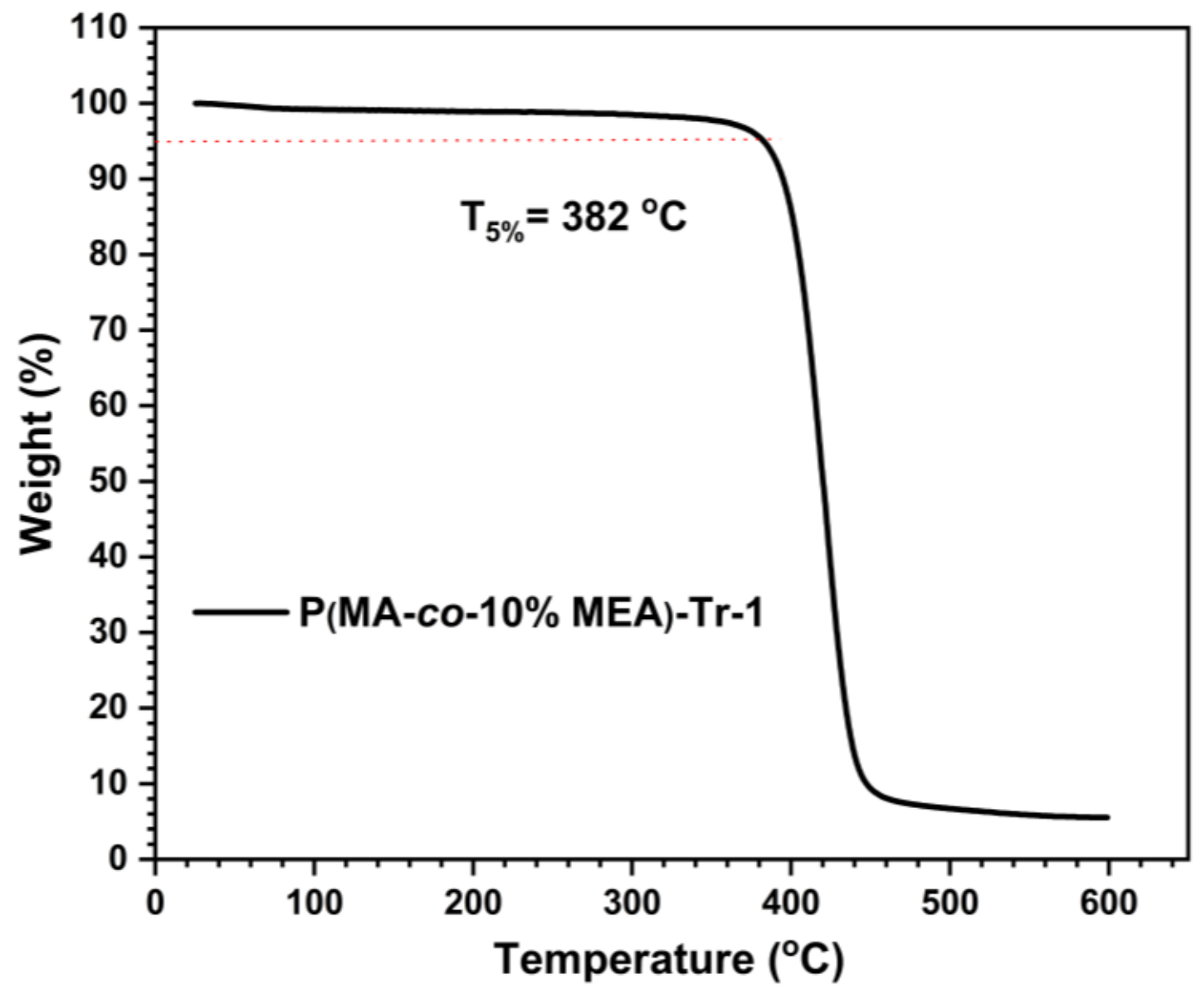

Figure S31. Thermogravimetric analysis curve of **P(MA-*co*-10%MEA)-Tr-1**. The red dashed line indicates the temperature at 5% weight loss (T_5%), which occurs at 382 °C.

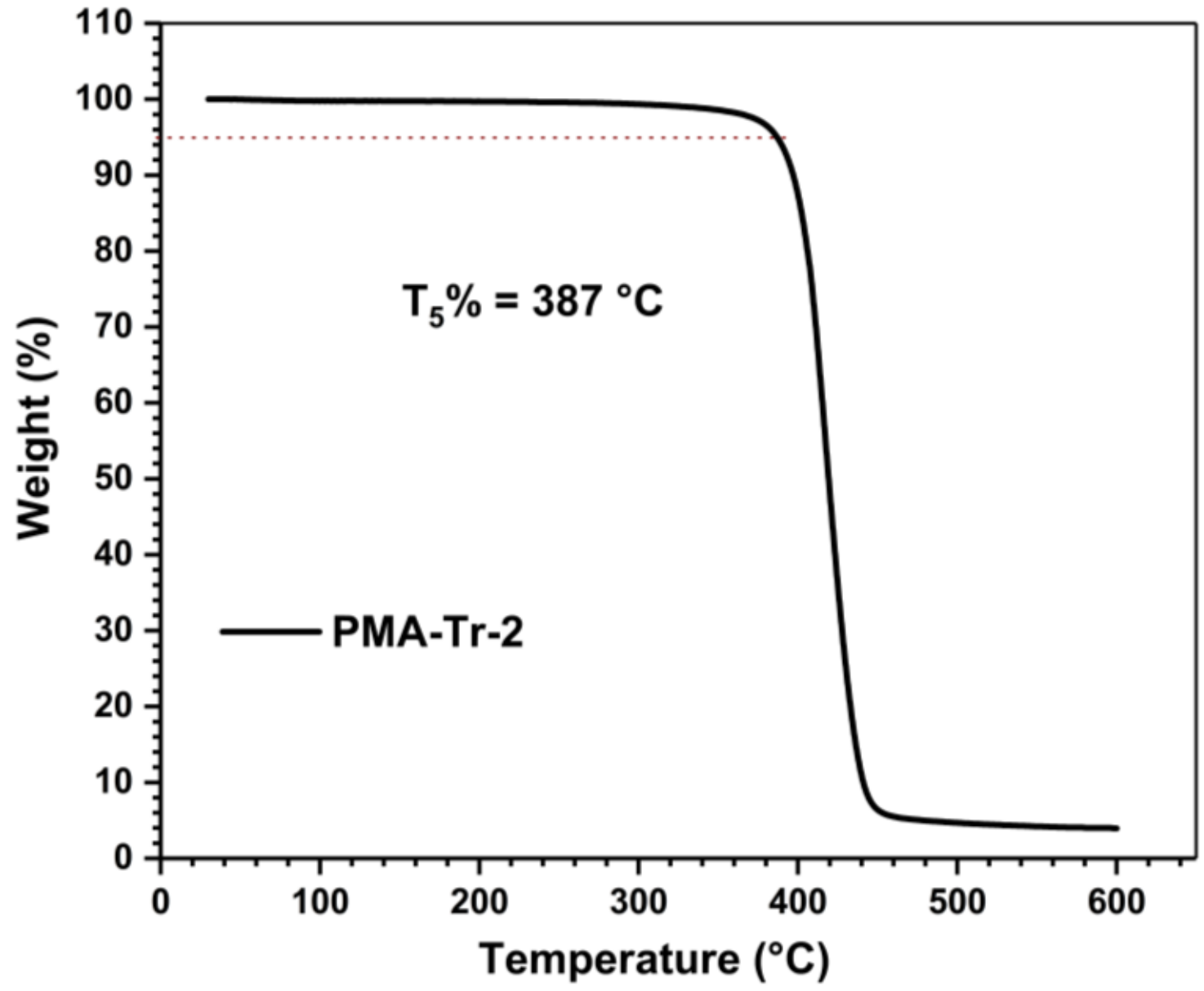

Figure S32. Thermogravimetric analysis curve of **PMA-Tr-2**. The red dashed line indicates the temperature at 5% weight loss (T_5%), which occurs at 387 °C.

7.2 Differential Scanning Calorimetry (DSC)

All samples exhibited similar thermal profiles, with glass transition temperatures (T_g) observed in the range of 18–20 °C (Figure S33–S38 report the second heating and cooling cycles). The incorporation of HEA/MEA at 5% and 10% did not significantly alter the T_g, suggesting that the copolymerization has a minimal effect on polymer chain mobility at these concentrations.

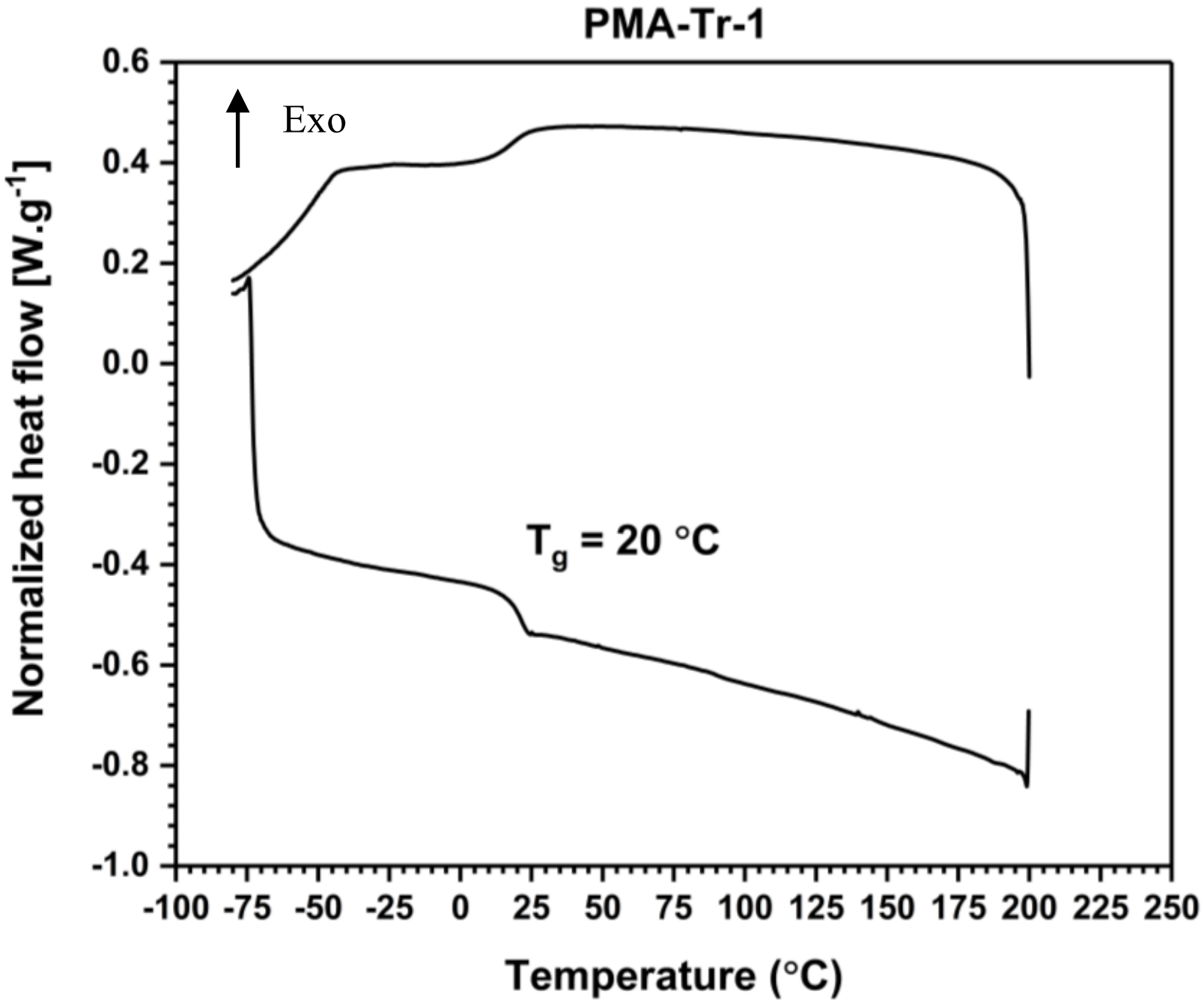

Figure S33. Differential scanning calorimetry of **PMA-Tr-1** (heating/cooling rates of 10 ^{o}C min^{-1}; second cycle).

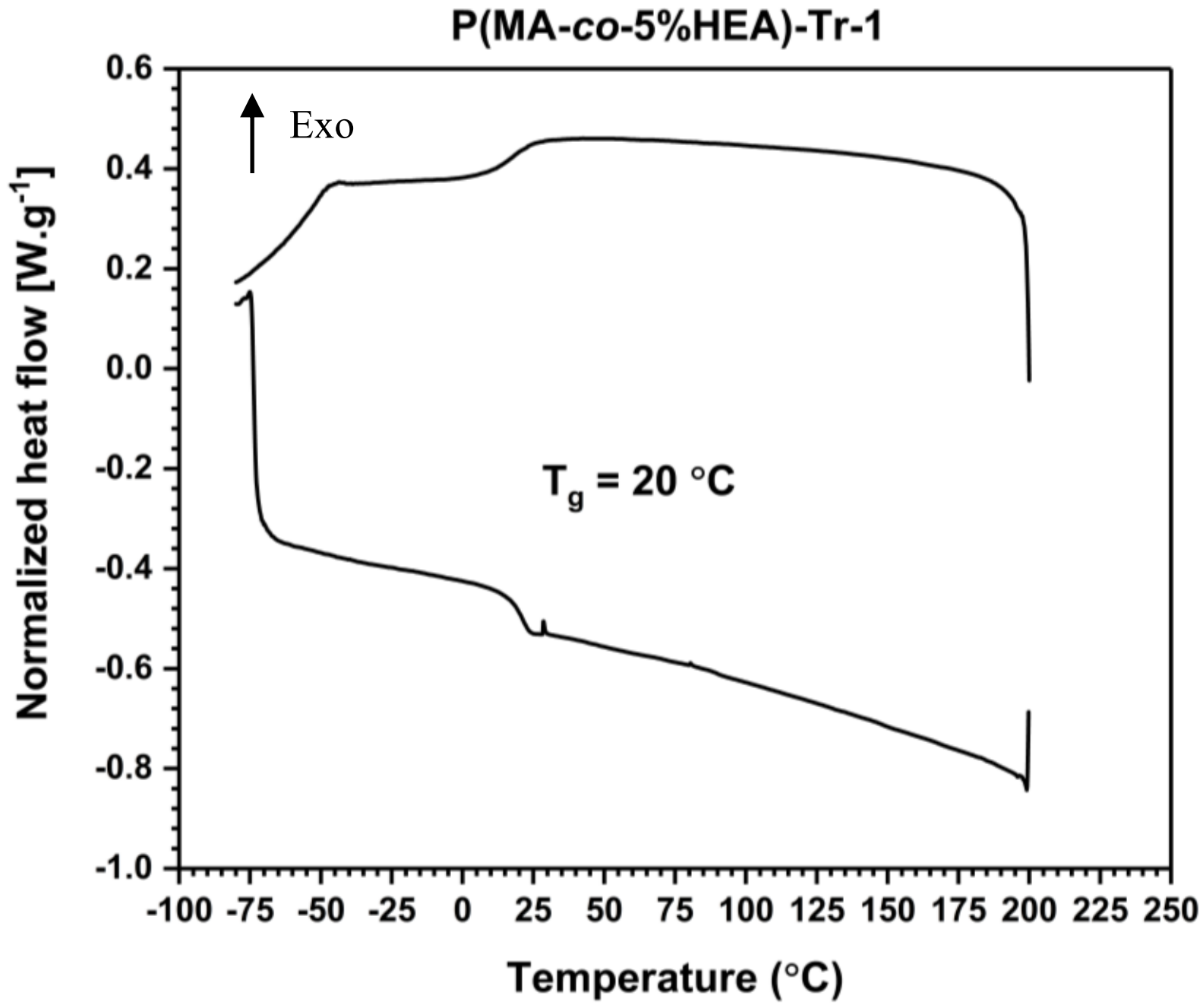

Figure S34. Differential scanning calorimetry of **P(MA-*co*-5%HEA)-Tr-1** (heating/cooling rates of 10 °C min^{-1}; second cycle).

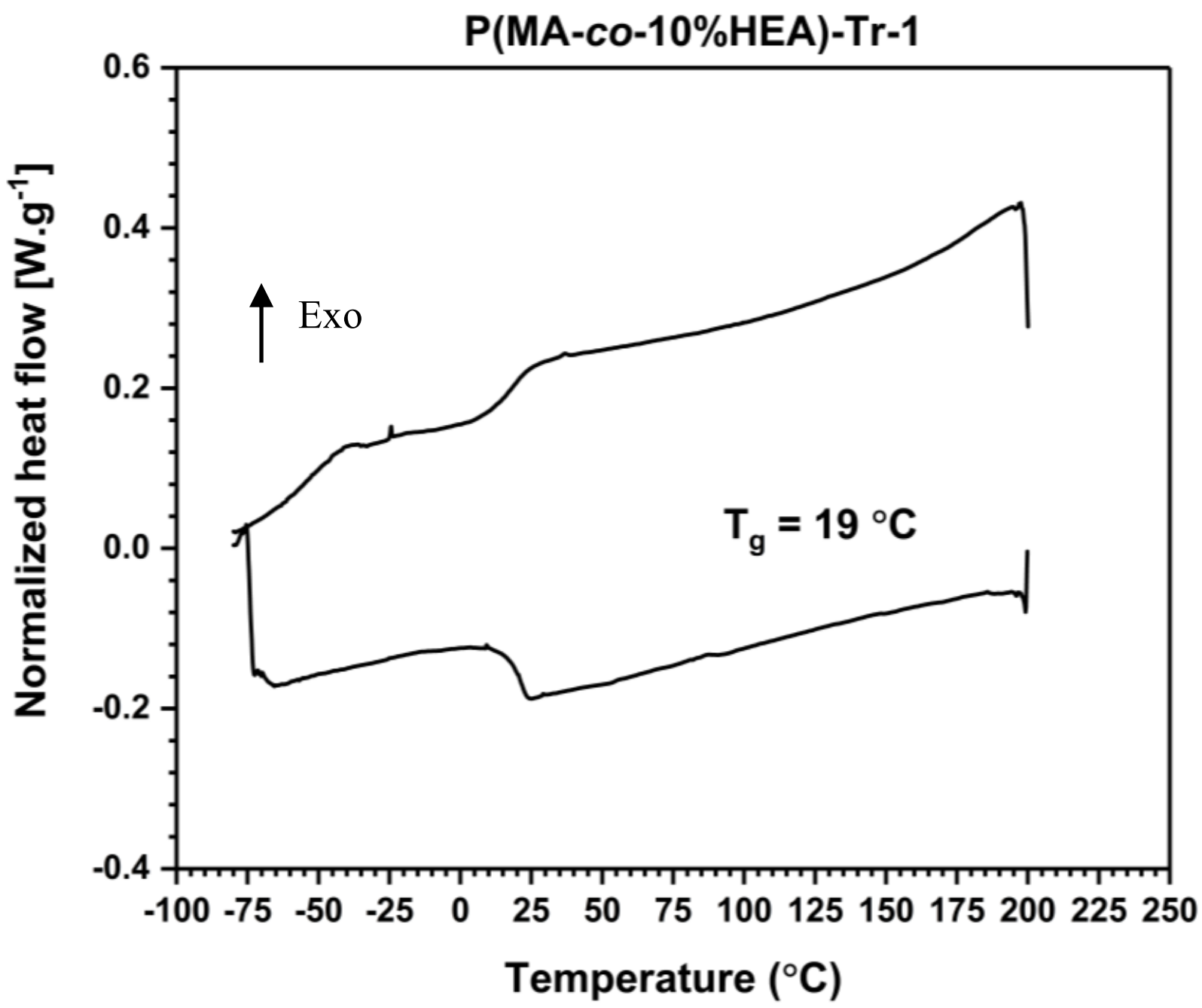

Figure S35. Differential scanning calorimetry of **P(MA-*co*-10%HEA)-Tr-1** (heating/cooling rates of 10 °C min^{-1}; second cycle).

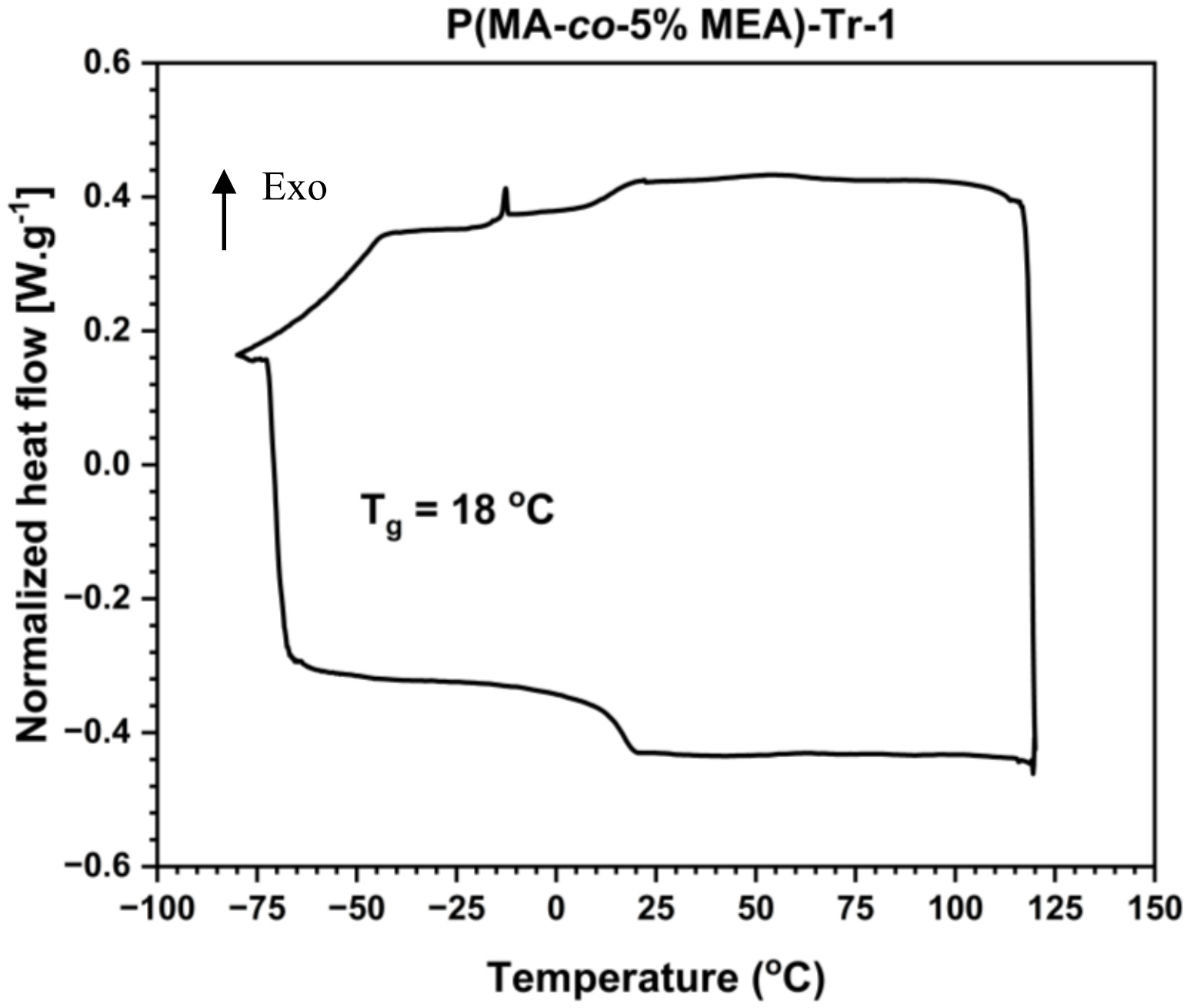

Figure S36. Differential scanning calorimetry of **P(MA-*co*-5%MEA)-Tr-1** (heating/cooling rates of 10 °C min^{-1}; second cycle).

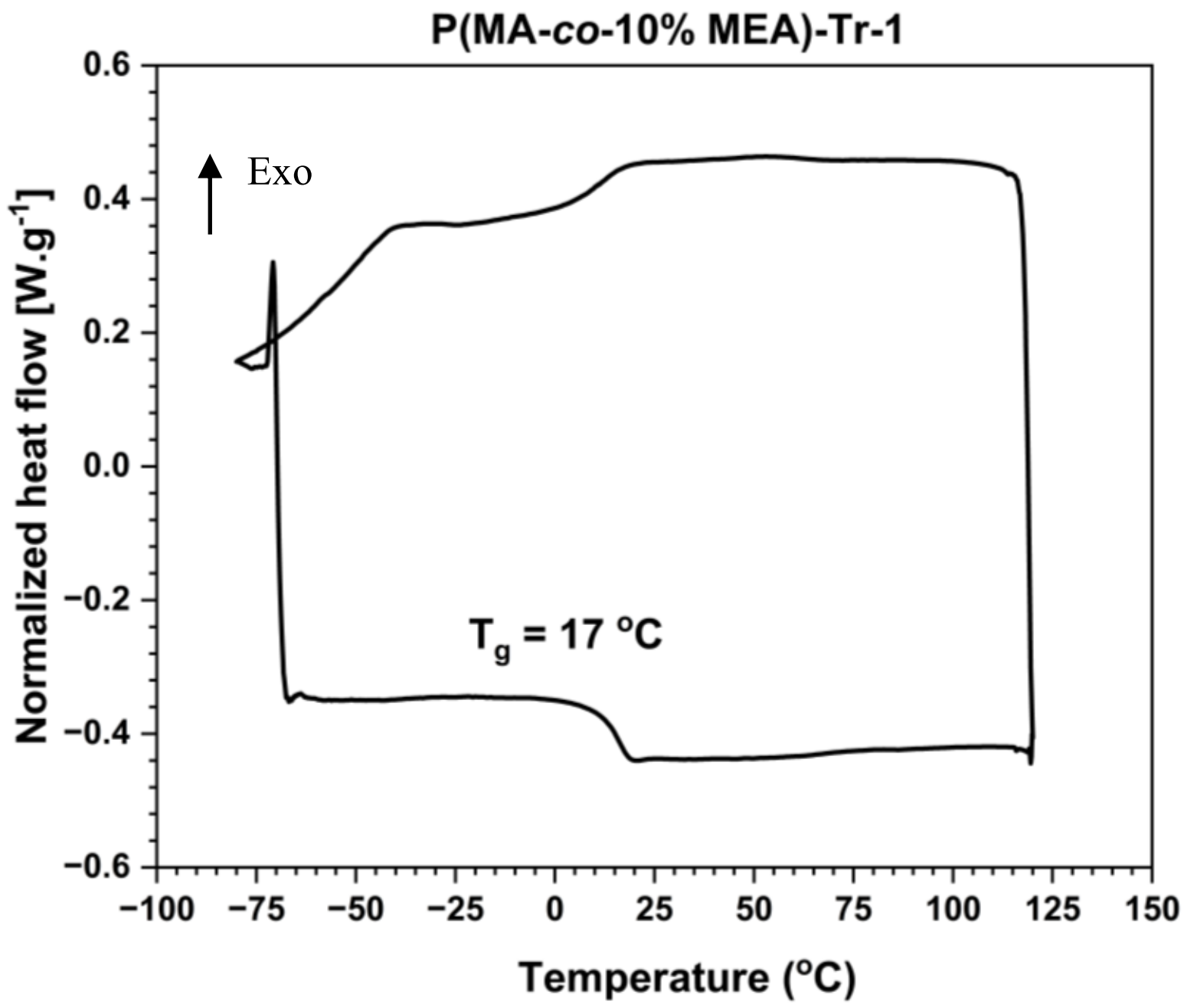

Figure S37. Differential scanning calorimetry of **P(MA-*co*-10%MEA)-Tr-1** (heating/cooling rates of 10 °C min^{-1}; second cycle).

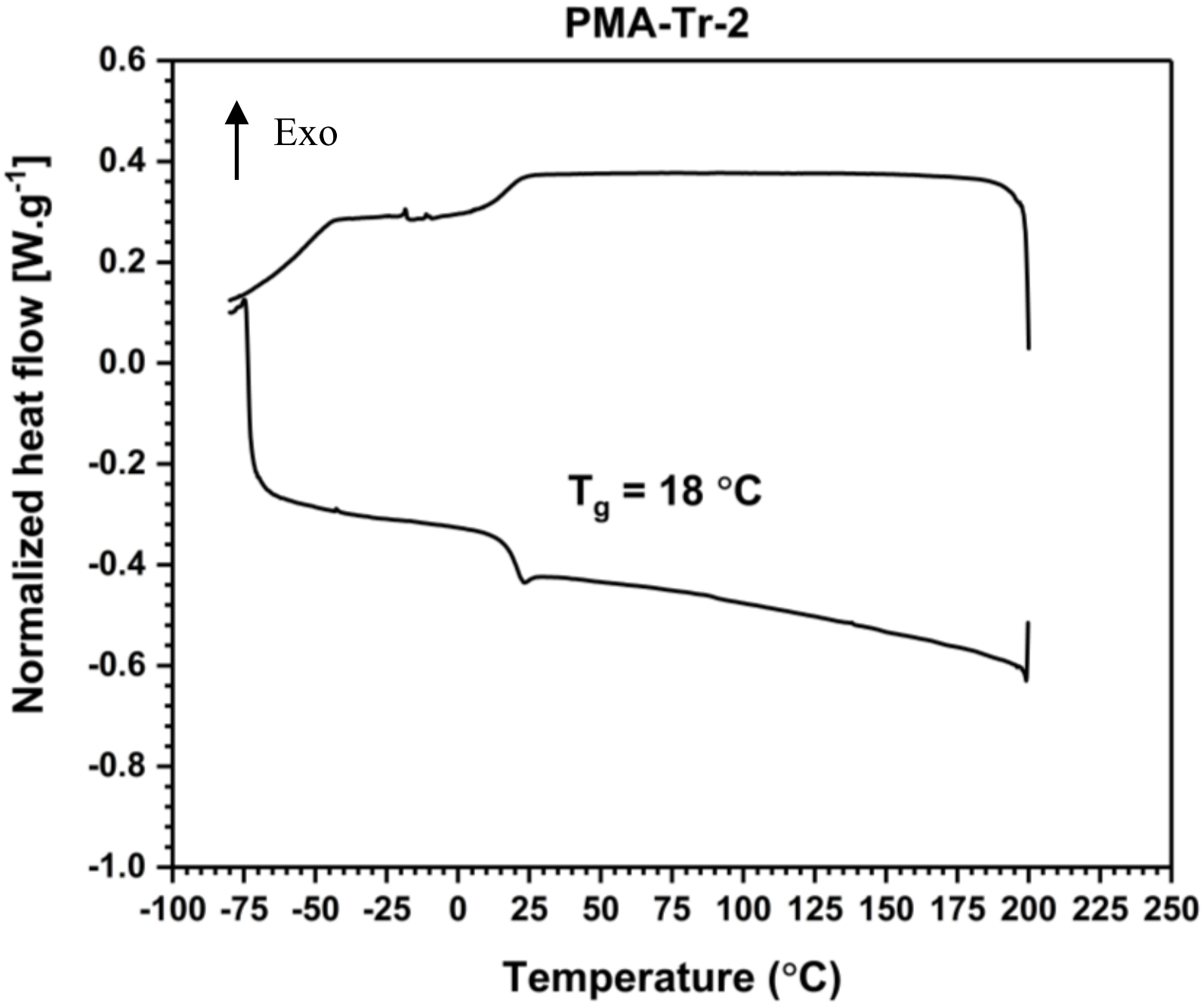

Figure S38. Differential scanning calorimetry of **PMA-Tr-2** (heating/cooling rates of 10 ^{o}C min^{-1}; second cycle).

8. Swelling Index (SI) and Gel Content (GC) Calculation:

The swelling index (SI) and gel content (GC) of polymer networks **PMA-Tr-1**, **P(MA-*co*-5%HEA/MEA)-Tr-1**, **P(MA-*co*-10%HEA/MEA)-Tr-1**, and **PMA-Tr-2** were investigated to assess network integrity and the extent of cross-linking. The swelling behavior of the polymer networks was evaluated using tetrahydrofuran (THF).
Polymer films were first dried, cut into rectangular specimens (2.0 x 0.3 cm), and then weighed to determine the initial dry mass (M_0) of each sample (approximately 20 mg). For each polymer composition, five replicates were prepared to ensure reproducibility. Each sample was immersed in 3–4 mL of THF in sealed vials and kept at room temperature (approximately 25 °C) for 24 hours to allow equilibrium swelling. After this period, the swollen samples were removed using tweezers, gently dried with filter paper to remove excess surface solvent, and immediately weighed to obtain the swollen mass (M_1). The swelling index was calculated by using Eq. 1

$$\text{Swelling Index (\%)} = \left(\frac{M_1 - M_0}{M_0}\right) \times 100 \qquad \text{Eq. 1}$$

The swelling percentage for **PMA-Tr-1**, **P(MA-*co*-5%HEA)-Tr-1**, and **P(MA-*co*-10%HEA)-Tr-1** are 181.6 ± 9.9, 192.2 ± 3.8, and 203.3 ± 3.9, respectively (Table S1). The swelling index of materials incorporating **Tr-1** increased upon increasing the **HEA** content, following the same trend as HEA, which can be attributed to changes in polymer–solvent affinity. The swelling percentage for **PMA-Tr-2** is 337.9 ± 10.5%, which is significantly higher than all **Tr-1-**featuring polymer networks. This difference is likely associated with the distinct chemical structures of cross-linkers (**Tr-1** vs **Tr-2**) and their influence on network formation.
To further evaluate the extent of cross-linking and the insoluble fraction of the networks, GC was calculated. The swollen samples were dried at 60 °C in a vacuum oven for 24 hours to remove residual solvent. After drying, the samples were cooled to room temperature and immediately weighed to obtain the final dried mass (M_2).
The GC was calculated using Eq. 2

$$\text{Gel Content (\%)} = \left(\frac{M_2}{M_0}\right) \times 100 \qquad \text{Eq. 2}$$

GC remains essentially constant across all polymer networks incorporating **Tr-1**, with values of approximately 95%, indicating comparable densities of cross-linking (Table S1). **PMA-Tr-2** exhibits a slightly lower value of approximately 90% (Table S1). Nevertheless, the high gel content indicates effective network formation and confirms a somewhat consistent level of cross-linking in all polymer systems.

Table S1. Swelling index (SI) and gel content (GC), and related standard deviations (measurements on five replicates) of cross-linked polymers containing **Tr-1** and **Tr-2**.

Samples Names	Swelling Index (SI)	Gel content (GC)
**PMA-Tr-1**	181.6 ± 9.9	95.3 ± 1.1
**P(MA-*co*-5% HEA)-Tr-1**	192.2 ± 3.8	97.5 ± 0.9
**P(MA-*co*-10% HEA)-Tr-1**	203.3 ± 3.9	97.3 ± 0.8
**P(MA-*co*-5%MEA)-Tr-1**	170.5 ± 4.5	95.7 ± 0.5
**P(MA-*co*-10%MEA)-Tr-1**	173.2 ± 3.7	96.1 ± 0.7
**PMA-Tr-2**	337.9 ± 10.5	89.7 ± 2.4

9. Dynamic Mechanical Analysis (DMA) of cross-linked polymer films

DMA was carried out on **PMA-Tr-1**, **P(MA-*co*-5%HEA)-Tr-1**, **P(MA-*co*-10%HEA)-Tr-1**, and **PMA-Tr-2** films to assess network integrity and cross-linking. Prismatic rectangular samples (≈15 × 5 × 0.2 mm^3) were analyzed by DMA using a Discovery DMA 850 (TA Instruments) equipped with a film tension clamp and an ACS3+ cooling system. Measurements were performed over a temperature range of –50 °C to 125 °C at 1 Hz, with 0.02 % strain and a heating rate of 2 °C min^{-1}.

The glass transition temperatures **(**T_g**)** of all samples were narrowly distributed between 33.4 °C and 35.7 °C (Table S2), with full-width at half-maximum (FWHM) values of 24–26 °C, indicating similar chain mobility and transition breadth across the series.

The storage modulus (E′) at −50 °C was 3050 MPa for **PMA-Tr-1**, while HEA-containing copolymers (**P(MA-*co*-5%HEA)-Tr-1** and **P(MA-*co*-10%HEA)-Tr-1** showed slightly lower values (2497 and 2853 MPa, respectively). **PMA-Tr-2** displayed mechanical behavior similar to **PMA-Tr-1**.

At 120 °C, corresponding to the rubbery plateau, E′ values remain comparable (0.518–0.548 MPa), supporting the fact that all network structures possess a similar density of cross-links (Table S2).

Table S2. Parameters determined upon studying all crosslinked polymer films containing **Tr-1** and **Tr-2** mechanophores with DMA. The glass transition temperature (T_g) was determined from the maximum of the tan δ peak, and the transition breadth was quantified by the full width at half maximum (FWHM). The storage modulus in the glassy region (E'_g) was measured at −50 °C, and the storage modulus in the rubbery region (E'_r) was measured at 120 °C.

**Sample**	$T_{g\text{-tan}\delta}$ **[ºC]**	**FWHM [ºC]**	$E'_{g(-50°C)}$ **[MPa]**	$E'_{r(120°C)}$ **[MPa]**
**PMA-Tr-1**	33.4	25	3050	0.545
**P(MA-*co*-5%HEA)-Tr-1**	35.7	24	2497	0.527
**P(MA-*co*-10%HEA)-Tr-1**	34.6	24	2853	0.548
**PMA-Tr-2**	34.1	26	3184	0.518

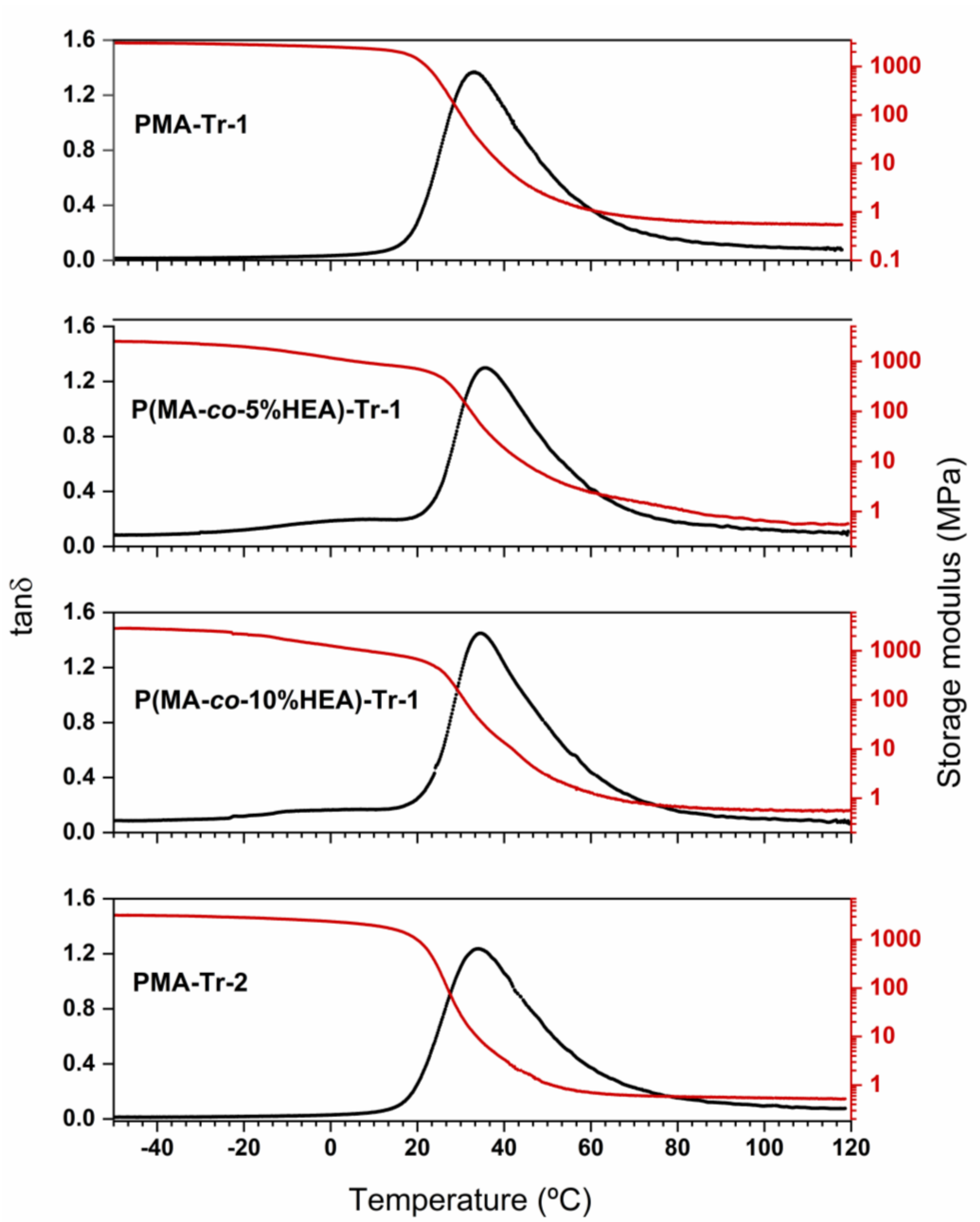

Figure S39. Dynamic mechanical analysis (DMA) of **PMA-Tr-1**, **P(MA-*co*-5%HEA)-Tr-1**, **P(MA-*co*-10%HEA)-Tr-1**, and **PMA-Tr-2** (from top to bottom), with the storage modulus trace shown in red and that of tan δ in black.

10. Mechanical activation of crosslinked polymer films and their dissociation mechanism

Photographs of **PMA-Tr-1**, **P(MA-*co*-5 %HEA)-Tr-1**, **P(MA-*co*-10 %HEA)-Tr-1**, and **PMA-Tr-2**, prior to tensile stretching are shown in Table S3. All films were colorless before stretching. Upon tensile deformation, **PMA-Tr-1** developed a distinct orange coloration (Table S3). In contrast, **P(MA-*co*-5 %HEA)-Tr-1** films exhibited a light bluish-green coloration upon stretching. This effect was further amplified in **P(MA-*co*-10 %HEA)-Tr-1**, where tensile deformation produced an intense bluish-green coloration (Table S3). These observations strongly suggest that higher HEA content—and thus stronger hydrogen-bonding interactions—correlates with enhanced heterolytic dissociation of the mechanophore. Similarly, **PMA-Tr-2** displayed a pronounced bluish-green coloration after deformation.

EPR spectroscopy revealed the presence of radical species in stretched **PMA-Tr-1** and **P(MA-*co*-5 %HEA)-Tr-1**, whereas no radicals were detected in **P(MA-*co*-10 %HEA)-Tr-1** and **PMA-Tr-2**.

Table S3. Cross-linked polymer films containing **Tr-1** or **Tr-2** before tensile stretching (colorless) and after stretching (colored). The stretched films were analysed by electron paramagnetic resonance (EPR) to examine the mechanophore dissociation mechanism.

Sr. no	Polymers	Polymer films before tensile stretching	Polymer films after tensile stretching	Observed cleavage
1	**PMA-Tr-1**	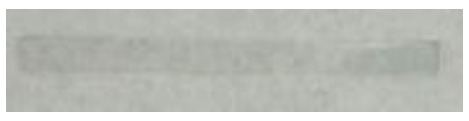	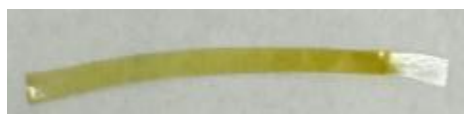	Predominant homolytic
2	**P(MA-*co*-5%HEA)-Tr-1**	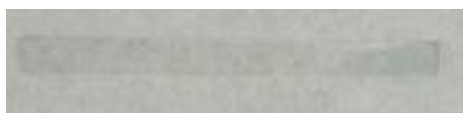	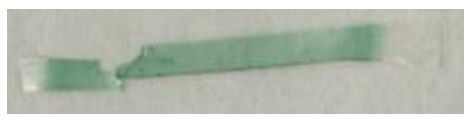	Heterolytic (major path) Homolytic (minor path)
3	**P(MA-*co*-10%HEA)-Tr-1**	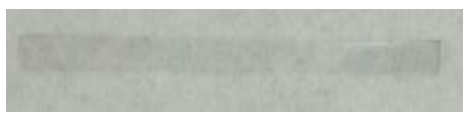	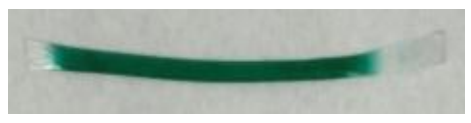	Predominant heterolytic
4	**P(MA-*co*-5%MEA)-Tr-1**	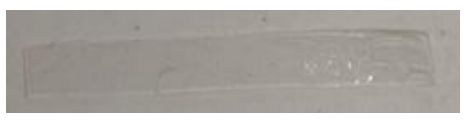	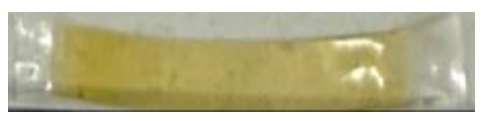	Homolytic
5	**P(MA-*co*-10%MEA)-Tr-1**	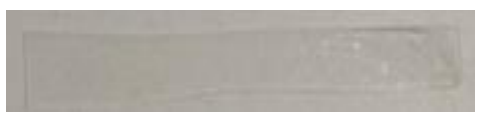	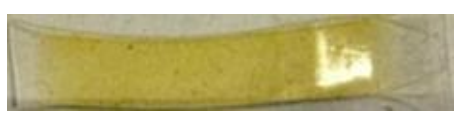	Homolytic
6	**PMA-Tr-2**	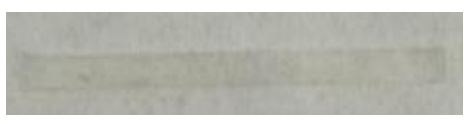	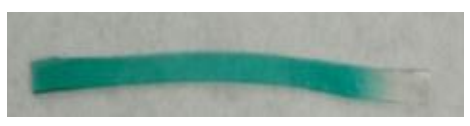	Heterolytic

11. UV–vis spectrum of **4**.

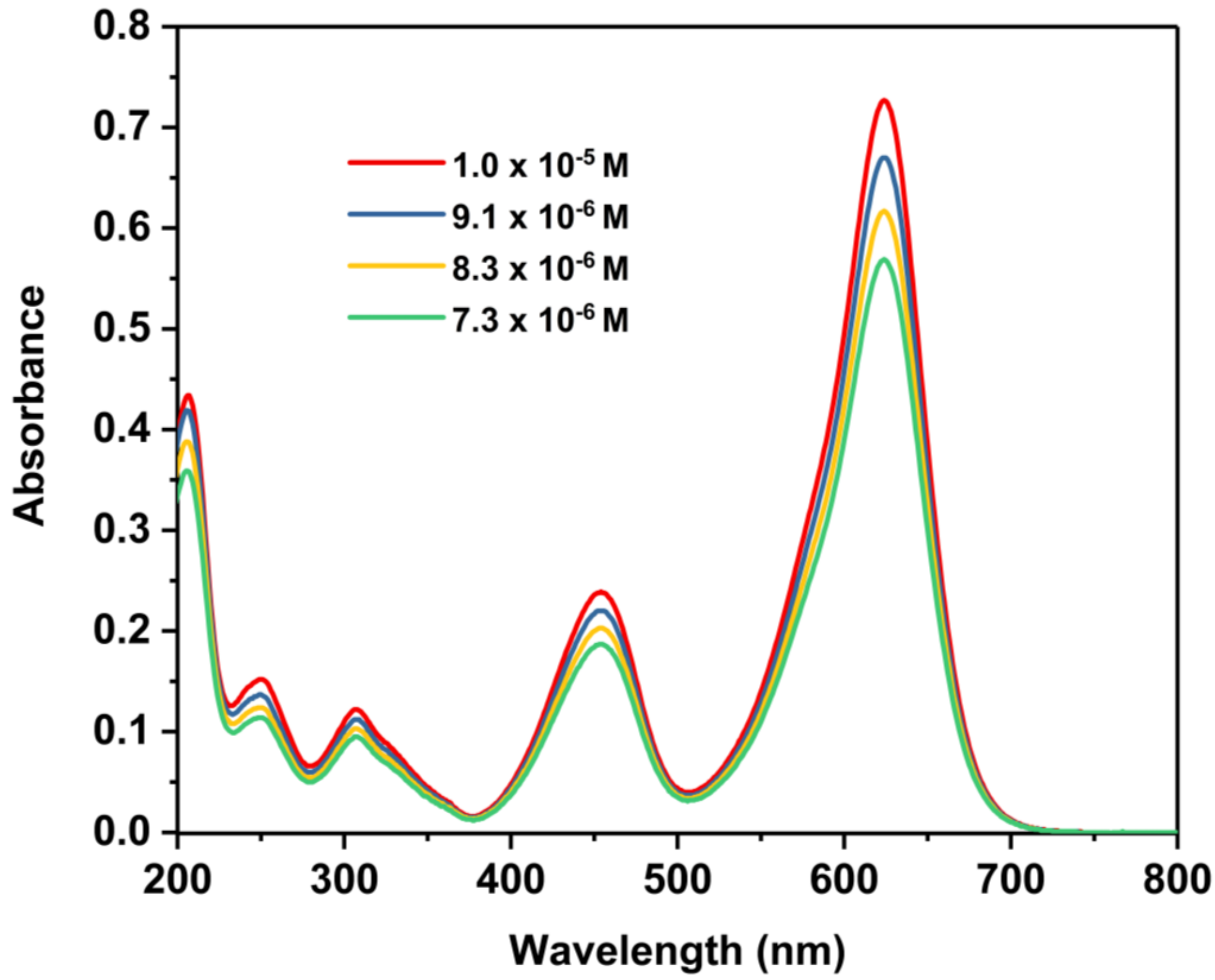

Figure S40. UV–vis absorption spectrum of **4** recorded in acetonitrile (CH_3CN) at four different concentrations at 25 °C.

12. Solvatochromism of Nile Red in **PMA-Tr-1** and **P(MA-*co*-HEA/MEA)-Tr-1** networks

The local polarity of the polymer films was investigated using Nile Red as a solvatochromic fluorescent probe. A stock solution of Nile Red (1×10^{-6} M) was prepared in THF (200 mL).

PMA-Tr-1, **P(MA-*co*-5%HEA/MEA)-Tr-1**, and **P(MA-*co*-10%HEA/MEA)-Tr-1** were immersed in the Nile Red solution and allowed to soak for 24 hours to enable dye uptake into the polymer matrix. After soaking, the films were removed from the solution, gently blotted to remove excess solvent, and dried in a vacuum oven at 60 °C for 24 hours.

The Nile Red-loaded films were then subjected to fluorescence spectroscopy. Emission spectra were recorded under identical instrumental conditions, and the wavelength of maximum emission (λ_{max}) was determined for each sample. The fluorescence measurements revealed that **PMA-Tr-1** exhibited an emission maximum at 584 nm, while **P(MA-*co*-5%HEA)-Tr-1** and **P(MA-*co*-10%HEA)-Tr-1** showed slightly red-shifted emission maxima at 587 and 591 nm, respectively (Figure S41). Additionally, **P(MA-*co*-5%MEA)-Tr-1** and **P(MA-*co*-10%MEA)-Tr-1** exhibited only a shift of 1–2 nm relative to **PMA-Tr-1** (Figure S41).

Overall, the observed shifts in λ_{max} are within 7 nm, indicating that all polymer samples possess comparable bulk polarity.

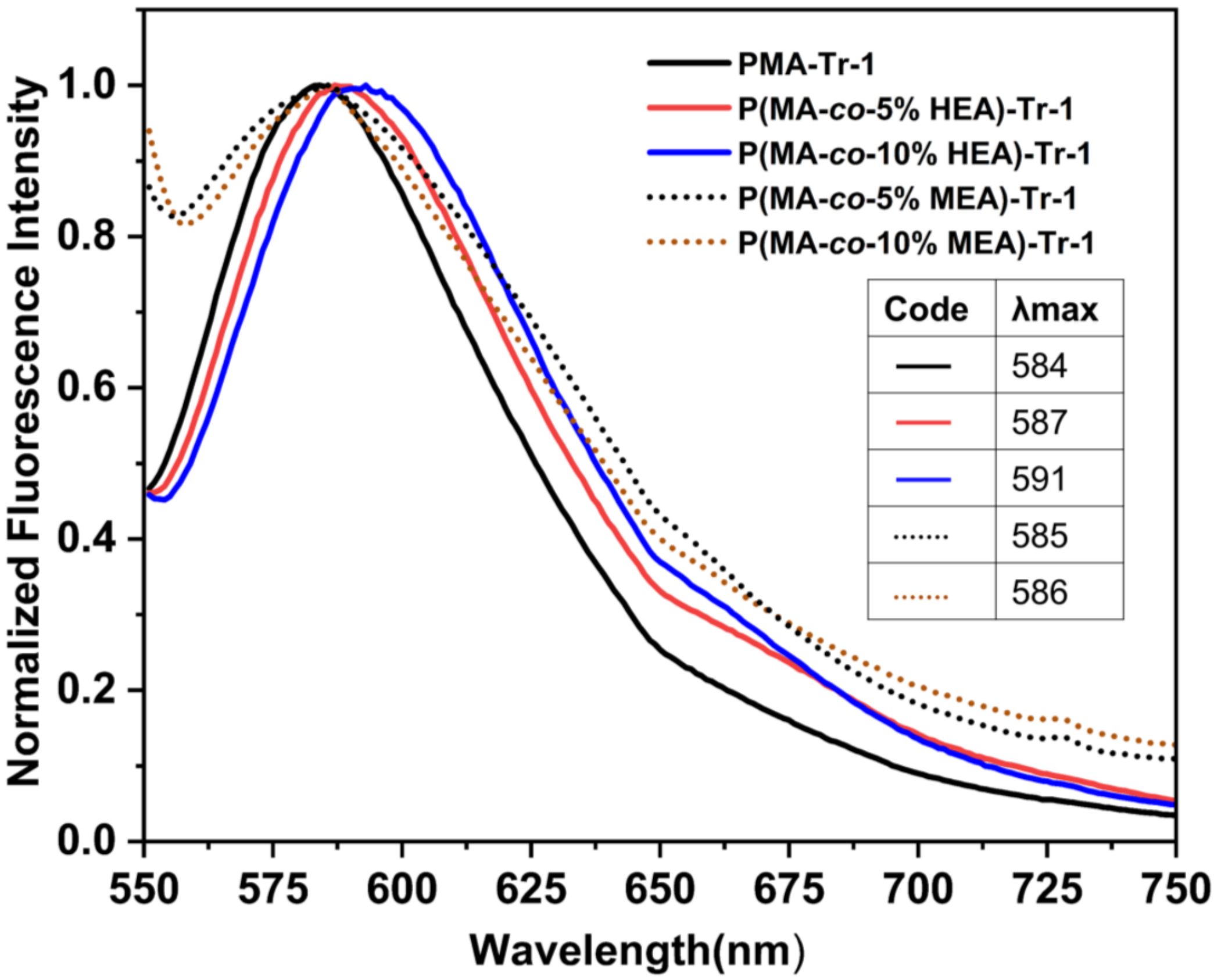

Figure S41. Emission profiles of Nile Red in **PMA-Tr1** and **P(MA-*co*-5/10%HEA/MEA)-Tr-1** networks.

13. Evaluation of Residual Monomer and Mechanophore Content and Mechanical Response of Washed Polymer Films

To validate the composition of the polymer networks and assess the presence of any unreacted monomer or mechanophore, extensive solvent extraction experiments were performed. Approximately 200 mg samples of **PMA-Tr-1**, **P(MA-*co*-5%HEA)-Tr-1**, **P(MA-*co*-10%HEA)-Tr-1,** and **PMA-Tr-2** were washed extensively with 80 mL of THF. The extracted films were dried under vacuum at 60 °C for 24 h. In situ transmittance measurements combined with uniaxial elongation were then conducted on the dried films. As shown in Figure S42, the extracted samples exhibited transmittance profiles identical to those of the corresponding unwashed films, indicating that solvent extraction did not alter the optical properties of the polymer networks.

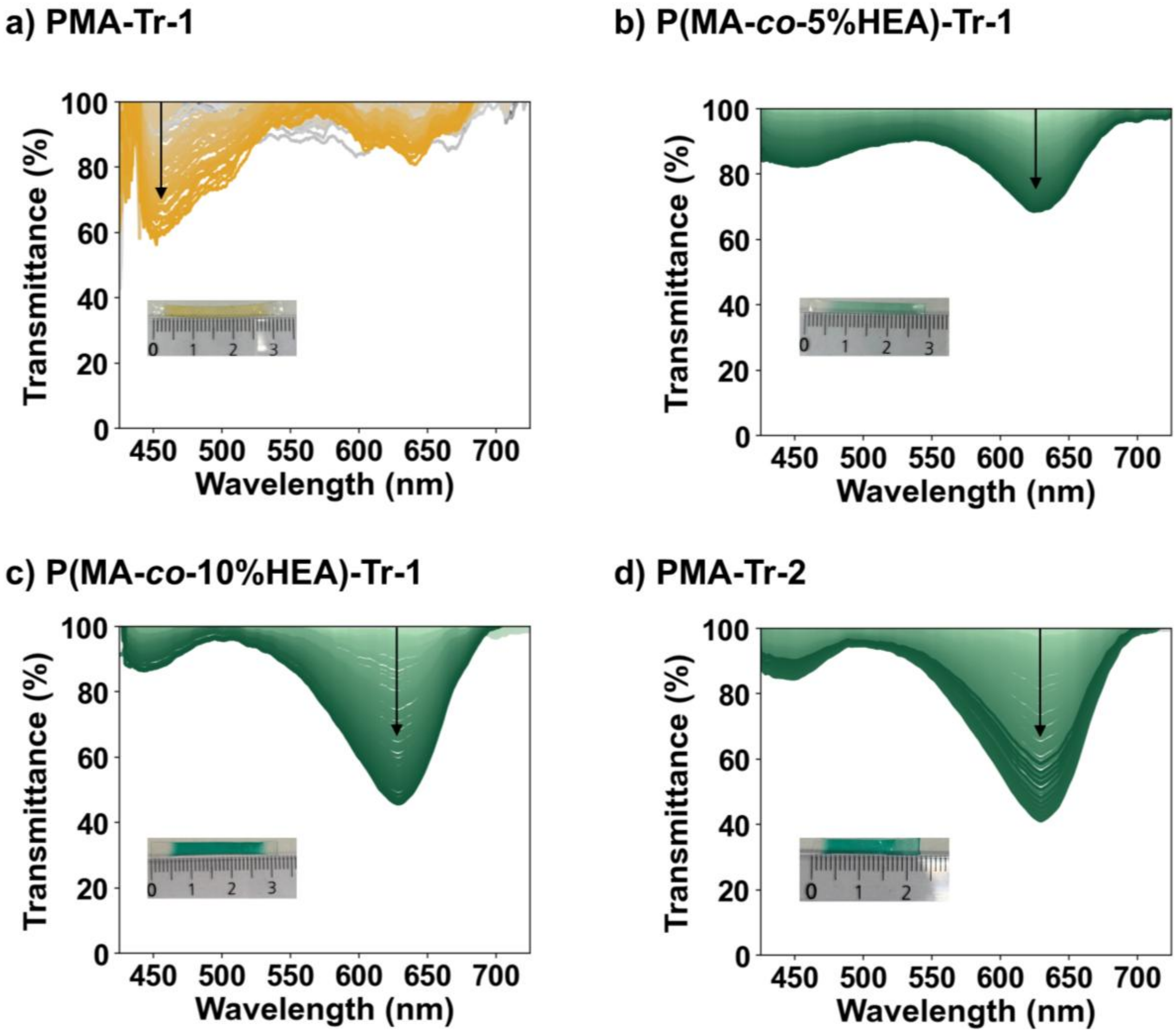

Figure S42. Transmittance spectra recorded while stretching of washed (a) **PMA-Tr-1**, (b) **P(MA-*co*-5%HEA)-Tr-1**, (c) **P(MA-*co*-10%HEA)-Tr-1** and (d) **PMA-Tr-2** films. The black arrow shows the direction of increasing applied strain (top → bottom). The insets show photographs of polymer films taken after tensile deformation, next to a ruler (dimensions in cm).

THF extracts were collected and evaporated to dryness, and the resulting residues were analyzed by ^{1}H NMR spectroscopy (Figure S43). We did not observe signals ascribable to unreacted monomers/mechanophores that leached out. These findings confirm the efficient incorporation of mechanophore into the crosslinked polymer networks.

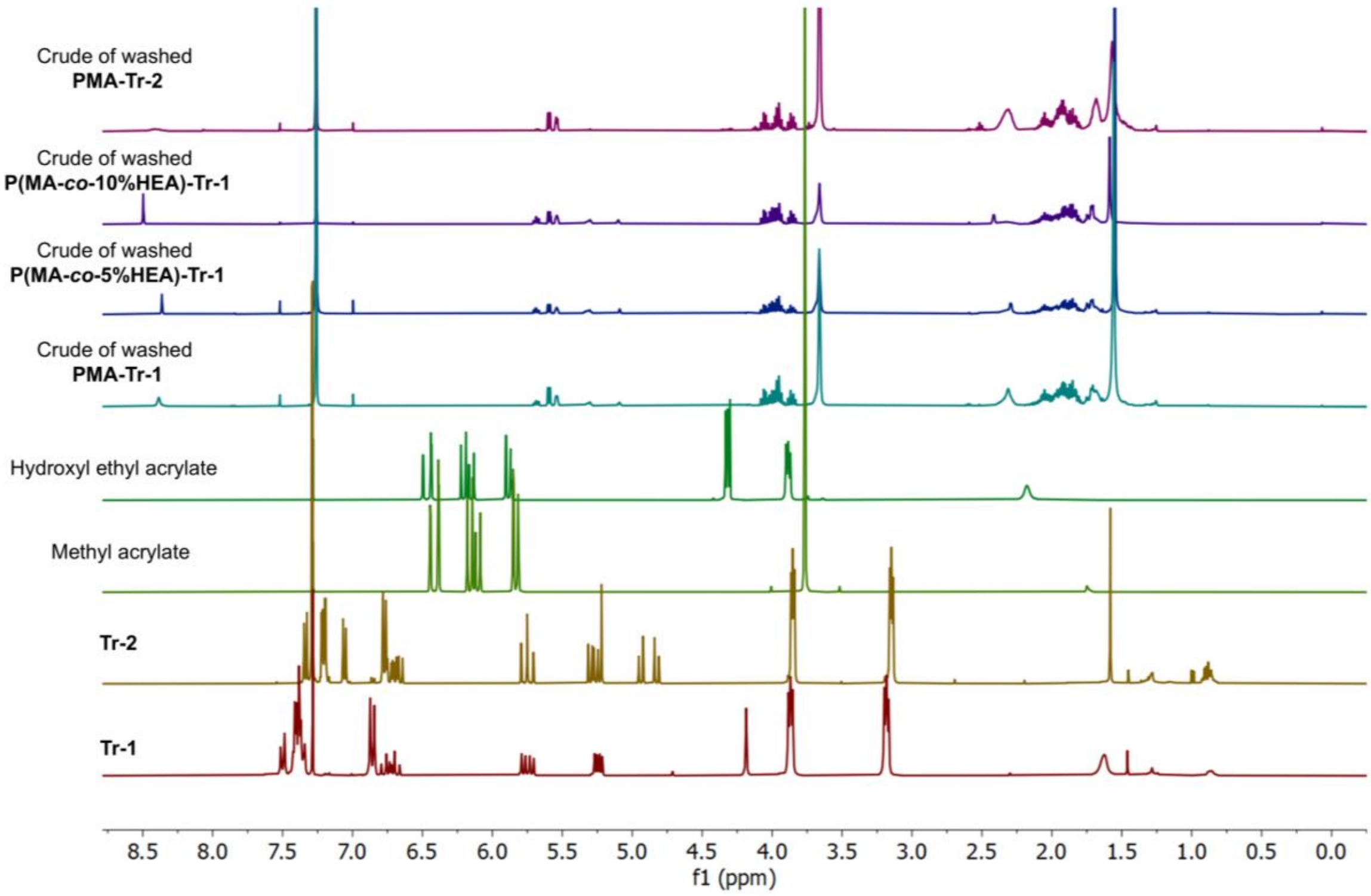

Figure S43. Stacked ^{1}H NMR spectra of **Tr-1**, **Tr-2**, MA, HEA, extracts of washed **PMA-Tr-1**, washed **P(MA-*co*-5%HEA)-Tr-1**, washed **P(MA-*co*-10%HEA)-Tr-1**, and washed **PMA-Tr-1** recorded at (400 MHz, $CDCl_3$).

14. Mechanochromic properties of **P(MA-*co*-5%MEA)-Tr-1**

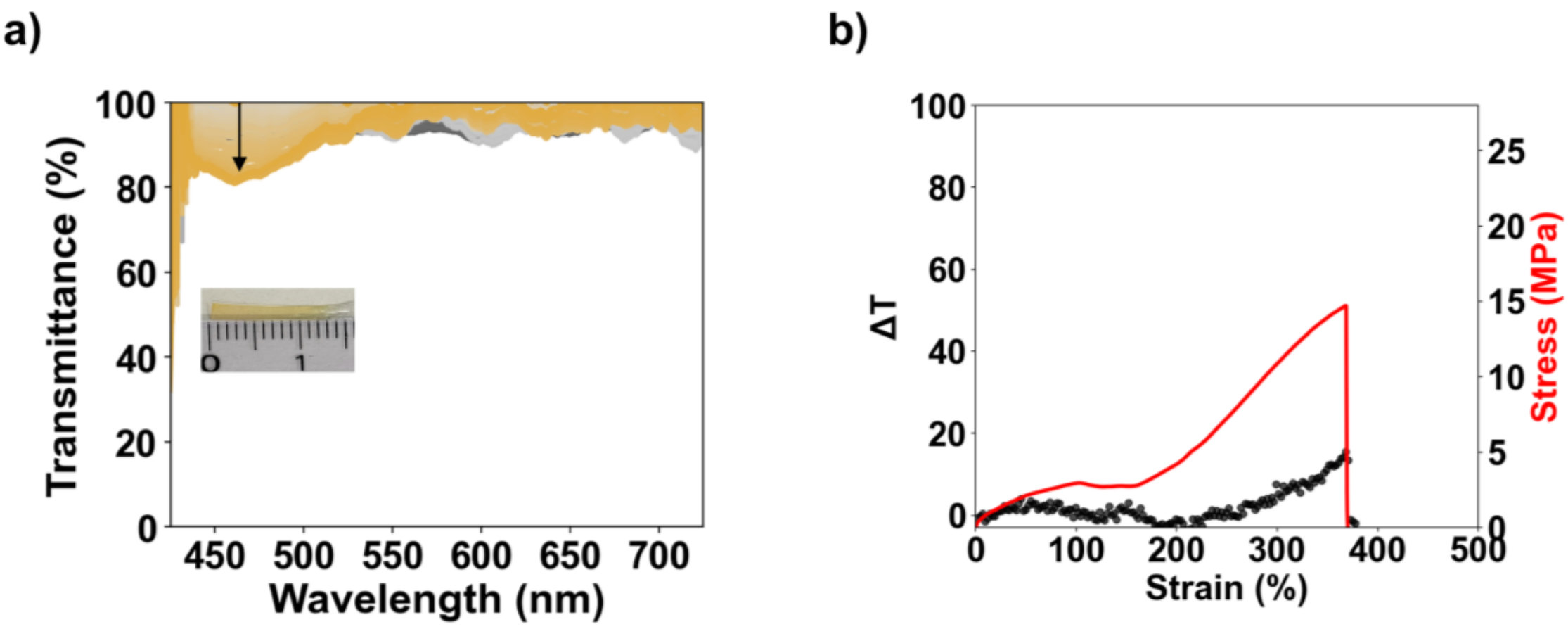

Figure S44. (a) Transmittance spectra recorded while stretching **P(MA-*co*-5%MEA)-Tr-1**. The black arrow shows the direction of increasing applied strain (top → bottom). The insets show a photograph of a polymer film taken after tensile deformation, next to a ruler (dimensions in cm). (b) Stress–strain curve (red trace) and simultaneously measured relative transmittance changes (black trace), where ΔTransmittance = T(700 nm) − T(444 nm).

15. Electron paramagnetic resonance characterization of P(MA-*co*-MEA) copolymers

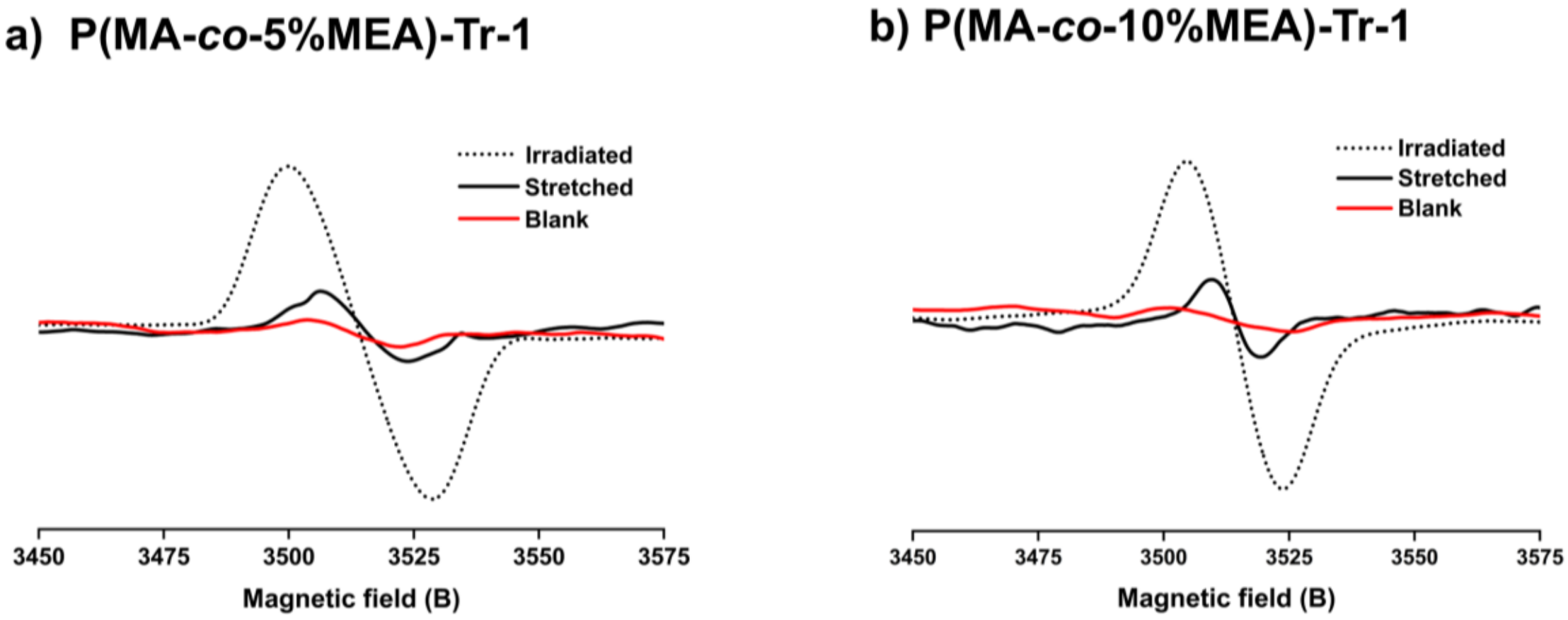

Figure S45. EPR spectra of (a) **P(MA-*co*-5%MEA)-Tr-1** and (b) **P(MA-*co*-10%MEA)-Tr-1**, before and after being manually pulled, and after irradiation with a Hg lamp (200–2000 nm, irradiation power of 100 W, irradiation time of 90 min).

16. References

(S1) Frisch, M.; Trucks, G.; Schlegel, H. B.; Scuseria, G. E.; Robb, M. A.; Cheeseman, J. R.; Scalmani, G.; Barone, V.; Mennucci, B.; Petersson, G. Gaussian 09, Gaussian. *Inc Wallingford CT* **2009**, *121*, 150–166.

(S2) Becke, A. D. A New Mixing of Hartree-Fock and Local Density-Functional Theories. *J. Chem. Phys.* **1993**, *98* (2), 1372–1377.

(S3) Lee, C.; Yang, W.; Parr, R. G. Development of the Colle-Salvetti Correlation-Energy Formula into a Functional of the Electron Density. *Phys. Rev. B* **1988**, *37* (2), 785.

(S4) Becke, A. D. Density-functional Thermochemistry. III. The Role of Exact Exchange. *J. Chem. Phys.* **1993**, *98* (7), 5648–5652.

(S5) Grimme, S. Density Functional Theory with London Dispersion Corrections. *Wiley Interdiscip. Rev. Comput. Mol. Sci.* **2011**, *1* (2), 211–228.

(S6) Hehre, W. J.; Ditchfield, R.; Pople, J. A. Self—Consistent Molecular Orbital Methods. XII. Further Extensions of Gaussian—Type Basis Sets for Use in Molecular Orbital Studies of Organic Molecules. *J. Chem. Phys.* **1972**, *56* (5), 2257–2261.

(S7) Cancès, E.; Mennucci, B.; Tomasi, J. A New Integral Equation Formalism for the Polarizable Continuum Model: Theoretical Background and Applications to Isotropic and Anisotropic Dielectrics. *J. Chem. Phys.* **1997**, *107* (8), 3032–3041.

(S8) Nikolov, I. D.; Ivanov, C. D. Optical Plastic Refractive Measurements in the Visible and the Near-Infrared Regions. *Appl. Opt.* **2000**, *39* (13), 2067–2070.

(S9) Hemmer, J. R.; Rader, C.; Wilts, B. D.; Weder, C.; Berrocal, J. A. Heterolytic Bond Cleavage in a Scissile Triarylmethane Mechanophore. *J. Am. Chem. Soc.* **2021**, *143* (45), 18859–18863.

17. Atomic coordinates of the calculated structures

Beyond the compounds discussed in the main text, we also conducted calculations on triaryl systems with *p*-nitro substitution, specifically **1-NO_2** and **2-NO_2** (Figure S46). The presence of the two nitro groups is expected to significantly discourage heterolytic cleavage, a phenomenon that should be consistent across all energy levels, regardless of the trends induced by implicit solvation. Therefore, examining **1-NO_2** and **2-NO_2** would further confirm that the conclusions remain unaffected by any over- or under-stabilization of the open-shell and closed-shell species when using DFT.

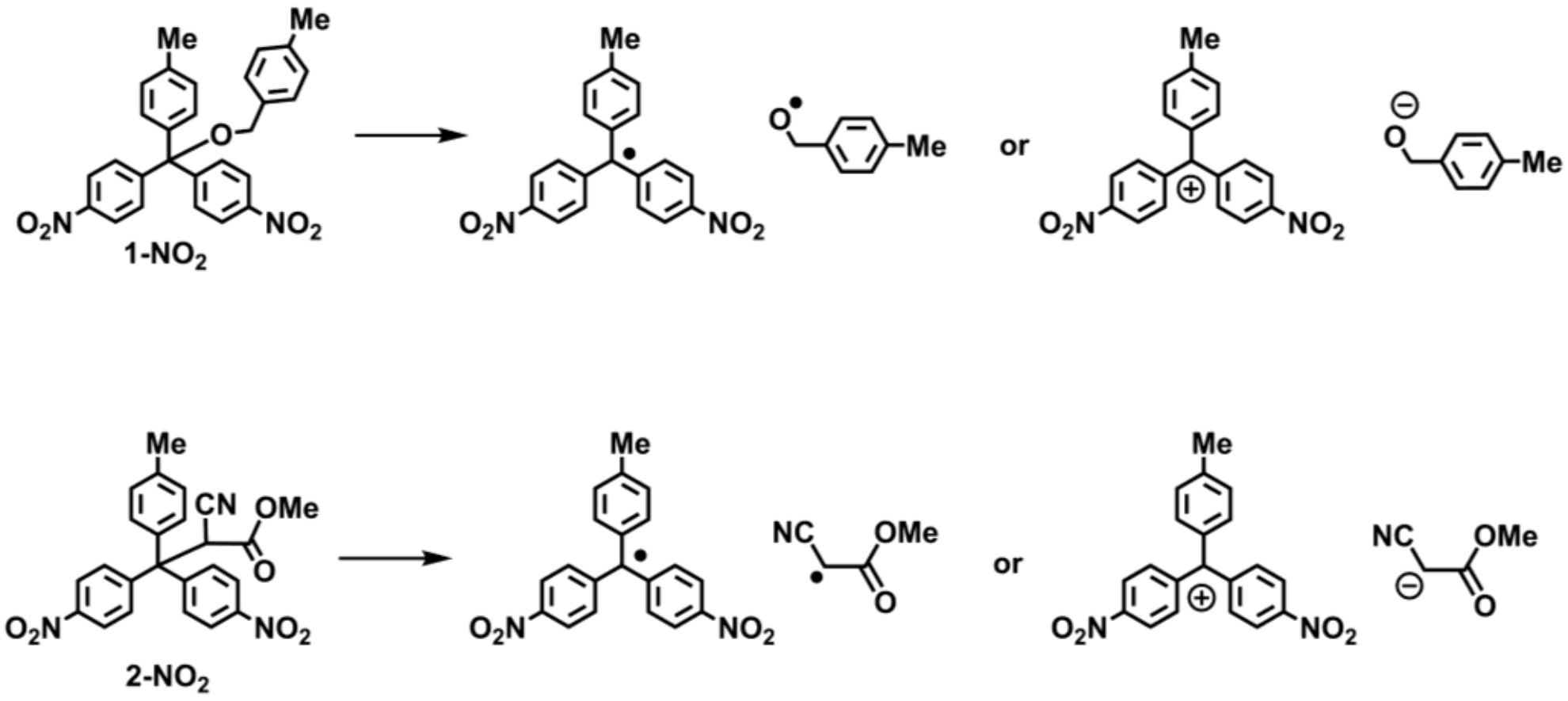

Figure S46. Model compounds **1-NO_2** and **2-NO_2** to assess correct energy trends in mechanochemical cleavage.

Indeed, it was observed that strongly electron-withdrawing groups significantly hinder heterolytic cleavage. This observation suggests that calculations accurately reflect the stability of the computed species and that the computational method used was not marred by artifacts. Consequently, the energies obtained for the experimentally studied systems can be relied upon to not only mirror the environmental trends affecting cleavage (through solvation) but also to accurately respond to changes in the electronics of the parent triaryl system.

Table S4. Calculated free energy (kcal/mol) for the homolysis and heterolysis of the C–O bond of **1-NO_2** (**ΔG_{hom}** and ΔG_{het}, respectively), the difference between the calculated free energy for the homolysis and heterolysis of the C–O bond of **1-NO_2** ($\Delta\Delta G$), and the calculated free energy for the heterolysis of the C–O bond of **1-NO_2** in the presence of an explicit molecule of methanol (MeOH) (ΔG_{het} MeOH) in ethanol, methyl butanoate, and poly(methyl methacrylate) (PMMA). Calculated free energy (kcal/mol) for the homolysis and heterolysis of the C–C bond of **2-NO_2** (ΔG_{hom} and ΔG_{het}, respectively) and the difference between the calculated free energy for the homolysis and heterolysis of the C–C bond of **2-NO_2** ($\Delta\Delta G$) (B3LYP-D3/6-311+G(d, p), PCM; 1 M, 298 K).

Solvent	ΔG_{hom} **1-NO_2**	ΔG_{het} **1-NO_2**	ΔG_{het} MeOH	$\Delta\Delta G$	ΔG_{hom} **2-NO_2**	ΔG_{het} **2-NO_2**	$\Delta\Delta G$
Ethanol	36.8	53.1	47.0	-16.3	21.8	28.4	-6.6
Methyl butanoate	37.6	68.7	(61.1)[a]	-31.1	21.6	41.1	-19.6
PMMA	37.9	77.4	(69.1)[a]	-39.5	21.9	48.9	-27.0

[a]At low polarity, no hydrogen bonding moieties are expected to be available in the polymer.

The dataset in ioChem-BD with DOI: 10.19061/iochem-bd-1-341, as well as the coordinates for the computational calculations listed below, have the following naming:
compound_(rad/an/cat)_(explicit-solvent)_PCM-solvent
Single-point calculations are preceded by the prefix "SP_" followed by the code of the optimised structure. The following structures are found suffixed according to the implicit solvation used, with ethanol (default, no suffix), methyl propanoate ("_MeProp"), methyl butanoate ("_MeButa") or poly(methyl methacrylate) ("_PMMA").

Table S5. Summary of the investigated compounds and their corresponding codes, together with the calculated fragments obtained from homolytic cleavage and from heterolytic cleavage yielding the respective cationic and anionic species.

Compound	Code	Fragments (homolytic)	Fragments (heterolytic)
**1**	ar3m2obn<br>ar3m2obn_meoh<br>ar3m2obn_m2(b,c)[a]	ar3m2_rad<br>obn_rad	ar3m2_cat<br>obn_an<br>obn_an_meoh<br>obn_an_m2(b,c)[a]
**2**	ar3m2cnoac	(ar3m2_rad)<br>cnoac_rad	(ar3m2_cat)<br>cnoac_an
**1-NO$_2$**[b]	ar3no2obn<br>ar3no2obn_meoh<br>ar3no2obn_m2(b,c)[a]	ar3no2_rad<br>(obn_rad)	ar3no2_cat<br>(obn_an)<br>(obn_an_meoh)<br>(obn_an_m2(b,c)[a])
**2-NO$_2$**[b]	ar3no2cnoac	(ar3no2_rad)<br>(cnoac_rad)	(ar3no2_cat)<br>(cnoac_an)
(other)	meoh		

[a]Using explicit solvation with a methanol dimer to validate the minimal explicit solvation model; additional conformations are shown as m2b and m2c. [b]With *para*-nitro groups instead of morpholine to validate the electronic effect of the substituent.
For example, the single-point energy determination of compound **1** modelled in methyl butanoate can be found under "SP_ar3m2obn_MeButa".

cnoac_an_MeButa
Energy (POTENTIAL) = -360.133081694 Eh

	Atom	X	Y	Z
1	C	-0.8576	0.7620	-0.0073
2	H	-0.7549	1.8410	0.0155
3	C	-2.1365	0.2079	-0.0645
4	N	-3.2254	-0.2387	-0.1130
5	C	0.2937	-0.0542	0.0237
6	O	0.3816	-1.2908	0.0050
7	O	1.4589	0.7241	0.0841
8	C	2.6705	-0.0189	0.1083
9	H	2.7165	-0.6963	0.9694
10	H	3.4758	0.7181	0.1761
11	H	2.7986	-0.6208	-0.7997

obn_an_m2c
Energy (POTENTIAL) = -617.081974800 Eh

	Atom	X	Y	Z
1	O	0.7838	-1.3039	-1.2622
2	C	1.4774	-0.3618	-0.4768
3	H	1.6868	-0.7626	0.5327
4	H	2.4580	-0.1819	-0.9417
5	C	0.7602	0.9735	-0.3166
6	C	1.4708	2.1779	-0.2938
7	C	-0.6334	1.0205	-0.1573
8	C	0.8145	3.3985	-0.1094
9	H	2.5514	2.1675	-0.4280
10	C	-1.2841	2.2405	0.0239
11	H	-1.1939	0.0874	-0.1743
12	C	-0.5749	3.4516	0.0543
13	H	1.3898	4.3223	-0.1025
14	H	-2.3664	2.2572	0.1433
15	C	-1.2921	4.7641	0.2749
16	H	-2.2168	4.8193	-0.3115
17	H	-1.5738	4.8915	1.3288
18	H	-0.6638	5.6173	-0.0018
19	O	-1.1869	-2.1646	0.1558
20	H	-0.0038	-1.6594	-0.6972
21	C	-0.8293	-2.1249	1.4966
22	H	-0.5862	-1.0976	1.8443
23	H	-1.6383	-2.4853	2.1704
24	H	0.0611	-2.7443	1.7470
25	O	-0.7818	-4.6384	-0.5177
26	H	-1.0204	-3.6805	-0.2617
27	C	0.6267	-4.7052	-0.4630
28	H	1.0108	-4.8601	0.5640
29	H	0.9755	-5.5552	-1.0681
30	H	1.1012	-3.7909	-0.8506

SP_ar3m2_cat_PMMA
Energy (POTENTIAL) = -1345.819017 Eh

	Atom	X	Y	Z
1	C	-0.1704	0.0031	1.1717
2	C	-0.1908	-1.2387	0.4569
3	C	0.3530	-1.3707	-0.8536
4	C	-0.7513	-2.4179	1.0253
5	C	0.3369	-2.5628	-1.5362
6	H	0.8580	-0.5220	-1.3024
7	C	-0.7942	-3.6104	0.3432
8	H	-1.2199	-2.3611	2.0018
9	C	-0.2438	-3.7378	-0.9683
10	H	0.8290	-2.6065	-2.4992
11	H	-1.3023	-4.4457	0.8067
12	C	-0.1229	-0.0150	2.6379
13	C	-0.8856	0.8961	3.4007
14	C	0.6819	-0.9472	3.3276
15	C	-0.8502	0.8638	4.7886
16	H	-1.5351	1.6020	2.8930
17	C	0.7275	-0.9560	4.7161
18	H	1.2947	-1.6422	2.7626
19	C	-0.0383	-0.0552	5.4730
20	H	-1.4661	1.5573	5.3550
21	H	1.3691	-1.6703	5.2250
22	C	-0.1974	1.2628	0.4876
23	C	0.3979	2.4302	1.0468
24	C	-0.8365	1.4268	-0.7737
25	C	0.3890	3.6396	0.3953
26	H	0.9265	2.3522	1.9906
27	C	-0.8728	2.6378	-1.4242
28	H	-1.3771	0.5901	-1.2034
29	C	-0.2431	3.7957	-0.8756
30	H	-1.4432	2.7093	-2.3409
31	C	-0.6886	-6.1869	-1.0202
32	C	-0.1452	-5.0364	-3.0982
33	C	0.1973	-7.3400	-1.5039
34	H	-1.7370	-6.3831	-1.2846

35	H	-0.6061	-6.1289	0.0646
36	C	0.7281	-6.2288	-3.4853
37	H	-1.1560	-5.1837	-3.5047
38	H	0.2610	-4.1254	-3.5323
39	H	-0.1997	-8.2876	-1.1303
40	H	1.2179	-7.2037	-1.1114
41	H	0.7197	-6.3590	-4.5707
42	H	1.7647	-6.0543	-3.1550
43	N	-0.2508	-4.9269	-1.6354
44	O	0.2220	-7.4304	-2.9202
45	N	-0.2369	4.9885	-1.5383
46	C	-0.7663	5.1398	-2.9001
47	C	-0.0650	6.2840	-0.8620
48	C	0.0712	6.1591	-3.6790
49	H	-1.8141	5.4692	-2.8569
50	H	-0.7190	4.1892	-3.4309
51	C	0.7507	7.2410	-1.7292
52	H	-1.0603	6.7163	-0.6858
53	H	0.4231	6.1604	0.1023
54	H	-0.3946	6.3487	-4.6497
55	H	1.0810	5.7500	-3.8442
56	O	0.1492	7.4060	-3.0057
57	H	0.7824	8.2286	-1.2616
58	H	1.7806	6.8637	-1.8345
59	H	0.9147	4.4682	0.8516
60	C	0.0283	-0.0545	6.9786
61	H	0.7824	0.6624	7.3292
62	H	-0.9290	0.2390	7.4211
63	H	0.3052	-1.0384	7.3697

ar3m2obn_meoh_MeButa
Energy (POTENTIAL) = -1846.89837103 Eh

	Atom	X	Y	Z
1	C	-0.0761	0.1737	-0.7615
2	C	-1.3082	-0.6171	-0.3109
3	C	-2.5527	-0.3341	-0.9033
4	C	-1.3010	-1.4991	0.7714
5	C	-3.7217	-0.9272	-0.4516
6	H	-2.6109	0.3922	-1.7075
7	C	-2.4696	-2.1163	1.2234
8	H	-0.3697	-1.7268	1.2785
9	C	-3.7102	-1.8466	0.6203
10	H	-4.6632	-0.6497	-0.9146
11	H	-2.3959	-2.8169	2.0468
12	C	1.2672	-0.3809	-0.2558
13	C	2.2578	0.4713	0.2500
14	C	1.5942	-1.7327	-0.4030
15	C	3.5045	-0.0134	0.6294
16	H	2.0584	1.5340	0.3273
17	C	2.8376	-2.2317	-0.0161
18	H	0.8629	-2.4201	-0.8181
19	C	3.8232	-1.3806	0.5172
20	H	4.2530	0.6855	0.9902
21	H	3.0245	-3.2938	-0.1244
22	C	0.0432	0.2723	-2.2917
23	C	0.6526	1.3890	-2.8787
24	C	-0.3501	-0.7809	-3.1242
25	C	0.8363	1.4570	-4.2586
26	H	0.9834	2.2083	-2.2503
27	C	-0.1577	-0.7104	-4.5045
28	H	-0.8178	-1.6620	-2.6962
29	C	0.4316	0.4112	-5.0996
30	H	1.3078	2.3379	-4.6894
31	H	-0.4766	-1.5422	-5.1286
32	N	5.1065	-1.8348	0.8935
33	C	5.5576	-1.4789	2.2482
34	C	5.4633	-3.2152	0.5689
35	C	7.0549	-1.7348	2.3833
36	H	5.0178	-2.0776	3.0030
37	H	5.3540	-0.4252	2.4483
38	C	6.9659	-3.4158	0.7612
39	H	4.9166	-3.9384	1.2008
40	H	5.2121	-3.4181	-0.4769
41	H	7.3805	-1.5535	3.4119
42	H	7.6070	-1.0581	1.7105
43	O	7.3744	-3.0904	2.0857
44	H	7.2308	-4.4648	0.5995
45	H	7.5131	-2.7950	0.0332
46	N	-4.9180	-2.4105	1.0753
47	C	-5.8007	-3.0127	0.0639
48	C	-4.8810	-3.2200	2.2927
49	C	-7.1883	-3.2465	0.6525
50	H	-5.3859	-3.9759	-0.2815
51	H	-5.8864	-2.3552	-0.8028
52	C	-6.3022	-3.4511	2.8038
53	H	-4.3924	-4.1951	2.1179

54	H	-4.3157	-2.6888	3.0645
55	H	-7.8217	-3.7729	-0.0675
56	H	-7.6542	-2.2773	0.8950
57	O	-7.1240	-4.0642	1.8168
58	H	-6.2875	-4.1258	3.6648
59	H	-6.7394	-2.4883	3.1153
60	O	-0.2304	1.5533	-0.3159
61	C	-0.5719	1.7508	1.0744
62	H	-1.6420	1.5552	1.2085
63	H	-0.0174	1.0426	1.6987
64	C	-0.2189	3.1705	1.4276
65	C	0.9285	3.4628	2.1720
66	C	-1.0195	4.2321	0.9832
67	C	1.2699	4.7842	2.4669
68	H	1.5617	2.6512	2.5234
69	C	-0.6723	5.5496	1.2752
70	H	-1.9045	4.0185	0.3897
71	C	0.4758	5.8494	2.0247
72	H	2.1663	4.9896	3.0477
73	H	-1.3029	6.3610	0.9179
74	C	0.8251	7.2791	2.3657
75	H	0.5757	7.9587	1.5434
76	H	0.2679	7.6210	3.2480
77	H	1.8913	7.3902	2.5891
78	C	0.6050	0.5061	-6.5970
79	H	-0.2163	1.0738	-7.0544
80	H	1.5370	1.0184	-6.8605
81	H	0.6169	-0.4845	-7.0636
82	C	-1.8128	3.8820	-2.5559
83	H	-2.6724	4.3971	-2.9967
84	H	-1.2192	4.6213	-1.9958
85	H	-1.1908	3.4828	-3.3698
86	O	-2.3162	2.8543	-1.7136
87	H	-1.5463	2.4065	-1.3053

SP_ar3no2obn_MeButa
Energy (POTENTIAL) = -1567.3583956 Eh

	Atom	X	Y	Z
1	C	-0.3800	-0.2323	-0.3915
2	C	-1.7390	0.1282	0.2331
3	C	-2.6737	0.8058	-0.5674
4	C	-2.0205	-0.0496	1.5936
5	C	-3.8730	1.2647	-0.0382
6	H	-2.4552	0.9739	-1.6160
7	C	-3.2190	0.4008	2.1431
8	H	-1.3030	-0.5384	2.2420
9	C	-4.1326	1.0478	1.3159
10	H	-4.5988	1.7824	-0.6524
11	H	-3.4424	0.2607	3.1932
12	C	0.4414	-1.2678	0.4042
13	C	1.8237	-1.0891	0.5541
14	C	-0.1446	-2.4482	0.8894
15	C	2.6034	-2.0454	1.1969
16	H	2.2915	-0.1960	0.1581
17	C	0.6187	-3.4156	1.5319
18	H	-1.2085	-2.6180	0.7662
19	C	1.9885	-3.1979	1.6827
20	H	3.6704	-1.9083	1.3197
21	H	0.1698	-4.3246	1.9116
22	C	-0.5308	-0.8155	-1.8082
23	C	0.3820	-0.4767	-2.8125
24	C	-1.5284	-1.7548	-2.0987
25	C	0.2882	-1.0579	-4.0773
26	H	1.1628	0.2452	-2.6039
27	C	-1.6143	-2.3328	-3.3637
28	H	-2.2534	-2.0338	-1.3396
29	C	-0.7095	-1.9935	-4.3786
30	H	1.0074	-0.7782	-4.8438
31	H	-2.3994	-3.0577	-3.5654
32	O	0.3663	0.9809	-0.5498
33	C	0.5065	1.8247	0.6103
34	H	-0.4144	2.4060	0.7444
35	H	0.6578	1.2222	1.5139
36	C	1.6907	2.7191	0.3639
37	C	2.8930	2.5342	1.0530
38	C	1.6196	3.7303	-0.6032
39	C	3.9979	3.3458	0.7873
40	H	2.9685	1.7489	1.8019
41	C	2.7245	4.5349	-0.8702
42	H	0.6935	3.8789	-1.1532
43	C	3.9323	4.3593	-0.1764
44	H	4.9238	3.1870	1.3351
45	H	2.6524	5.3134	-1.6264
46	C	5.1152	5.2592	-0.4464
47	H	5.1894	5.5141	-1.5092
48	H	5.0252	6.2033	0.1071

49	H	6.0557	4.7893	-0.1411
50	C	-0.8256	-2.5950	-5.7590
51	H	-1.4821	-1.9892	-6.3976
52	H	0.1491	-2.6508	-6.2549
53	H	-1.2496	-3.6041	-5.7220
54	N	-5.3994	1.5228	1.8854
55	O	-6.1920	2.0966	1.1358
56	O	-5.6069	1.3240	3.0841
57	N	2.8007	-4.2090	2.3648
58	O	2.2361	-5.2235	2.7810
59	O	4.0089	-3.9955	2.4900

SP_ar3m2_cat_MeButa
Energy (POTENTIAL) = -1345.8238166 Eh

	Atom	X	Y	Z
1	C	-0.1700	0.0030	1.1709
2	C	-0.1906	-1.2388	0.4564
3	C	0.3530	-1.3709	-0.8542
4	C	-0.7510	-2.4179	1.0252
5	C	0.3367	-2.5630	-1.5368
6	H	0.8580	-0.5223	-1.3033
7	C	-0.7942	-3.6104	0.3431
8	H	-1.2193	-2.3612	2.0017
9	C	-0.2441	-3.7377	-0.9685
10	H	0.8285	-2.6066	-2.4999
11	H	-1.3021	-4.4458	0.8068
12	C	-0.1226	-0.0150	2.6374
13	C	-0.8855	0.8960	3.4000
14	C	0.6824	-0.9469	3.3272
15	C	-0.8500	0.8640	4.7880
16	H	-1.5351	1.6018	2.8922
17	C	0.7280	-0.9556	4.7157
18	H	1.2951	-1.6420	2.7623
19	C	-0.0379	-0.0547	5.4725
20	H	-1.4659	1.5577	5.3542
21	H	1.3696	-1.6700	5.2246
22	C	-0.1971	1.2626	0.4869
23	C	0.3973	2.4303	1.0465
24	C	-0.8356	1.4263	-0.7748
25	C	0.3877	3.6399	0.3953
26	H	0.9257	2.3527	1.9904
27	C	-0.8728	2.6374	-1.4250
28	H	-1.3752	0.5893	-1.2051
29	C	-0.2446	3.7957	-0.8756
30	H	-1.4423	2.7081	-2.3423
31	C	-0.6895	-6.1867	-1.0207
32	C	-0.1455	-5.0364	-3.0983
33	C	0.1976	-7.3392	-1.5027
34	H	-1.7376	-6.3834	-1.2862
35	H	-0.6084	-6.1287	0.0642
36	C	0.7291	-6.2278	-3.4847
37	H	-1.1560	-5.1845	-3.5051
38	H	0.2603	-4.1254	-3.5327
39	H	-0.1991	-8.2868	-1.1289
40	H	1.2178	-7.2019	-1.1096
41	H	0.7214	-6.3579	-4.5701
42	H	1.7652	-6.0524	-3.1537
43	N	-0.2516	-4.9268	-1.6357
44	O	0.2242	-7.4307	-2.9196
45	N	-0.2402	4.9889	-1.5374
46	C	-0.7694	5.1415	-2.8990
47	C	-0.0664	6.2841	-0.8614
48	C	0.0717	6.1572	-3.6782
49	H	-1.8159	5.4748	-2.8554
50	H	-0.7261	4.1909	-3.4299
51	C	0.7534	7.2382	-1.7276
52	H	-1.0610	6.7188	-0.6874
53	H	0.4194	6.1603	0.1039
54	H	-0.3931	6.3477	-4.6491
55	H	1.0802	5.7447	-3.8426
56	O	0.1543	7.4050	-3.0057
57	H	0.7876	8.2257	-1.2601
58	H	1.7821	6.8574	-1.8315
59	H	0.9126	4.4687	0.8520
60	C	0.0289	-0.0537	6.9782
61	H	0.7831	0.6634	7.3286
62	H	-0.9284	0.2400	7.4206
63	H	0.3060	-1.0375	7.3693

ar3no2obn_MeButa
Energy (POTENTIAL) = -1566.94167074 Eh

	Atom	X	Y	Z
1	C	-0.3800	-0.2323	-0.3915
2	C	-1.7390	0.1282	0.2331
3	C	-2.6737	0.8058	-0.5674
4	C	-2.0205	-0.0497	1.5936

5	C	-3.8730	1.2647	-0.0382
6	H	-2.4552	0.9739	-1.6160
7	C	-3.2190	0.4008	2.1431
8	H	-1.3030	-0.5384	2.2420
9	C	-4.1326	1.0478	1.3159
10	H	-4.5988	1.7824	-0.6524
11	H	-3.4424	0.2607	3.1932
12	C	0.4414	-1.2678	0.4042
13	C	1.8237	-1.0891	0.5541
14	C	-0.1446	-2.4482	0.8894
15	C	2.6034	-2.0454	1.1969
16	H	2.2915	-0.1960	0.1581
17	C	0.6187	-3.4156	1.5319
18	H	-1.2085	-2.6180	0.7662
19	C	1.9885	-3.1979	1.6827
20	H	3.6704	-1.9083	1.3197
21	H	0.1698	-4.3246	1.9116
22	C	-0.5308	-0.8155	-1.8082
23	C	0.3820	-0.4767	-2.8125
24	C	-1.5284	-1.7548	-2.0987
25	C	0.2882	-1.0579	-4.0773
26	H	1.1628	0.2452	-2.6039
27	C	-1.6143	-2.3328	-3.3637
28	H	-2.2534	-2.0338	-1.3396
29	C	-0.7095	-1.9935	-4.3786
30	H	1.0074	-0.7782	-4.8438
31	H	-2.3994	-3.0577	-3.5654
32	O	0.3663	0.9809	-0.5498
33	C	0.5065	1.8247	0.6103
34	H	-0.4144	2.4060	0.7444
35	H	0.6578	1.2222	1.5139
36	C	1.6907	2.7191	0.3639
37	C	2.8930	2.5342	1.0530
38	C	1.6196	3.7303	-0.6032
39	C	3.9979	3.3458	0.7873
40	H	2.9685	1.7489	1.8019
41	C	2.7245	4.5349	-0.8702
42	H	0.6935	3.8789	-1.1532
43	C	3.9323	4.3593	-0.1764
44	H	4.9238	3.1870	1.3351
45	H	2.6524	5.3134	-1.6264
46	C	5.1152	5.2592	-0.4464
47	H	5.1894	5.5141	-1.5092
48	H	5.0252	6.2033	0.1071
49	H	6.0557	4.7893	-0.1411
50	C	-0.8256	-2.5950	-5.7590
51	H	-1.4821	-1.9892	-6.3976
52	H	0.1491	-2.6508	-6.2549
53	H	-1.2496	-3.6041	-5.7220
54	N	-5.3994	1.5228	1.8854
55	O	-6.1920	2.0966	1.1358
56	O	-5.6069	1.3240	3.0841
57	N	2.8007	-4.2090	2.3648
58	O	2.2361	-5.2235	2.7810
59	O	4.0089	-3.9955	2.4900

SP_cnoac_rad
Energy (POTENTIAL) = -360.0952747 Eh

	Atom	X	Y	Z
1	C	-0.8645	0.7919	-0.0050
2	H	-0.7736	1.8720	0.0260
3	C	-2.1274	0.2093	-0.0663
4	N	-3.1947	-0.2738	-0.1173
5	C	0.3361	-0.0593	0.0185
6	O	0.3179	-1.2785	-0.0145
7	O	1.4439	0.6953	0.0817
8	C	2.6910	-0.0326	0.1126
9	H	2.7277	-0.6796	0.9927
10	H	3.4689	0.7281	0.1595
11	H	2.7959	-0.6393	-0.7904

SP_obn_an_PMMA
Energy (POTENTIAL) = -385.6860308 Eh

	Atom	X	Y	Z
1	O	-1.0854	-3.1799	0.2059
2	C	0.0820	-2.5754	0.3626
3	H	0.6062	-2.7347	1.3705
4	H	0.9185	-2.8886	-0.3571
5	C	0.0745	-1.0269	0.2234
6	C	1.2240	-0.2395	0.3809
7	C	-1.1235	-0.3771	-0.0721
8	C	1.1749	1.1477	0.2503
9	H	2.1739	-0.7266	0.6091
10	C	-1.1794	1.0145	-0.2049
11	H	-1.9967	-1.0144	-0.1911
12	C	-0.0339	1.8028	-0.0441

13	H	2.0827	1.7384	0.3768
14	H	-2.1279	1.5001	-0.4368
15	C	-0.0844	3.3099	-0.1674
16	H	-1.0573	3.6486	-0.5412
17	H	0.0849	3.8039	0.7996
18	H	0.6855	3.6868	-0.8534

SP_obn_an
Energy (POTENTIAL) = -385.7107601 Eh

	Atom	X	Y	Z
1	O	-1.0798	-3.1912	0.2112
2	C	0.0882	-2.5735	0.3632
3	H	0.6148	-2.7353	1.3661
4	H	0.9183	-2.8859	-0.3597
5	C	0.0708	-1.0272	0.2254
6	C	1.2238	-0.2417	0.3717
7	C	-1.1286	-0.3744	-0.0587
8	C	1.1758	1.1454	0.2383
9	H	2.1754	-0.7285	0.5910
10	C	-1.1831	1.0174	-0.1944
11	H	-2.0095	-1.0020	-0.1708
12	C	-0.0341	1.8034	-0.0465
13	H	2.0860	1.7333	0.3542
14	H	-2.1323	1.5032	-0.4197
15	C	-0.0837	3.3103	-0.1684
16	H	-1.0260	3.6456	-0.6154
17	H	0.0057	3.7987	0.8117
18	H	0.7371	3.6920	-0.7882

SP_cnoac_an
Energy (POTENTIAL) = -360.2753495 Eh

	Atom	X	Y	Z
1	C	-0.8579	0.7699	-0.0079
2	H	-0.7583	1.8491	0.0146
3	C	-2.1325	0.2088	-0.0654
4	N	-3.2150	-0.2537	-0.1141
5	C	0.2936	-0.0482	0.0237
6	O	0.3703	-1.2868	0.0051
7	O	1.4575	0.7214	0.0842
8	C	2.6720	-0.0224	0.1091
9	H	2.7191	-0.6938	0.9742
10	H	3.4757	0.7157	0.1716
11	H	2.7967	-0.6265	-0.7974

ar3no2_rad
Energy (POTENTIAL) = -1181.41380139 Eh

	Atom	X	Y	Z
1	C	-0.0006	-0.0017	0.4630
2	C	-0.0021	-1.2656	-0.2600
3	C	0.6525	-1.4019	-1.5121
4	C	-0.6509	-2.4105	0.2718
5	C	0.6614	-2.6063	-2.1950
6	H	1.1843	-0.5553	-1.9313
7	C	-0.6572	-3.6171	-0.4072
8	H	-1.1776	-2.3329	1.2159
9	C	0.0023	-3.7074	-1.6378
10	H	1.1779	-2.7089	-3.1409
11	H	-1.1711	-4.4817	-0.0066
12	C	-0.0016	0.0010	1.9259
13	C	-0.7121	0.9809	2.6584
14	C	0.7044	-0.9769	2.6634
15	C	-0.7163	0.9752	4.0471
16	H	-1.2875	1.7311	2.1252
17	C	0.7030	-0.9672	4.0533
18	H	1.2763	-1.7332	2.1352
19	C	-0.0053	0.0063	4.7743
20	H	-1.2882	1.7301	4.5810
21	H	1.2669	-1.7251	4.5913
22	C	0.0021	1.2603	-0.2635
23	C	0.6491	2.4069	0.2669
24	C	-0.6499	1.3932	-1.5173
25	C	0.6560	3.6119	-0.4150
26	H	1.1738	2.3319	1.2124
27	C	-0.6580	2.5960	-2.2032
28	H	-1.1803	0.5453	-1.9356
29	C	-0.0008	3.6988	-1.6471
30	H	-1.1726	2.6959	-3.1504
31	H	1.1685	4.4777	-0.0152
32	C	0.0147	0.0314	6.2819
33	H	0.7593	0.7506	6.6485
34	H	-0.9554	0.3364	6.6890
35	H	0.2718	-0.9480	6.6972
36	N	-0.0011	4.9641	-2.3659
37	O	-0.5705	5.0151	-3.4628
38	O	0.5682	5.9325	-1.8481
39	N	0.0033	-4.9744	-2.3535

40	O	-0.5676	-5.9412	-1.8346
41	O	0.5749	-5.0283	-3.4492

SP_obn_an_m2c
Energy (POTENTIAL) = -617.2980515 Eh

	Atom	X	Y	Z
1	O	0.7838	-1.3039	-1.2622
2	C	1.4774	-0.3618	-0.4768
3	H	1.6868	-0.7626	0.5327
4	H	2.4580	-0.1819	-0.9417
5	C	0.7602	0.9735	-0.3166
6	C	1.4708	2.1779	-0.2938
7	C	-0.6334	1.0205	-0.1573
8	C	0.8145	3.3985	-0.1094
9	H	2.5514	2.1675	-0.4280
10	C	-1.2841	2.2405	0.0239
11	H	-1.1939	0.0874	-0.1743
12	C	-0.5749	3.4516	0.0543
13	H	1.3898	4.3223	-0.1025
14	H	-2.3664	2.2572	0.1433
15	C	-1.2921	4.7641	0.2749
16	H	-2.2168	4.8193	-0.3115
17	H	-1.5738	4.8915	1.3288
18	H	-0.6638	5.6173	-0.0018
19	O	-1.1869	-2.1646	0.1558
20	H	-0.0038	-1.6594	-0.6972
21	C	-0.8293	-2.1249	1.4966
22	H	-0.5862	-1.0976	1.8443
23	H	-1.6383	-2.4853	2.1704
24	H	0.0611	-2.7443	1.7470
25	O	-0.7818	-4.6384	-0.5177
26	H	-1.0204	-3.6805	-0.2617
27	C	0.6267	-4.7052	-0.4630
28	H	1.0108	-4.8601	0.5640
29	H	0.9755	-5.5552	-1.0681
30	H	1.1012	-3.7909	-0.8506

obn_an_m2b_MeButa
Energy (POTENTIAL) = -617.070282199 Eh

	Atom	X	Y	Z
1	O	0.0808	-1.4553	-1.0784
2	C	1.1150	-0.7176	-0.5558
3	H	1.5066	-1.1163	0.4126
4	H	2.0133	-0.7199	-1.2205
5	C	0.7829	0.7562	-0.3005
6	C	1.7376	1.6328	0.2354
7	C	-0.4787	1.2707	-0.6063
8	C	1.4396	2.9760	0.4592
9	H	2.7309	1.2555	0.4787
10	C	-0.7794	2.6175	-0.3805
11	H	-1.2210	0.5901	-1.0095
12	C	0.1704	3.4944	0.1559
13	H	2.1999	3.6359	0.8750
14	H	-1.7714	2.9940	-0.6264
15	C	-0.1624	4.9437	0.4307
16	H	-1.0093	5.2828	-0.1761
17	H	-0.4337	5.1015	1.4839
18	H	0.6897	5.6003	0.2182
19	O	-2.0446	-1.6957	0.3971
20	H	-1.1850	-1.5264	-0.1258
21	C	-2.6865	-2.7510	-0.2843
22	H	-1.9936	-3.5735	-0.5191
23	H	-3.4915	-3.1508	0.3494
24	H	-3.1516	-2.4289	-1.2357
25	O	0.5636	-3.9489	-0.5075
26	H	0.4010	-2.9893	-0.8107
27	C	0.5496	-3.9049	0.9027
28	H	-0.2808	-3.2968	1.2908
29	H	0.4376	-4.9270	1.2933
30	H	1.4861	-3.4951	1.3284

cnoac_rad_PMMA
Energy (POTENTIAL) = -359.983290569 Eh

	Atom	X	Y	Z
1	C	-0.8648	0.7856	-0.0043
2	H	-0.7705	1.8657	0.0263
3	C	-2.1298	0.2090	-0.0663
4	N	-3.2024	-0.2625	-0.1181
5	C	0.3367	-0.0638	0.0196
6	O	0.3249	-1.2816	-0.0128
7	O	1.4441	0.6967	0.0823
8	C	2.6904	-0.0300	0.1120
9	H	2.7279	-0.6805	0.9897
10	H	3.4684	0.7308	0.1609
11	H	2.7964	-0.6360	-0.7916

SP_obn_an_m1_MeButa
Energy (POTENTIAL) = -501.4920305 Eh

	Atom	X	Y	Z
1	O	0.9634	-2.3772	-1.4221
2	C	1.6329	-1.4277	-0.7251
3	H	2.0021	-1.7575	0.2886
4	H	2.5800	-1.1013	-1.2351
5	C	0.8648	-0.1207	-0.4422
6	C	1.5015	0.9966	0.1175
7	C	-0.4970	-0.0171	-0.7360
8	C	0.8024	2.1744	0.3776
9	H	2.5661	0.9423	0.3481
10	C	-1.2012	1.1619	-0.4703
11	H	-0.9931	-0.8892	-1.1500
12	C	-0.5681	2.2781	0.0889
13	H	1.3230	3.0297	0.8073
14	H	-2.2641	1.2170	-0.7035
15	C	-1.3319	3.5459	0.4008
16	H	-2.2717	3.5962	-0.1601
17	H	-1.5856	3.6124	1.4680
18	H	-0.7457	4.4403	0.1567
19	O	-1.0796	-3.2862	-0.2329
20	H	-0.2177	-2.9169	-0.7108
21	C	-0.9379	-2.9985	1.1304
22	H	-0.7889	-1.9217	1.3335
23	H	-1.8485	-3.3067	1.6689
24	H	-0.0905	-3.5316	1.6073

cnoac_rad_MeProp
Energy (POTENTIAL) = -359.984395548 Eh

	Atom	X	Y	Z
1	C	-0.8647	0.7879	-0.0046
2	H	-0.7717	1.8680	0.0260
3	C	-2.1290	0.2091	-0.0663
4	N	-3.1997	-0.2665	-0.1178
5	C	0.3365	-0.0622	0.0193
6	O	0.3222	-1.2806	-0.0132
7	O	1.4440	0.6961	0.0821
8	C	2.6907	-0.0309	0.1122
9	H	2.7278	-0.6803	0.9907
10	H	3.4686	0.7299	0.1606
11	H	2.7964	-0.6370	-0.7913

SP_ar3no2_rad
Energy (POTENTIAL) = -1181.730777 Eh

	Atom	X	Y	Z
1	C	-0.0006	-0.0017	0.4630
2	C	-0.0021	-1.2656	-0.2600
3	C	0.6525	-1.4019	-1.5121
4	C	-0.6509	-2.4105	0.2718
5	C	0.6614	-2.6063	-2.1950
6	H	1.1843	-0.5553	-1.9313
7	C	-0.6572	-3.6171	-0.4072
8	H	-1.1776	-2.3329	1.2159
9	C	0.0023	-3.7074	-1.6378
10	H	1.1779	-2.7089	-3.1409
11	H	-1.1711	-4.4817	-0.0066
12	C	-0.0016	0.0010	1.9259
13	C	-0.7121	0.9809	2.6584
14	C	0.7044	-0.9769	2.6634
15	C	-0.7163	0.9752	4.0471
16	H	-1.2875	1.7311	2.1252
17	C	0.7030	-0.9672	4.0533
18	H	1.2763	-1.7332	2.1352
19	C	-0.0053	0.0063	4.7743
20	H	-1.2882	1.7301	4.5810
21	H	1.2669	-1.7251	4.5913
22	C	0.0021	1.2603	-0.2635
23	C	0.6491	2.4069	0.2669
24	C	-0.6499	1.3932	-1.5173
25	C	0.6560	3.6119	-0.4150
26	H	1.1738	2.3319	1.2124
27	C	-0.6580	2.5960	-2.2032
28	H	-1.1803	0.5453	-1.9356
29	C	-0.0008	3.6988	-1.6471
30	H	-1.1726	2.6959	-3.1504
31	H	1.1685	4.4777	-0.0152
32	C	0.0147	0.0314	6.2819
33	H	0.7593	0.7506	6.6485
34	H	-0.9554	0.3364	6.6890
35	H	0.2718	-0.9480	6.6972
36	N	-0.0011	4.9641	-2.3659
37	O	-0.5705	5.0150	-3.4628
38	O	0.5682	5.9325	-1.8481
39	N	0.0033	-4.9744	-2.3535
40	O	-0.5676	-5.9412	-1.8346

41	O	0.5749	-5.0283	-3.4492

ar3m2obn_PMMA
Energy (POTENTIAL) = -1731.15396608 Eh

	Atom	X	Y	Z
1	C	-0.1406	0.2982	-0.9006
2	C	-1.4398	-0.3576	-0.4148
3	C	-2.6237	-0.1391	-1.1397
4	C	-1.5542	-1.0260	0.8059
5	C	-3.8516	-0.5945	-0.6838
6	H	-2.5798	0.4143	-2.0727
7	C	-2.7823	-1.5044	1.2694
8	H	-0.6725	-1.1927	1.4155
9	C	-3.9615	-1.3020	0.5329
10	H	-4.7439	-0.3758	-1.2621
11	H	-2.8032	-2.0455	2.2083
12	C	1.1465	-0.2824	-0.2809
13	C	2.2079	0.5667	0.0625
14	C	1.3595	-1.6587	-0.1569
15	C	3.4152	0.0649	0.5335
16	H	2.0887	1.6378	-0.0590
17	C	2.5635	-2.1736	0.3253
18	H	0.5700	-2.3537	-0.4263
19	C	3.6219	-1.3203	0.6844
20	H	4.2213	0.7567	0.7582
21	H	2.6615	-3.2481	0.4288
22	C	0.0198	0.1887	-2.4279
23	C	0.4906	1.2672	-3.1828
24	C	-0.2303	-1.0227	-3.0863
25	C	0.6972	1.1364	-4.5577
26	H	0.6947	2.2114	-2.6920
27	C	-0.0207	-1.1482	-4.4576
28	H	-0.6008	-1.8742	-2.5232
29	C	0.4453	-0.0691	-5.2219
30	H	1.0631	1.9912	-5.1228
31	H	-0.2245	-2.1003	-4.9433
32	N	4.8711	-1.7979	1.1403
33	C	5.3735	-1.2314	2.4013
34	C	5.1132	-3.2384	1.0816
35	C	6.8493	-1.5750	2.5750
36	H	4.8042	-1.6330	3.2584
37	H	5.2552	-0.1460	2.3999
38	C	6.5992	-3.5187	1.3011
39	H	4.5251	-3.7840	1.8417
40	H	4.8262	-3.6142	0.0944
41	H	7.2089	-1.2302	3.5490
42	H	7.4375	-1.0825	1.7831
43	O	7.0584	-2.9831	2.5372
44	H	6.7788	-4.5971	1.3404
45	H	7.1781	-3.0925	0.4654
46	N	-5.2276	-1.7313	0.9818
47	C	-6.0477	-2.4967	0.0299
48	C	-5.3209	-2.2958	2.3273
49	C	-7.4873	-2.5768	0.5273
50	H	-5.6469	-3.5190	-0.0864
51	H	-6.0333	-2.0178	-0.9509
52	C	-6.7872	-2.3891	2.7477
53	H	-4.8609	-3.2989	2.3811
54	H	-4.7965	-1.6423	3.0314
55	H	-8.0822	-3.2153	-0.1325
56	H	-7.9299	-1.5671	0.5397
57	O	-7.5524	-3.1568	1.8259
58	H	-6.8703	-2.8882	3.7176
59	H	-7.2070	-1.3734	2.8343
60	O	-0.2160	1.7144	-0.6327
61	C	-0.5333	2.0932	0.7129
62	H	-1.6132	1.9824	0.8788
63	H	-0.0187	1.4446	1.4324
64	C	-0.0981	3.5239	0.8884
65	C	1.0241	3.8511	1.6550
66	C	-0.7918	4.5566	0.2441
67	C	1.4384	5.1787	1.7835
68	H	1.5825	3.0606	2.1513
69	C	-0.3743	5.8795	0.3689
70	H	-1.6599	4.3156	-0.3647
71	C	0.7464	6.2145	1.1449
72	H	2.3133	5.4119	2.3861
73	H	-0.9246	6.6675	-0.1409
74	C	1.1730	7.6553	1.3032
75	H	1.0540	8.2128	0.3674
76	H	0.5657	8.1658	2.0625
77	H	2.2198	7.7325	1.6148
78	C	0.6406	-0.2011	-6.7143
79	H	-0.3080	-0.0692	-7.2521
80	H	1.3391	0.5513	-7.0954
81	H	1.0274	-1.1909	-6.9818

ar3no2_rad_PMMA
Energy (POTENTIAL) = -1181.40937234 Eh

	Atom	X	Y	Z
1	C	-0.0033	-0.0021	0.4763
2	C	-0.0065	-1.2649	-0.2499
3	C	0.6482	-1.3996	-1.5018
4	C	-0.6575	-2.4100	0.2781
5	C	0.6552	-2.6027	-2.1881
6	H	1.1808	-0.5520	-1.9184
7	C	-0.6652	-3.6153	-0.4042
8	H	-1.1840	-2.3333	1.2227
9	C	-0.0057	-3.7034	-1.6340
10	H	1.1711	-2.7059	-3.1344
11	H	-1.1799	-4.4814	-0.0078
12	C	-0.0025	-0.0027	1.9394
13	C	-0.7095	0.9771	2.6754
14	C	0.7020	-0.9834	2.6744
15	C	-0.7114	0.9687	4.0639
16	H	-1.2836	1.7299	2.1443
17	C	0.7023	-0.9767	4.0642
18	H	1.2708	-1.7402	2.1435
19	C	-0.0021	-0.0031	4.7883
20	H	-1.2807	1.7242	4.6002
21	H	1.2649	-1.7373	4.5999
22	C	-0.0003	1.2618	-0.2479
23	C	0.6497	2.4064	0.2823
24	C	-0.6547	1.3984	-1.4998
25	C	0.6568	3.6130	-0.3978
26	H	1.1759	2.3284	1.2269
27	C	-0.6623	2.6028	-2.1839
28	H	-1.1866	0.5513	-1.9182
29	C	-0.0024	3.7029	-1.6276
30	H	-1.1780	2.7074	-3.1302
31	H	1.1707	4.4788	0.0005
32	C	0.0205	0.0196	6.2960
33	H	0.7621	0.7415	6.6634
34	H	-0.9504	0.3185	6.7058
35	H	0.2824	-0.9593	6.7097
36	N	-0.0025	4.9726	-2.3457
37	O	-0.5764	5.0231	-3.4386
38	O	0.5716	5.9353	-1.8259
39	N	-0.0063	-4.9717	-2.3546
40	O	-0.5807	-5.9351	-1.8364
41	O	0.5674	-5.0204	-3.4477

ar3no2_cat_PMMA
Energy (POTENTIAL) = -1181.21602753 Eh

	Atom	X	Y	Z
1	C	-0.0050	-0.0028	0.5220
2	C	0.0346	-1.2525	-0.2306
3	C	0.7786	-1.3376	-1.4313
4	C	-0.6835	-2.3856	0.2197
5	C	0.8273	-2.5258	-2.1462
6	H	1.3510	-0.4837	-1.7752
7	C	-0.6614	-3.5681	-0.5073
8	H	-1.2945	-2.3173	1.1124
9	C	0.1008	-3.6177	-1.6729
10	H	1.4117	-2.6158	-3.0528
11	H	-1.2244	-4.4361	-0.1894
12	C	-0.0058	-0.0073	1.9473
13	C	-0.5933	1.0644	2.6870
14	C	0.5794	-1.0844	2.6806
15	C	-0.6038	1.0430	4.0660
16	H	-1.0943	1.8675	2.1591
17	C	0.5852	-1.0742	4.0601
18	H	1.0781	-1.8859	2.1480
19	C	-0.0085	-0.0175	4.7824
20	H	-1.0877	1.8472	4.6114
21	H	1.0619	-1.8860	4.6006
22	C	-0.0437	1.2519	-0.2223
23	C	0.6727	2.3824	0.2372
24	C	-0.7852	1.3450	-1.4239
25	C	0.6512	3.5699	-0.4814
26	H	1.2820	2.3082	1.1307
27	C	-0.8333	2.5381	-2.1306
28	H	-1.3561	0.4931	-1.7751
29	C	-0.1087	3.6273	-1.6481
30	H	-1.4158	2.6340	-3.0378
31	H	1.2130	4.4361	-0.1563
32	C	0.0162	-0.0040	6.2811
33	H	0.8896	0.5658	6.6283
34	H	-0.8723	0.4846	6.6921
35	H	0.0969	-1.0128	6.6949
36	N	-0.1437	4.8962	-2.4108
37	O	-0.8257	4.9242	-3.4319

38	O	0.5106	5.8388	-1.9722
39	N	0.1364	-4.8812	-2.4445
40	O	0.8210	-4.9028	-3.4640
41	O	-0.5202	-5.8261	-2.0143

meoh
Energy (POTENTIAL) = -115.720317782 Eh

	Atom	X	Y	Z
1	C	0.0184	0.0015	-0.6649
2	H	-0.9920	0.0068	-1.0841
3	H	0.5486	0.8915	-1.0355
4	H	0.5403	-0.8929	-1.0370
5	O	-0.1206	0.0010	0.7532
6	H	0.7721	0.0008	1.1315

ar3m2obn_B
Energy (POTENTIAL) = -1731.15666864 Eh

	Atom	X	Y	Z
1	C	-0.6061	-0.7007	-0.3551
2	C	-1.8660	0.1605	-0.1408
3	C	-2.3444	0.4687	1.1424
4	C	-3.5283	1.1691	1.3334
5	C	-4.3204	1.5895	0.2406
6	C	-3.8457	1.2649	-1.0456
7	C	-2.6483	0.5701	-1.2223
8	H	-2.3297	0.3247	-2.2296
9	H	-4.4120	1.5330	-1.9294
10	N	-5.4841	2.3501	0.4597
11	C	-6.2682	2.8280	-0.6841
12	C	-7.2845	1.7782	-1.1432
13	H	-7.9257	2.1715	-1.9380
14	H	-6.7644	0.8829	-1.5195
15	O	-8.1496	1.4218	-0.0627
16	C	-7.4125	0.9291	1.0568
17	C	-6.3965	1.9644	1.5491
18	H	-5.8449	1.5710	2.4032
19	H	-6.9284	2.8641	1.8818
20	H	-8.1468	0.7007	1.8356
21	H	-6.8850	-0.0004	0.7846
22	H	-5.6100	3.1377	-1.4986
23	H	-6.8176	3.7182	-0.3530
24	H	-3.8250	1.4147	2.3475
25	H	-1.7726	0.1723	2.0164
26	C	0.5775	-0.3031	0.5340
27	C	1.6474	-1.1972	0.7043
28	C	2.8143	-0.8289	1.3545
29	C	2.9853	0.4764	1.8696
30	C	1.9076	1.3690	1.7135
31	C	0.7422	0.9838	1.0504
32	H	-0.0491	1.7176	0.9388
33	H	1.9613	2.3766	2.1079
34	N	4.2135	0.8519	2.4372
35	C	4.4417	2.2418	2.8409
36	C	3.9881	2.4873	4.2825
37	H	4.2449	3.4992	4.6106
38	H	2.8976	2.3546	4.3662
39	O	4.6591	1.5900	5.1701
40	C	4.4246	0.2275	4.8139
41	C	4.8830	-0.0577	3.3799
42	H	4.6843	-1.0991	3.1244
43	H	5.9652	0.1061	3.3055
44	H	4.9839	-0.3814	5.5311
45	H	3.3518	-0.0103	4.9089
46	H	3.9577	2.9269	2.1413
47	H	5.5222	2.4253	2.7832
48	H	3.6265	-1.5459	1.4143
49	H	1.5759	-2.1970	0.2877
50	C	-1.0292	-2.1650	-0.1336
51	C	-1.1438	-2.6870	1.1623
52	C	-1.5921	-3.9891	1.3741
53	C	-1.9449	-4.8195	0.3006
54	C	-1.8247	-4.2950	-0.9914
55	C	-1.3786	-2.9893	-1.2084
56	H	-1.2930	-2.6099	-2.2196
57	H	-2.0816	-4.9168	-1.8465
58	C	-2.4584	-6.2208	0.5363
59	H	-1.9559	-6.6940	1.3872
60	H	-3.5338	-6.2153	0.7591
61	H	-2.3108	-6.8556	-0.3437
62	H	-1.6628	-4.3697	2.3909
63	H	-0.8631	-2.0759	2.0146
64	O	-0.2059	-0.6361	-1.7460
65	C	0.4331	0.5643	-2.1925
66	H	0.0957	1.4331	-1.6162
67	H	0.0776	0.7039	-3.2212
68	C	1.9445	0.4655	-2.1776

69	C	2.5797	-0.6901	-2.6479
70	C	3.9699	-0.7845	-2.6582
71	C	4.7707	0.2722	-2.1978
72	C	4.1296	1.4298	-1.7399
73	C	2.7366	1.5244	-1.7277
74	H	2.2629	2.4243	-1.3442
75	H	4.7260	2.2624	-1.3729
76	C	6.2761	0.1479	-2.1580
77	H	6.6475	-0.5021	-2.9578
78	H	6.7631	1.1240	-2.2572
79	H	6.6098	-0.2861	-1.2058
80	H	4.4441	-1.6934	-3.0231
81	H	1.9734	-1.5262	-2.9861

cnoac_an_MeProp
Energy (POTENTIAL) = -360.134547894 Eh

	Atom	X	Y	Z
1	C	-0.8577	0.7628	-0.0073
2	H	-0.7552	1.8418	0.0154
3	C	-2.1361	0.2080	-0.0646
4	N	-3.2244	-0.2402	-0.1131
5	C	0.2937	-0.0536	0.0238
6	O	0.3804	-1.2904	0.0050
7	O	1.4587	0.7238	0.0841
8	C	2.6706	-0.0192	0.1084
9	H	2.7168	-0.6960	0.9699
10	H	3.4758	0.7178	0.1756
11	H	2.7984	-0.6214	-0.7995

obn_rad_MeProp
Energy (POTENTIAL) = -385.438398931 Eh

	Atom	X	Y	Z
1	O	0.0688	-2.9243	-0.8991
2	C	0.1192	-2.5520	0.4300
3	H	-0.7457	-2.9124	1.0100
4	H	1.0528	-2.8609	0.9273
5	C	0.0696	-1.0300	0.2991
6	C	1.2510	-0.2973	0.1273
7	C	-1.1623	-0.3656	0.2426
8	C	1.2008	1.0851	-0.0337
9	H	2.2084	-0.8116	0.1324
10	C	-1.2054	1.0170	0.0814
11	H	-2.0844	-0.9329	0.3375
12	C	-0.0263	1.7659	-0.0556
13	H	2.1252	1.6457	-0.1488
14	H	-2.1667	1.5245	0.0564
15	C	-0.0759	3.2675	-0.1995
16	H	-0.9945	3.5922	-0.6993
17	H	-0.0507	3.7544	0.7845
18	H	0.7788	3.6442	-0.7709

SP_cnoac_rad_MeButa
Energy (POTENTIAL) = -360.0930438 Eh

	Atom	X	Y	Z
1	C	-0.8647	0.7875	-0.0045
2	H	-0.7715	1.8676	0.0261
3	C	-2.1291	0.2090	-0.0663
4	N	-3.2002	-0.2658	-0.1178
5	C	0.3365	-0.0625	0.0193
6	O	0.3227	-1.2807	-0.0132
7	O	1.4440	0.6962	0.0821
8	C	2.6906	-0.0308	0.1121
9	H	2.7278	-0.6804	0.9905
10	H	3.4685	0.7301	0.1607
11	H	2.7964	-0.6368	-0.7914

obn_an_m1_MeButa
Energy (POTENTIAL) = -501.317082876 Eh

	Atom	X	Y	Z
1	O	0.9634	-2.3772	-1.4221
2	C	1.6329	-1.4277	-0.7251
3	H	2.0021	-1.7575	0.2886
4	H	2.5800	-1.1013	-1.2351
5	C	0.8648	-0.1207	-0.4422
6	C	1.5015	0.9966	0.1175
7	C	-0.4970	-0.0171	-0.7360
8	C	0.8024	2.1744	0.3776
9	H	2.5661	0.9423	0.3481
10	C	-1.2012	1.1619	-0.4703
11	H	-0.9931	-0.8892	-1.1500
12	C	-0.5681	2.2781	0.0889
13	H	1.3230	3.0297	0.8073
14	H	-2.2641	1.2170	-0.7035
15	C	-1.3319	3.5459	0.4008
16	H	-2.2717	3.5962	-0.1601

17	H	-1.5856	3.6124	1.4680
18	H	-0.7457	4.4403	0.1567
19	O	-1.0797	-3.2862	-0.2329
20	H	-0.2177	-2.9169	-0.7108
21	C	-0.9379	-2.9985	1.1304
22	H	-0.7889	-1.9217	1.3335
23	H	-1.8485	-3.3067	1.6689
24	H	-0.0905	-3.5316	1.6073

SP_obn_an_MeProp
Energy (POTENTIAL) = -385.6961906 Eh

	Atom	X	Y	Z
1	O	-1.0858	-3.1833	0.2227
2	C	0.0839	-2.5746	0.3642
3	H	0.6195	-2.7351	1.3645
4	H	0.9097	-2.8894	-0.3654
5	C	0.0732	-1.0270	0.2246
6	C	1.2252	-0.2408	0.3718
7	C	-1.1261	-0.3753	-0.0622
8	C	1.1769	1.1463	0.2390
9	H	2.1765	-0.7284	0.5923
10	C	-1.1811	1.0162	-0.1975
11	H	-2.0029	-1.0087	-0.1746
12	C	-0.0331	1.8030	-0.0469
13	H	2.0866	1.7354	0.3563
14	H	-2.1304	1.5021	-0.4242
15	C	-0.0845	3.3102	-0.1666
16	H	-1.0283	3.6449	-0.6118
17	H	0.0055	3.7994	0.8134
18	H	0.7339	3.6947	-0.7885

SP_ar3m2obn
Energy (POTENTIAL) = -1731.6085155 Eh

	Atom	X	Y	Z
1	C	-0.1344	0.2964	-0.8974
2	C	-1.4346	-0.3587	-0.4141
3	C	-2.6184	-0.1364	-1.1383
4	C	-1.5509	-1.0305	0.8047
5	C	-3.8477	-0.5906	-0.6844
6	H	-2.5749	0.4183	-2.0707
7	C	-2.7798	-1.5079	1.2668
8	H	-0.6703	-1.2011	1.4148
9	C	-3.9600	-1.3008	0.5314
10	H	-4.7377	-0.3692	-1.2647
11	H	-2.8007	-2.0515	2.2040
12	C	1.1517	-0.2857	-0.2770
13	C	2.2134	0.5614	0.0708
14	C	1.3647	-1.6629	-0.1571
15	C	3.4202	0.0582	0.5421
16	H	2.0952	1.6331	-0.0450
17	C	2.5683	-2.1798	0.3242
18	H	0.5756	-2.3575	-0.4286
19	C	3.6274	-1.3281	0.6882
20	H	4.2237	0.7509	0.7730
21	H	2.6646	-3.2547	0.4241
22	C	0.0290	0.1873	-2.4247
23	C	0.5071	1.2633	-3.1792
24	C	-0.2243	-1.0232	-3.0845
25	C	0.7170	1.1320	-4.5540
26	H	0.7151	2.2070	-2.6889
27	C	-0.0112	-1.1498	-4.4554
28	H	-0.5996	-1.8737	-2.5230
29	C	0.4614	-0.0726	-5.2192
30	H	1.0880	1.9854	-5.1179
31	H	-0.2179	-2.1010	-4.9413
32	N	4.8755	-1.8084	1.1412
33	C	5.3913	-1.2314	2.3929
34	C	5.1087	-3.2518	1.0958
35	C	6.8656	-1.5827	2.5604
36	H	4.8248	-1.6204	3.2573
37	H	5.2821	-0.1454	2.3820
38	C	6.5940	-3.5396	1.3091
39	H	4.5205	-3.7850	1.8641
40	H	4.8154	-3.6373	0.1145
41	H	7.2332	-1.2282	3.5278
42	H	7.4522	-1.1042	1.7593
43	O	7.0653	-2.9938	2.5374
44	H	6.7656	-4.6189	1.3575
45	H	7.1711	-3.1261	0.4661
46	N	-5.2254	-1.7261	0.9823
47	C	-6.0652	-2.4613	0.0228
48	C	-5.3133	-2.3166	2.3180
49	C	-7.5004	-2.5352	0.5328
50	H	-5.6772	-3.4849	-0.1192
51	H	-6.0578	-1.9624	-0.9477
52	C	-6.7761	-2.4035	2.7508

53	H	-4.8616	-3.3240	2.3466
54	H	-4.7779	-1.6831	3.0316
55	H	-8.1085	-3.1514	-0.1358
56	H	-7.9309	-1.5213	0.5726
57	O	-7.5598	-3.1435	1.8200
58	H	-6.8533	-2.9220	3.7108
59	H	-7.1850	-1.3863	2.8634
60	O	-0.2091	1.7137	-0.6291
61	C	-0.5324	2.0921	0.7167
62	H	-1.6116	1.9740	0.8802
63	H	-0.0141	1.4478	1.4370
64	C	-0.1085	3.5264	0.8903
65	C	1.0370	3.8596	1.6199
66	C	-0.8352	4.5571	0.2795
67	C	1.4425	5.1905	1.7435
68	H	1.6194	3.0719	2.0924
69	C	-0.4269	5.8838	0.3996
70	H	-1.7241	4.3132	-0.2978
71	C	0.7179	6.2243	1.1375
72	H	2.3354	5.4279	2.3173
73	H	-1.0035	6.6699	-0.0831
74	C	1.1359	7.6680	1.2917
75	H	0.9824	8.2300	0.3637
76	H	0.5469	8.1676	2.0723
77	H	2.1908	7.7529	1.5723
78	C	0.6599	-0.2058	-6.7110
79	H	-0.2883	-0.0763	-7.2497
80	H	1.3574	0.5476	-7.0916
81	H	1.0477	-1.1956	-6.9764

SP_ar3m2_rad_MeButa
Energy (POTENTIAL) = -1345.9697248 Eh

	Atom	X	Y	Z
1	C	-0.0912	-0.0165	1.2406
2	C	-0.0197	-1.2860	0.5256
3	C	0.7063	-1.4233	-0.6854
4	C	-0.6688	-2.4522	0.9923
5	C	0.7777	-2.6250	-1.3684
6	H	1.2523	-0.5684	-1.0719
7	C	-0.6131	-3.6588	0.3040
8	H	-1.2601	-2.3987	1.9012
9	C	0.1137	-3.7802	-0.8974
10	H	1.3894	-2.6839	-2.2634
11	H	-1.1687	-4.5026	0.6972
12	C	-0.1947	0.0008	2.7002
13	C	-0.9354	0.9979	3.3795
14	C	0.4351	-0.9790	3.5050
15	C	-1.0385	1.0088	4.7661
16	H	-1.4529	1.7565	2.8006
17	C	0.3291	-0.9587	4.8911
18	H	1.0262	-1.7540	3.0269
19	C	-0.4076	0.0347	5.5546
20	H	-1.6309	1.7834	5.2488
21	H	0.8353	-1.7255	5.4741
22	C	-0.0654	1.2421	0.4989
23	C	0.5417	2.4073	1.0189
24	C	-0.6486	1.3672	-0.7872
25	C	0.5646	3.6082	0.3181
26	H	1.0120	2.3657	1.9966
27	C	-0.6248	2.5579	-1.4933
28	H	-1.1366	0.5048	-1.2307
29	C	-0.0172	3.7171	-0.9610
30	H	-1.0698	2.5892	-2.4830
31	C	-0.3185	-6.2034	-0.9855
32	C	-0.0324	-4.9672	-3.0460
33	C	0.2151	-7.4380	-1.7114
34	H	-1.4226	-6.2102	-1.0175
35	H	-0.0071	-6.2524	0.0623
36	C	0.4941	-6.2412	-3.6986
37	H	-1.1169	-4.8838	-3.2354
38	H	0.4540	-4.1033	-3.5021
39	H	-0.2567	-8.3414	-1.3139
40	H	1.3043	-7.5096	-1.5577
41	H	0.2233	-6.2644	-4.7583
42	H	1.5925	-6.2743	-3.6111
43	N	0.2304	-4.9948	-1.5988
44	O	-0.0802	-7.4003	-3.1033
45	N	0.0250	4.9024	-1.7199
46	C	-1.2389	5.3727	-2.3078
47	C	0.8189	6.0206	-1.2118
48	C	-0.9590	6.4212	-3.3794
49	H	-1.8822	5.8141	-1.5267
50	H	-1.7816	4.5386	-2.7560
51	C	1.0262	7.0515	-2.3205
52	H	0.3313	6.5064	-0.3478
53	H	1.7978	5.6535	-0.8888

54	H	-1.8974	6.8366	-3.7582
55	H	-0.4117	5.9565	-4.2160
56	O	-0.2119	7.5120	-2.8506
57	H	1.5459	7.9294	-1.9259
58	H	1.6388	6.6073	-3.1222
59	H	1.0368	4.4668	0.7816
60	C	-0.4932	0.0701	7.0613
61	H	0.3436	0.6361	7.4933
62	H	-1.4176	0.5510	7.3994
63	H	-0.4561	-0.9375	7.4896

SP_obn_an_m2b_PMMA
Energy (POTENTIAL) = -617.2782451 Eh

	Atom	X	Y	Z
1	O	0.1057	-1.4150	-1.0190
2	C	1.1051	-0.6910	-0.4192
3	H	1.4069	-1.0864	0.5829
4	H	2.0557	-0.7117	-1.0063
5	C	0.7725	0.7888	-0.2016
6	C	1.7212	1.6797	0.3213
7	C	-0.4897	1.2916	-0.5231
8	C	1.4164	3.0253	0.5186
9	H	2.7157	1.3114	0.5737
10	C	-0.7975	2.6408	-0.3228
11	H	-1.2250	0.5989	-0.9194
12	C	0.1458	3.5318	0.2007
13	H	2.1727	3.6971	0.9230
14	H	-1.7903	3.0082	-0.5795
15	C	-0.1908	4.9862	0.4427
16	H	-1.0920	5.2854	-0.1037
17	H	-0.3735	5.1859	1.5077
18	H	0.6271	5.6472	0.1301
19	O	-2.0717	-1.7832	0.3514
20	H	-1.1966	-1.5637	-0.1251
21	C	-2.6558	-2.8119	-0.4174
22	H	-1.9257	-3.5898	-0.6882
23	H	-3.4605	-3.2842	0.1653
24	H	-3.1097	-2.4419	-1.3570
25	O	0.6090	-3.9361	-0.6261
26	H	0.4435	-2.9600	-0.8710
27	C	0.5790	-3.9700	0.7833
28	H	-0.2599	-3.3888	1.1944
29	H	0.4698	-5.0127	1.1164
30	H	1.5078	-3.5780	1.2420

obn_rad
Energy (POTENTIAL) = -385.439530863 Eh

	Atom	X	Y	Z
1	O	0.0692	-2.9195	-0.8996
2	C	0.1192	-2.5522	0.4314
3	H	-0.7462	-2.9131	1.0097
4	H	1.0527	-2.8617	0.9278
5	C	0.0696	-1.0301	0.2993
6	C	1.2515	-0.2976	0.1265
7	C	-1.1628	-0.3658	0.2421
8	C	1.2011	1.0849	-0.0346
9	H	2.2091	-0.8114	0.1331
10	C	-1.2058	1.0168	0.0808
11	H	-2.0849	-0.9327	0.3388
12	C	-0.0263	1.7658	-0.0564
13	H	2.1255	1.6456	-0.1492
14	H	-2.1670	1.5244	0.0565
15	C	-0.0759	3.2672	-0.2000
16	H	-0.9947	3.5920	-0.6992
17	H	-0.0509	3.7529	0.7846
18	H	0.7793	3.6440	-0.7703

SP_ar3m2obn_MeProp
Energy (POTENTIAL) = -1731.6045676 Eh

	Atom	X	Y	Z
1	C	-0.1381	0.2977	-0.8994
2	C	-1.4377	-0.3577	-0.4145
3	C	-2.6216	-0.1376	-1.1390
4	C	-1.5529	-1.0276	0.8053
5	C	-3.8500	-0.5925	-0.6839
6	H	-2.5778	0.4166	-2.0717
7	C	-2.7813	-1.5056	1.2683
8	H	-0.6715	-1.1960	1.4150
9	C	-3.9609	-1.3013	0.5323
10	H	-4.7415	-0.3725	-1.2629
11	H	-2.8022	-2.0478	2.2064
12	C	1.1486	-0.2836	-0.2796
13	C	2.2104	0.5647	0.0648
14	C	1.3615	-1.6602	-0.1568
15	C	3.4175	0.0623	0.5359
16	H	2.0917	1.6360	-0.0551

17	C	2.5653	-2.1759	0.3251
18	H	0.5720	-2.3550	-0.4268
19	C	3.6242	-1.3232	0.6856
20	H	4.2229	0.7544	0.7624
21	H	2.6626	-3.2505	0.4274
22	C	0.0233	0.1883	-2.4268
23	C	0.4969	1.2657	-3.1816
24	C	-0.2282	-1.0229	-3.0856
25	C	0.7047	1.1345	-4.5565
26	H	0.7026	2.2097	-2.6911
27	C	-0.0174	-1.1490	-4.4567
28	H	-0.6005	-1.8739	-2.5229
29	C	0.4512	-0.0707	-5.2210
30	H	1.0726	1.9886	-5.1213
31	H	-0.2224	-2.1007	-4.9424
32	N	4.8729	-1.8018	1.1406
33	C	5.3801	-1.2310	2.3981
34	C	5.1113	-3.2435	1.0876
35	C	6.8551	-1.5779	2.5702
36	H	4.8113	-1.6273	3.2579
37	H	5.2656	-0.1453	2.3926
38	C	6.5968	-3.5270	1.3054
39	H	4.5226	-3.7840	1.8508
40	H	4.8222	-3.6233	0.1026
41	H	7.2175	-1.2291	3.5417
42	H	7.4432	-1.0912	1.7749
43	O	7.0603	-2.9872	2.5387
44	H	6.7731	-4.6058	1.3486
45	H	7.1756	-3.1061	0.4671
46	N	-5.2268	-1.7291	0.9819
47	C	-6.0545	-2.4825	0.0266
48	C	-5.3177	-2.3044	2.3234
49	C	-7.4924	-2.5608	0.5290
50	H	-5.6584	-3.5052	-0.1004
51	H	-6.0432	-1.9952	-0.9499
52	C	-6.7826	-2.3958	2.7487
53	H	-4.8605	-3.3091	2.3669
54	H	-4.7891	-1.6587	3.0315
55	H	-8.0923	-3.1904	-0.1345
56	H	-7.9305	-1.5495	0.5528
57	O	-7.5548	-3.1525	1.8230
58	H	-6.8631	-2.9029	3.7146
59	H	-7.1985	-1.3796	2.8460
60	O	-0.2131	1.7143	-0.6314
61	C	-0.5325	2.0928	0.7144
62	H	-1.6121	1.9791	0.8796
63	H	-0.0162	1.4458	1.4339
64	C	-0.1019	3.5249	0.8892
65	C	1.0313	3.8542	1.6392
66	C	-0.8103	4.5573	0.2603
67	C	1.4420	5.1830	1.7658
68	H	1.6004	3.0644	2.1244
69	C	-0.3965	5.8816	0.3833
70	H	-1.6879	4.3154	-0.3344
71	C	0.7355	6.2184	1.1422
72	H	2.3254	5.4177	2.3555
73	H	-0.9585	6.6692	-0.1141
74	C	1.1588	7.6603	1.2988
75	H	1.0257	8.2198	0.3661
76	H	0.5591	8.1665	2.0670
77	H	2.2091	7.7405	1.5976
78	C	0.6476	-0.2033	-6.7131
79	H	-0.3010	-0.0734	-7.2512
80	H	1.3451	0.5499	-7.0943
81	H	1.0357	-1.1929	-6.9796

obn_an_m2_MeProp
Energy (POTENTIAL) = -617.071754360 Eh

	Atom	X	Y	Z
1	O	0.4605	-1.6832	-1.2595
2	C	1.2297	-0.9333	-0.4014
3	H	1.2721	-1.3465	0.6372
4	H	2.3011	-0.9029	-0.7131
5	C	0.7868	0.5250	-0.2542
6	C	1.5824	1.4597	0.4245
7	C	-0.4283	0.9640	-0.7855
8	C	1.1761	2.7850	0.5694
9	H	2.5391	1.1432	0.8398
10	C	-0.8384	2.2930	-0.6372
11	H	-1.0522	0.2398	-1.2989
12	C	-0.0472	3.2274	0.0405
13	H	1.8165	3.4917	1.0957
14	H	-1.7914	2.6099	-1.0587
15	C	-0.4956	4.6602	0.2210
16	H	-1.3142	4.9133	-0.4615
17	H	-0.8532	4.8441	1.2435

18	H	0.3249	5.3650	0.0390
19	O	-1.9828	-2.0548	-0.4441
20	H	-1.0170	-1.9100	-0.7514
21	C	-2.0371	-1.7018	0.9176
22	H	-1.6954	-0.6694	1.1040
23	H	-3.0787	-1.7734	1.2616
24	H	-1.4334	-2.3695	1.5581
25	O	1.2360	-4.1030	-0.7519
26	H	0.9646	-3.1589	-1.0298
27	C	0.3620	-4.4425	0.2995
28	H	-0.6875	-4.2001	0.0707
29	H	0.4314	-5.5234	0.4921
30	H	0.6084	-3.9276	1.2491

ar3no2cnoac_PMMA
Energy (POTENTIAL) = -1541.45811428 Eh

	Atom	X	Y	Z
1	C	-0.3324	0.0155	-0.3431
2	C	0.2547	1.4432	-0.2054
3	C	0.0564	2.1971	0.9613
4	C	0.6262	3.4587	1.1113
5	C	1.4060	3.9706	0.0784
6	C	1.6393	3.2450	-1.0880
7	C	1.0678	1.9839	-1.2166
8	H	1.2816	1.4173	-2.1154
9	H	2.2611	3.6606	-1.8708
10	H	0.4716	4.0393	2.0120
11	H	-0.5459	1.7987	1.7679
12	C	-1.5137	-0.1824	0.6335
13	C	-1.5256	-1.1719	1.6196
14	C	-2.6303	-1.3317	2.4599
15	C	-3.7570	-0.5126	2.3471
16	C	-3.7391	0.4876	1.3632
17	C	-2.6387	0.6552	0.5293
18	H	-2.6570	1.4479	-0.2121
19	H	-4.5964	1.1471	1.2521
20	H	-2.6107	-2.1151	3.2134
21	H	-0.6781	-1.8360	1.7390
22	C	-0.9365	-0.1102	-1.8339
23	H	-1.2648	0.8900	-2.1258
24	C	0.0295	-0.5459	-2.8454
25	N	0.7825	-0.8754	-3.6647
26	C	-2.1847	-1.0149	-1.9288
27	O	-2.2086	-2.2064	-1.7161
28	O	-3.2423	-0.2843	-2.2904
29	C	-4.5065	-0.9877	-2.3197
30	H	-4.4519	-1.8357	-3.0053
31	H	-5.2349	-0.2545	-2.6626
32	H	-4.7501	-1.3380	-1.3136
33	C	0.7884	-1.0156	-0.0796
34	C	1.9153	-0.6654	0.6805
35	C	2.9118	-1.5943	0.9667
36	C	2.7760	-2.8929	0.4851
37	C	1.6659	-3.2803	-0.2619
38	C	0.6774	-2.3410	-0.5343
39	H	-0.1977	-2.6532	-1.0904
40	H	1.5805	-4.2992	-0.6179
41	H	3.7809	-1.3200	1.5510
42	H	2.0308	0.3419	1.0598
43	C	-4.9439	-0.6721	3.2661
44	H	-4.9541	-1.6578	3.7414
45	H	-4.9246	0.0804	4.0651
46	H	-5.8876	-0.5431	2.7245
47	N	2.0053	5.3035	0.2260
48	O	2.6925	5.7334	-0.7017
49	O	1.7887	5.9212	1.2700
50	N	3.8235	-3.8792	0.7791
51	O	3.6770	-5.0240	0.3475
52	O	4.7940	-3.5114	1.4439

ar3no2obn_meoh_PMMA
Energy (POTENTIAL) = -1682.68139993 Eh

	Atom	X	Y	Z
1	C	-0.3248	-0.3949	-0.2731
2	C	-1.7002	-0.2596	0.4011
3	C	-2.6523	0.5771	-0.2079
4	C	-1.9929	-0.8209	1.6503
5	C	-3.8777	0.8226	0.3988
6	H	-2.4220	1.0605	-1.1508
7	C	-3.2194	-0.5895	2.2701
8	H	-1.2648	-1.4422	2.1579
9	C	-4.1484	0.2254	1.6304
10	H	-4.6143	1.4667	-0.0646
11	H	-3.4529	-1.0252	3.2333
12	C	0.5695	-1.5111	0.2977
13	C	1.9186	-1.2637	0.5828

14	C	0.0770	-2.8200	0.4284
15	C	2.7536	-2.2861	1.0249
16	H	2.3222	-0.2682	0.4463
17	C	0.8959	-3.8523	0.8681
18	H	-0.9562	-3.0363	0.1787
19	C	2.2288	-3.5683	1.1684
20	H	3.7957	-2.0999	1.2527
21	H	0.5202	-4.8619	0.9760
22	C	-0.4313	-0.6767	-1.7816
23	C	0.5750	-0.2239	-2.6438
24	C	-1.4617	-1.4616	-2.3101
25	C	0.5305	-0.5256	-4.0034
26	H	1.3932	0.3687	-2.2497
27	C	-1.4957	-1.7654	-3.6708
28	H	-2.2515	-1.8336	-1.6647
29	C	-0.5063	-1.2979	-4.5445
30	H	1.3199	-0.1575	-4.6547
31	H	-2.3099	-2.3728	-4.0586
32	O	0.3498	0.8785	-0.1695
33	C	0.3960	1.5067	1.1387
34	H	-0.5697	1.9874	1.3304
35	H	0.5597	0.7479	1.9111
36	C	1.5187	2.5065	1.1149
37	C	2.7267	2.2484	1.7701
38	C	1.3814	3.7050	0.3998
39	C	3.7770	3.1667	1.7129
40	H	2.8489	1.3257	2.3329
41	C	2.4342	4.6145	0.3406
42	H	0.4506	3.9107	-0.1221
43	C	3.6491	4.3635	0.9979
44	H	4.7081	2.9491	2.2308
45	H	2.3130	5.5377	-0.2218
46	C	4.7724	5.3721	0.9527
47	H	4.8674	5.8194	-0.0431
48	H	4.5921	6.1929	1.6592
49	H	5.7327	4.9170	1.2156
50	C	-0.5664	-1.5929	-6.0239
51	H	-1.0749	-0.7834	-6.5640
52	H	0.4363	-1.6897	-6.4537
53	H	-1.1168	-2.5173	-6.2270
54	C	-0.1580	3.2259	-2.9438
55	H	-0.7247	4.0125	-3.4509
56	H	0.8216	3.6373	-2.6553
57	H	0.0056	2.4010	-3.6518
58	O	-0.9168	2.8170	-1.8125
59	H	-0.4068	2.1235	-1.3494
60	N	3.1007	-4.6498	1.6385
61	O	4.2757	-4.3773	1.8936
62	O	2.6138	-5.7763	1.7562
63	N	-5.4447	0.4734	2.2763
64	O	-5.6601	-0.0619	3.3653
65	O	-6.2491	1.2035	1.6952

ar3m2cnoac
Energy (POTENTIAL) = -1705.68066150 Eh

	Atom	X	Y	Z
1	C	-1.0507	0.0018	-0.4277
2	C	-0.2634	1.3283	-0.3350
3	C	-0.1546	2.0284	0.8710
4	C	0.6096	3.1910	0.9847
5	C	1.3206	3.7023	-0.1152
6	C	1.2335	2.9837	-1.3253
7	C	0.4659	1.8308	-1.4259
8	H	0.4689	1.3065	-2.3762
9	H	1.7980	3.3128	-2.1917
10	H	0.6279	3.7012	1.9405
11	H	-0.6820	1.6727	1.7493
12	C	-2.1599	-0.0299	0.6494
13	C	-2.2606	-1.0406	1.6083
14	C	-3.3009	-1.0441	2.5426
15	C	-4.2746	-0.0417	2.5536
16	C	-4.1658	0.9810	1.5982
17	C	-3.1270	0.9917	0.6734
18	H	-3.0663	1.8057	-0.0420
19	H	-4.9022	1.7813	1.5825
20	H	-3.3527	-1.8484	3.2727
21	H	-1.5313	-1.8414	1.6299
22	C	-1.8055	-0.0125	-1.8598
23	H	-1.9863	1.0300	-2.1312
24	C	-1.0154	-0.5974	-2.9449
25	N	-0.4061	-1.0378	-3.8295
26	C	-3.1871	-0.6949	-1.8392
27	O	-3.3928	-1.8755	-1.6561
28	O	-4.1424	0.2136	-2.0579
29	C	-5.5024	-0.2632	-1.9381
30	H	-5.6780	-1.0896	-2.6298

31	H	-6.1299	0.5916	-2.1859
32	H	-5.6830	-0.5909	-0.9112
33	C	-0.0792	-1.1898	-0.2886
34	C	1.1944	-1.0322	0.2794
35	C	2.0593	-2.1078	0.4498
36	C	1.6918	-3.4128	0.0677
37	C	0.4113	-3.5763	-0.4930
38	C	-0.4446	-2.4896	-0.6654
39	H	-1.4286	-2.6723	-1.0814
40	H	0.0576	-4.5576	-0.7870
41	H	3.0463	-1.9209	0.8604
42	H	1.5344	-0.0501	0.5859
43	N	2.1311	4.8522	-0.0359
44	C	1.9295	5.8798	-1.0706
45	C	2.3249	5.4619	1.2801
46	C	3.0872	6.8722	-1.0550
47	H	0.9844	6.4216	-0.8921
48	H	1.8696	5.4172	-2.0572
49	C	3.4685	6.4731	1.2166
50	H	1.4086	5.9678	1.6328
51	H	2.5864	4.6857	2.0057
52	H	2.8991	7.6846	-1.7630
53	H	4.0188	6.3580	-1.3425
54	O	3.2347	7.4722	0.2291
55	H	3.5644	6.9926	2.1743
56	H	4.4122	5.9449	1.0037
57	N	2.6018	-4.4786	0.2149
58	C	3.2558	-4.6224	1.5255
59	C	2.2238	-5.7825	-0.3307
60	C	4.4411	-5.5761	1.4183
61	H	2.5399	-5.0143	2.2689
62	H	3.6109	-3.6535	1.8805
63	C	3.4445	-6.7001	-0.3717
64	H	1.4282	-6.2589	0.2694
65	H	1.8506	-5.6554	-1.3513
66	H	4.8761	-5.7518	2.4065
67	H	5.2109	-5.1360	0.7636
68	O	4.0381	-6.8465	0.9147
69	H	3.1510	-7.7015	-0.6996
70	H	4.1822	-6.2959	-1.0838
71	C	-5.3947	-0.0369	3.5662
72	H	-5.4678	-0.9957	4.0889
73	H	-5.2393	0.7433	4.3226
74	H	-6.3606	0.1669	3.0896

obn_an_m2c_MeButa
Energy (POTENTIAL) = -617.068552861 Eh

	Atom	X	Y	Z
1	O	0.8106	-1.3018	-1.2765
2	C	1.4961	-0.3587	-0.4904
3	H	1.7083	-0.7576	0.5200
4	H	2.4763	-0.1707	-0.9543
5	C	0.7719	0.9730	-0.3260
6	C	1.4752	2.1810	-0.2940
7	C	-0.6224	1.0106	-0.1697
8	C	0.8121	3.3970	-0.1037
9	H	2.5563	2.1772	-0.4265
10	C	-1.2793	2.2259	0.0179
11	H	-1.1768	0.0735	-0.1929
12	C	-0.5774	3.4407	0.0575
13	H	1.3821	4.3245	-0.0900
14	H	-2.3620	2.2354	0.1355
15	C	-1.3031	4.7474	0.2856
16	H	-2.2247	4.8032	-0.3059
17	H	-1.5920	4.8654	1.3389
18	H	-0.6784	5.6068	0.0192
19	O	-1.1658	-2.1424	0.1427
20	H	0.0170	-1.6554	-0.7161
21	C	-0.8117	-2.1053	1.4824
22	H	-0.5840	-1.0761	1.8376
23	H	-1.6166	-2.4816	2.1531
24	H	0.0877	-2.7132	1.7323
25	O	-0.8146	-4.6416	-0.4740
26	H	-1.0461	-3.6748	-0.2494
27	C	0.5928	-4.7053	-0.4419
28	H	0.9929	-4.8869	0.5757
29	H	0.9383	-5.5378	-1.0743
30	H	1.0593	-3.7796	-0.8108

meoh_MeButa
Energy (POTENTIAL) = -115.719290945 Eh

	Atom	X	Y	Z
1	C	0.0191	0.0016	-0.6648
2	H	-0.9918	0.0065	-1.0826
3	H	0.5486	0.8921	-1.0362
4	H	0.5404	-0.8933	-1.0372

5	O	-0.1209	0.0013	0.7525
6	H	0.7714	0.0003	1.1315

ar3no2_cat_MeButa
Energy (POTENTIAL) = -1181.22260308 Eh

	Atom	X	Y	Z
1	C	-0.0054	-0.0026	0.5226
2	C	0.0348	-1.2523	-0.2303
3	C	0.7801	-1.3371	-1.4299
4	C	-0.6843	-2.3849	0.2191
5	C	0.8293	-2.5251	-2.1449
6	H	1.3529	-0.4833	-1.7730
7	C	-0.6615	-3.5673	-0.5078
8	H	-1.2964	-2.3166	1.1110
9	C	0.1020	-3.6170	-1.6725
10	H	1.4148	-2.6140	-3.0509
11	H	-1.2251	-4.4348	-0.1896
12	C	-0.0060	-0.0071	1.9473
13	C	-0.5923	1.0654	2.6869
14	C	0.5782	-1.0851	2.6802
15	C	-0.6027	1.0438	4.0658
16	H	-1.0928	1.8689	2.1590
17	C	0.5839	-1.0750	4.0596
18	H	1.0762	-1.8869	2.1476
19	C	-0.0086	-0.0175	4.7817
20	H	-1.0856	1.8485	4.6114
21	H	1.0596	-1.8873	4.6000
22	C	-0.0443	1.2521	-0.2221
23	C	0.6734	2.3818	0.2364
24	C	-0.7870	1.3447	-1.4227
25	C	0.6514	3.5693	-0.4823
26	H	1.2836	2.3077	1.1291
27	C	-0.8352	2.5377	-2.1298
28	H	-1.3586	0.4930	-1.7731
29	C	-0.1097	3.6266	-1.6482
30	H	-1.4188	2.6324	-3.0363
31	H	1.2138	4.4349	-0.1569
32	C	0.0163	-0.0044	6.2804
33	H	0.8936	0.5595	6.6272
34	H	-0.8689	0.4898	6.6914
35	H	0.0908	-1.0140	6.6935
36	N	-0.1450	4.8945	-2.4105
37	O	-0.8273	4.9243	-3.4319
38	O	0.5094	5.8384	-1.9738
39	N	0.1381	-4.8796	-2.4436
40	O	0.8237	-4.9031	-3.4630
41	O	-0.5189	-5.8257	-2.0156

ar3no2cnoac
Energy (POTENTIAL) = -1541.46437403 Eh

	Atom	X	Y	Z
1	C	-0.3126	0.0178	-0.3384
2	C	0.2964	1.4368	-0.2126
3	C	0.1361	2.1850	0.9637
4	C	0.7240	3.4385	1.1076
5	C	1.4838	3.9479	0.0578
6	C	1.6787	3.2275	-1.1190
7	C	1.0893	1.9742	-1.2409
8	H	1.2717	1.4144	-2.1508
9	H	2.2840	3.6397	-1.9162
10	H	0.5973	4.0130	2.0163
11	H	-0.4502	1.7883	1.7828
12	C	-1.4991	-0.1473	0.6381
13	C	-1.5332	-1.1239	1.6361
14	C	-2.6431	-1.2496	2.4764
15	C	-3.7518	-0.4081	2.3508
16	C	-3.7099	0.5808	1.3555
17	C	-2.6047	0.7149	0.5225
18	H	-2.6037	1.5003	-0.2269
19	H	-4.5521	1.2578	1.2351
20	H	-2.6409	-2.0229	3.2404
21	H	-0.6996	-1.8029	1.7684
22	C	-0.9142	-0.1173	-1.8306
23	H	-1.2127	0.8857	-2.1434
24	C	0.0458	-0.6003	-2.8257
25	N	0.7964	-0.9706	-3.6298
26	C	-2.1868	-0.9890	-1.9163
27	O	-2.2396	-2.1789	-1.6926
28	O	-3.2236	-0.2355	-2.2816
29	C	-4.5103	-0.8998	-2.2991
30	H	-4.4833	-1.7578	-2.9737
31	H	-5.2155	-0.1488	-2.6509
32	H	-4.7627	-1.2262	-1.2873
33	C	0.7892	-1.0308	-0.0633
34	C	1.9418	-0.6879	0.6603
35	C	2.9204	-1.6343	0.9517

36	C	2.7406	-2.9425	0.5120
37	C	1.6042	-3.3225	-0.1998
38	C	0.6352	-2.3656	-0.4779
39	H	-0.2588	-2.6686	-1.0087
40	H	1.4831	-4.3484	-0.5238
41	H	3.8083	-1.3640	1.5089
42	H	2.0924	0.3258	1.0082
43	C	-4.9475	-0.5331	3.2636
44	H	-4.9277	-1.4721	3.8250
45	H	-4.9734	0.2895	3.9899
46	H	-5.8849	-0.4913	2.6977
47	N	2.1015	5.2706	0.1976
48	O	2.7715	5.7008	-0.7438
49	O	1.9209	5.8875	1.2497
50	N	3.7672	-3.9456	0.8111
51	O	3.5867	-5.0985	0.4124
52	O	4.7614	-3.5886	1.4475

obn_an_m2c_MeProp
Energy (POTENTIAL) = -617.069971404 Eh

	Atom	X	Y	Z
1	O	0.8090	-1.3020	-1.2750
2	C	1.4950	-0.3587	-0.4890
3	H	1.7072	-0.7575	0.5213
4	H	2.4752	-0.1711	-0.9532
5	C	0.7711	0.9732	-0.3251
6	C	1.4749	2.1811	-0.2946
7	C	-0.6230	1.0114	-0.1681
8	C	0.8121	3.3974	-0.1049
9	H	2.5559	2.1769	-0.4277
10	C	-1.2796	2.2271	0.0188
11	H	-1.1778	0.0745	-0.1903
12	C	-0.5774	3.4416	0.0571
13	H	1.3824	4.3247	-0.0923
14	H	-2.3622	2.2369	0.1370
15	C	-1.3026	4.7488	0.2845
16	H	-2.2252	4.8037	-0.3054
17	H	-1.5894	4.8682	1.3381
18	H	-0.6781	5.6077	0.0157
19	O	-1.1671	-2.1440	0.1442
20	H	0.0159	-1.6556	-0.7142
21	C	-0.8122	-2.1073	1.4839
22	H	-0.5830	-1.0784	1.8388
23	H	-1.6172	-2.4824	2.1550
24	H	0.0865	-2.7162	1.7333
25	O	-0.8130	-4.6407	-0.4790
26	H	-1.0446	-3.6747	-0.2509
27	C	0.5944	-4.7057	-0.4438
28	H	0.9920	-4.8866	0.5748
29	H	0.9403	-5.5390	-1.0747
30	H	1.0625	-3.7806	-0.8125

cnoac_rad
Energy (POTENTIAL) = -359.986113310 Eh

	Atom	X	Y	Z
1	C	-0.8645	0.7919	-0.0050
2	H	-0.7736	1.8720	0.0260
3	C	-2.1274	0.2093	-0.0663
4	N	-3.1947	-0.2738	-0.1173
5	C	0.3361	-0.0593	0.0185
6	O	0.3179	-1.2785	-0.0145
7	O	1.4439	0.6953	0.0817
8	C	2.6910	-0.0326	0.1126
9	H	2.7277	-0.6796	0.9927
10	H	3.4689	0.7281	0.1595
11	H	2.7959	-0.6393	-0.7904

SP_obn_an_m2b
Energy (POTENTIAL) = -617.298544 Eh

	Atom	X	Y	Z
1	O	0.3318	-1.5791	-0.7562
2	C	1.2062	-0.7867	-0.0310
3	H	1.2932	-1.1059	1.0365
4	H	2.2473	-0.8488	-0.4185
5	C	0.8176	0.6919	-0.0142
6	C	1.7591	1.7087	-0.2001
7	C	-0.5176	1.0689	0.2037
8	C	1.3884	3.0578	-0.1667
9	H	2.8000	1.4459	-0.3834
10	C	-0.8867	2.4121	0.2382
11	H	-1.2605	0.2904	0.3551
12	C	0.0592	3.4343	0.0539
13	H	2.1432	3.8268	-0.3220
14	H	-1.9288	2.6784	0.4098
15	C	-0.3529	4.8880	0.1072
16	H	-1.2367	5.0777	-0.5139

17	H	-0.6115	5.1927	1.1301
18	H	0.4506	5.5465	-0.2394
19	O	-1.8909	-2.0162	0.4508
20	H	-0.9720	-1.8421	0.0142
21	C	-2.8193	-2.0020	-0.6093
22	H	-2.6200	-2.7826	-1.3652
23	H	-3.8259	-2.1829	-0.2083
24	H	-2.8436	-1.0337	-1.1405
25	O	1.2071	-3.9924	-0.4606
26	H	0.8839	-3.0380	-0.6367
27	C	0.7028	-4.3323	0.8116
28	H	-0.3531	-4.0441	0.9367
29	H	0.7791	-5.4203	0.9523
30	H	1.2649	-3.8590	1.6403

ar3m2obn
Energy (POTENTIAL) = -1731.15919733 Eh

	Atom	X	Y	Z
1	C	-0.1344	0.2964	-0.8974
2	C	-1.4346	-0.3587	-0.4141
3	C	-2.6184	-0.1364	-1.1383
4	C	-1.5509	-1.0305	0.8047
5	C	-3.8477	-0.5906	-0.6844
6	H	-2.5749	0.4183	-2.0707
7	C	-2.7798	-1.5079	1.2668
8	H	-0.6703	-1.2011	1.4148
9	C	-3.9600	-1.3008	0.5314
10	H	-4.7377	-0.3692	-1.2647
11	H	-2.8007	-2.0515	2.2040
12	C	1.1517	-0.2857	-0.2770
13	C	2.2134	0.5614	0.0708
14	C	1.3647	-1.6629	-0.1571
15	C	3.4202	0.0582	0.5421
16	H	2.0952	1.6331	-0.0450
17	C	2.5683	-2.1798	0.3242
18	H	0.5756	-2.3575	-0.4286
19	C	3.6274	-1.3281	0.6882
20	H	4.2237	0.7509	0.7730
21	H	2.6646	-3.2547	0.4241
22	C	0.0290	0.1873	-2.4247
23	C	0.5071	1.2633	-3.1792
24	C	-0.2243	-1.0232	-3.0845
25	C	0.7170	1.1320	-4.5540
26	H	0.7151	2.2070	-2.6889
27	C	-0.0112	-1.1498	-4.4554
28	H	-0.5996	-1.8737	-2.5230
29	C	0.4614	-0.0726	-5.2192
30	H	1.0880	1.9854	-5.1179
31	H	-0.2179	-2.1010	-4.9413
32	N	4.8755	-1.8084	1.1412
33	C	5.3913	-1.2314	2.3929
34	C	5.1087	-3.2518	1.0958
35	C	6.8656	-1.5827	2.5604
36	H	4.8248	-1.6204	3.2573
37	H	5.2821	-0.1454	2.3820
38	C	6.5940	-3.5396	1.3091
39	H	4.5205	-3.7850	1.8641
40	H	4.8154	-3.6373	0.1145
41	H	7.2332	-1.2282	3.5278
42	H	7.4522	-1.1042	1.7593
43	O	7.0653	-2.9938	2.5374
44	H	6.7656	-4.6189	1.3575
45	H	7.1711	-3.1261	0.4661
46	N	-5.2254	-1.7261	0.9823
47	C	-6.0652	-2.4613	0.0228
48	C	-5.3133	-2.3166	2.3180
49	C	-7.5004	-2.5352	0.5328
50	H	-5.6772	-3.4849	-0.1192
51	H	-6.0578	-1.9624	-0.9477
52	C	-6.7761	-2.4035	2.7508
53	H	-4.8616	-3.3240	2.3466
54	H	-4.7779	-1.6831	3.0316
55	H	-8.1085	-3.1514	-0.1358
56	H	-7.9309	-1.5213	0.5726
57	O	-7.5598	-3.1435	1.8200
58	H	-6.8533	-2.9220	3.7108
59	H	-7.1850	-1.3863	2.8634
60	O	-0.2091	1.7137	-0.6291
61	C	-0.5324	2.0921	0.7167
62	H	-1.6116	1.9740	0.8802
63	H	-0.0141	1.4478	1.4370
64	C	-0.1085	3.5264	0.8903
65	C	1.0370	3.8596	1.6199
66	C	-0.8352	4.5571	0.2795
67	C	1.4425	5.1905	1.7435
68	H	1.6194	3.0719	2.0924

69	C	-0.4269	5.8838	0.3996
70	H	-1.7241	4.3132	-0.2978
71	C	0.7179	6.2243	1.1375
72	H	2.3354	5.4279	2.3173
73	H	-1.0035	6.6699	-0.0831
74	C	1.1359	7.6680	1.2917
75	H	0.9824	8.2300	0.3637
76	H	0.5469	8.1676	2.0723
77	H	2.1908	7.7529	1.5723
78	C	0.6599	-0.2058	-6.7110
79	H	-0.2883	-0.0763	-7.2497
80	H	1.3574	0.5476	-7.0916
81	H	1.0477	-1.1956	-6.9764

SP_ar3m2_cat
Energy (POTENTIAL) = -1345.8329385 Eh

	Atom	X	Y	Z
1	C	-0.1734	0.0030	1.1688
2	C	-0.1948	-1.2388	0.4546
3	C	0.3467	-1.3705	-0.8570
4	C	-0.7537	-2.4182	1.0246
5	C	0.3302	-2.5627	-1.5394
6	H	0.8501	-0.5219	-1.3074
7	C	-0.7973	-3.6109	0.3428
8	H	-1.2207	-2.3619	2.0017
9	C	-0.2489	-3.7374	-0.9696
10	H	0.8200	-2.6059	-2.5035
11	H	-1.3038	-4.4467	0.8071
12	C	-0.1241	-0.0150	2.6358
13	C	-0.8860	0.8961	3.3990
14	C	0.6818	-0.9465	3.3247
15	C	-0.8479	0.8654	4.7872
16	H	-1.5359	1.6021	2.8921
17	C	0.7294	-0.9547	4.7134
18	H	1.2929	-1.6426	2.7594
19	C	-0.0347	-0.0530	5.4708
20	H	-1.4622	1.5602	5.3538
21	H	1.3712	-1.6697	5.2214
22	C	-0.2015	1.2624	0.4853
23	C	0.3916	2.4308	1.0452
24	C	-0.8402	1.4256	-0.7766
25	C	0.3798	3.6407	0.3949
26	H	0.9211	2.3536	1.9885
27	C	-0.8797	2.6368	-1.4262
28	H	-1.3782	0.5882	-1.2079
29	C	-0.2544	3.7962	-0.8753
30	H	-1.4485	2.7064	-2.3440
31	C	-0.6849	-6.1878	-1.0190
32	C	-0.1535	-5.0368	-3.0988
33	C	0.2108	-7.3339	-1.4995
34	H	-1.7317	-6.3927	-1.2832
35	H	-0.6034	-6.1281	0.0656
36	C	0.7304	-6.2206	-3.4858
37	H	-1.1639	-5.1943	-3.5022
38	H	0.2428	-4.1232	-3.5361
39	H	-0.1781	-8.2839	-1.1233
40	H	1.2302	-7.1876	-1.1082
41	H	0.7211	-6.3519	-4.5711
42	H	1.7655	-6.0361	-3.1574
43	N	-0.2563	-4.9265	-1.6364
44	O	0.2376	-7.4287	-2.9176
45	N	-0.2551	4.9905	-1.5342
46	C	-0.7746	5.1461	-2.8988
47	C	-0.0761	6.2852	-0.8601
48	C	0.0867	6.1470	-3.6748
49	H	-1.8160	5.4952	-2.8617
50	H	-0.7431	4.1943	-3.4278
51	C	0.7656	7.2261	-1.7193
52	H	-1.0683	6.7313	-0.7017
53	H	0.3942	6.1606	0.1125
54	H	-0.3679	6.3406	-4.6501
55	H	1.0904	5.7201	-3.8296
56	O	0.1825	7.3974	-3.0058
57	H	0.8077	8.2145	-1.2541
58	H	1.7899	6.8313	-1.8114
59	H	0.9045	4.4698	0.8512
60	C	0.0351	-0.0507	6.9766
61	H	0.7930	0.6635	7.3248
62	H	-0.9201	0.2474	7.4205
63	H	0.3091	-1.0354	7.3677

SP_ar3m2obn_meoh_PMMA
Energy (POTENTIAL) = -1847.3881199 Eh

	Atom	X	Y	Z
1	C	-0.0763	0.1740	-0.7612
2	C	-1.3083	-0.6167	-0.3101

3	C	-2.5535	-0.3302	-0.8996
4	C	-1.2999	-1.5019	0.7695
5	C	-3.7220	-0.9234	-0.4473
6	H	-2.6124	0.3992	-1.7009
7	C	-2.4684	-2.1193	1.2215
8	H	-0.3679	-1.7317	1.2743
9	C	-3.7094	-1.8462	0.6213
10	H	-4.6645	-0.6427	-0.9066
11	H	-2.3944	-2.8227	2.0426
12	C	1.2672	-0.3803	-0.2556
13	C	2.2566	0.4718	0.2524
14	C	1.5953	-1.7315	-0.4050
15	C	3.5034	-0.0127	0.6319
16	H	2.0563	1.5343	0.3306
17	C	2.8385	-2.2303	-0.0175
18	H	0.8648	-2.4183	-0.8225
19	C	3.8229	-1.3793	0.5179
20	H	4.2518	0.6860	0.9935
21	H	3.0267	-3.2921	-0.1274
22	C	0.0424	0.2721	-2.2914
23	C	0.6556	1.3867	-2.8782
24	C	-0.3554	-0.7792	-3.1239
25	C	0.8385	1.4547	-4.2581
26	H	0.9899	2.2043	-2.2493
27	C	-0.1639	-0.7086	-4.5043
28	H	-0.8263	-1.6586	-2.6959
29	C	0.4291	0.4109	-5.0991
30	H	1.3131	2.3339	-4.6888
31	H	-0.4866	-1.5389	-5.1287
32	N	5.1064	-1.8330	0.8951
33	C	5.5535	-1.4828	2.2523
34	C	5.4667	-3.2108	0.5647
35	C	7.0513	-1.7356	2.3891
36	H	5.0138	-2.0866	3.0033
37	H	5.3461	-0.4306	2.4569
38	C	6.9695	-3.4087	0.7589
39	H	4.9208	-3.9385	1.1923
40	H	5.2174	-3.4089	-0.4826
41	H	7.3748	-1.5590	3.4192
42	H	7.6026	-1.0540	1.7207
43	O	7.3746	-3.0885	2.0853
44	H	7.2377	-4.4563	0.5932
45	H	7.5164	-2.7832	0.0346
46	N	-4.9175	-2.4106	1.0761
47	C	-5.7984	-3.0145	0.0644
48	C	-4.8810	-3.2189	2.2938
49	C	-7.1867	-3.2480	0.6519
50	H	-5.3830	-3.9781	-0.2794
51	H	-5.8826	-2.3578	-0.8033
52	C	-6.3028	-3.4499	2.8040
53	H	-4.3924	-4.1945	2.1205
54	H	-4.3159	-2.6867	3.0652
55	H	-7.8193	-3.7759	-0.0678
56	H	-7.6530	-2.2785	0.8924
57	O	-7.1232	-4.0639	1.8170
58	H	-6.2891	-4.1238	3.6656
59	H	-6.7401	-2.4866	3.1141
60	O	-0.2301	1.5536	-0.3163
61	C	-0.5737	1.7521	1.0731
62	H	-1.6444	1.5586	1.2054
63	H	-0.0216	1.0430	1.6987
64	C	-0.2185	3.1712	1.4268
65	C	0.9262	3.4610	2.1763
66	C	-1.0145	4.2344	0.9782
67	C	1.2693	4.7815	2.4724
68	H	1.5557	2.6478	2.5308
69	C	-0.6653	5.5510	1.2714
70	H	-1.8972	4.0225	0.3807
71	C	0.4799	5.8483	2.0261
72	H	2.1636	4.9851	3.0573
73	H	-1.2923	6.3638	0.9110
74	C	0.8310	7.2773	2.3684
75	H	0.5871	7.9573	1.5447
76	H	0.2706	7.6209	3.2480
77	H	1.8964	7.3861	2.5965
78	C	0.6015	0.5061	-6.5967
79	H	-0.2178	1.0773	-7.0532
80	H	1.5355	1.0146	-6.8608
81	H	0.6092	-0.4842	-7.0641
82	C	-1.8173	3.8753	-2.5647
83	H	-2.6788	4.3883	-3.0043
84	H	-1.2191	4.6182	-2.0140
85	H	-1.2008	3.4689	-3.3793
86	O	-2.3173	2.8554	-1.7117
87	H	-1.5458	2.4078	-1.3065

SP_ar3no2_rad_PMMA
Energy (POTENTIAL) = -1181.7251756 Eh

	Atom	X	Y	Z
1	C	-0.0033	-0.0021	0.4763
2	C	-0.0065	-1.2649	-0.2499
3	C	0.6482	-1.3996	-1.5018
4	C	-0.6575	-2.4100	0.2781
5	C	0.6552	-2.6027	-2.1881
6	H	1.1808	-0.5520	-1.9184
7	C	-0.6652	-3.6153	-0.4042
8	H	-1.1840	-2.3333	1.2227
9	C	-0.0057	-3.7034	-1.6340
10	H	1.1711	-2.7059	-3.1344
11	H	-1.1799	-4.4814	-0.0078
12	C	-0.0025	-0.0027	1.9394
13	C	-0.7095	0.9771	2.6754
14	C	0.7020	-0.9834	2.6744
15	C	-0.7114	0.9687	4.0639
16	H	-1.2836	1.7299	2.1443
17	C	0.7023	-0.9767	4.0642
18	H	1.2708	-1.7402	2.1436
19	C	-0.0021	-0.0031	4.7883
20	H	-1.2807	1.7242	4.6002
21	H	1.2649	-1.7373	4.5999
22	C	-0.0003	1.2618	-0.2479
23	C	0.6497	2.4064	0.2824
24	C	-0.6547	1.3984	-1.4998
25	C	0.6568	3.6130	-0.3978
26	H	1.1759	2.3284	1.2269
27	C	-0.6623	2.6028	-2.1839
28	H	-1.1866	0.5513	-1.9182
29	C	-0.0024	3.7029	-1.6276
30	H	-1.1780	2.7074	-3.1302
31	H	1.1707	4.4788	0.0005
32	C	0.0205	0.0196	6.2960
33	H	0.7621	0.7415	6.6634
34	H	-0.9504	0.3185	6.7058
35	H	0.2824	-0.9593	6.7097
36	N	-0.0025	4.9726	-2.3457
37	O	-0.5764	5.0231	-3.4386
38	O	0.5716	5.9353	-1.8259
39	N	-0.0063	-4.9717	-2.3546
40	O	-0.5807	-5.9351	-1.8364
41	O	0.5674	-5.0204	-3.4477

SP_cnoac_rad_PMMA
Energy (POTENTIAL) = -360.0919865 Eh

	Atom	X	Y	Z
1	C	-0.8648	0.7856	-0.0044
2	H	-0.7705	1.8657	0.0263
3	C	-2.1298	0.2090	-0.0663
4	N	-3.2024	-0.2625	-0.1181
5	C	0.3367	-0.0638	0.0196
6	O	0.3249	-1.2816	-0.0128
7	O	1.4441	0.6967	0.0823
8	C	2.6904	-0.0300	0.1120
9	H	2.7279	-0.6805	0.9897
10	H	3.4684	0.7308	0.1609
11	H	2.7964	-0.6360	-0.7916

uvvisfrag1_radb3lyp
Energy (POTENTIAL) = -1345.9071691 Eh

	Atom	X	Y	Z
1	C	-0.0893	-0.0160	1.2397
2	C	-0.0201	-1.2856	0.5244
3	C	0.7034	-1.4243	-0.6879
4	C	-0.6695	-2.4517	0.9918
5	C	0.7729	-2.6261	-1.3714
6	H	1.2491	-0.5702	-1.0769
7	C	-0.6162	-3.6585	0.3037
8	H	-1.2596	-2.3986	1.9015
9	C	0.1091	-3.7817	-0.8992
10	H	1.3821	-2.6833	-2.2680
11	H	-1.1722	-4.5011	0.6987
12	C	-0.1902	0.0007	2.6998
13	C	-0.9283	0.9983	3.3815
14	C	0.4399	-0.9802	3.5032
15	C	-1.0287	1.0089	4.7684
16	H	-1.4461	1.7584	2.8046
17	C	0.3368	-0.9606	4.8898
18	H	1.0289	-1.7563	3.0242
19	C	-0.3973	0.0335	5.5555
20	H	-1.6191	1.7841	5.2526
21	H	0.8431	-1.7285	5.4712
22	C	-0.0642	1.2429	0.4984
23	C	0.5416	2.4091	1.0185

24	C	-0.6478	1.3687	-0.7877
25	C	0.5629	3.6105	0.3186
26	H	1.0122	2.3686	1.9961
27	C	-0.6264	2.5599	-1.4932
28	H	-1.1352	0.5066	-1.2324
29	C	-0.0198	3.7203	-0.9607
30	H	-1.0731	2.5900	-2.4820
31	C	-0.3426	-6.2012	-0.9911
32	C	-0.0123	-4.9702	-3.0497
33	C	0.1908	-7.4414	-1.7070
34	H	-1.4459	-6.1975	-1.0382
35	H	-0.0464	-6.2532	0.0607
36	C	0.5139	-6.2498	-3.6910
37	H	-1.0924	-4.8774	-3.2576
38	H	0.4901	-4.1128	-3.5003
39	H	-0.2955	-8.3392	-1.3148
40	H	1.2767	-7.5232	-1.5366
41	H	0.2618	-6.2711	-4.7552
42	H	1.6100	-6.2937	-3.5839
43	N	0.2246	-4.9969	-1.5976
44	O	-0.0821	-7.4033	-3.1041
45	N	0.0220	4.9057	-1.7176
46	C	-1.2378	5.3673	-2.3223
47	C	0.8034	6.0296	-1.2010
48	C	-0.9528	6.4185	-3.3895
49	H	-1.8938	5.8030	-1.5487
50	H	-1.7688	4.5306	-2.7790
51	C	1.0156	7.0626	-2.3065
52	H	0.3037	6.5103	-0.3414
53	H	1.7819	5.6703	-0.8689
54	H	-1.8900	6.8261	-3.7793
55	H	-0.3915	5.9595	-4.2197
56	O	-0.2203	7.5154	-2.8504
57	H	1.5252	7.9431	-1.9048
58	H	1.6397	6.6238	-3.1020
59	H	1.0343	4.4689	0.7832
60	C	-0.4800	0.0682	7.0623
61	H	0.3586	0.6329	7.4926
62	H	-1.4029	0.5505	7.4022
63	H	-0.4429	-0.9397	7.4898

cnoac_an
Energy (POTENTIAL) = -360.146663918 Eh

	Atom	X	Y	Z
1	C	-0.8579	0.7699	-0.0079
2	H	-0.7583	1.8491	0.0146
3	C	-2.1325	0.2088	-0.0654
4	N	-3.2150	-0.2537	-0.1141
5	C	0.2936	-0.0482	0.0237
6	O	0.3703	-1.2868	0.0051
7	O	1.4575	0.7214	0.0842
8	C	2.6720	-0.0224	0.1091
9	H	2.7191	-0.6938	0.9742
10	H	3.4757	0.7157	0.1716
11	H	2.7967	-0.6265	-0.7974

obn_an_m1_MeProp
Energy (POTENTIAL) = -501.318581156 Eh

	Atom	X	Y	Z
1	O	0.9636	-2.3763	-1.4229
2	C	1.6327	-1.4273	-0.7241
3	H	2.0001	-1.7585	0.2897
4	H	2.5807	-1.1014	-1.2325
5	C	0.8647	-0.1203	-0.4416
6	C	1.5017	0.9973	0.1171
7	C	-0.4972	-0.0169	-0.7347
8	C	0.8025	2.1752	0.3768
9	H	2.5664	0.9434	0.3471
10	C	-1.2015	1.1621	-0.4694
11	H	-0.9936	-0.8892	-1.1479
12	C	-0.5681	2.2788	0.0888
13	H	1.3234	3.0308	0.8057
14	H	-2.2645	1.2170	-0.7022
15	C	-1.3317	3.5468	0.4000
16	H	-2.2721	3.5965	-0.1599
17	H	-1.5843	3.6142	1.4674
18	H	-0.7458	4.4409	0.1545
19	O	-1.0783	-3.2874	-0.2338
20	H	-0.2167	-2.9164	-0.7112
21	C	-0.9380	-3.0004	1.1300
22	H	-0.7923	-1.9233	1.3339
23	H	-1.8478	-3.3118	1.6678
24	H	-0.0892	-3.5314	1.6067

meoh_PMMA
Energy (POTENTIAL) = -115.718795679 Eh

	Atom	X	Y	Z
1	C	0.0194	0.0016	-0.6647
2	H	-0.9917	0.0065	-1.0821
3	H	0.5486	0.8923	-1.0365
4	H	0.5404	-0.8935	-1.0376
5	O	-0.1209	0.0013	0.7522
6	H	0.7710	0.0003	1.1318

ar3m2_rad
Energy (POTENTIAL) = -1345.62485505 Eh

	Atom	X	Y	Z
1	C	-0.0893	-0.0160	1.2397
2	C	-0.0201	-1.2856	0.5244
3	C	0.7034	-1.4243	-0.6879
4	C	-0.6695	-2.4517	0.9917
5	C	0.7729	-2.6261	-1.3714
6	H	1.2491	-0.5702	-1.0769
7	C	-0.6162	-3.6585	0.3037
8	H	-1.2595	-2.3986	1.9015
9	C	0.1091	-3.7817	-0.8992
10	H	1.3821	-2.6833	-2.2680
11	H	-1.1722	-4.5011	0.6987
12	C	-0.1902	0.0007	2.6998
13	C	-0.9283	0.9983	3.3815
14	C	0.4399	-0.9802	3.5032
15	C	-1.0287	1.0089	4.7684
16	H	-1.4461	1.7584	2.8046
17	C	0.3368	-0.9606	4.8898
18	H	1.0289	-1.7563	3.0242
19	C	-0.3973	0.0335	5.5555
20	H	-1.6191	1.7841	5.2526
21	H	0.8431	-1.7285	5.4712
22	C	-0.0642	1.2429	0.4984
23	C	0.5416	2.4091	1.0185
24	C	-0.6478	1.3687	-0.7877
25	C	0.5629	3.6105	0.3186
26	H	1.0122	2.3686	1.9961
27	C	-0.6264	2.5599	-1.4932
28	H	-1.1352	0.5066	-1.2324
29	C	-0.0198	3.7203	-0.9607
30	H	-1.0731	2.5900	-2.4820
31	C	-0.3426	-6.2012	-0.9911
32	C	-0.0123	-4.9702	-3.0497
33	C	0.1908	-7.4414	-1.7070
34	H	-1.4459	-6.1975	-1.0382
35	H	-0.0464	-6.2532	0.0607
36	C	0.5139	-6.2498	-3.6910
37	H	-1.0924	-4.8774	-3.2576
38	H	0.4901	-4.1128	-3.5003
39	H	-0.2955	-8.3392	-1.3148
40	H	1.2767	-7.5232	-1.5366
41	H	0.2618	-6.2711	-4.7552
42	H	1.6100	-6.2937	-3.5839
43	N	0.2246	-4.9969	-1.5976
44	O	-0.0821	-7.4033	-3.1041
45	N	0.0220	4.9057	-1.7176
46	C	-1.2379	5.3673	-2.3223
47	C	0.8034	6.0296	-1.2010
48	C	-0.9528	6.4185	-3.3895
49	H	-1.8938	5.8030	-1.5487
50	H	-1.7688	4.5306	-2.7790
51	C	1.0156	7.0626	-2.3065
52	H	0.3037	6.5103	-0.3414
53	H	1.7819	5.6703	-0.8689
54	H	-1.8900	6.8261	-3.7793
55	H	-0.3915	5.9595	-4.2197
56	O	-0.2203	7.5154	-2.8504
57	H	1.5252	7.9431	-1.9048
58	H	1.6397	6.6238	-3.1020
59	H	1.0343	4.4689	0.7832
60	C	-0.4800	0.0682	7.0623
61	H	0.3586	0.6329	7.4926
62	H	-1.4029	0.5505	7.4022
63	H	-0.4429	-0.9397	7.4898

SP_ar3m2_cat_MeProp
Energy (POTENTIAL) = -1345.8247912 Eh

	Atom	X	Y	Z
1	C	-0.1700	0.0030	1.1707
2	C	-0.1906	-1.2388	0.4563
3	C	0.3529	-1.3709	-0.8544
4	C	-0.7510	-2.4179	1.0251
5	C	0.3366	-2.5631	-1.5369
6	H	0.8578	-0.5224	-1.3035
7	C	-0.7943	-3.6105	0.3431
8	H	-1.2193	-2.3612	2.0017

9	C	-0.2443	-3.7377	-0.9686
10	H	0.8282	-2.6066	-2.5001
11	H	-1.3022	-4.4458	0.8068
12	C	-0.1226	-0.0150	2.6372
13	C	-0.8855	0.8960	3.3998
14	C	0.6825	-0.9468	3.3270
15	C	-0.8499	0.8641	4.7879
16	H	-1.5350	1.6018	2.8921
17	C	0.7281	-0.9555	4.7156
18	H	1.2951	-1.6421	2.7622
19	C	-0.0377	-0.0546	5.4724
20	H	-1.4657	1.5579	5.3541
21	H	1.3696	-1.6699	5.2245
22	C	-0.1971	1.2626	0.4868
23	C	0.3971	2.4303	1.0464
24	C	-0.8355	1.4262	-0.7750
25	C	0.3873	3.6399	0.3953
26	H	0.9255	2.3528	1.9903
27	C	-0.8729	2.6373	-1.4252
28	H	-1.3750	0.5892	-1.2055
29	C	-0.2450	3.7957	-0.8756
30	H	-1.4423	2.7078	-2.3426
31	C	-0.6896	-6.1867	-1.0207
32	C	-0.1457	-5.0364	-3.0983
33	C	0.1980	-7.3389	-1.5025
34	H	-1.7375	-6.3836	-1.2864
35	H	-0.6086	-6.1286	0.0641
36	C	0.7293	-6.2275	-3.4847
37	H	-1.1561	-5.1848	-3.5051
38	H	0.2599	-4.1254	-3.5328
39	H	-0.1986	-8.2866	-1.1286
40	H	1.2180	-7.2013	-1.1093
41	H	0.7216	-6.3576	-4.5700
42	H	1.7653	-6.0518	-3.1536
43	N	-0.2518	-4.9268	-1.6357
44	O	0.2248	-7.4307	-2.9195
45	N	-0.2410	4.9890	-1.5372
46	C	-0.7700	5.1419	-2.8988
47	C	-0.0669	6.2841	-0.8613
48	C	0.0721	6.1568	-3.6779
49	H	-1.8162	5.4761	-2.8553
50	H	-0.7276	4.1913	-3.4297
51	C	0.7541	7.2375	-1.7271
52	H	-1.0614	6.7195	-0.6879
53	H	0.4182	6.1603	0.1044
54	H	-0.3923	6.3475	-4.6491
55	H	1.0803	5.7434	-3.8420
56	O	0.1557	7.4047	-3.0057
57	H	0.7888	8.2250	-1.2596
58	H	1.7825	6.8559	-1.8306
59	H	0.9121	4.4688	0.8520
60	C	0.0291	-0.0535	6.9781
61	H	0.7834	0.6635	7.3284
62	H	-0.9282	0.2403	7.4205
63	H	0.3061	-1.0373	7.3692

SP_ar3no2obn_meoh_MeButa
Energy (POTENTIAL) = -1683.1433136 Eh

	Atom	X	Y	Z
1	C	-0.3264	-0.3957	-0.2730
2	C	-1.7018	-0.2602	0.4012
3	C	-2.6548	0.5741	-0.2096
4	C	-1.9931	-0.8182	1.6520
5	C	-3.8800	0.8205	0.3969
6	H	-2.4256	1.0543	-1.1544
7	C	-3.2193	-0.5859	2.2720
8	H	-1.2645	-1.4376	2.1612
9	C	-4.1493	0.2266	1.6305
10	H	-4.6172	1.4624	-0.0684
11	H	-3.4511	-1.0193	3.2366
12	C	0.5679	-1.5120	0.2974
13	C	1.9181	-1.2655	0.5783
14	C	0.0742	-2.8201	0.4315
15	C	2.7534	-2.2879	1.0195
16	H	2.3224	-0.2706	0.4397
17	C	0.8933	-3.8525	0.8704
18	H	-0.9600	-3.0361	0.1858
19	C	2.2275	-3.5695	1.1662
20	H	3.7961	-2.1016	1.2441
21	H	0.5161	-4.8613	0.9808
22	C	-0.4325	-0.6778	-1.7816
23	C	0.5730	-0.2236	-2.6439
24	C	-1.4617	-1.4645	-2.3098
25	C	0.5289	-0.5255	-4.0036
26	H	1.3905	0.3703	-2.2503
27	C	-1.4954	-1.7686	-3.6705

28	H	-2.2506	-1.8383	-1.6643
29	C	-0.5068	-1.2995	-4.5444
30	H	1.3176	-0.1561	-4.6550
31	H	-2.3085	-2.3776	-4.0580
32	O	0.3474	0.8781	-0.1692
33	C	0.3964	1.5038	1.1405
34	H	-0.5695	1.9825	1.3360
35	H	0.5635	0.7437	1.9107
36	C	1.5174	2.5054	1.1155
37	C	2.7285	2.2470	1.7650
38	C	1.3755	3.7062	0.4051
39	C	3.7772	3.1672	1.7064
40	H	2.8546	1.3225	2.3240
41	C	2.4266	4.6177	0.3445
42	H	0.4424	3.9123	-0.1123
43	C	3.6446	4.3663	0.9959
44	H	4.7107	2.9491	2.2196
45	H	2.3017	5.5425	-0.2145
46	C	4.7664	5.3766	0.9492
47	H	4.8571	5.8265	-0.0457
48	H	4.5872	6.1955	1.6583
49	H	5.7281	4.9221	1.2079
50	C	-0.5667	-1.5948	-6.0238
51	H	-1.0779	-0.7868	-6.5636
52	H	0.4362	-1.6884	-6.4541
53	H	-1.1146	-2.5207	-6.2264
54	C	-0.1378	3.2285	-2.9368
55	H	-0.6962	4.0173	-3.4498
56	H	0.8377	3.6380	-2.6323
57	H	0.0356	2.4059	-3.6449
58	O	-0.9137	2.8164	-1.8179
59	H	-0.4091	2.1232	-1.3481
60	N	3.0992	-4.6507	1.6350
61	O	4.2758	-4.3801	1.8862
62	O	2.6123	-5.7771	1.7562
63	N	-5.4447	0.4756	2.2762
64	O	-5.6602	-0.0565	3.3670
65	O	-6.2506	1.2040	1.6942

SP_obn_an_m2c_PMMA
Energy (POTENTIAL) = -617.2764204 Eh

	Atom	X	Y	Z
1	O	0.8155	-1.3015	-1.2836
2	C	1.4998	-0.3596	-0.4976
3	H	1.7137	-0.7580	0.5129
4	H	2.4798	-0.1691	-0.9619
5	C	0.7745	0.9715	-0.3304
6	C	1.4765	2.1800	-0.2923
7	C	-0.6202	1.0068	-0.1770
8	C	0.8121	3.3947	-0.0990
9	H	2.5580	2.1774	-0.4227
10	C	-1.2781	2.2209	0.0138
11	H	-1.1731	0.0687	-0.2042
12	C	-0.5776	3.4362	0.0594
13	H	1.3811	4.3229	-0.0806
14	H	-2.3611	2.2288	0.1297
15	C	-1.3054	4.7412	0.2908
16	H	-2.2216	4.8019	-0.3088
17	H	-1.6041	4.8519	1.3423
18	H	-0.6783	5.6028	0.0362
19	O	-1.1605	-2.1353	0.1377
20	H	0.0204	-1.6549	-0.7236
21	C	-0.8102	-2.0961	1.4774
22	H	-0.5856	-1.0658	1.8329
23	H	-1.6160	-2.4736	2.1466
24	H	0.0901	-2.7018	1.7310
25	O	-0.8197	-4.6451	-0.4505
26	H	-1.0517	-3.6756	-0.2411
27	C	0.5874	-4.7031	-0.4347
28	H	0.9999	-4.8871	0.5780
29	H	0.9301	-5.5321	-1.0739
30	H	1.0464	-3.7744	-0.8051

ar3m2obn_MeProp
Energy (POTENTIAL) = -1731.15593330 Eh

	Atom	X	Y	Z
1	C	-0.1381	0.2977	-0.8994
2	C	-1.4377	-0.3577	-0.4145
3	C	-2.6216	-0.1376	-1.1390
4	C	-1.5529	-1.0276	0.8053
5	C	-3.8500	-0.5925	-0.6839
6	H	-2.5778	0.4166	-2.0717
7	C	-2.7813	-1.5056	1.2683
8	H	-0.6715	-1.1960	1.4150
9	C	-3.9609	-1.3013	0.5323
10	H	-4.7415	-0.3725	-1.2629

11	H	-2.8022	-2.0478	2.2064
12	C	1.1486	-0.2836	-0.2796
13	C	2.2104	0.5647	0.0648
14	C	1.3615	-1.6602	-0.1568
15	C	3.4175	0.0623	0.5359
16	H	2.0917	1.6360	-0.0551
17	C	2.5653	-2.1759	0.3251
18	H	0.5720	-2.3550	-0.4268
19	C	3.6242	-1.3232	0.6856
20	H	4.2229	0.7544	0.7624
21	H	2.6626	-3.2505	0.4274
22	C	0.0233	0.1883	-2.4268
23	C	0.4969	1.2657	-3.1816
24	C	-0.2282	-1.0229	-3.0856
25	C	0.7047	1.1345	-4.5565
26	H	0.7026	2.2097	-2.6911
27	C	-0.0174	-1.1490	-4.4567
28	H	-0.6005	-1.8739	-2.5229
29	C	0.4512	-0.0707	-5.2210
30	H	1.0726	1.9886	-5.1213
31	H	-0.2224	-2.1007	-4.9424
32	N	4.8729	-1.8018	1.1406
33	C	5.3801	-1.2310	2.3981
34	C	5.1113	-3.2435	1.0876
35	C	6.8551	-1.5779	2.5702
36	H	4.8113	-1.6273	3.2579
37	H	5.2656	-0.1453	2.3926
38	C	6.5968	-3.5270	1.3054
39	H	4.5226	-3.7840	1.8508
40	H	4.8222	-3.6233	0.1026
41	H	7.2175	-1.2291	3.5417
42	H	7.4432	-1.0912	1.7749
43	O	7.0603	-2.9872	2.5387
44	H	6.7731	-4.6058	1.3486
45	H	7.1756	-3.1061	0.4671
46	N	-5.2268	-1.7291	0.9819
47	C	-6.0545	-2.4825	0.0266
48	C	-5.3177	-2.3044	2.3234
49	C	-7.4924	-2.5608	0.5290
50	H	-5.6584	-3.5052	-0.1004
51	H	-6.0432	-1.9952	-0.9499
52	C	-6.7826	-2.3958	2.7487
53	H	-4.8605	-3.3091	2.3669
54	H	-4.7891	-1.6587	3.0315
55	H	-8.0923	-3.1904	-0.1345
56	H	-7.9305	-1.5495	0.5528
57	O	-7.5548	-3.1525	1.8230
58	H	-6.8631	-2.9029	3.7146
59	H	-7.1985	-1.3796	2.8460
60	O	-0.2131	1.7143	-0.6314
61	C	-0.5325	2.0928	0.7143
62	H	-1.6121	1.9791	0.8796
63	H	-0.0162	1.4458	1.4339
64	C	-0.1019	3.5249	0.8892
65	C	1.0313	3.8542	1.6392
66	C	-0.8103	4.5573	0.2603
67	C	1.4420	5.1830	1.7658
68	H	1.6004	3.0644	2.1244
69	C	-0.3965	5.8816	0.3833
70	H	-1.6879	4.3154	-0.3344
71	C	0.7355	6.2184	1.1422
72	H	2.3254	5.4177	2.3555
73	H	-0.9585	6.6692	-0.1141
74	C	1.1588	7.6603	1.2988
75	H	1.0257	8.2198	0.3661
76	H	0.5591	8.1665	2.0670
77	H	2.2091	7.7405	1.5976
78	C	0.6476	-0.2033	-6.7131
79	H	-0.3010	-0.0734	-7.2512
80	H	1.3451	0.5499	-7.0943
81	H	1.0357	-1.1929	-6.9796

SP_meoh_MeButa
Energy (POTENTIAL) = -115.770379 Eh

	Atom	X	Y	Z
1	C	0.0191	0.0016	-0.6648
2	H	-0.9918	0.0065	-1.0826
3	H	0.5486	0.8921	-1.0362
4	H	0.5404	-0.8933	-1.0372
5	O	-0.1209	0.0013	0.7525
6	H	0.7714	0.0003	1.1315

SP_ar3no2obn_meoh
Energy (POTENTIAL) = -1683.1479476 Eh

	Atom	X	Y	Z
1	C	-0.3288	-0.3989	-0.2724

2	C	-1.7039	-0.2619	0.4019
3	C	-2.6582	0.5690	-0.2118
4	C	-1.9929	-0.8137	1.6559
5	C	-3.8826	0.8180	0.3946
6	H	-2.4311	1.0425	-1.1604
7	C	-3.2181	-0.5786	2.2764
8	H	-1.2637	-1.4301	2.1676
9	C	-4.1495	0.2303	1.6321
10	H	-4.6208	1.4562	-0.0741
11	H	-3.4468	-1.0077	3.2436
12	C	0.5643	-1.5164	0.2970
13	C	1.9167	-1.2729	0.5703
14	C	0.0669	-2.8226	0.4365
15	C	2.7513	-2.2964	1.0095
16	H	2.3235	-0.2796	0.4288
17	C	0.8853	-3.8563	0.8734
18	H	-0.9693	-3.0369	0.1981
19	C	2.2222	-3.5764	1.1615
20	H	3.7952	-2.1108	1.2288
21	H	0.5042	-4.8629	0.9884
22	C	-0.4344	-0.6806	-1.7811
23	C	0.5713	-0.2263	-2.6433
24	C	-1.4634	-1.4682	-2.3093
25	C	0.5275	-0.5280	-4.0033
26	H	1.3888	0.3678	-2.2501
27	C	-1.4967	-1.7721	-3.6703
28	H	-2.2518	-1.8433	-1.6640
29	C	-0.5082	-1.3024	-4.5443
30	H	1.3161	-0.1581	-4.6543
31	H	-2.3092	-2.3820	-4.0575
32	O	0.3448	0.8755	-0.1673
33	C	0.3988	1.4956	1.1455
34	H	-0.5681	1.9684	1.3495
35	H	0.5749	0.7331	1.9109
36	C	1.5141	2.5034	1.1173
37	C	2.7340	2.2459	1.7509
38	C	1.3583	3.7098	0.4193
39	C	3.7771	3.1724	1.6885
40	H	2.8716	1.3172	2.3000
41	C	2.4038	4.6279	0.3542
42	H	0.4187	3.9154	-0.0864
43	C	3.6305	4.3772	0.9899
44	H	4.7176	2.9545	2.1888
45	H	2.2678	5.5564	-0.1957
46	C	4.7467	5.3934	0.9390
47	H	4.8230	5.8527	-0.0528
48	H	4.5716	6.2051	1.6573
49	H	5.7133	4.9413	1.1826
50	C	-0.5678	-1.5978	-6.0236
51	H	-1.0789	-0.7896	-6.5633
52	H	0.4351	-1.6909	-6.4537
53	H	-1.1161	-2.5236	-6.2260
54	C	-0.0861	3.2330	-2.9213
55	H	-0.6298	4.0215	-3.4505
56	H	0.8746	3.6459	-2.5785
57	H	0.1183	2.4155	-3.6269
58	O	-0.9006	2.8093	-1.8334
59	H	-0.4065	2.1197	-1.3469
60	N	3.0923	-4.6581	1.6271
61	O	4.2723	-4.3928	1.8708
62	O	2.6043	-5.7840	1.7544
63	N	-5.4427	0.4825	2.2775
64	O	-5.6592	-0.0437	3.3715
65	O	-6.2506	1.2089	1.6945

obn_an_MeButa
Energy (POTENTIAL) = -385.559818597 Eh

	Atom	X	Y	Z
1	O	-1.0838	-3.1837	0.2102
2	C	0.0840	-2.5748	0.3629
3	H	0.6111	-2.7349	1.3681
4	H	0.9170	-2.8878	-0.3596
5	C	0.0732	-1.0270	0.2240
6	C	1.2236	-0.2401	0.3791
7	C	-1.1249	-0.3763	-0.0693
8	C	1.1748	1.1471	0.2478
9	H	2.1738	-0.7270	0.6057
10	C	-1.1804	1.0154	-0.2029
11	H	-2.0005	-1.0105	-0.1869
12	C	-0.0341	1.8030	-0.0448
13	H	2.0831	1.7370	0.3722
14	H	-2.1289	1.5009	-0.4336
15	C	-0.0841	3.3100	-0.1677
16	H	-1.0500	3.6481	-0.5594
17	H	0.0660	3.8024	0.8032
18	H	0.6988	3.6878	-0.8379

SP_ar3m2obn_PMMA
Energy (POTENTIAL) = -1731.6021951 Eh

	Atom	X	Y	Z
1	C	-0.1406	0.2982	-0.9006
2	C	-1.4398	-0.3576	-0.4148
3	C	-2.6237	-0.1391	-1.1397
4	C	-1.5542	-1.0260	0.8059
5	C	-3.8516	-0.5945	-0.6838
6	H	-2.5798	0.4143	-2.0727
7	C	-2.7823	-1.5044	1.2694
8	H	-0.6725	-1.1927	1.4155
9	C	-3.9615	-1.3020	0.5329
10	H	-4.7439	-0.3758	-1.2621
11	H	-2.8032	-2.0455	2.2083
12	C	1.1465	-0.2824	-0.2809
13	C	2.2079	0.5667	0.0625
14	C	1.3595	-1.6587	-0.1569
15	C	3.4152	0.0649	0.5335
16	H	2.0887	1.6378	-0.0590
17	C	2.5635	-2.1736	0.3253
18	H	0.5700	-2.3537	-0.4263
19	C	3.6219	-1.3203	0.6844
20	H	4.2213	0.7567	0.7582
21	H	2.6615	-3.2481	0.4288
22	C	0.0198	0.1887	-2.4279
23	C	0.4906	1.2672	-3.1828
24	C	-0.2303	-1.0227	-3.0863
25	C	0.6972	1.1364	-4.5577
26	H	0.6947	2.2114	-2.6920
27	C	-0.0207	-1.1482	-4.4576
28	H	-0.6008	-1.8742	-2.5232
29	C	0.4453	-0.0691	-5.2219
30	H	1.0631	1.9912	-5.1228
31	H	-0.2245	-2.1003	-4.9433
32	N	4.8711	-1.7979	1.1403
33	C	5.3735	-1.2314	2.4013
34	C	5.1132	-3.2384	1.0816
35	C	6.8493	-1.5750	2.5750
36	H	4.8042	-1.6330	3.2584
37	H	5.2552	-0.1460	2.3999
38	C	6.5992	-3.5187	1.3011
39	H	4.5251	-3.7840	1.8417
40	H	4.8262	-3.6142	0.0944
41	H	7.2089	-1.2302	3.5490
42	H	7.4375	-1.0825	1.7831
43	O	7.0584	-2.9831	2.5372
44	H	6.7788	-4.5971	1.3404
45	H	7.1781	-3.0925	0.4654
46	N	-5.2276	-1.7313	0.9818
47	C	-6.0477	-2.4967	0.0299
48	C	-5.3209	-2.2958	2.3273
49	C	-7.4873	-2.5768	0.5273
50	H	-5.6469	-3.5190	-0.0864
51	H	-6.0333	-2.0178	-0.9509
52	C	-6.7872	-2.3891	2.7477
53	H	-4.8609	-3.2989	2.3811
54	H	-4.7965	-1.6423	3.0314
55	H	-8.0822	-3.2153	-0.1325
56	H	-7.9299	-1.5671	0.5397
57	O	-7.5524	-3.1568	1.8259
58	H	-6.8703	-2.8882	3.7176
59	H	-7.2070	-1.3734	2.8343
60	O	-0.2160	1.7144	-0.6327
61	C	-0.5333	2.0932	0.7129
62	H	-1.6132	1.9824	0.8788
63	H	-0.0187	1.4446	1.4324
64	C	-0.0981	3.5239	0.8884
65	C	1.0241	3.8511	1.6550
66	C	-0.7918	4.5566	0.2441
67	C	1.4384	5.1787	1.7835
68	H	1.5825	3.0606	2.1513
69	C	-0.3743	5.8795	0.3689
70	H	-1.6599	4.3156	-0.3647
71	C	0.7464	6.2145	1.1449
72	H	2.3133	5.4119	2.3861
73	H	-0.9246	6.6675	-0.1409
74	C	1.1730	7.6553	1.3032
75	H	1.0540	8.2128	0.3674
76	H	0.5657	8.1658	2.0625
77	H	2.2198	7.7325	1.6148
78	C	0.6406	-0.2011	-6.7143
79	H	-0.3080	-0.0692	-7.2521
80	H	1.3391	0.5513	-7.0954
81	H	1.0274	-1.1909	-6.9818

ar3no2obn_meoh_MeButa
Energy (POTENTIAL) = -1682.68316183 Eh

	Atom	X	Y	Z
1	C	-0.3264	-0.3957	-0.2730
2	C	-1.7018	-0.2602	0.4012
3	C	-2.6548	0.5741	-0.2096
4	C	-1.9931	-0.8182	1.6520
5	C	-3.8800	0.8205	0.3969
6	H	-2.4256	1.0543	-1.1544
7	C	-3.2193	-0.5859	2.2720
8	H	-1.2645	-1.4376	2.1612
9	C	-4.1493	0.2266	1.6305
10	H	-4.6172	1.4624	-0.0684
11	H	-3.4511	-1.0193	3.2366
12	C	0.5679	-1.5120	0.2974
13	C	1.9181	-1.2655	0.5783
14	C	0.0742	-2.8201	0.4315
15	C	2.7534	-2.2879	1.0195
16	H	2.3224	-0.2706	0.4397
17	C	0.8933	-3.8525	0.8704
18	H	-0.9600	-3.0361	0.1858
19	C	2.2275	-3.5695	1.1662
20	H	3.7961	-2.1016	1.2441
21	H	0.5161	-4.8613	0.9808
22	C	-0.4325	-0.6778	-1.7816
23	C	0.5730	-0.2236	-2.6439
24	C	-1.4617	-1.4645	-2.3098
25	C	0.5289	-0.5255	-4.0036
26	H	1.3905	0.3703	-2.2503
27	C	-1.4954	-1.7686	-3.6705
28	H	-2.2506	-1.8383	-1.6643
29	C	-0.5068	-1.2995	-4.5444
30	H	1.3176	-0.1561	-4.6550
31	H	-2.3085	-2.3776	-4.0580
32	O	0.3474	0.8781	-0.1692
33	C	0.3964	1.5038	1.1405
34	H	-0.5695	1.9825	1.3360
35	H	0.5635	0.7437	1.9107
36	C	1.5174	2.5054	1.1155
37	C	2.7285	2.2470	1.7650
38	C	1.3755	3.7062	0.4051
39	C	3.7772	3.1672	1.7064
40	H	2.8546	1.3225	2.3240
41	C	2.4266	4.6177	0.3445
42	H	0.4424	3.9123	-0.1123
43	C	3.6446	4.3663	0.9959
44	H	4.7107	2.9491	2.2196
45	H	2.3017	5.5425	-0.2145
46	C	4.7664	5.3766	0.9492
47	H	4.8571	5.8265	-0.0457
48	H	4.5872	6.1955	1.6583
49	H	5.7281	4.9221	1.2079
50	C	-0.5667	-1.5948	-6.0238
51	H	-1.0779	-0.7868	-6.5636
52	H	0.4362	-1.6884	-6.4541
53	H	-1.1146	-2.5207	-6.2264
54	C	-0.1378	3.2285	-2.9368
55	H	-0.6962	4.0173	-3.4498
56	H	0.8377	3.6380	-2.6323
57	H	0.0356	2.4059	-3.6449
58	O	-0.9137	2.8164	-1.8179
59	H	-0.4091	2.1232	-1.3481
60	N	3.0992	-4.6507	1.6350
61	O	4.2758	-4.3801	1.8862
62	O	2.6123	-5.7771	1.7562
63	N	-5.4447	0.4756	2.2762
64	O	-5.6602	-0.0565	3.3671
65	O	-6.2506	1.2040	1.6942

obn_an_m2b_MeProp
Energy (POTENTIAL) = -617.071674752 Eh

	Atom	X	Y	Z
1	O	0.0781	-1.4549	-1.0836
2	C	1.1137	-0.7193	-0.5600
3	H	1.5053	-1.1207	0.4072
4	H	2.0116	-0.7216	-1.2254
5	C	0.7837	0.7544	-0.3015
6	C	1.7391	1.6278	0.2385
7	C	-0.4763	1.2721	-0.6085
8	C	1.4432	2.9710	0.4651
9	H	2.7312	1.2480	0.4827
10	C	-0.7749	2.6189	-0.3800
11	H	-1.2193	0.5941	-1.0149
12	C	0.1755	3.4926	0.1605
13	H	2.2039	3.6283	0.8839
14	H	-1.7656	2.9980	-0.6270

15	C	-0.1548	4.9420	0.4380
16	H	-1.0005	5.2839	-0.1689
17	H	-0.4267	5.0980	1.4912
18	H	0.6988	5.5973	0.2277
19	O	-2.0446	-1.6900	0.3965
20	H	-1.1859	-1.5234	-0.1287
21	C	-2.6920	-2.7431	-0.2833
22	H	-2.0030	-3.5688	-0.5182
23	H	-3.4980	-3.1387	0.3515
24	H	-3.1566	-2.4197	-1.2345
25	O	0.5564	-3.9496	-0.5130
26	H	0.3951	-2.9895	-0.8151
27	C	0.5454	-3.9070	0.8975
28	H	-0.2830	-3.2975	1.2877
29	H	0.4318	-4.9292	1.2872
30	H	1.4835	-3.4997	1.3215

obn_an
Energy (POTENTIAL) = -385.574981019 Eh

	Atom	X	Y	Z
1	O	-1.0798	-3.1912	0.2112
2	C	0.0882	-2.5735	0.3633
3	H	0.6148	-2.7353	1.3661
4	H	0.9183	-2.8859	-0.3597
5	C	0.0708	-1.0272	0.2254
6	C	1.2238	-0.2417	0.3717
7	C	-1.1286	-0.3744	-0.0587
8	C	1.1758	1.1454	0.2383
9	H	2.1754	-0.7285	0.5910
10	C	-1.1831	1.0174	-0.1944
11	H	-2.0095	-1.0021	-0.1708
12	C	-0.0341	1.8034	-0.0465
13	H	2.0860	1.7333	0.3542
14	H	-2.1323	1.5032	-0.4197
15	C	-0.0837	3.3103	-0.1684
16	H	-1.0260	3.6456	-0.6154
17	H	0.0057	3.7987	0.8117
18	H	0.7371	3.6920	-0.7882

obn_rad_MeButa
Energy (POTENTIAL) = -385.438273659 Eh

	Atom	X	Y	Z
1	O	0.0687	-2.9249	-0.8990
2	C	0.1192	-2.5520	0.4298
3	H	-0.7457	-2.9123	1.0100
4	H	1.0528	-2.8608	0.9272
5	C	0.0696	-1.0300	0.2991
6	C	1.2510	-0.2973	0.1273
7	C	-1.1623	-0.3656	0.2426
8	C	1.2007	1.0851	-0.0336
9	H	2.2083	-0.8117	0.1323
10	C	-1.2053	1.0170	0.0814
11	H	-2.0843	-0.9329	0.3374
12	C	-0.0263	1.7660	-0.0555
13	H	2.1252	1.6457	-0.1488
14	H	-2.1666	1.5245	0.0564
15	C	-0.0759	3.2675	-0.1995
16	H	-0.9944	3.5922	-0.6993
17	H	-0.0507	3.7546	0.7845
18	H	0.7788	3.6442	-0.7709

SP_ar3m2cnoac_MeButa
Energy (POTENTIAL) = -1706.1285571 Eh

	Atom	X	Y	Z
1	C	-1.0510	0.0049	-0.4260
2	C	-0.2607	1.3299	-0.3329
3	C	-0.1649	2.0424	0.8667
4	C	0.6006	3.2047	0.9768
5	C	1.3258	3.7019	-0.1195
6	C	1.2531	2.9699	-1.3223
7	C	0.4843	1.8180	-1.4200
8	H	0.4999	1.2800	-2.3624
9	H	1.8322	3.2874	-2.1835
10	H	0.6090	3.7254	1.9271
11	H	-0.7032	1.6971	1.7424
12	C	-2.1582	-0.0290	0.6531
13	C	-2.2605	-1.0471	1.6039
14	C	-3.2987	-1.0547	2.5399
15	C	-4.2692	-0.0494	2.5608
16	C	-4.1602	0.9795	1.6125
17	C	-3.1233	0.9939	0.6854
18	H	-3.0618	1.8126	-0.0247
19	H	-4.8946	1.7818	1.6044
20	H	-3.3523	-1.8650	3.2632
21	H	-1.5340	-1.8506	1.6158
22	C	-1.8102	-0.0023	-1.8549

23	H	-1.9966	1.0421	-2.1152
24	C	-1.0244	-0.5730	-2.9512
25	N	-0.4213	-0.9988	-3.8471
26	C	-3.1887	-0.6905	-1.8327
27	O	-3.3912	-1.8713	-1.6536
28	O	-4.1496	0.2167	-2.0450
29	C	-5.5048	-0.2691	-1.9201
30	H	-5.6795	-1.0942	-2.6139
31	H	-6.1396	0.5826	-2.1607
32	H	-5.6778	-0.6037	-0.8940
33	C	-0.0807	-1.1876	-0.2904
34	C	1.1816	-1.0346	0.3038
35	C	2.0451	-2.1104	0.4760
36	C	1.6870	-3.4102	0.0695
37	C	0.4184	-3.5691	-0.5169
38	C	-0.4370	-2.4824	-0.6913
39	H	-1.4130	-2.6630	-1.1262
40	H	0.0736	-4.5473	-0.8315
41	H	3.0252	-1.9285	0.9054
42	H	1.5135	-0.0553	0.6281
43	N	2.1373	4.8521	-0.0442
44	C	1.9380	5.8736	-1.0847
45	C	2.3270	5.4688	1.2685
46	C	3.0959	6.8663	-1.0714
47	H	0.9924	6.4169	-0.9120
48	H	1.8800	5.4043	-2.0685
49	C	3.4714	6.4793	1.2023
50	H	1.4103	5.9782	1.6158
51	H	2.5850	4.6955	1.9987
52	H	2.9098	7.6757	-1.7834
53	H	4.0279	6.3503	-1.3546
54	O	3.2401	7.4723	0.2093
55	H	3.5654	7.0048	2.1570
56	H	4.4152	5.9490	0.9950
57	N	2.5976	-4.4760	0.2205
58	C	3.2092	-4.6479	1.5473
59	C	2.2442	-5.7674	-0.3676
60	C	4.4043	-5.5911	1.4540
61	H	2.4733	-5.0638	2.2578
62	H	3.5437	-3.6849	1.9372
63	C	3.4723	-6.6759	-0.3933
64	H	1.4346	-6.2644	0.1965
65	H	1.9001	-5.6157	-1.3952
66	H	4.8095	-5.7901	2.4506
67	H	5.1912	-5.1291	0.8354
68	O	4.0265	-6.8500	0.9065
69	H	3.1973	-7.6713	-0.7541
70	H	4.2282	-6.2488	-1.0723
71	C	-5.3837	-0.0476	3.5797
72	H	-5.4937	-1.0269	4.0562
73	H	-5.1920	0.6868	4.3731
74	H	-6.3429	0.2184	3.1212

ar3m2_cat_PMMA
Energy (POTENTIAL) = -1345.48109008 Eh

	Atom	X	Y	Z
1	C	-0.1704	0.0031	1.1717
2	C	-0.1908	-1.2387	0.4569
3	C	0.3530	-1.3707	-0.8536
4	C	-0.7513	-2.4179	1.0253
5	C	0.3369	-2.5628	-1.5362
6	H	0.8580	-0.5220	-1.3024
7	C	-0.7942	-3.6104	0.3432
8	H	-1.2199	-2.3611	2.0018
9	C	-0.2438	-3.7378	-0.9683
10	H	0.8290	-2.6065	-2.4992
11	H	-1.3023	-4.4457	0.8067
12	C	-0.1229	-0.0150	2.6379
13	C	-0.8856	0.8961	3.4007
14	C	0.6819	-0.9472	3.3276
15	C	-0.8502	0.8638	4.7886
16	H	-1.5351	1.6020	2.8930
17	C	0.7275	-0.9560	4.7161
18	H	1.2947	-1.6422	2.7626
19	C	-0.0383	-0.0552	5.4730
20	H	-1.4661	1.5573	5.3550
21	H	1.3691	-1.6703	5.2250
22	C	-0.1974	1.2628	0.4876
23	C	0.3979	2.4302	1.0468
24	C	-0.8365	1.4268	-0.7737
25	C	0.3890	3.6396	0.3953
26	H	0.9265	2.3522	1.9906
27	C	-0.8728	2.6379	-1.4242
28	H	-1.3771	0.5901	-1.2034
29	C	-0.2431	3.7957	-0.8756
30	H	-1.4432	2.7093	-2.3409

31	C	-0.6886	-6.1869	-1.0202
32	C	-0.1452	-5.0364	-3.0982
33	C	0.1973	-7.3400	-1.5039
34	H	-1.7370	-6.3831	-1.2846
35	H	-0.6061	-6.1289	0.0646
36	C	0.7281	-6.2288	-3.4853
37	H	-1.1560	-5.1837	-3.5047
38	H	0.2610	-4.1254	-3.5323
39	H	-0.1997	-8.2876	-1.1303
40	H	1.2179	-7.2037	-1.1114
41	H	0.7197	-6.3590	-4.5707
42	H	1.7647	-6.0543	-3.1550
43	N	-0.2508	-4.9269	-1.6354
44	O	0.2220	-7.4304	-2.9202
45	N	-0.2369	4.9885	-1.5383
46	C	-0.7663	5.1398	-2.9001
47	C	-0.0650	6.2840	-0.8620
48	C	0.0712	6.1591	-3.6790
49	H	-1.8141	5.4692	-2.8569
50	H	-0.7190	4.1892	-3.4309
51	C	0.7507	7.2410	-1.7292
52	H	-1.0603	6.7163	-0.6858
53	H	0.4231	6.1604	0.1023
54	H	-0.3946	6.3487	-4.6497
55	H	1.0810	5.7500	-3.8442
56	O	0.1492	7.4060	-3.0057
57	H	0.7824	8.2286	-1.2616
58	H	1.7806	6.8637	-1.8345
59	H	0.9147	4.4682	0.8516
60	C	0.0283	-0.0545	6.9786
61	H	0.7824	0.6624	7.3292
62	H	-0.9290	0.2390	7.4211
63	H	0.3052	-1.0384	7.3697

ar3m2cnoac_MeProp
Energy (POTENTIAL) = -1705.67679336 Eh

	Atom	X	Y	Z
1	C	-1.0508	0.0044	-0.4262
2	C	-0.2607	1.3296	-0.3331
3	C	-0.1635	2.0407	0.8671
4	C	0.6017	3.2031	0.9776
5	C	1.3252	3.7020	-0.1192
6	C	1.2510	2.9714	-1.3227
7	C	0.4824	1.8192	-1.4207
8	H	0.4967	1.2827	-2.3639
9	H	1.8285	3.2901	-2.1845
10	H	0.6112	3.7227	1.9285
11	H	-0.7006	1.6943	1.7432
12	C	-2.1582	-0.0291	0.6527
13	C	-2.2600	-1.0460	1.6048
14	C	-3.2983	-1.0529	2.5408
15	C	-4.2692	-0.0480	2.5604
16	C	-4.1606	0.9798	1.6110
17	C	-3.1236	0.9936	0.6839
18	H	-3.0624	1.8116	-0.0271
19	H	-4.8953	1.7818	1.6018
20	H	-3.3515	-1.8623	3.2652
21	H	-1.5330	-1.8491	1.6180
22	C	-1.8094	-0.0036	-1.8555
23	H	-1.9950	1.0406	-2.1171
24	C	-1.0232	-0.5761	-2.9504
25	N	-0.4195	-1.0036	-3.8451
26	C	-3.1884	-0.6909	-1.8335
27	O	-3.3915	-1.8717	-1.6539
28	O	-4.1485	0.2166	-2.0467
29	C	-5.5043	-0.2680	-1.9225
30	H	-5.6790	-1.0936	-2.6157
31	H	-6.1382	0.5839	-2.1645
32	H	-5.6785	-0.6013	-0.8962
33	C	-0.0804	-1.1881	-0.2902
34	C	1.1834	-1.0344	0.3004
35	C	2.0470	-2.1104	0.4723
36	C	1.6875	-3.4108	0.0692
37	C	0.4172	-3.5703	-0.5136
38	C	-0.4382	-2.4835	-0.6877
39	H	-1.4154	-2.6644	-1.1198
40	H	0.0711	-4.5489	-0.8253
41	H	3.0283	-1.9279	0.8990
42	H	1.5167	-0.0547	0.6221
43	N	2.1364	4.8523	-0.0434
44	C	1.9368	5.8743	-1.0834
45	C	2.3264	5.4683	1.2695
46	C	3.0945	6.8672	-1.0698
47	H	0.9912	6.4172	-0.9103
48	H	1.8789	5.4057	-2.0675
49	C	3.4704	6.4791	1.2037

50	H	1.4096	5.9771	1.6174
51	H	2.5848	4.6948	1.9993
52	H	2.9081	7.6768	-1.7814
53	H	4.0266	6.3516	-1.3533
54	O	3.2388	7.4727	0.2113
55	H	3.5644	7.0041	2.1588
56	H	4.4144	5.9492	0.9959
57	N	2.5979	-4.4767	0.2198
58	C	3.2143	-4.6454	1.5448
59	C	2.2414	-5.7696	-0.3634
60	C	4.4082	-5.5899	1.4500
61	H	2.4805	-5.0585	2.2592
62	H	3.5512	-3.6817	1.9308
63	C	3.4686	-6.6793	-0.3909
64	H	1.4333	-6.2641	0.2049
65	H	1.8939	-5.6207	-1.3902
66	H	4.8168	-5.7863	2.4457
67	H	5.1933	-5.1305	0.8272
68	O	4.0272	-6.8502	0.9075
69	H	3.1913	-7.6754	-0.7479
70	H	4.2225	-6.2548	-1.0738
71	C	-5.3841	-0.0456	3.5790
72	H	-5.4912	-1.0234	4.0594
73	H	-5.1950	0.6926	4.3693
74	H	-6.3440	0.2154	3.1189

cnoac_rad_MeButa
Energy (POTENTIAL) = -359.984202811 Eh

	Atom	X	Y	Z
1	C	-0.8647	0.7875	-0.0045
2	H	-0.7715	1.8676	0.0261
3	C	-2.1291	0.2090	-0.0663
4	N	-3.2002	-0.2658	-0.1178
5	C	0.3365	-0.0625	0.0193
6	O	0.3227	-1.2807	-0.0132
7	O	1.4440	0.6962	0.0821
8	C	2.6906	-0.0308	0.1121
9	H	2.7278	-0.6804	0.9905
10	H	3.4685	0.7301	0.1607
11	H	2.7964	-0.6368	-0.7914

SP_ar3no2cnoac_MeButa
Energy (POTENTIAL) = -1541.8801529 Eh

	Atom	X	Y	Z
1	C	-0.3314	0.0144	-0.3432
2	C	0.2540	1.4426	-0.2051
3	C	0.0625	2.1905	0.9666
4	C	0.6312	3.4523	1.1187
5	C	1.4028	3.9704	0.0825
6	C	1.6287	3.2509	-1.0893
7	C	1.0585	1.9895	-1.2197
8	H	1.2663	1.4284	-2.1233
9	H	2.2438	3.6709	-1.8750
10	H	0.4816	4.0277	2.0235
11	H	-0.5332	1.7873	1.7757
12	C	-1.5134	-0.1831	0.6327
13	C	-1.5261	-1.1712	1.6201
14	C	-2.6318	-1.3297	2.4597
15	C	-3.7583	-0.5106	2.3445
16	C	-3.7391	0.4886	1.3595
17	C	-2.6379	0.6551	0.5266
18	H	-2.6552	1.4471	-0.2156
19	H	-4.5962	1.1480	1.2467
20	H	-2.6129	-2.1118	3.2145
21	H	-0.6787	-1.8349	1.7420
22	C	-0.9333	-0.1139	-1.8349
23	H	-1.2588	0.8860	-2.1306
24	C	0.0342	-0.5551	-2.8424
25	N	0.7889	-0.8897	-3.6582
26	C	-2.1834	-1.0162	-1.9301
27	O	-2.2087	-2.2081	-1.7174
28	O	-3.2391	-0.2841	-2.2912
29	C	-4.5059	-0.9837	-2.3200
30	H	-4.4541	-1.8315	-3.0061
31	H	-5.2319	-0.2481	-2.6624
32	H	-4.7500	-1.3329	-1.3137
33	C	0.7898	-1.0161	-0.0784
34	C	1.9222	-0.6631	0.6721
35	C	2.9189	-1.5920	0.9574
36	C	2.7779	-2.8934	0.4846
37	C	1.6622	-3.2835	-0.2531
38	C	0.6739	-2.3441	-0.5248
39	H	-0.2049	-2.6574	-1.0743
40	H	1.5723	-4.3043	-0.6024
41	H	3.7920	-1.3150	1.5345
42	H	2.0422	0.3460	1.0448

43	C	-4.9467	-0.6689	3.2617
44	H	-4.9511	-1.6495	3.7476
45	H	-4.9355	0.0926	4.0522
46	H	-5.8895	-0.5527	2.7157
47	N	2.0009	5.3028	0.2320
48	O	2.6800	5.7396	-0.6988
49	O	1.7928	5.9156	1.2808
50	N	3.8251	-3.8791	0.7773
51	O	3.6749	-5.0267	0.3535
52	O	4.8012	-3.5100	1.4337

obn_an_MeProp
Energy (POTENTIAL) = -385.561434511 Eh

	Atom	X	Y	Z
1	O	-1.0858	-3.1833	0.2227
2	C	0.0839	-2.5746	0.3642
3	H	0.6195	-2.7351	1.3645
4	H	0.9097	-2.8894	-0.3655
5	C	0.0732	-1.0270	0.2246
6	C	1.2252	-0.2408	0.3718
7	C	-1.1261	-0.3753	-0.0622
8	C	1.1769	1.1463	0.2390
9	H	2.1765	-0.7284	0.5923
10	C	-1.1811	1.0162	-0.1975
11	H	-2.0029	-1.0087	-0.1746
12	C	-0.0331	1.8030	-0.0469
13	H	2.0866	1.7354	0.3563
14	H	-2.1304	1.5021	-0.4242
15	C	-0.0845	3.3102	-0.1666
16	H	-1.0283	3.6449	-0.6118
17	H	0.0055	3.7994	0.8134
18	H	0.7339	3.6947	-0.7885

SP_ar3no2cnoac
Energy (POTENTIAL) = -1541.885216 Eh

	Atom	X	Y	Z
1	C	-0.3126	0.0178	-0.3384
2	C	0.2964	1.4368	-0.2126
3	C	0.1361	2.1850	0.9637
4	C	0.7240	3.4385	1.1076
5	C	1.4838	3.9479	0.0578
6	C	1.6787	3.2275	-1.1190
7	C	1.0893	1.9742	-1.2409
8	H	1.2717	1.4144	-2.1508
9	H	2.2840	3.6397	-1.9162
10	H	0.5973	4.0130	2.0163
11	H	-0.4502	1.7883	1.7828
12	C	-1.4991	-0.1473	0.6381
13	C	-1.5332	-1.1239	1.6361
14	C	-2.6431	-1.2496	2.4764
15	C	-3.7518	-0.4081	2.3508
16	C	-3.7099	0.5808	1.3555
17	C	-2.6047	0.7149	0.5225
18	H	-2.6037	1.5003	-0.2269
19	H	-4.5521	1.2578	1.2351
20	H	-2.6409	-2.0229	3.2404
21	H	-0.6996	-1.8029	1.7684
22	C	-0.9142	-0.1173	-1.8306
23	H	-1.2127	0.8857	-2.1434
24	C	0.0458	-0.6003	-2.8257
25	N	0.7964	-0.9706	-3.6298
26	C	-2.1868	-0.9890	-1.9163
27	O	-2.2396	-2.1789	-1.6926
28	O	-3.2236	-0.2355	-2.2816
29	C	-4.5103	-0.8998	-2.2991
30	H	-4.4833	-1.7578	-2.9737
31	H	-5.2155	-0.1488	-2.6509
32	H	-4.7627	-1.2262	-1.2873
33	C	0.7892	-1.0308	-0.0633
34	C	1.9418	-0.6879	0.6603
35	C	2.9204	-1.6343	0.9517
36	C	2.7406	-2.9425	0.5120
37	C	1.6042	-3.3225	-0.1998
38	C	0.6352	-2.3656	-0.4779
39	H	-0.2588	-2.6686	-1.0087
40	H	1.4831	-4.3484	-0.5238
41	H	3.8083	-1.3640	1.5089
42	H	2.0924	0.3258	1.0082
43	C	-4.9475	-0.5331	3.2636
44	H	-4.9277	-1.4721	3.8250
45	H	-4.9734	0.2895	3.9899
46	H	-5.8849	-0.4913	2.6977
47	N	2.1015	5.2706	0.1976
48	O	2.7715	5.7008	-0.7438
49	O	1.9209	5.8875	1.2497
50	N	3.7672	-3.9456	0.8111

51	O	3.5867	-5.0985	0.4124
52	O	4.7614	-3.5886	1.4475

ar3no2cnoac_MeButa
Energy (POTENTIAL) = -1541.46012924 Eh

	Atom	X	Y	Z
1	C	-0.3314	0.0144	-0.3432
2	C	0.2540	1.4426	-0.2051
3	C	0.0625	2.1905	0.9666
4	C	0.6312	3.4523	1.1187
5	C	1.4028	3.9704	0.0825
6	C	1.6287	3.2509	-1.0893
7	C	1.0585	1.9895	-1.2197
8	H	1.2663	1.4284	-2.1233
9	H	2.2438	3.6709	-1.8750
10	H	0.4816	4.0277	2.0235
11	H	-0.5332	1.7873	1.7757
12	C	-1.5134	-0.1831	0.6327
13	C	-1.5261	-1.1712	1.6201
14	C	-2.6318	-1.3297	2.4597
15	C	-3.7583	-0.5106	2.3445
16	C	-3.7391	0.4886	1.3595
17	C	-2.6379	0.6551	0.5266
18	H	-2.6552	1.4471	-0.2156
19	H	-4.5962	1.1480	1.2467
20	H	-2.6129	-2.1118	3.2145
21	H	-0.6787	-1.8349	1.7420
22	C	-0.9333	-0.1139	-1.8349
23	H	-1.2588	0.8860	-2.1306
24	C	0.0342	-0.5551	-2.8424
25	N	0.7889	-0.8897	-3.6582
26	C	-2.1834	-1.0162	-1.9301
27	O	-2.2087	-2.2081	-1.7174
28	O	-3.2391	-0.2841	-2.2912
29	C	-4.5059	-0.9837	-2.3200
30	H	-4.4541	-1.8315	-3.0061
31	H	-5.2319	-0.2481	-2.6624
32	H	-4.7500	-1.3329	-1.3137
33	C	0.7898	-1.0161	-0.0784
34	C	1.9222	-0.6631	0.6721
35	C	2.9189	-1.5920	0.9574
36	C	2.7779	-2.8934	0.4846
37	C	1.6622	-3.2835	-0.2531
38	C	0.6739	-2.3441	-0.5248
39	H	-0.2049	-2.6574	-1.0743
40	H	1.5723	-4.3043	-0.6024
41	H	3.7920	-1.3150	1.5345
42	H	2.0422	0.3460	1.0448
43	C	-4.9467	-0.6689	3.2617
44	H	-4.9511	-1.6495	3.7476
45	H	-4.9355	0.0926	4.0522
46	H	-5.8895	-0.5527	2.7157
47	N	2.0009	5.3028	0.2320
48	O	2.6800	5.7396	-0.6988
49	O	1.7928	5.9156	1.2808
50	N	3.8251	-3.8791	0.7773
51	O	3.6749	-5.0267	0.3535
52	O	4.8011	-3.5100	1.4337

SP_cnoac_an_MeButa
Energy (POTENTIAL) = -360.2616277 Eh

	Atom	X	Y	Z
1	C	-0.8576	0.7620	-0.0073
2	H	-0.7549	1.8410	0.0155
3	C	-2.1365	0.2079	-0.0645
4	N	-3.2254	-0.2387	-0.1130
5	C	0.2937	-0.0542	0.0237
6	O	0.3816	-1.2908	0.0050
7	O	1.4589	0.7241	0.0841
8	C	2.6705	-0.0189	0.1083
9	H	2.7165	-0.6963	0.9694
10	H	3.4758	0.7181	0.1761
11	H	2.7986	-0.6208	-0.7997

ar3m2_cat_MeButa
Energy (POTENTIAL) = -1345.48558493 Eh

	Atom	X	Y	Z
1	C	-0.1700	0.0031	1.1709
2	C	-0.1906	-1.2388	0.4564
3	C	0.3530	-1.3709	-0.8542
4	C	-0.7510	-2.4179	1.0252
5	C	0.3367	-2.5630	-1.5368
6	H	0.8580	-0.5223	-1.3033
7	C	-0.7942	-3.6104	0.3431
8	H	-1.2193	-2.3612	2.0017
9	C	-0.2441	-3.7377	-0.9685

10	H	0.8285	-2.6066	-2.4999
11	H	-1.3021	-4.4458	0.8068
12	C	-0.1226	-0.0150	2.6374
13	C	-0.8855	0.8960	3.4000
14	C	0.6824	-0.9469	3.3272
15	C	-0.8500	0.8640	4.7880
16	H	-1.5351	1.6018	2.8922
17	C	0.7280	-0.9556	4.7157
18	H	1.2951	-1.6420	2.7623
19	C	-0.0379	-0.0547	5.4725
20	H	-1.4659	1.5577	5.3542
21	H	1.3696	-1.6700	5.2246
22	C	-0.1971	1.2626	0.4869
23	C	0.3973	2.4303	1.0465
24	C	-0.8356	1.4263	-0.7748
25	C	0.3877	3.6399	0.3953
26	H	0.9257	2.3527	1.9904
27	C	-0.8728	2.6374	-1.4250
28	H	-1.3752	0.5893	-1.2051
29	C	-0.2446	3.7957	-0.8756
30	H	-1.4423	2.7081	-2.3423
31	C	-0.6895	-6.1867	-1.0207
32	C	-0.1455	-5.0364	-3.0983
33	C	0.1976	-7.3392	-1.5027
34	H	-1.7376	-6.3834	-1.2862
35	H	-0.6084	-6.1287	0.0642
36	C	0.7291	-6.2278	-3.4847
37	H	-1.1560	-5.1845	-3.5051
38	H	0.2603	-4.1254	-3.5327
39	H	-0.1991	-8.2868	-1.1289
40	H	1.2178	-7.2019	-1.1096
41	H	0.7214	-6.3579	-4.5701
42	H	1.7652	-6.0524	-3.1537
43	N	-0.2516	-4.9268	-1.6357
44	O	0.2242	-7.4307	-2.9196
45	N	-0.2402	4.9889	-1.5374
46	C	-0.7694	5.1415	-2.8990
47	C	-0.0664	6.2841	-0.8614
48	C	0.0717	6.1572	-3.6782
49	H	-1.8159	5.4748	-2.8554
50	H	-0.7261	4.1909	-3.4299
51	C	0.7534	7.2382	-1.7276
52	H	-1.0610	6.7188	-0.6874
53	H	0.4194	6.1603	0.1039
54	H	-0.3931	6.3477	-4.6491
55	H	1.0802	5.7447	-3.8426
56	O	0.1543	7.4050	-3.0057
57	H	0.7876	8.2257	-1.2601
58	H	1.7821	6.8574	-1.8315
59	H	0.9126	4.4687	0.8520
60	C	0.0289	-0.0537	6.9782
61	H	0.7831	0.6634	7.3286
62	H	-0.9284	0.2400	7.4206
63	H	0.3060	-1.0375	7.3693

obn_an_m1_PMMA
Energy (POTENTIAL) = -501.309731375 Eh

	Atom	X	Y	Z
1	O	0.9631	-2.3802	-1.4194
2	C	1.6339	-1.4293	-0.7294
3	H	2.0100	-1.7538	0.2844
4	H	2.5779	-1.1006	-1.2451
5	C	0.8654	-0.1221	-0.4449
6	C	1.5008	0.9937	0.1188
7	C	-0.4957	-0.0178	-0.7417
8	C	0.8015	2.1711	0.3801
9	H	2.5650	0.9382	0.3516
10	C	-1.2001	1.1604	-0.4743
11	H	-0.9901	-0.8893	-1.1591
12	C	-0.5683	2.2753	0.0890
13	H	1.3212	3.0256	0.8132
14	H	-2.2627	1.2162	-0.7093
15	C	-1.3334	3.5416	0.4036
16	H	-2.2672	3.5982	-0.1671
17	H	-1.5994	3.5999	1.4685
18	H	-0.7437	4.4374	0.1732
19	O	-1.0846	-3.2821	-0.2279
20	H	-0.2222	-2.9196	-0.7085
21	C	-0.9369	-2.9900	1.1327
22	H	-0.7794	-1.9134	1.3318
23	H	-1.8483	-3.2894	1.6753
24	H	-0.0919	-3.5273	1.6101

SP_ar3m2_rad_MeProp
Energy (POTENTIAL) = -1345.9700555 Eh

	Atom	X	Y	Z

1	C	-0.0911	-0.0165	1.2406
2	C	-0.0198	-1.2860	0.5256
3	C	0.7059	-1.4233	-0.6855
4	C	-0.6689	-2.4522	0.9923
5	C	0.7772	-2.6250	-1.3687
6	H	1.2519	-0.5685	-1.0723
7	C	-0.6134	-3.6588	0.3040
8	H	-1.2601	-2.3988	1.9013
9	C	0.1133	-3.7803	-0.8976
10	H	1.3887	-2.6837	-2.2638
11	H	-1.1690	-4.5025	0.6973
12	C	-0.1943	0.0008	2.7002
13	C	-0.9347	0.9979	3.3798
14	C	0.4355	-0.9791	3.5049
15	C	-1.0375	1.0089	4.7664
16	H	-1.4522	1.7567	2.8011
17	C	0.3298	-0.9589	4.8911
18	H	1.0264	-1.7543	3.0267
19	C	-0.4066	0.0346	5.5548
20	H	-1.6297	1.7835	5.2493
21	H	0.8360	-1.7258	5.4739
22	C	-0.0654	1.2422	0.4989
23	C	0.5417	2.4075	1.0189
24	C	-0.6488	1.3673	-0.7871
25	C	0.5645	3.6083	0.3181
26	H	1.0120	2.3659	1.9965
27	C	-0.6252	2.5581	-1.4932
28	H	-1.1368	0.5050	-1.2307
29	C	-0.0176	3.7173	-0.9609
30	H	-1.0705	2.5893	-2.4827
31	C	-0.3209	-6.2031	-0.9862
32	C	-0.0303	-4.9674	-3.0464
33	C	0.2127	-7.4383	-1.7111
34	H	-1.4249	-6.2088	-1.0197
35	H	-0.0110	-6.2524	0.0620
36	C	0.4962	-6.2419	-3.6980
37	H	-1.1143	-4.8829	-3.2378
38	H	0.4578	-4.1041	-3.5019
39	H	-0.2605	-8.3411	-1.3142
40	H	1.3015	-7.5109	-1.5556
41	H	0.2273	-6.2648	-4.7581
42	H	1.5944	-6.2762	-3.6084
43	N	0.2299	-4.9950	-1.5987
44	O	-0.0803	-7.4005	-3.1035
45	N	0.0247	4.9027	-1.7197
46	C	-1.2389	5.3721	-2.3093
47	C	0.8174	6.0213	-1.2108
48	C	-0.9584	6.4208	-3.3805
49	H	-1.8833	5.8131	-1.5288
50	H	-1.7805	4.5378	-2.7582
51	C	1.0253	7.0524	-2.3194
52	H	0.3287	6.5068	-0.3473
53	H	1.7963	5.6550	-0.8870
54	H	-1.8967	6.8355	-3.7603
55	H	-0.4098	5.9566	-4.2165
56	O	-0.2126	7.5122	-2.8508
57	H	1.5441	7.9305	-1.9241
58	H	1.6390	6.6086	-3.1204
59	H	1.0366	4.4670	0.7817
60	C	-0.4919	0.0700	7.0615
61	H	0.3452	0.6358	7.4933
62	H	-1.4161	0.5510	7.3998
63	H	-0.4548	-0.9377	7.4897

cnoac_an_PMMA
Energy (POTENTIAL) = -360.125814287 Eh

	Atom	X	Y	Z
1	C	-0.8576	0.7583	-0.0073
2	H	-0.7534	1.8373	0.0153
3	C	-2.1385	0.2075	-0.0639
4	N	-3.2302	-0.2319	-0.1118
5	C	0.2938	-0.0569	0.0236
6	O	0.3873	-1.2925	0.0057
7	O	1.4597	0.7257	0.0832
8	C	2.6697	-0.0172	0.1079
9	H	2.7141	-0.6981	0.9666
10	H	3.4761	0.7189	0.1795
11	H	2.8003	-0.6176	-0.8012

uvvisfrag1_catb3lyp
Energy (POTENTIAL) = -1345.7674817 Eh

	Atom	X	Y	Z
1	C	-0.1734	0.0030	1.1688
2	C	-0.1948	-1.2388	0.4546
3	C	0.3467	-1.3705	-0.8570
4	C	-0.7537	-2.4182	1.0246

5	C	0.3302	-2.5627	-1.5394
6	H	0.8501	-0.5219	-1.3074
7	C	-0.7973	-3.6109	0.3428
8	H	-1.2207	-2.3619	2.0017
9	C	-0.2489	-3.7374	-0.9696
10	H	0.8200	-2.6059	-2.5035
11	H	-1.3038	-4.4467	0.8071
12	C	-0.1241	-0.0150	2.6358
13	C	-0.8860	0.8961	3.3990
14	C	0.6818	-0.9465	3.3247
15	C	-0.8479	0.8654	4.7872
16	H	-1.5359	1.6021	2.8921
17	C	0.7294	-0.9547	4.7134
18	H	1.2929	-1.6426	2.7594
19	C	-0.0347	-0.0530	5.4708
20	H	-1.4622	1.5602	5.3538
21	H	1.3712	-1.6697	5.2214
22	C	-0.2015	1.2624	0.4853
23	C	0.3916	2.4308	1.0452
24	C	-0.8402	1.4256	-0.7766
25	C	0.3798	3.6407	0.3949
26	H	0.9211	2.3536	1.9885
27	C	-0.8797	2.6368	-1.4262
28	H	-1.3782	0.5882	-1.2079
29	C	-0.2544	3.7962	-0.8753
30	H	-1.4485	2.7064	-2.3440
31	C	-0.6849	-6.1878	-1.0190
32	C	-0.1535	-5.0368	-3.0988
33	C	0.2108	-7.3339	-1.4995
34	H	-1.7317	-6.3927	-1.2832
35	H	-0.6034	-6.1281	0.0656
36	C	0.7304	-6.2206	-3.4858
37	H	-1.1639	-5.1943	-3.5022
38	H	0.2428	-4.1232	-3.5361
39	H	-0.1781	-8.2839	-1.1233
40	H	1.2302	-7.1876	-1.1082
41	H	0.7211	-6.3519	-4.5711
42	H	1.7655	-6.0361	-3.1574
43	N	-0.2563	-4.9265	-1.6364
44	O	0.2376	-7.4287	-2.9176
45	N	-0.2551	4.9905	-1.5342
46	C	-0.7746	5.1461	-2.8988
47	C	-0.0761	6.2852	-0.8601
48	C	0.0867	6.1470	-3.6748
49	H	-1.8160	5.4952	-2.8617
50	H	-0.7431	4.1943	-3.4278
51	C	0.7656	7.2261	-1.7193
52	H	-1.0683	6.7313	-0.7017
53	H	0.3942	6.1606	0.1125
54	H	-0.3679	6.3406	-4.6501
55	H	1.0904	5.7201	-3.8296
56	O	0.1825	7.3974	-3.0058
57	H	0.8077	8.2145	-1.2541
58	H	1.7899	6.8313	-1.8114
59	H	0.9045	4.4698	0.8512
60	C	0.0351	-0.0507	6.9766
61	H	0.7930	0.6635	7.3248
62	H	-0.9201	0.2474	7.4205
63	H	0.3091	-1.0354	7.3677

SP_ar3no2_cat_MeButa
Energy (POTENTIAL) = -1181.5286902 Eh

	Atom	X	Y	Z
1	C	-0.0054	-0.0026	0.5226
2	C	0.0348	-1.2523	-0.2303
3	C	0.7801	-1.3371	-1.4299
4	C	-0.6843	-2.3849	0.2191
5	C	0.8293	-2.5251	-2.1449
6	H	1.3529	-0.4833	-1.7730
7	C	-0.6615	-3.5673	-0.5078
8	H	-1.2964	-2.3166	1.1110
9	C	0.1020	-3.6170	-1.6725
10	H	1.4148	-2.6140	-3.0509
11	H	-1.2251	-4.4348	-0.1896
12	C	-0.0060	-0.0071	1.9473
13	C	-0.5923	1.0654	2.6869
14	C	0.5782	-1.0851	2.6802
15	C	-0.6027	1.0438	4.0658
16	H	-1.0928	1.8689	2.1590
17	C	0.5839	-1.0750	4.0596
18	H	1.0762	-1.8869	2.1476
19	C	-0.0086	-0.0175	4.7817
20	H	-1.0856	1.8485	4.6114
21	H	1.0596	-1.8873	4.6000
22	C	-0.0443	1.2521	-0.2221
23	C	0.6734	2.3818	0.2364

24	C	-0.7870	1.3447	-1.4227
25	C	0.6514	3.5693	-0.4823
26	H	1.2836	2.3077	1.1291
27	C	-0.8352	2.5376	-2.1298
28	H	-1.3586	0.4930	-1.7731
29	C	-0.1097	3.6266	-1.6482
30	H	-1.4188	2.6324	-3.0363
31	H	1.2138	4.4349	-0.1569
32	C	0.0163	-0.0044	6.2804
33	H	0.8936	0.5595	6.6272
34	H	-0.8689	0.4898	6.6914
35	H	0.0908	-1.0140	6.6935
36	N	-0.1450	4.8945	-2.4105
37	O	-0.8273	4.9243	-3.4319
38	O	0.5094	5.8384	-1.9738
39	N	0.1381	-4.8796	-2.4436
40	O	0.8237	-4.9031	-3.4630
41	O	-0.5189	-5.8257	-2.0156

ar3no2obn_PMMA
Energy (POTENTIAL) = -1566.94007134 Eh

	Atom	X	Y	Z
1	C	-0.3824	-0.2306	-0.3930
2	C	-1.7416	0.1277	0.2324
3	C	-2.6782	0.8031	-0.5677
4	C	-2.0220	-0.0495	1.5932
5	C	-3.8780	1.2603	-0.0378
6	H	-2.4605	0.9704	-1.6167
7	C	-3.2209	0.3993	2.1434
8	H	-1.3029	-0.5368	2.2411
9	C	-4.1362	1.0441	1.3165
10	H	-4.6056	1.7763	-0.6514
11	H	-3.4439	0.2599	3.1936
12	C	0.4417	-1.2641	0.4027
13	C	1.8238	-1.0827	0.5510
14	C	-0.1413	-2.4451	0.8897
15	C	2.6059	-2.0370	1.1939
16	H	2.2893	-0.1890	0.1532
17	C	0.6245	-3.4104	1.5324
18	H	-1.2050	-2.6169	0.7674
19	C	1.9939	-3.1901	1.6816
20	H	3.6730	-1.8985	1.3156
21	H	0.1785	-4.3203	1.9136
22	C	-0.5335	-0.8143	-1.8094
23	C	0.3764	-0.4721	-2.8152
24	C	-1.5285	-1.7566	-2.0984
25	C	0.2823	-1.0534	-4.0797
26	H	1.1547	0.2528	-2.6077
27	C	-1.6146	-2.3347	-3.3633
28	H	-2.2513	-2.0379	-1.3381
29	C	-0.7127	-1.9924	-4.3795
30	H	0.9992	-0.7713	-4.8475
31	H	-2.3977	-3.0622	-3.5638
32	O	0.3616	0.9837	-0.5524
33	C	0.5025	1.8276	0.6072
34	H	-0.4173	2.4113	0.7397
35	H	0.6506	1.2254	1.5116
36	C	1.6896	2.7186	0.3628
37	C	2.8869	2.5365	1.0612
38	C	1.6266	3.7235	-0.6114
39	C	3.9946	3.3450	0.7980
40	H	2.9562	1.7558	1.8154
41	C	2.7343	4.5248	-0.8757
42	H	0.7047	3.8691	-1.1691
43	C	3.9369	4.3523	-0.1725
44	H	4.9166	3.1886	1.3531
45	H	2.6686	5.2984	-1.6375
46	C	5.1229	5.2489	-0.4399
47	H	5.2023	5.5008	-1.5031
48	H	5.0327	6.1946	0.1108
49	H	6.0612	4.7778	-0.1296
50	C	-0.8288	-2.5940	-5.7598
51	H	-1.4826	-1.9866	-6.3997
52	H	0.1465	-2.6529	-6.2544
53	H	-1.2553	-3.6021	-5.7231
54	N	-5.4041	1.5175	1.8868
55	O	-6.1973	2.0892	1.1367
56	O	-5.6093	1.3188	3.0857
57	N	2.8092	-4.1993	2.3643
58	O	2.2461	-5.2137	2.7822
59	O	4.0167	-3.9826	2.4874

SP_cnoac_an_PMMA
Energy (POTENTIAL) = -360.2543252 Eh

	Atom	X	Y	Z
1	C	-0.8576	0.7583	-0.0073

2	H	-0.7534	1.8373	0.0153
3	C	-2.1385	0.2075	-0.0639
4	N	-3.2302	-0.2319	-0.1118
5	C	0.2938	-0.0569	0.0236
6	O	0.3873	-1.2925	0.0057
7	O	1.4597	0.7257	0.0832
8	C	2.6697	-0.0172	0.1079
9	H	2.7141	-0.6981	0.9666
10	H	3.4761	0.7189	0.1795
11	H	2.8003	-0.6176	-0.8012

SP_obn_rad
Energy (POTENTIAL) = -385.5481546 Eh

	Atom	X	Y	Z
1	O	0.0692	-2.9195	-0.8996
2	C	0.1192	-2.5522	0.4314
3	H	-0.7462	-2.9131	1.0097
4	H	1.0527	-2.8617	0.9278
5	C	0.0696	-1.0301	0.2993
6	C	1.2515	-0.2976	0.1265
7	C	-1.1628	-0.3658	0.2421
8	C	1.2011	1.0849	-0.0346
9	H	2.2091	-0.8114	0.1331
10	C	-1.2058	1.0168	0.0808
11	H	-2.0849	-0.9327	0.3388
12	C	-0.0263	1.7658	-0.0564
13	H	2.1255	1.6456	-0.1492
14	H	-2.1670	1.5244	0.0565
15	C	-0.0759	3.2672	-0.2000
16	H	-0.9947	3.5920	-0.6992
17	H	-0.0509	3.7529	0.7846
18	H	0.7793	3.6440	-0.7703

obn_an_m2
Energy (POTENTIAL) = -617.083567298 Eh

	Atom	X	Y	Z
1	O	0.4556	-1.6727	-1.2485
2	C	1.1971	-0.9304	-0.3554
3	H	1.1822	-1.3442	0.6826
4	H	2.2813	-0.9189	-0.6177
5	C	0.7680	0.5336	-0.2316
6	C	1.5726	1.4697	0.4349
7	C	-0.4438	0.9773	-0.7674
8	C	1.1777	2.8004	0.5636
9	H	2.5269	1.1510	0.8534
10	C	-0.8422	2.3118	-0.6359
11	H	-1.0761	0.2541	-1.2721
12	C	-0.0421	3.2474	0.0299
13	H	1.8249	3.5075	1.0801
14	H	-1.7922	2.6319	-1.0612
15	C	-0.4765	4.6867	0.1916
16	H	-1.2961	4.9375	-0.4902
17	H	-0.8265	4.8872	1.2134
18	H	0.3501	5.3804	-0.0035
19	O	-1.9840	-2.0713	-0.4411
20	H	-1.0239	-1.9146	-0.7589
21	C	-2.0238	-1.7287	0.9259
22	H	-1.6801	-0.6978	1.1145
23	H	-3.0614	-1.8031	1.2800
24	H	-1.4126	-2.4012	1.5530
25	O	1.2273	-4.0985	-0.7917
26	H	0.9537	-3.1445	-1.0352
27	C	0.3787	-4.4700	0.2714
28	H	-0.6765	-4.2266	0.0718
29	H	0.4565	-5.5547	0.4335
30	H	0.6447	-3.9784	1.2271

ar3m2obn_MeButa
Energy (POTENTIAL) = -1731.15558334 Eh

	Atom	X	Y	Z
1	C	-0.1385	0.2977	-0.8996
2	C	-1.4380	-0.3578	-0.4145
3	C	-2.6220	-0.1377	-1.1390
4	C	-1.5530	-1.0276	0.8053
5	C	-3.8503	-0.5927	-0.6838
6	H	-2.5782	0.4165	-2.0716
7	C	-2.7814	-1.5056	1.2684
8	H	-0.6716	-1.1958	1.4150
9	C	-3.9610	-1.3014	0.5324
10	H	-4.7419	-0.3727	-1.2626
11	H	-2.8023	-2.0478	2.2066
12	C	1.1483	-0.2834	-0.2798
13	C	2.2100	0.5651	0.0645
14	C	1.3613	-1.6600	-0.1569
15	C	3.4171	0.0628	0.5355
16	H	2.0912	1.6363	-0.0557

17	C	2.5651	-2.1755	0.3250
18	H	0.5718	-2.3548	-0.4269
19	C	3.6238	-1.3227	0.6854
20	H	4.2226	0.7548	0.7618
21	H	2.6625	-3.2501	0.4275
22	C	0.0227	0.1883	-2.4269
23	C	0.4960	1.2658	-3.1818
24	C	-0.2287	-1.0229	-3.0857
25	C	0.7035	1.1346	-4.5567
26	H	0.7015	2.2098	-2.6913
27	C	-0.0181	-1.1489	-4.4568
28	H	-0.6008	-1.8739	-2.5230
29	C	0.4502	-0.0706	-5.2211
30	H	1.0712	1.9889	-5.1215
31	H	-0.2230	-2.1007	-4.9425
32	N	4.8727	-1.8011	1.1406
33	C	5.3790	-1.2311	2.3987
34	C	5.1117	-3.2426	1.0865
35	C	6.8541	-1.5774	2.5711
36	H	4.8101	-1.6283	3.2580
37	H	5.2638	-0.1455	2.3940
38	C	6.5974	-3.5255	1.3046
39	H	4.5232	-3.7840	1.8491
40	H	4.8231	-3.6216	0.1011
41	H	7.2160	-1.2293	3.5431
42	H	7.4423	-1.0896	1.7764
43	O	7.0600	-2.9865	2.5384
44	H	6.7742	-4.6042	1.3471
45	H	7.1762	-3.1036	0.4668
46	N	-5.2269	-1.7294	0.9819
47	C	-6.0535	-2.4845	0.0271
48	C	-5.3182	-2.3031	2.3240
49	C	-7.4916	-2.5631	0.5288
50	H	-5.6568	-3.5072	-0.0983
51	H	-6.0417	-1.9985	-0.9500
52	C	-6.7833	-2.3948	2.7486
53	H	-4.8607	-3.3077	2.3690
54	H	-4.7902	-1.6564	3.0315
55	H	-8.0908	-3.1940	-0.1342
56	H	-7.9304	-1.5520	0.5509
57	O	-7.5545	-3.1531	1.8234
58	H	-6.8642	-2.9007	3.7150
59	H	-7.1998	-1.3787	2.8443
60	O	-0.2136	1.7143	-0.6316
61	C	-0.5327	2.0929	0.7141
62	H	-1.6123	1.9796	0.8794
63	H	-0.0166	1.4456	1.4337
64	C	-0.1013	3.5247	0.8891
65	C	1.0299	3.8536	1.6420
66	C	-0.8072	4.5571	0.2574
67	C	1.4413	5.1823	1.7689
68	H	1.5972	3.0638	2.1291
69	C	-0.3927	5.8812	0.3807
70	H	-1.6831	4.3153	-0.3398
71	C	0.7372	6.2177	1.1426
72	H	2.3232	5.4168	2.3609
73	H	-0.9527	6.6688	-0.1189
74	C	1.1610	7.6595	1.2995
75	H	1.0305	8.2186	0.3663
76	H	0.5599	8.1664	2.0661
77	H	2.2107	7.7391	1.6007
78	C	0.6464	-0.2031	-6.7133
79	H	-0.3021	-0.0727	-7.2514
80	H	1.3442	0.5498	-7.0945
81	H	1.0341	-1.1928	-6.9800

ar3m2obn_C
Energy (POTENTIAL) = -1731.16245873 Eh

	Atom	X	Y	Z
1	C	-0.5611	-0.7302	-0.3389
2	C	-1.8044	0.1588	-0.1467
3	C	-2.2218	0.6016	1.1177
4	C	-3.4003	1.3174	1.2885
5	C	-4.2432	1.6205	0.1960
6	C	-3.8282	1.1631	-1.0708
7	C	-2.6388	0.4522	-1.2283
8	H	-2.3647	0.1008	-2.2175
9	H	-4.4382	1.3372	-1.9493
10	N	-5.3982	2.3996	0.3906
11	C	-6.2085	2.8065	-0.7613
12	C	-7.2589	1.7484	-1.1112
13	H	-7.9198	2.0983	-1.9102
14	H	-6.7688	0.8183	-1.4405
15	O	-8.0925	1.4867	0.0199
16	C	-7.3266	1.0564	1.1456
17	C	-6.2776	2.1036	1.5333

18	H	-5.7008	1.7555	2.3908
19	H	-6.7840	3.0320	1.8250
20	H	-8.0380	0.8949	1.9616
21	H	-6.8240	0.1009	0.9202
22	H	-5.5691	3.0394	-1.6157
23	H	-6.7304	3.7309	-0.4827
24	H	-3.6520	1.6720	2.2825
25	H	-1.6063	0.4038	1.9897
26	C	0.6313	-0.3413	0.5408
27	C	1.6308	-1.2807	0.8190
28	C	2.8196	-0.9322	1.4470
29	C	3.0825	0.4004	1.8373
30	C	2.0654	1.3437	1.5731
31	C	0.8821	0.9770	0.9396
32	H	0.1460	1.7505	0.7458
33	H	2.1868	2.3818	1.8578
34	N	4.2676	0.7643	2.5012
35	C	5.3987	-0.1759	2.5026
36	C	6.1639	-0.0915	1.1793
37	H	7.0473	-0.7370	1.1882
38	H	5.5108	-0.3945	0.3466
39	O	6.6423	1.2417	0.9670
40	C	5.5806	2.2031	0.9931
41	C	4.8024	2.1240	2.3089
42	H	4.0108	2.8727	2.3355
43	H	5.4763	2.3360	3.1466
44	H	6.0522	3.1830	0.8714
45	H	4.8958	2.0314	0.1501
46	H	5.0668	-1.1932	2.7140
47	H	6.0669	0.1188	3.3197
48	H	3.5556	-1.7111	1.5988
49	H	1.4948	-2.3124	0.5109
50	C	-1.0111	-2.1813	-0.0849
51	C	-1.2607	-2.6211	1.2238
52	C	-1.7179	-3.9121	1.4721
53	C	-1.9473	-4.8156	0.4229
54	C	-1.6943	-4.3734	-0.8794
55	C	-1.2358	-3.0771	-1.1337
56	H	-1.0462	-2.7632	-2.1528
57	H	-1.8549	-5.0519	-1.7147
58	C	-2.4672	-6.2070	0.6984
59	H	-1.9665	-6.6573	1.5633
60	H	-3.5420	-6.1908	0.9228
61	H	-2.3209	-6.8680	-0.1620
62	H	-1.8956	-4.2270	2.4982
63	H	-1.0815	-1.9503	2.0587
64	O	-0.1549	-0.7054	-1.7305
65	C	0.4090	0.5035	-2.2333
66	H	0.0317	1.3755	-1.6864
67	H	0.0288	0.5910	-3.2597
68	C	1.9260	0.5127	-2.2574
69	C	2.6657	-0.6705	-2.3035
70	C	4.0601	-0.6373	-2.3797
71	C	4.7570	0.5768	-2.4211
72	C	4.0062	1.7618	-2.3857
73	C	2.6152	1.7309	-2.2981
74	H	2.0591	2.6655	-2.2570
75	H	4.5207	2.7201	-2.4146
76	C	6.2668	0.6229	-2.4676
77	H	6.6890	-0.3546	-2.7234
78	H	6.6207	1.3498	-3.2082
79	H	6.6791	0.9207	-1.4952
80	H	4.6164	-1.5721	-2.4048
81	H	2.1420	-1.6199	-2.2575

SP_obn_an_MeButa
Energy (POTENTIAL) = -385.6944688 Eh

	Atom	X	Y	Z
1	O	-1.0838	-3.1837	0.2102
2	C	0.0840	-2.5748	0.3629
3	H	0.6111	-2.7349	1.3681
4	H	0.9170	-2.8878	-0.3596
5	C	0.0732	-1.0270	0.2240
6	C	1.2236	-0.2401	0.3791
7	C	-1.1249	-0.3763	-0.0693
8	C	1.1748	1.1471	0.2478
9	H	2.1738	-0.7270	0.6057
10	C	-1.1804	1.0154	-0.2029
11	H	-2.0005	-1.0105	-0.1869
12	C	-0.0341	1.8030	-0.0448
13	H	2.0831	1.7370	0.3722
14	H	-2.1289	1.5009	-0.4336
15	C	-0.0841	3.3100	-0.1677
16	H	-1.0500	3.6481	-0.5594
17	H	0.0660	3.8024	0.8032
18	H	0.6988	3.6878	-0.8379

obn_an_m2c_PMMA
Energy (POTENTIAL) = -617.061608649 Eh

	Atom	X	Y	Z
1	O	0.8155	-1.3015	-1.2836
2	C	1.4998	-0.3596	-0.4976
3	H	1.7137	-0.7580	0.5129
4	H	2.4798	-0.1691	-0.9619
5	C	0.7745	0.9715	-0.3304
6	C	1.4765	2.1800	-0.2923
7	C	-0.6202	1.0068	-0.1770
8	C	0.8121	3.3947	-0.0990
9	H	2.5580	2.1774	-0.4227
10	C	-1.2781	2.2209	0.0138
11	H	-1.1731	0.0687	-0.2042
12	C	-0.5776	3.4362	0.0594
13	H	1.3811	4.3229	-0.0806
14	H	-2.3611	2.2288	0.1297
15	C	-1.3054	4.7412	0.2908
16	H	-2.2216	4.8019	-0.3088
17	H	-1.6041	4.8519	1.3423
18	H	-0.6783	5.6028	0.0362
19	O	-1.1605	-2.1353	0.1377
20	H	0.0204	-1.6549	-0.7236
21	C	-0.8102	-2.0961	1.4774
22	H	-0.5856	-1.0658	1.8329
23	H	-1.6160	-2.4736	2.1466
24	H	0.0901	-2.7018	1.7310
25	O	-0.8197	-4.6451	-0.4505
26	H	-1.0517	-3.6756	-0.2411
27	C	0.5874	-4.7031	-0.4347
28	H	0.9999	-4.8871	0.5780
29	H	0.9301	-5.5321	-1.0739
30	H	1.0464	-3.7744	-0.8051

SP_ar3no2_cat_PMMA
Energy (POTENTIAL) = -1181.5216719 Eh

	Atom	X	Y	Z
1	C	-0.0050	-0.0028	0.5220
2	C	0.0346	-1.2525	-0.2306
3	C	0.7786	-1.3376	-1.4313
4	C	-0.6835	-2.3856	0.2197
5	C	0.8273	-2.5258	-2.1462
6	H	1.3510	-0.4837	-1.7752
7	C	-0.6614	-3.5681	-0.5073
8	H	-1.2945	-2.3173	1.1124
9	C	0.1008	-3.6177	-1.6729
10	H	1.4117	-2.6158	-3.0528
11	H	-1.2244	-4.4361	-0.1894
12	C	-0.0058	-0.0073	1.9473
13	C	-0.5933	1.0644	2.6870
14	C	0.5794	-1.0844	2.6806
15	C	-0.6038	1.0430	4.0660
16	H	-1.0943	1.8675	2.1591
17	C	0.5852	-1.0742	4.0601
18	H	1.0781	-1.8859	2.1480
19	C	-0.0085	-0.0175	4.7824
20	H	-1.0877	1.8472	4.6114
21	H	1.0619	-1.8860	4.6006
22	C	-0.0437	1.2520	-0.2223
23	C	0.6727	2.3824	0.2372
24	C	-0.7852	1.3450	-1.4239
25	C	0.6512	3.5699	-0.4814
26	H	1.2820	2.3082	1.1307
27	C	-0.8333	2.5381	-2.1306
28	H	-1.3561	0.4931	-1.7751
29	C	-0.1087	3.6273	-1.6481
30	H	-1.4158	2.6340	-3.0378
31	H	1.2130	4.4361	-0.1563
32	C	0.0162	-0.0040	6.2811
33	H	0.8896	0.5658	6.6283
34	H	-0.8723	0.4846	6.6921
35	H	0.0969	-1.0128	6.6949
36	N	-0.1437	4.8962	-2.4108
37	O	-0.8257	4.9242	-3.4319
38	O	0.5106	5.8388	-1.9722
39	N	0.1364	-4.8812	-2.4445
40	O	0.8210	-4.9028	-3.4640
41	O	-0.5202	-5.8261	-2.0143

SP_ar3m2obn_meoh_MeButa
Energy (POTENTIAL) = -1847.3902267 Eh

	Atom	X	Y	Z
1	C	-0.0761	0.1737	-0.7615
2	C	-1.3082	-0.6171	-0.3109
3	C	-2.5527	-0.3341	-0.9033

4	C	-1.3010	-1.4991	0.7714
5	C	-3.7217	-0.9272	-0.4516
6	H	-2.6109	0.3922	-1.7075
7	C	-2.4696	-2.1163	1.2234
8	H	-0.3697	-1.7268	1.2785
9	C	-3.7102	-1.8466	0.6203
10	H	-4.6632	-0.6497	-0.9146
11	H	-2.3959	-2.8169	2.0468
12	C	1.2672	-0.3809	-0.2558
13	C	2.2578	0.4713	0.2500
14	C	1.5942	-1.7327	-0.4030
15	C	3.5045	-0.0134	0.6294
16	H	2.0584	1.5340	0.3273
17	C	2.8376	-2.2317	-0.0161
18	H	0.8629	-2.4201	-0.8181
19	C	3.8232	-1.3806	0.5172
20	H	4.2530	0.6855	0.9902
21	H	3.0245	-3.2938	-0.1244
22	C	0.0432	0.2723	-2.2917
23	C	0.6526	1.3890	-2.8787
24	C	-0.3501	-0.7809	-3.1242
25	C	0.8363	1.4570	-4.2586
26	H	0.9834	2.2083	-2.2503
27	C	-0.1577	-0.7104	-4.5045
28	H	-0.8178	-1.6620	-2.6962
29	C	0.4316	0.4112	-5.0996
30	H	1.3078	2.3379	-4.6894
31	H	-0.4766	-1.5422	-5.1286
32	N	5.1065	-1.8348	0.8935
33	C	5.5576	-1.4789	2.2482
34	C	5.4633	-3.2152	0.5689
35	C	7.0549	-1.7348	2.3833
36	H	5.0178	-2.0776	3.0030
37	H	5.3540	-0.4252	2.4483
38	C	6.9658	-3.4158	0.7612
39	H	4.9166	-3.9384	1.2008
40	H	5.2121	-3.4181	-0.4769
41	H	7.3805	-1.5535	3.4119
42	H	7.6070	-1.0581	1.7105
43	O	7.3744	-3.0904	2.0857
44	H	7.2308	-4.4648	0.5995
45	H	7.5131	-2.7950	0.0332
46	N	-4.9180	-2.4105	1.0753
47	C	-5.8007	-3.0127	0.0639
48	C	-4.8810	-3.2200	2.2927
49	C	-7.1883	-3.2465	0.6525
50	H	-5.3859	-3.9759	-0.2815
51	H	-5.8864	-2.3552	-0.8028
52	C	-6.3022	-3.4511	2.8038
53	H	-4.3924	-4.1951	2.1179
54	H	-4.3157	-2.6888	3.0645
55	H	-7.8217	-3.7729	-0.0675
56	H	-7.6542	-2.2773	0.8950
57	O	-7.1240	-4.0642	1.8168
58	H	-6.2875	-4.1258	3.6648
59	H	-6.7394	-2.4883	3.1153
60	O	-0.2304	1.5533	-0.3159
61	C	-0.5719	1.7508	1.0744
62	H	-1.6420	1.5552	1.2085
63	H	-0.0174	1.0426	1.6987
64	C	-0.2189	3.1705	1.4276
65	C	0.9285	3.4628	2.1720
66	C	-1.0195	4.2321	0.9832
67	C	1.2699	4.7842	2.4669
68	H	1.5617	2.6512	2.5234
69	C	-0.6723	5.5496	1.2752
70	H	-1.9045	4.0185	0.3897
71	C	0.4758	5.8494	2.0247
72	H	2.1663	4.9896	3.0477
73	H	-1.3029	6.3610	0.9179
74	C	0.8251	7.2791	2.3657
75	H	0.5757	7.9587	1.5434
76	H	0.2679	7.6210	3.2480
77	H	1.8913	7.3902	2.5891
78	C	0.6050	0.5061	-6.5970
79	H	-0.2163	1.0738	-7.0544
80	H	1.5370	1.0184	-6.8605
81	H	0.6169	-0.4845	-7.0636
82	C	-1.8128	3.8820	-2.5559
83	H	-2.6724	4.3971	-2.9967
84	H	-1.2192	4.6213	-1.9958
85	H	-1.1908	3.4828	-3.3698
86	O	-2.3162	2.8543	-1.7136
87	H	-1.5463	2.4065	-1.3053

meoh_MeProp

Energy (POTENTIAL) = -115.719395221 Eh

	Atom	X	Y	Z
1	C	0.0191	0.0016	-0.6648
2	H	-0.9918	0.0065	-1.0828
3	H	0.5486	0.8921	-1.0361
4	H	0.5404	-0.8933	-1.0372
5	O	-0.1209	0.0013	0.7525
6	H	0.7714	0.0003	1.1314

SP_ar3m2_rad_PMMA

Energy (POTENTIAL) = -1345.968185 Eh

	Atom	X	Y	Z
1	C	-0.0914	-0.0170	1.2400
2	C	-0.0190	-1.2865	0.5254
3	C	0.7079	-1.4236	-0.6849
4	C	-0.6680	-2.4526	0.9923
5	C	0.7801	-2.6255	-1.3675
6	H	1.2543	-0.5685	-1.0707
7	C	-0.6114	-3.6593	0.3042
8	H	-1.2598	-2.3986	1.9008
9	C	0.1158	-3.7803	-0.8966
10	H	1.3930	-2.6855	-2.2617
11	H	-1.1668	-4.5035	0.6969
12	C	-0.1963	0.0008	2.6994
13	C	-0.9385	0.9974	3.3774
14	C	0.4338	-0.9780	3.5050
15	C	-1.0430	1.0088	4.7637
16	H	-1.4562	1.7551	2.7973
17	C	0.3264	-0.9572	4.8910
18	H	1.0263	-1.7523	3.0276
19	C	-0.4120	0.0357	5.5532
20	H	-1.6368	1.7830	5.2456
21	H	0.8329	-1.7231	5.4749
22	C	-0.0650	1.2415	0.4982
23	C	0.5417	2.4065	1.0190
24	C	-0.6473	1.3665	-0.7881
25	C	0.5652	3.6073	0.3181
26	H	1.0111	2.3643	1.9970
27	C	-0.6226	2.5571	-1.4942
28	H	-1.1349	0.5040	-1.2317
29	C	-0.0155	3.7160	-0.9612
30	H	-1.0661	2.5889	-2.4846
31	C	-0.3073	-6.2052	-0.9823
32	C	-0.0430	-4.9673	-3.0437
33	C	0.2259	-7.4373	-1.7127
34	H	-1.4116	-6.2167	-1.0067
35	H	0.0116	-6.2523	0.0635
36	C	0.4834	-6.2388	-3.7016
37	H	-1.1295	-4.8885	-3.2239
38	H	0.4354	-4.1003	-3.5028
39	H	-0.2388	-8.3432	-1.3125
40	H	1.3166	-7.5041	-1.5673
41	H	0.2033	-6.2634	-4.7588
42	H	1.5827	-6.2668	-3.6236
43	N	0.2328	-4.9948	-1.5992
44	O	-0.0806	-7.4003	-3.1018
45	N	0.0267	4.9016	-1.7204
46	C	-1.2387	5.3748	-2.3024
47	C	0.8243	6.0179	-1.2146
48	C	-0.9609	6.4232	-3.3747
49	H	-1.8778	5.8175	-1.5184
50	H	-1.7849	4.5416	-2.7480
51	C	1.0295	7.0488	-2.3238
52	H	0.3407	6.5047	-0.3488
53	H	1.8034	5.6481	-0.8950
54	H	-1.8997	6.8413	-3.7496
55	H	-0.4181	5.9574	-4.2138
56	O	-0.2094	7.5119	-2.8487
57	H	1.5522	7.9259	-1.9311
58	H	1.6384	6.6036	-3.1277
59	H	1.0369	4.4661	0.7819
60	C	-0.4991	0.0718	7.0598
61	H	0.3367	0.6388	7.4925
62	H	-1.4244	0.5517	7.3968
63	H	-0.4617	-0.9357	7.4886

ar3m2_cat

Energy (POTENTIAL) = -1345.49407605 Eh

	Atom	X	Y	Z
1	C	-0.1734	0.0030	1.1688
2	C	-0.1948	-1.2388	0.4546
3	C	0.3467	-1.3705	-0.8570
4	C	-0.7537	-2.4182	1.0246
5	C	0.3302	-2.5627	-1.5394
6	H	0.8501	-0.5219	-1.3074
7	C	-0.7973	-3.6109	0.3428

8	H	-1.2207	-2.3619	2.0017
9	C	-0.2489	-3.7374	-0.9696
10	H	0.8200	-2.6059	-2.5035
11	H	-1.3038	-4.4467	0.8071
12	C	-0.1241	-0.0150	2.6358
13	C	-0.8860	0.8961	3.3990
14	C	0.6818	-0.9465	3.3247
15	C	-0.8479	0.8654	4.7872
16	H	-1.5359	1.6021	2.8921
17	C	0.7294	-0.9547	4.7134
18	H	1.2929	-1.6426	2.7594
19	C	-0.0347	-0.0530	5.4708
20	H	-1.4622	1.5602	5.3538
21	H	1.3712	-1.6697	5.2214
22	C	-0.2015	1.2624	0.4853
23	C	0.3916	2.4308	1.0452
24	C	-0.8402	1.4256	-0.7766
25	C	0.3798	3.6407	0.3949
26	H	0.9211	2.3536	1.9885
27	C	-0.8797	2.6368	-1.4262
28	H	-1.3782	0.5882	-1.2079
29	C	-0.2544	3.7962	-0.8753
30	H	-1.4485	2.7064	-2.3440
31	C	-0.6849	-6.1878	-1.0190
32	C	-0.1535	-5.0368	-3.0988
33	C	0.2108	-7.3339	-1.4995
34	H	-1.7317	-6.3927	-1.2832
35	H	-0.6034	-6.1281	0.0656
36	C	0.7304	-6.2206	-3.4858
37	H	-1.1639	-5.1943	-3.5022
38	H	0.2428	-4.1232	-3.5361
39	H	-0.1781	-8.2839	-1.1233
40	H	1.2302	-7.1876	-1.1082
41	H	0.7211	-6.3519	-4.5711
42	H	1.7655	-6.0361	-3.1574
43	N	-0.2563	-4.9265	-1.6364
44	O	0.2376	-7.4287	-2.9175
45	N	-0.2551	4.9905	-1.5342
46	C	-0.7746	5.1461	-2.8988
47	C	-0.0761	6.2852	-0.8601
48	C	0.0867	6.1470	-3.6748
49	H	-1.8160	5.4952	-2.8617
50	H	-0.7431	4.1943	-3.4278
51	C	0.7656	7.2261	-1.7193
52	H	-1.0683	6.7313	-0.7017
53	H	0.3942	6.1606	0.1125
54	H	-0.3679	6.3406	-4.6501
55	H	1.0904	5.7201	-3.8296
56	O	0.1825	7.3974	-3.0058
57	H	0.8077	8.2145	-1.2541
58	H	1.7899	6.8313	-1.8114
59	H	0.9045	4.4698	0.8512
60	C	0.0351	-0.0507	6.9766
61	H	0.7930	0.6635	7.3248
62	H	-0.9201	0.2474	7.4205
63	H	0.3091	-1.0354	7.3677

SP_ar3m2_rad
Energy (POTENTIAL) = -1345.9730832 Eh

	Atom	X	Y	Z
1	C	-0.0893	-0.0160	1.2397
2	C	-0.0201	-1.2856	0.5244
3	C	0.7034	-1.4243	-0.6879
4	C	-0.6695	-2.4517	0.9918
5	C	0.7729	-2.6261	-1.3714
6	H	1.2491	-0.5702	-1.0769
7	C	-0.6162	-3.6585	0.3037
8	H	-1.2596	-2.3986	1.9015
9	C	0.1091	-3.7817	-0.8992
10	H	1.3821	-2.6833	-2.2680
11	H	-1.1722	-4.5011	0.6987
12	C	-0.1902	0.0007	2.6998
13	C	-0.9283	0.9983	3.3815
14	C	0.4399	-0.9802	3.5032
15	C	-1.0287	1.0089	4.7684
16	H	-1.4461	1.7584	2.8046
17	C	0.3368	-0.9606	4.8898
18	H	1.0289	-1.7563	3.0242
19	C	-0.3973	0.0335	5.5555
20	H	-1.6191	1.7841	5.2526
21	H	0.8431	-1.7285	5.4712
22	C	-0.0642	1.2429	0.4984
23	C	0.5416	2.4091	1.0185
24	C	-0.6478	1.3687	-0.7877
25	C	0.5629	3.6105	0.3186
26	H	1.0122	2.3686	1.9961

27	C	-0.6264	2.5599	-1.4932
28	H	-1.1352	0.5066	-1.2324
29	C	-0.0198	3.7203	-0.9607
30	H	-1.0731	2.5900	-2.4820
31	C	-0.3426	-6.2012	-0.9911
32	C	-0.0123	-4.9702	-3.0497
33	C	0.1908	-7.4414	-1.7070
34	H	-1.4459	-6.1975	-1.0382
35	H	-0.0464	-6.2532	0.0607
36	C	0.5139	-6.2498	-3.6910
37	H	-1.0924	-4.8774	-3.2576
38	H	0.4901	-4.1128	-3.5003
39	H	-0.2955	-8.3392	-1.3148
40	H	1.2767	-7.5232	-1.5366
41	H	0.2618	-6.2711	-4.7552
42	H	1.6100	-6.2937	-3.5839
43	N	0.2246	-4.9969	-1.5976
44	O	-0.0821	-7.4033	-3.1041
45	N	0.0220	4.9057	-1.7176
46	C	-1.2378	5.3673	-2.3223
47	C	0.8034	6.0296	-1.2010
48	C	-0.9528	6.4185	-3.3895
49	H	-1.8938	5.8030	-1.5487
50	H	-1.7688	4.5306	-2.7790
51	C	1.0156	7.0626	-2.3065
52	H	0.3037	6.5103	-0.3414
53	H	1.7819	5.6703	-0.8689
54	H	-1.8900	6.8261	-3.7793
55	H	-0.3915	5.9595	-4.2197
56	O	-0.2203	7.5154	-2.8504
57	H	1.5252	7.9431	-1.9048
58	H	1.6397	6.6238	-3.1020
59	H	1.0343	4.4689	0.7832
60	C	-0.4800	0.0682	7.0623
61	H	0.3586	0.6329	7.4926
62	H	-1.4029	0.5505	7.4022
63	H	-0.4429	-0.9397	7.4898

obn_an_m2b_PMMA
Energy (POTENTIAL) = -617.063442582 Eh

	Atom	X	Y	Z
1	O	0.1057	-1.4151	-1.0190
2	C	1.1051	-0.6910	-0.4192
3	H	1.4069	-1.0864	0.5829
4	H	2.0557	-0.7117	-1.0063
5	C	0.7725	0.7888	-0.2016
6	C	1.7212	1.6797	0.3213
7	C	-0.4897	1.2916	-0.5231
8	C	1.4164	3.0253	0.5186
9	H	2.7157	1.3114	0.5737
10	C	-0.7975	2.6408	-0.3228
11	H	-1.2250	0.5989	-0.9194
12	C	0.1458	3.5318	0.2007
13	H	2.1727	3.6971	0.9230
14	H	-1.7903	3.0082	-0.5795
15	C	-0.1908	4.9862	0.4427
16	H	-1.0920	5.2854	-0.1037
17	H	-0.3735	5.1859	1.5077
18	H	0.6271	5.6472	0.1301
19	O	-2.0717	-1.7832	0.3514
20	H	-1.1966	-1.5637	-0.1251
21	C	-2.6558	-2.8119	-0.4174
22	H	-1.9257	-3.5898	-0.6882
23	H	-3.4605	-3.2842	0.1653
24	H	-3.1097	-2.4419	-1.3570
25	O	0.6090	-3.9361	-0.6261
26	H	0.4435	-2.9600	-0.8710
27	C	0.5790	-3.9700	0.7833
28	H	-0.2599	-3.3888	1.1944
29	H	0.4698	-5.0127	1.1164
30	H	1.5078	-3.5780	1.2420

SP_ar3no2obn_PMMA
Energy (POTENTIAL) = -1567.3564322 Eh

	Atom	X	Y	Z
1	C	-0.3824	-0.2306	-0.3930
2	C	-1.7416	0.1277	0.2324
3	C	-2.6782	0.8031	-0.5677
4	C	-2.0220	-0.0495	1.5932
5	C	-3.8780	1.2603	-0.0378
6	H	-2.4605	0.9704	-1.6167
7	C	-3.2209	0.3993	2.1434
8	H	-1.3029	-0.5368	2.2411
9	C	-4.1362	1.0441	1.3165
10	H	-4.6056	1.7763	-0.6514
11	H	-3.4439	0.2599	3.1936

12	C	0.4417	-1.2641	0.4027
13	C	1.8238	-1.0827	0.5510
14	C	-0.1413	-2.4451	0.8897
15	C	2.6059	-2.0370	1.1939
16	H	2.2893	-0.1890	0.1532
17	C	0.6245	-3.4104	1.5324
18	H	-1.2050	-2.6169	0.7674
19	C	1.9939	-3.1901	1.6816
20	H	3.6730	-1.8985	1.3156
21	H	0.1785	-4.3203	1.9136
22	C	-0.5335	-0.8143	-1.8094
23	C	0.3764	-0.4721	-2.8152
24	C	-1.5285	-1.7566	-2.0984
25	C	0.2823	-1.0534	-4.0797
26	H	1.1547	0.2528	-2.6077
27	C	-1.6146	-2.3347	-3.3633
28	H	-2.2513	-2.0379	-1.3381
29	C	-0.7126	-1.9924	-4.3795
30	H	0.9992	-0.7713	-4.8475
31	H	-2.3977	-3.0622	-3.5638
32	O	0.3616	0.9837	-0.5524
33	C	0.5025	1.8276	0.6072
34	H	-0.4173	2.4113	0.7397
35	H	0.6506	1.2254	1.5116
36	C	1.6896	2.7186	0.3628
37	C	2.8869	2.5365	1.0612
38	C	1.6266	3.7235	-0.6114
39	C	3.9946	3.3450	0.7980
40	H	2.9562	1.7558	1.8154
41	C	2.7343	4.5248	-0.8757
42	H	0.7047	3.8691	-1.1691
43	C	3.9369	4.3523	-0.1725
44	H	4.9166	3.1886	1.3531
45	H	2.6686	5.2984	-1.6375
46	C	5.1229	5.2489	-0.4399
47	H	5.2022	5.5008	-1.5031
48	H	5.0327	6.1946	0.1108
49	H	6.0612	4.7778	-0.1296
50	C	-0.8288	-2.5940	-5.7598
51	H	-1.4826	-1.9866	-6.3997
52	H	0.1465	-2.6529	-6.2544
53	H	-1.2553	-3.6021	-5.7232
54	N	-5.4041	1.5175	1.8868
55	O	-6.1973	2.0892	1.1367
56	O	-5.6093	1.3188	3.0857
57	N	2.8092	-4.1993	2.3643
58	O	2.2461	-5.2137	2.7822
59	O	4.0167	-3.9826	2.4874

obn_an_m1
Energy (POTENTIAL) = -501.331188661 Eh

	Atom	X	Y	Z
1	O	0.9590	-2.3642	-1.4368
2	C	1.6265	-1.4254	-0.7151
3	H	1.9715	-1.7739	0.2989
4	H	2.5851	-1.1056	-1.2041
5	C	0.8623	-0.1170	-0.4347
6	C	1.5026	1.0028	0.1163
7	C	-0.5008	-0.0138	-0.7223
8	C	0.8044	2.1824	0.3725
9	H	2.5681	0.9508	0.3419
10	C	-1.2040	1.1670	-0.4613
11	H	-0.9991	-0.8872	-1.1307
12	C	-0.5674	2.2862	0.0885
13	H	1.3277	3.0397	0.7940
14	H	-2.2674	1.2215	-0.6916
15	C	-1.3277	3.5578	0.3923
16	H	-2.2744	3.6002	-0.1571
17	H	-1.5670	3.6375	1.4617
18	H	-0.7441	4.4482	0.1295
19	O	-1.0676	-3.2993	-0.2418
20	H	-0.2118	-2.9122	-0.7175
21	C	-0.9350	-3.0199	1.1266
22	H	-0.8187	-1.9411	1.3380
23	H	-1.8355	-3.3606	1.6608
24	H	-0.0721	-3.5315	1.5973

SP_obn_rad_PMMA
Energy (POTENTIAL) = -385.5457777 Eh

	Atom	X	Y	Z
1	O	0.0682	-2.9286	-0.8982
2	C	0.1192	-2.5520	0.4292
3	H	-0.7454	-2.9113	1.0108
4	H	1.0530	-2.8600	0.9271
5	C	0.0696	-1.0300	0.2985
6	C	1.2508	-0.2972	0.1274

7	C	-1.1620	-0.3655	0.2424
8	C	1.2006	1.0853	-0.0334
9	H	2.2080	-0.8117	0.1317
10	C	-1.2051	1.0171	0.0814
11	H	-2.0840	-0.9330	0.3363
12	C	-0.0262	1.7661	-0.0552
13	H	2.1251	1.6459	-0.1486
14	H	-2.1665	1.5246	0.0561
15	C	-0.0759	3.2678	-0.1990
16	H	-0.9943	3.5925	-0.6990
17	H	-0.0506	3.7550	0.7849
18	H	0.7785	3.6446	-0.7707

ar3m2cnoac_PMMA
Energy (POTENTIAL) = -1705.67440492 Eh

	Atom	X	Y	Z
1	C	-1.0607	0.0298	-0.3977
2	C	-0.2468	1.3388	-0.2834
3	C	-0.1348	2.0307	0.9266
4	C	0.6497	3.1790	1.0512
5	C	1.3784	3.6812	-0.0401
6	C	1.2905	2.9683	-1.2532
7	C	0.5029	1.8307	-1.3656
8	H	0.5068	1.3058	-2.3154
9	H	1.8730	3.2890	-2.1110
10	H	0.6698	3.6853	2.0092
11	H	-0.6753	1.6809	1.7991
12	C	-2.1538	-0.0160	0.6954
13	C	-2.2619	-1.0614	1.6155
14	C	-3.2882	-1.0809	2.5641
15	C	-4.2408	-0.0607	2.6289
16	C	-4.1260	0.9954	1.7118
17	C	-3.1009	1.0212	0.7719
18	H	-3.0342	1.8601	0.0863
19	H	-4.8464	1.8100	1.7382
20	H	-3.3467	-1.9125	3.2625
21	H	-1.5488	-1.8766	1.5931
22	C	-1.8398	0.0723	-1.8155
23	H	-2.0147	1.1263	-2.0430
24	C	-1.0791	-0.4781	-2.9397
25	N	-0.4976	-0.8866	-3.8576
26	C	-3.2274	-0.5967	-1.7908
27	O	-3.4447	-1.7794	-1.6469
28	O	-4.1792	0.3310	-1.9550
29	C	-5.5375	-0.1410	-1.8173
30	H	-5.7403	-0.9353	-2.5390
31	H	-6.1655	0.7281	-2.0089
32	H	-5.6916	-0.5146	-0.8017
33	C	-0.1088	-1.1812	-0.3054
34	C	1.1552	-1.0672	0.2940
35	C	2.0013	-2.1616	0.4308
36	C	1.6230	-3.4412	-0.0186
37	C	0.3531	-3.5609	-0.6106
38	C	-0.4852	-2.4558	-0.7492
39	H	-1.4637	-2.6074	-1.1894
40	H	-0.0066	-4.5225	-0.9580
41	H	2.9843	-2.0101	0.8657
42	H	1.5023	-0.1042	0.6501
43	N	2.2071	4.8183	0.0484
44	C	2.0160	5.8584	-0.9748
45	C	2.4130	5.4116	1.3691
46	C	3.1872	6.8353	-0.9527
47	H	1.0788	6.4117	-0.7887
48	H	1.9460	5.4046	-1.9652
49	C	3.5705	6.4077	1.3117
50	H	1.5054	5.9281	1.7302
51	H	2.6643	4.6231	2.0854
52	H	3.0080	7.6585	-1.6507
53	H	4.1106	6.3114	-1.2496
54	O	3.3466	7.4188	0.3361
55	H	3.6772	6.9172	2.2738
56	H	4.5058	5.8679	1.0905
57	N	2.5168	-4.5258	0.0983
58	C	3.1172	-4.7550	1.4210
59	C	2.1470	-5.7888	-0.5385
60	C	4.2982	-5.7130	1.3002
61	H	2.3708	-5.1844	2.1124
62	H	3.4637	-3.8115	1.8469
63	C	3.3614	-6.7148	-0.5907
64	H	1.3266	-6.2940	0.0024
65	H	1.8112	-5.5932	-1.5615
66	H	4.6940	-5.9550	2.2911
67	H	5.0961	-5.2406	0.7037
68	O	3.9044	-6.9445	0.7049
69	H	3.0739	-7.6923	-0.9888
70	H	4.1277	-6.2747	-1.2496

71	C	-5.3428	-0.0718	3.6614
72	H	-5.4577	-1.0612	4.1154
73	H	-5.1339	0.6411	4.4697
74	H	-6.3048	0.2148	3.2213

SP_obn_an_m2c_MeProp
Energy (POTENTIAL) = -617.2852064 Eh

	Atom	X	Y	Z
1	O	0.8090	-1.3020	-1.2750
2	C	1.4950	-0.3587	-0.4890
3	H	1.7072	-0.7575	0.5213
4	H	2.4752	-0.1711	-0.9532
5	C	0.7711	0.9732	-0.3251
6	C	1.4749	2.1811	-0.2946
7	C	-0.6230	1.0114	-0.1681
8	C	0.8121	3.3974	-0.1049
9	H	2.5559	2.1769	-0.4277
10	C	-1.2796	2.2271	0.0188
11	H	-1.1778	0.0745	-0.1903
12	C	-0.5774	3.4416	0.0571
13	H	1.3824	4.3247	-0.0923
14	H	-2.3622	2.2369	0.1370
15	C	-1.3026	4.7488	0.2845
16	H	-2.2252	4.8037	-0.3054
17	H	-1.5894	4.8682	1.3381
18	H	-0.6781	5.6077	0.0157
19	O	-1.1671	-2.1440	0.1442
20	H	0.0159	-1.6556	-0.7142
21	C	-0.8122	-2.1073	1.4839
22	H	-0.5830	-1.0784	1.8388
23	H	-1.6172	-2.4824	2.1550
24	H	0.0865	-2.7162	1.7333
25	O	-0.8130	-4.6407	-0.4790
26	H	-1.0446	-3.6747	-0.2509
27	C	0.5944	-4.7057	-0.4438
28	H	0.9920	-4.8866	0.5748
29	H	0.9403	-5.5390	-1.0747
30	H	1.0625	-3.7806	-0.8125

SP_ar3m2obn_meoh
Energy (POTENTIAL) = -1847.3949624 Eh

	Atom	X	Y	Z
1	C	-0.0745	0.1728	-0.7600
2	C	-1.3072	-0.6175	-0.3106
3	C	-2.5502	-0.3408	-0.9091
4	C	-1.3029	-1.4934	0.7768
5	C	-3.7202	-0.9337	-0.4593
6	H	-2.6062	0.3793	-1.7189
7	C	-2.4721	-2.1101	1.2281
8	H	-0.3736	-1.7170	1.2891
9	C	-3.7114	-1.8468	0.6186
10	H	-4.6598	-0.6628	-0.9300
11	H	-2.3994	-2.8055	2.0559
12	C	1.2685	-0.3832	-0.2550
13	C	2.2624	0.4686	0.2451
14	C	1.5923	-1.7366	-0.3972
15	C	3.5091	-0.0170	0.6239
16	H	2.0657	1.5319	0.3205
17	C	2.8358	-2.2366	-0.0119
18	H	0.8586	-2.4251	-0.8060
19	C	3.8250	-1.3856	0.5160
20	H	4.2580	0.6825	0.9825
21	H	3.0194	-3.2996	-0.1161
22	C	0.0450	0.2723	-2.2904
23	C	0.6434	1.3940	-2.8793
24	C	-0.3377	-0.7861	-3.1218
25	C	0.8269	1.4621	-4.2596
26	H	0.9664	2.2179	-2.2527
27	C	-0.1455	-0.7159	-4.5024
28	H	-0.7965	-1.6715	-2.6931
29	C	0.4329	0.4109	-5.0993
30	H	1.2893	2.3472	-4.6913
31	H	-0.4555	-1.5521	-5.1251
32	N	5.1077	-1.8416	0.8901
33	C	5.5688	-1.4721	2.2386
34	C	5.4553	-3.2284	0.5799
35	C	7.0648	-1.7359	2.3698
36	H	5.0284	-2.0582	3.0025
37	H	5.3744	-0.4148	2.4277
38	C	6.9573	-3.4360	0.7675
39	H	4.9065	-3.9403	1.2222
40	H	5.1992	-3.4428	-0.4622
41	H	7.3956	-1.5437	3.3945
42	H	7.6184	-1.0716	1.6864
43	O	7.3746	-3.0979	2.0868
44	H	7.2143	-4.4883	0.6157

45	H	7.5055	-2.8269	0.0305
46	N	-4.9190	-2.4104	1.0727
47	C	-5.8035	-3.0098	0.0603
48	C	-4.8818	-3.2231	2.2888
49	C	-7.1904	-3.2453	0.6492
50	H	-5.3885	-3.9715	-0.2881
51	H	-5.8916	-2.3501	-0.8045
52	C	-6.3025	-3.4560	2.8001
53	H	-4.3923	-4.1970	2.1109
54	H	-4.3174	-2.6943	3.0626
55	H	-7.8238	-3.7689	-0.0726
56	H	-7.6564	-2.2772	0.8955
57	O	-7.1254	-4.0670	1.8115
58	H	-6.2861	-4.1325	3.6594
59	H	-6.7404	-2.4946	3.1142
60	O	-0.2284	1.5526	-0.3131
61	C	-0.5657	1.7486	1.0789
62	H	-1.6343	1.5475	1.2173
63	H	-0.0050	1.0438	1.7012
64	C	-0.2195	3.1705	1.4297
65	C	0.9364	3.4712	2.1581
66	C	-1.0343	4.2263	0.9976
67	C	1.2718	4.7953	2.4483
68	H	1.5807	2.6648	2.5005
69	C	-0.6932	5.5468	1.2846
70	H	-1.9258	4.0066	0.4164
71	C	0.4634	5.8549	2.0177
72	H	2.1749	5.0071	3.0162
73	H	-1.3349	6.3533	0.9365
74	C	0.8073	7.2871	2.3537
75	H	0.5394	7.9649	1.5358
76	H	0.2611	7.6241	3.2447
77	H	1.8758	7.4058	2.5609
78	C	0.6065	0.5048	-6.5968
79	H	-0.2201	1.0637	-7.0553
80	H	1.5332	1.0264	-6.8602
81	H	0.6281	-0.4865	-7.0614
82	C	-1.7971	3.9057	-2.5304
83	H	-2.6531	4.4214	-2.9772
84	H	-1.2230	4.6381	-1.9425
85	H	-1.1547	3.5307	-3.3399
86	O	-2.3060	2.8536	-1.7204
87	H	-1.5399	2.4071	-1.3031

obn_an_m2b
Energy (POTENTIAL) = -617.081930770 Eh

	Atom	X	Y	Z
1	O	0.3318	-1.5791	-0.7562
2	C	1.2062	-0.7867	-0.0310
3	H	1.2932	-1.1059	1.0365
4	H	2.2473	-0.8488	-0.4185
5	C	0.8176	0.6919	-0.0142
6	C	1.7591	1.7087	-0.2001
7	C	-0.5176	1.0689	0.2037
8	C	1.3884	3.0578	-0.1667
9	H	2.8000	1.4459	-0.3834
10	C	-0.8867	2.4121	0.2382
11	H	-1.2605	0.2904	0.3551
12	C	0.0592	3.4343	0.0539
13	H	2.1432	3.8268	-0.3220
14	H	-1.9288	2.6784	0.4098
15	C	-0.3529	4.8880	0.1072
16	H	-1.2367	5.0777	-0.5139
17	H	-0.6115	5.1927	1.1301
18	H	0.4506	5.5465	-0.2394
19	O	-1.8909	-2.0162	0.4508
20	H	-0.9720	-1.8421	0.0142
21	C	-2.8193	-2.0020	-0.6093
22	H	-2.6200	-2.7826	-1.3652
23	H	-3.8259	-2.1829	-0.2083
24	H	-2.8436	-1.0337	-1.1405
25	O	1.2071	-3.9924	-0.4606
26	H	0.8839	-3.0380	-0.6367
27	C	0.7028	-4.3323	0.8116
28	H	-0.3531	-4.0441	0.9367
29	H	0.7791	-5.4203	0.9523
30	H	1.2649	-3.8590	1.6403

ar3no2_rad_MeButa
Energy (POTENTIAL) = -1181.41081071 Eh

	Atom	X	Y	Z
1	C	-0.0032	-0.0021	0.4762
2	C	-0.0065	-1.2649	-0.2498
3	C	0.6479	-1.3995	-1.5019
4	C	-0.6571	-2.4100	0.2788
5	C	0.6551	-2.6026	-2.1879

6	H	1.1804	-0.5521	-1.9190
7	C	-0.6648	-3.6153	-0.4032
8	H	-1.1834	-2.3334	1.2234
9	C	-0.0055	-3.7036	-1.6334
10	H	1.1710	-2.7052	-3.1342
11	H	-1.1795	-4.4810	-0.0058
12	C	-0.0026	-0.0025	1.9391
13	C	-0.7100	0.9773	2.6748
14	C	0.7021	-0.9831	2.6742
15	C	-0.7122	0.9691	4.0634
16	H	-1.2843	1.7299	2.1437
17	C	0.7023	-0.9762	4.0640
18	H	1.2713	-1.7398	2.1437
19	C	-0.0026	-0.0026	4.7879
20	H	-1.2817	1.7244	4.5995
21	H	1.2651	-1.7364	4.5999
22	C	-0.0001	1.2616	-0.2481
23	C	0.6497	2.4063	0.2825
24	C	-0.6542	1.3979	-1.5003
25	C	0.6569	3.6127	-0.3976
26	H	1.1757	2.3285	1.2271
27	C	-0.6618	2.6021	-2.1843
28	H	-1.1861	0.5509	-1.9188
29	C	-0.0020	3.7025	-1.6279
30	H	-1.1775	2.7059	-3.1307
31	H	1.1710	4.4781	0.0013
32	C	0.0196	0.0201	6.2956
33	H	0.7611	0.7421	6.6629
34	H	-0.9514	0.3191	6.7050
35	H	0.2816	-0.9588	6.7092
36	N	-0.0019	4.9712	-2.3456
37	O	-0.5750	5.0227	-3.4394
38	O	0.5714	5.9351	-1.8262
39	N	-0.0061	-4.9710	-2.3532
40	O	-0.5797	-5.9356	-1.8353
41	O	0.5668	-5.0209	-3.4471

SP_cnoac_rad_MeProp
Energy (POTENTIAL) = -360.0932679 Eh

	Atom	X	Y	Z
1	C	-0.8647	0.7879	-0.0046
2	H	-0.7717	1.8680	0.0260
3	C	-2.1290	0.2091	-0.0663
4	N	-3.1997	-0.2665	-0.1178
5	C	0.3365	-0.0622	0.0193
6	O	0.3222	-1.2806	-0.0132
7	O	1.4440	0.6961	0.0821
8	C	2.6907	-0.0309	0.1122
9	H	2.7278	-0.6803	0.9907
10	H	3.4686	0.7299	0.1606
11	H	2.7964	-0.6370	-0.7913

SP_meoh_MeProp
Energy (POTENTIAL) = -115.7705042 Eh

	Atom	X	Y	Z
1	C	0.0191	0.0016	-0.6648
2	H	-0.9918	0.0065	-1.0828
3	H	0.5486	0.8921	-1.0361
4	H	0.5404	-0.8933	-1.0372
5	O	-0.1209	0.0013	0.7525
6	H	0.7714	0.0003	1.1314

ar3m2_rad_MeProp
Energy (POTENTIAL) = -1345.62238829 Eh

	Atom	X	Y	Z
1	C	-0.0911	-0.0165	1.2406
2	C	-0.0198	-1.2860	0.5256
3	C	0.7059	-1.4233	-0.6855
4	C	-0.6689	-2.4522	0.9923
5	C	0.7772	-2.6250	-1.3687
6	H	1.2519	-0.5685	-1.0723
7	C	-0.6134	-3.6588	0.3040
8	H	-1.2601	-2.3988	1.9013
9	C	0.1133	-3.7803	-0.8976
10	H	1.3887	-2.6837	-2.2638
11	H	-1.1690	-4.5025	0.6973
12	C	-0.1943	0.0008	2.7002
13	C	-0.9347	0.9979	3.3798
14	C	0.4355	-0.9791	3.5049
15	C	-1.0375	1.0089	4.7664
16	H	-1.4522	1.7567	2.8011
17	C	0.3298	-0.9589	4.8911
18	H	1.0264	-1.7543	3.0267
19	C	-0.4066	0.0346	5.5548
20	H	-1.6297	1.7835	5.2493
21	H	0.8360	-1.7258	5.4739

22	C	-0.0654	1.2422	0.4989
23	C	0.5417	2.4075	1.0189
24	C	-0.6488	1.3673	-0.7871
25	C	0.5645	3.6083	0.3181
26	H	1.0120	2.3659	1.9965
27	C	-0.6252	2.5581	-1.4932
28	H	-1.1368	0.5050	-1.2307
29	C	-0.0176	3.7173	-0.9609
30	H	-1.0705	2.5893	-2.4827
31	C	-0.3209	-6.2031	-0.9862
32	C	-0.0303	-4.9674	-3.0464
33	C	0.2127	-7.4383	-1.7111
34	H	-1.4249	-6.2088	-1.0197
35	H	-0.0110	-6.2524	0.0620
36	C	0.4962	-6.2419	-3.6980
37	H	-1.1143	-4.8829	-3.2378
38	H	0.4578	-4.1041	-3.5019
39	H	-0.2605	-8.3411	-1.3142
40	H	1.3015	-7.5109	-1.5556
41	H	0.2273	-6.2648	-4.7581
42	H	1.5944	-6.2761	-3.6084
43	N	0.2299	-4.9950	-1.5987
44	O	-0.0803	-7.4005	-3.1035
45	N	0.0247	4.9027	-1.7197
46	C	-1.2389	5.3721	-2.3093
47	C	0.8174	6.0213	-1.2108
48	C	-0.9584	6.4208	-3.3805
49	H	-1.8833	5.8131	-1.5288
50	H	-1.7805	4.5378	-2.7582
51	C	1.0253	7.0523	-2.3194
52	H	0.3287	6.5068	-0.3473
53	H	1.7963	5.6550	-0.8870
54	H	-1.8967	6.8355	-3.7603
55	H	-0.4098	5.9566	-4.2165
56	O	-0.2126	7.5122	-2.8508
57	H	1.5441	7.9305	-1.9241
58	H	1.6390	6.6086	-3.1204
59	H	1.0366	4.4670	0.7817
60	C	-0.4919	0.0700	7.0615
61	H	0.3452	0.6358	7.4933
62	H	-1.4161	0.5510	7.3998
63	H	-0.4548	-0.9377	7.4897

SP_obn_an_m1_PMMA
Energy (POTENTIAL) = -501.4842842 Eh

	Atom	X	Y	Z
1	O	0.9631	-2.3802	-1.4194
2	C	1.6339	-1.4293	-0.7294
3	H	2.0100	-1.7538	0.2844
4	H	2.5779	-1.1006	-1.2451
5	C	0.8654	-0.1221	-0.4449
6	C	1.5008	0.9937	0.1188
7	C	-0.4957	-0.0178	-0.7417
8	C	0.8015	2.1711	0.3801
9	H	2.5650	0.9382	0.3516
10	C	-1.2001	1.1604	-0.4743
11	H	-0.9901	-0.8893	-1.1591
12	C	-0.5683	2.2753	0.0890
13	H	1.3212	3.0256	0.8132
14	H	-2.2627	1.2162	-0.7093
15	C	-1.3334	3.5416	0.4036
16	H	-2.2672	3.5982	-0.1671
17	H	-1.5994	3.5999	1.4685
18	H	-0.7437	4.4374	0.1732
19	O	-1.0846	-3.2821	-0.2279
20	H	-0.2222	-2.9196	-0.7085
21	C	-0.9369	-2.9900	1.1327
22	H	-0.7794	-1.9134	1.3318
23	H	-1.8483	-3.2894	1.6753
24	H	-0.0919	-3.5273	1.6101

SP_ar3no2_cat
Energy (POTENTIAL) = -1181.5423022 Eh

	Atom	X	Y	Z
1	C	-0.0031	0.0008	0.5316
2	C	0.0177	-1.2540	-0.2144
3	C	0.7636	-1.3574	-1.4115
4	C	-0.7211	-2.3709	0.2405
5	C	0.7948	-2.5502	-2.1196
6	H	1.3501	-0.5149	-1.7588
7	C	-0.7153	-3.5583	-0.4784
8	H	-1.3337	-2.2873	1.1304
9	C	0.0494	-3.6275	-1.6415
10	H	1.3804	-2.6515	-3.0238
11	H	-1.2929	-4.4139	-0.1537
12	C	-0.0058	0.0049	1.9551

13	C	-0.5750	1.0920	2.6858
14	C	0.5590	-1.0789	2.6959
15	C	-0.5872	1.0805	4.0653
16	H	-1.0621	1.8994	2.1517
17	C	0.5633	-1.0588	4.0742
18	H	1.0446	-1.8920	2.1691
19	C	-0.0119	0.0149	4.7880
20	H	-1.0566	1.8973	4.6041
21	H	1.0245	-1.8750	4.6216
22	C	-0.0204	1.2520	-0.2206
23	C	0.7165	2.3711	0.2320
24	C	-0.7616	1.3500	-1.4211
25	C	0.7131	3.5554	-0.4922
26	H	1.3259	2.2916	1.1245
27	C	-0.7903	2.5396	-2.1346
28	H	-1.3468	0.5059	-1.7668
29	C	-0.0471	3.6192	-1.6584
30	H	-1.3726	2.6368	-3.0415
31	H	1.2893	4.4126	-0.1690
32	C	0.0162	0.0285	6.2867
33	H	1.0019	0.3709	6.6310
34	H	-0.7349	0.7055	6.7012
35	H	-0.1357	-0.9751	6.6964
36	N	-0.0618	4.8818	-2.4257
37	O	-0.7384	4.9195	-3.4519
38	O	0.6027	5.8218	-1.9933
39	N	0.0667	-4.8934	-2.4032
40	O	0.7471	-4.9358	-3.4267
41	O	-0.5996	-5.8314	-1.9692

SP_obn_rad_MeButa
Energy (POTENTIAL) = -385.5465302 Eh

	Atom	X	Y	Z
1	O	0.0687	-2.9249	-0.8990
2	C	0.1192	-2.5520	0.4298
3	H	-0.7457	-2.9123	1.0100
4	H	1.0528	-2.8608	0.9272
5	C	0.0696	-1.0300	0.2991
6	C	1.2510	-0.2973	0.1273
7	C	-1.1623	-0.3656	0.2426
8	C	1.2007	1.0851	-0.0336
9	H	2.2083	-0.8116	0.1323
10	C	-1.2053	1.0170	0.0814
11	H	-2.0843	-0.9329	0.3374
12	C	-0.0263	1.7660	-0.0555
13	H	2.1252	1.6457	-0.1488
14	H	-2.1666	1.5245	0.0564
15	C	-0.0759	3.2675	-0.1995
16	H	-0.9944	3.5922	-0.6993
17	H	-0.0507	3.7546	0.7845
18	H	0.7788	3.6442	-0.7709

obn_an_PMMA
Energy (POTENTIAL) = -385.551865436 Eh

	Atom	X	Y	Z
1	O	-1.0854	-3.1799	0.2059
2	C	0.0820	-2.5754	0.3626
3	H	0.6062	-2.7347	1.3705
4	H	0.9185	-2.8886	-0.3571
5	C	0.0745	-1.0269	0.2234
6	C	1.2240	-0.2395	0.3809
7	C	-1.1235	-0.3771	-0.0721
8	C	1.1749	1.1477	0.2503
9	H	2.1739	-0.7266	0.6091
10	C	-1.1794	1.0145	-0.2049
11	H	-1.9967	-1.0144	-0.1911
12	C	-0.0339	1.8028	-0.0441
13	H	2.0827	1.7384	0.3768
14	H	-2.1279	1.5001	-0.4368
15	C	-0.0844	3.3099	-0.1674
16	H	-1.0573	3.6486	-0.5412
17	H	0.0849	3.8039	0.7996
18	H	0.6855	3.6868	-0.8534

ar3no2obn
Energy (POTENTIAL) = -1566.94509823 Eh

	Atom	X	Y	Z
1	C	-0.3728	-0.2375	-0.3861
2	C	-1.7316	0.1290	0.2352
3	C	-2.6607	0.8126	-0.5668
4	C	-2.0168	-0.0495	1.5948
5	C	-3.8589	1.2768	-0.0402
6	H	-2.4395	0.9816	-1.6147
7	C	-3.2142	0.4062	2.1419
8	H	-1.3038	-0.5422	2.2451
9	C	-4.1226	1.0591	1.3132

10	H	-4.5795	1.7989	-0.6567
11	H	-3.4395	0.2650	3.1913
12	C	0.4400	-1.2798	0.4095
13	C	1.8232	-1.1104	0.5629
14	C	-0.1553	-2.4574	0.8902
15	C	2.5951	-2.0731	1.2052
16	H	2.2983	-0.2195	0.1710
17	C	0.5999	-3.4311	1.5323
18	H	-1.2200	-2.6201	0.7652
19	C	1.9711	-3.2229	1.6867
20	H	3.6624	-1.9415	1.3311
21	H	0.1425	-4.3371	1.9087
22	C	-0.5220	-0.8185	-1.8040
23	C	0.3982	-0.4869	-2.8041
24	C	-1.5255	-1.7502	-2.0995
25	C	0.3062	-1.0671	-4.0698
26	H	1.1844	0.2281	-2.5920
27	C	-1.6096	-2.3274	-3.3651
28	H	-2.2560	-2.0244	-1.3440
29	C	-0.6974	-1.9949	-4.3760
30	H	1.0312	-0.7928	-4.8327
31	H	-2.3994	-3.0462	-3.5704
32	O	0.3805	0.9723	-0.5404
33	C	0.5198	1.8145	0.6222
34	H	-0.4047	2.3880	0.7632
35	H	0.6819	1.2103	1.5225
36	C	1.6940	2.7199	0.3691
37	C	2.9122	2.5275	1.0283
38	C	1.5975	3.7495	-0.5766
39	C	4.0077	3.3491	0.7535
40	H	3.0073	1.7292	1.7608
41	C	2.6931	4.5642	-0.8526
42	H	0.6584	3.9058	-1.1020
43	C	3.9168	4.3805	-0.1892
44	H	4.9460	3.1840	1.2778
45	H	2.6007	5.3569	-1.5916
46	C	5.0899	5.2903	-0.4688
47	H	5.1467	5.5544	-1.5305
48	H	5.0013	6.2295	0.0932
49	H	6.0376	4.8242	-0.1805
50	C	-0.8120	-2.5957	-5.7568
51	H	-1.4712	-1.9916	-6.3942
52	H	0.1626	-2.6471	-6.2533
53	H	-1.2329	-3.6061	-5.7203
54	N	-5.3870	1.5392	1.8797
55	O	-6.1766	2.1188	1.1303
56	O	-5.6004	1.3409	3.0780
57	N	2.7742	-4.2401	2.3671
58	O	2.2032	-5.2524	2.7817
59	O	3.9847	-4.0379	2.4948

SP_ar3m2cnoac_PMMA
Energy (POTENTIAL) = -1706.1262412 Eh

	Atom	X	Y	Z
1	C	-1.0607	0.0298	-0.3977
2	C	-0.2468	1.3388	-0.2834
3	C	-0.1348	2.0307	0.9266
4	C	0.6497	3.1790	1.0512
5	C	1.3784	3.6812	-0.0401
6	C	1.2905	2.9683	-1.2532
7	C	0.5029	1.8307	-1.3656
8	H	0.5068	1.3058	-2.3154
9	H	1.8730	3.2890	-2.1110
10	H	0.6698	3.6853	2.0092
11	H	-0.6753	1.6809	1.7991
12	C	-2.1538	-0.0160	0.6954
13	C	-2.2619	-1.0614	1.6155
14	C	-3.2882	-1.0809	2.5641
15	C	-4.2408	-0.0607	2.6289
16	C	-4.1260	0.9954	1.7118
17	C	-3.1009	1.0212	0.7719
18	H	-3.0342	1.8601	0.0863
19	H	-4.8464	1.8100	1.7382
20	H	-3.3467	-1.9125	3.2625
21	H	-1.5488	-1.8766	1.5931
22	C	-1.8398	0.0723	-1.8155
23	H	-2.0147	1.1263	-2.0430
24	C	-1.0791	-0.4781	-2.9397
25	N	-0.4976	-0.8866	-3.8576
26	C	-3.2274	-0.5967	-1.7908
27	O	-3.4447	-1.7794	-1.6469
28	O	-4.1792	0.3310	-1.9550
29	C	-5.5375	-0.1410	-1.8173
30	H	-5.7403	-0.9353	-2.5390
31	H	-6.1655	0.7281	-2.0089
32	H	-5.6916	-0.5146	-0.8017

33	C	-0.1088	-1.1812	-0.3054
34	C	1.1552	-1.0672	0.2940
35	C	2.0013	-2.1616	0.4308
36	C	1.6230	-3.4412	-0.0186
37	C	0.3531	-3.5609	-0.6106
38	C	-0.4852	-2.4558	-0.7492
39	H	-1.4637	-2.6074	-1.1894
40	H	-0.0066	-4.5225	-0.9580
41	H	2.9843	-2.0101	0.8657
42	H	1.5023	-0.1042	0.6501
43	N	2.2071	4.8183	0.0484
44	C	2.0160	5.8584	-0.9748
45	C	2.4130	5.4116	1.3691
46	C	3.1872	6.8353	-0.9527
47	H	1.0788	6.4117	-0.7887
48	H	1.9460	5.4046	-1.9652
49	C	3.5705	6.4077	1.3117
50	H	1.5054	5.9281	1.7302
51	H	2.6643	4.6231	2.0854
52	H	3.0080	7.6585	-1.6507
53	H	4.1106	6.3114	-1.2496
54	O	3.3466	7.4188	0.3361
55	H	3.6772	6.9172	2.2738
56	H	4.5058	5.8679	1.0905
57	N	2.5168	-4.5258	0.0983
58	C	3.1172	-4.7550	1.4210
59	C	2.1470	-5.7888	-0.5385
60	C	4.2982	-5.7130	1.3002
61	H	2.3708	-5.1844	2.1124
62	H	3.4637	-3.8115	1.8469
63	C	3.3614	-6.7148	-0.5907
64	H	1.3266	-6.2940	0.0024
65	H	1.8112	-5.5932	-1.5615
66	H	4.6940	-5.9550	2.2911
67	H	5.0961	-5.2406	0.7037
68	O	3.9044	-6.9445	0.7049
69	H	3.0739	-7.6923	-0.9888
70	H	4.1277	-6.2747	-1.2496
71	C	-5.3428	-0.0718	3.6614
72	H	-5.4577	-1.0612	4.1154
73	H	-5.1339	0.6411	4.4697
74	H	-6.3048	0.2148	3.2213

SP_obn_an_m2_MeButa
Energy (POTENTIAL) = -617.2860991 Eh

	Atom	X	Y	Z
1	O	0.4605	-1.6841	-1.2594
2	C	1.2317	-0.9335	-0.4042
3	H	1.2784	-1.3465	0.6344
4	H	2.3021	-0.9018	-0.7195
5	C	0.7879	0.5244	-0.2555
6	C	1.5827	1.4590	0.4242
7	C	-0.4273	0.9632	-0.7868
8	C	1.1758	2.7840	0.5700
9	H	2.5394	1.1425	0.8396
10	C	-0.8381	2.2918	-0.6376
11	H	-1.0505	0.2388	-1.3010
12	C	-0.0476	3.2260	0.0411
13	H	1.8155	3.4906	1.0971
14	H	-1.7911	2.6085	-1.0590
15	C	-0.4970	4.6584	0.2227
16	H	-1.3145	4.9120	-0.4610
17	H	-0.8566	4.8410	1.2448
18	H	0.3235	5.3639	0.0430
19	O	-1.9834	-2.0534	-0.4440
20	H	-1.0176	-1.9096	-0.7515
21	C	-2.0371	-1.6999	0.9174
22	H	-1.6939	-0.6679	1.1037
23	H	-3.0789	-1.7698	1.2615
24	H	-1.4345	-2.3683	1.5583
25	O	1.2367	-4.1036	-0.7494
26	H	0.9656	-3.1602	-1.0296
27	C	0.3609	-4.4407	0.3009
28	H	-0.6881	-4.1981	0.0700
29	H	0.4293	-5.5213	0.4955
30	H	0.6059	-3.9244	1.2502

obn_rad_PMMA
Energy (POTENTIAL) = -385.437685028 Eh

	Atom	X	Y	Z
1	O	0.0682	-2.9286	-0.8982
2	C	0.1192	-2.5520	0.4292
3	H	-0.7454	-2.9113	1.0108
4	H	1.0530	-2.8600	0.9271
5	C	0.0696	-1.0300	0.2985
6	C	1.2508	-0.2972	0.1274

7	C	-1.1620	-0.3655	0.2424
8	C	1.2006	1.0853	-0.0334
9	H	2.2080	-0.8117	0.1317
10	C	-1.2051	1.0171	0.0814
11	H	-2.0840	-0.9330	0.3363
12	C	-0.0262	1.7661	-0.0552
13	H	2.1251	1.6459	-0.1486
14	H	-2.1665	1.5246	0.0561
15	C	-0.0759	3.2678	-0.1990
16	H	-0.9943	3.5925	-0.6990
17	H	-0.0506	3.7550	0.7849
18	H	0.7785	3.6446	-0.7707

uvvisfrag1_rad
Energy (POTENTIAL) = -1345.344542 Eh

	Atom	X	Y	Z
1	C	-0.0893	-0.0160	1.2397
2	C	-0.0201	-1.2856	0.5244
3	C	0.7034	-1.4243	-0.6879
4	C	-0.6695	-2.4517	0.9918
5	C	0.7729	-2.6261	-1.3714
6	H	1.2491	-0.5702	-1.0769
7	C	-0.6162	-3.6585	0.3037
8	H	-1.2596	-2.3986	1.9015
9	C	0.1091	-3.7817	-0.8992
10	H	1.3821	-2.6833	-2.2680
11	H	-1.1722	-4.5011	0.6987
12	C	-0.1902	0.0007	2.6998
13	C	-0.9283	0.9983	3.3815
14	C	0.4399	-0.9802	3.5032
15	C	-1.0287	1.0089	4.7684
16	H	-1.4461	1.7584	2.8046
17	C	0.3368	-0.9606	4.8898
18	H	1.0289	-1.7563	3.0242
19	C	-0.3973	0.0335	5.5555
20	H	-1.6191	1.7841	5.2526
21	H	0.8431	-1.7285	5.4712
22	C	-0.0642	1.2429	0.4984
23	C	0.5416	2.4091	1.0185
24	C	-0.6478	1.3687	-0.7877
25	C	0.5629	3.6105	0.3186
26	H	1.0122	2.3686	1.9961
27	C	-0.6264	2.5599	-1.4932
28	H	-1.1352	0.5066	-1.2324
29	C	-0.0198	3.7203	-0.9607
30	H	-1.0731	2.5900	-2.4820
31	C	-0.3426	-6.2012	-0.9911
32	C	-0.0123	-4.9702	-3.0497
33	C	0.1908	-7.4414	-1.7070
34	H	-1.4459	-6.1975	-1.0382
35	H	-0.0464	-6.2532	0.0607
36	C	0.5139	-6.2498	-3.6910
37	H	-1.0924	-4.8774	-3.2576
38	H	0.4901	-4.1128	-3.5003
39	H	-0.2955	-8.3392	-1.3148
40	H	1.2767	-7.5232	-1.5366
41	H	0.2618	-6.2711	-4.7552
42	H	1.6100	-6.2937	-3.5839
43	N	0.2246	-4.9969	-1.5976
44	O	-0.0821	-7.4033	-3.1041
45	N	0.0220	4.9057	-1.7176
46	C	-1.2378	5.3673	-2.3223
47	C	0.8034	6.0296	-1.2010
48	C	-0.9528	6.4185	-3.3895
49	H	-1.8938	5.8030	-1.5487
50	H	-1.7688	4.5306	-2.7790
51	C	1.0156	7.0626	-2.3065
52	H	0.3037	6.5103	-0.3414
53	H	1.7819	5.6703	-0.8689
54	H	-1.8900	6.8261	-3.7793
55	H	-0.3915	5.9595	-4.2197
56	O	-0.2203	7.5154	-2.8504
57	H	1.5252	7.9431	-1.9048
58	H	1.6397	6.6238	-3.1020
59	H	1.0343	4.4689	0.7832
60	C	-0.4800	0.0682	7.0623
61	H	0.3586	0.6329	7.4926
62	H	-1.4029	0.5505	7.4022
63	H	-0.4429	-0.9397	7.4898

SP_ar3no2_rad_MeButa
Energy (POTENTIAL) = -1181.7269784 Eh

	Atom	X	Y	Z
1	C	-0.0032	-0.0021	0.4762
2	C	-0.0065	-1.2649	-0.2498
3	C	0.6479	-1.3995	-1.5019

4	C	-0.6571	-2.4100	0.2788
5	C	0.6551	-2.6026	-2.1879
6	H	1.1804	-0.5521	-1.9190
7	C	-0.6648	-3.6153	-0.4032
8	H	-1.1834	-2.3334	1.2234
9	C	-0.0055	-3.7036	-1.6334
10	H	1.1710	-2.7052	-3.1342
11	H	-1.1795	-4.4810	-0.0058
12	C	-0.0026	-0.0025	1.9391
13	C	-0.7100	0.9773	2.6748
14	C	0.7021	-0.9831	2.6742
15	C	-0.7122	0.9691	4.0634
16	H	-1.2843	1.7299	2.1437
17	C	0.7023	-0.9762	4.0640
18	H	1.2713	-1.7398	2.1437
19	C	-0.0026	-0.0026	4.7879
20	H	-1.2817	1.7244	4.5995
21	H	1.2651	-1.7364	4.5999
22	C	-0.0001	1.2616	-0.2481
23	C	0.6497	2.4063	0.2825
24	C	-0.6542	1.3979	-1.5003
25	C	0.6569	3.6127	-0.3976
26	H	1.1757	2.3285	1.2271
27	C	-0.6618	2.6021	-2.1843
28	H	-1.1861	0.5509	-1.9188
29	C	-0.0020	3.7025	-1.6279
30	H	-1.1775	2.7059	-3.1307
31	H	1.1710	4.4781	0.0013
32	C	0.0196	0.0201	6.2956
33	H	0.7611	0.7421	6.6629
34	H	-0.9514	0.3191	6.7050
35	H	0.2816	-0.9588	6.7092
36	N	-0.0019	4.9712	-2.3456
37	O	-0.5750	5.0227	-3.4394
38	O	0.5714	5.9351	-1.8262
39	N	-0.0061	-4.9710	-2.3532
40	O	-0.5798	-5.9356	-1.8353
41	O	0.5668	-5.0209	-3.4471

ar3m2_rad_PMMA
Energy (POTENTIAL) = -1345.62085794 Eh

	Atom	X	Y	Z
1	C	-0.0914	-0.0170	1.2400
2	C	-0.0190	-1.2865	0.5254
3	C	0.7079	-1.4236	-0.6849
4	C	-0.6680	-2.4526	0.9923
5	C	0.7801	-2.6255	-1.3675
6	H	1.2543	-0.5685	-1.0707
7	C	-0.6114	-3.6593	0.3042
8	H	-1.2598	-2.3986	1.9008
9	C	0.1158	-3.7803	-0.8966
10	H	1.3930	-2.6855	-2.2617
11	H	-1.1668	-4.5035	0.6969
12	C	-0.1963	0.0008	2.6994
13	C	-0.9385	0.9974	3.3774
14	C	0.4338	-0.9780	3.5050
15	C	-1.0430	1.0088	4.7637
16	H	-1.4562	1.7551	2.7973
17	C	0.3264	-0.9572	4.8910
18	H	1.0263	-1.7523	3.0276
19	C	-0.4120	0.0357	5.5532
20	H	-1.6368	1.7830	5.2456
21	H	0.8329	-1.7231	5.4749
22	C	-0.0650	1.2415	0.4983
23	C	0.5417	2.4065	1.0190
24	C	-0.6473	1.3665	-0.7881
25	C	0.5652	3.6073	0.3181
26	H	1.0111	2.3643	1.9970
27	C	-0.6226	2.5571	-1.4942
28	H	-1.1349	0.5040	-1.2317
29	C	-0.0155	3.7160	-0.9612
30	H	-1.0661	2.5889	-2.4846
31	C	-0.3073	-6.2052	-0.9823
32	C	-0.0430	-4.9673	-3.0437
33	C	0.2259	-7.4373	-1.7127
34	H	-1.4116	-6.2167	-1.0067
35	H	0.0116	-6.2523	0.0635
36	C	0.4834	-6.2388	-3.7016
37	H	-1.1295	-4.8885	-3.2239
38	H	0.4354	-4.1003	-3.5028
39	H	-0.2388	-8.3432	-1.3125
40	H	1.3166	-7.5041	-1.5673
41	H	0.2033	-6.2634	-4.7588
42	H	1.5827	-6.2668	-3.6236
43	N	0.2328	-4.9948	-1.5992
44	O	-0.0806	-7.4003	-3.1018

45	N	0.0267	4.9016	-1.7204
46	C	-1.2387	5.3748	-2.3024
47	C	0.8243	6.0179	-1.2146
48	C	-0.9609	6.4232	-3.3747
49	H	-1.8778	5.8175	-1.5184
50	H	-1.7849	4.5416	-2.7480
51	C	1.0295	7.0488	-2.3238
52	H	0.3407	6.5047	-0.3488
53	H	1.8034	5.6481	-0.8950
54	H	-1.8997	6.8413	-3.7496
55	H	-0.4181	5.9574	-4.2138
56	O	-0.2094	7.5119	-2.8487
57	H	1.5522	7.9259	-1.9311
58	H	1.6384	6.6036	-3.1277
59	H	1.0369	4.4661	0.7819
60	C	-0.4991	0.0718	7.0598
61	H	0.3367	0.6388	7.4925
62	H	-1.4244	0.5517	7.3968
63	H	-0.4617	-0.9357	7.4886

SP_obn_an_m1_MeProp
Energy (POTENTIAL) = -501.4936177 Eh

	Atom	X	Y	Z
1	O	0.9636	-2.3763	-1.4229
2	C	1.6327	-1.4273	-0.7241
3	H	2.0001	-1.7585	0.2897
4	H	2.5807	-1.1014	-1.2325
5	C	0.8647	-0.1203	-0.4416
6	C	1.5017	0.9973	0.1171
7	C	-0.4972	-0.0169	-0.7347
8	C	0.8025	2.1752	0.3768
9	H	2.5664	0.9434	0.3471
10	C	-1.2015	1.1621	-0.4694
11	H	-0.9936	-0.8892	-1.1479
12	C	-0.5681	2.2788	0.0888
13	H	1.3234	3.0308	0.8057
14	H	-2.2645	1.2170	-0.7022
15	C	-1.3317	3.5468	0.4000
16	H	-2.2721	3.5965	-0.1599
17	H	-1.5843	3.6142	1.4674
18	H	-0.7458	4.4409	0.1545
19	O	-1.0783	-3.2874	-0.2338
20	H	-0.2167	-2.9164	-0.7112
21	C	-0.9380	-3.0004	1.1300
22	H	-0.7923	-1.9233	1.3339
23	H	-1.8478	-3.3118	1.6678
24	H	-0.0892	-3.5314	1.6067

obn_an_m2_PMMA
Energy (POTENTIAL) = -617.063491345 Eh

	Atom	X	Y	Z
1	O	0.4605	-1.6871	-1.2571
2	C	1.2377	-0.9344	-0.4117
3	H	1.2984	-1.3453	0.6276
4	H	2.3048	-0.8980	-0.7386
5	C	0.7912	0.5226	-0.2587
6	C	1.5832	1.4571	0.4243
7	C	-0.4240	0.9603	-0.7909
8	C	1.1739	2.7811	0.5723
9	H	2.5397	1.1409	0.8406
10	C	-0.8370	2.2878	-0.6393
11	H	-1.0445	0.2352	-1.3073
12	C	-0.0493	3.2219	0.0425
13	H	1.8116	3.4879	1.1022
14	H	-1.7902	2.6038	-1.0613
15	C	-0.5020	4.6530	0.2269
16	H	-1.3157	4.9078	-0.4611
17	H	-0.8686	4.8318	1.2472
18	H	0.3182	5.3608	0.0545
19	O	-1.9864	-2.0480	-0.4428
20	H	-1.0205	-1.9087	-0.7520
21	C	-2.0358	-1.6934	0.9174
22	H	-1.6885	-0.6624	1.1032
23	H	-3.0773	-1.7593	1.2640
24	H	-1.4344	-2.3634	1.5583
25	O	1.2406	-4.1059	-0.7408
26	H	0.9704	-3.1651	-1.0289
27	C	0.3567	-4.4359	0.3037
28	H	-0.6900	-4.1900	0.0654
29	H	0.4195	-5.5164	0.5027
30	H	0.5972	-3.9178	1.2538

ar3m2obn_meoh_PMMA
Energy (POTENTIAL) = -1846.89659716 Eh

	Atom	X	Y	Z
1	C	-0.0763	0.1740	-0.7612

2	C	-1.3083	-0.6167	-0.3101
3	C	-2.5535	-0.3302	-0.8996
4	C	-1.2999	-1.5019	0.7695
5	C	-3.7220	-0.9234	-0.4473
6	H	-2.6124	0.3992	-1.7009
7	C	-2.4684	-2.1193	1.2215
8	H	-0.3679	-1.7317	1.2743
9	C	-3.7094	-1.8462	0.6213
10	H	-4.6645	-0.6427	-0.9066
11	H	-2.3944	-2.8227	2.0426
12	C	1.2672	-0.3803	-0.2556
13	C	2.2566	0.4718	0.2524
14	C	1.5953	-1.7315	-0.4050
15	C	3.5034	-0.0127	0.6319
16	H	2.0563	1.5343	0.3306
17	C	2.8385	-2.2303	-0.0175
18	H	0.8648	-2.4183	-0.8225
19	C	3.8229	-1.3793	0.5179
20	H	4.2518	0.6860	0.9935
21	H	3.0267	-3.2921	-0.1274
22	C	0.0424	0.2721	-2.2914
23	C	0.6556	1.3867	-2.8782
24	C	-0.3554	-0.7792	-3.1239
25	C	0.8385	1.4547	-4.2581
26	H	0.9899	2.2043	-2.2493
27	C	-0.1639	-0.7086	-4.5043
28	H	-0.8263	-1.6586	-2.6959
29	C	0.4291	0.4109	-5.0991
30	H	1.3131	2.3339	-4.6888
31	H	-0.4866	-1.5389	-5.1287
32	N	5.1064	-1.8330	0.8951
33	C	5.5535	-1.4828	2.2523
34	C	5.4667	-3.2108	0.5647
35	C	7.0513	-1.7356	2.3891
36	H	5.0138	-2.0866	3.0033
37	H	5.3461	-0.4306	2.4569
38	C	6.9695	-3.4087	0.7589
39	H	4.9208	-3.9385	1.1923
40	H	5.2174	-3.4089	-0.4826
41	H	7.3748	-1.5590	3.4192
42	H	7.6026	-1.0540	1.7207
43	O	7.3746	-3.0885	2.0853
44	H	7.2377	-4.4563	0.5932
45	H	7.5164	-2.7832	0.0346
46	N	-4.9175	-2.4106	1.0761
47	C	-5.7984	-3.0145	0.0644
48	C	-4.8810	-3.2189	2.2938
49	C	-7.1867	-3.2480	0.6519
50	H	-5.3830	-3.9781	-0.2794
51	H	-5.8826	-2.3578	-0.8033
52	C	-6.3028	-3.4499	2.8040
53	H	-4.3924	-4.1945	2.1205
54	H	-4.3159	-2.6867	3.0652
55	H	-7.8193	-3.7759	-0.0678
56	H	-7.6530	-2.2785	0.8924
57	O	-7.1232	-4.0639	1.8170
58	H	-6.2891	-4.1238	3.6656
59	H	-6.7401	-2.4866	3.1141
60	O	-0.2301	1.5536	-0.3163
61	C	-0.5737	1.7521	1.0731
62	H	-1.6444	1.5586	1.2054
63	H	-0.0216	1.0430	1.6987
64	C	-0.2185	3.1712	1.4268
65	C	0.9262	3.4610	2.1763
66	C	-1.0145	4.2344	0.9782
67	C	1.2693	4.7815	2.4724
68	H	1.5557	2.6478	2.5308
69	C	-0.6653	5.5510	1.2714
70	H	-1.8972	4.0225	0.3807
71	C	0.4799	5.8483	2.0261
72	H	2.1636	4.9851	3.0573
73	H	-1.2923	6.3638	0.9110
74	C	0.8310	7.2773	2.3684
75	H	0.5871	7.9573	1.5447
76	H	0.2706	7.6209	3.2480
77	H	1.8964	7.3861	2.5965
78	C	0.6015	0.5061	-6.5967
79	H	-0.2178	1.0773	-7.0532
80	H	1.5355	1.0146	-6.8608
81	H	0.6092	-0.4842	-7.0641
82	C	-1.8173	3.8753	-2.5647
83	H	-2.6788	4.3883	-3.0043
84	H	-1.2191	4.6182	-2.0140
85	H	-1.2008	3.4689	-3.3793
86	O	-2.3173	2.8554	-1.7117
87	H	-1.5458	2.4078	-1.3065

SP_obn_an_m2b_MeProp
Energy (POTENTIAL) = -617.286731 Eh

	Atom	X	Y	Z
1	O	0.0781	-1.4549	-1.0836
2	C	1.1137	-0.7193	-0.5600
3	H	1.5053	-1.1207	0.4072
4	H	2.0116	-0.7216	-1.2254
5	C	0.7837	0.7544	-0.3015
6	C	1.7391	1.6278	0.2385
7	C	-0.4763	1.2721	-0.6085
8	C	1.4432	2.9710	0.4651
9	H	2.7312	1.2480	0.4827
10	C	-0.7749	2.6189	-0.3800
11	H	-1.2193	0.5941	-1.0149
12	C	0.1755	3.4926	0.1605
13	H	2.2039	3.6283	0.8839
14	H	-1.7656	2.9980	-0.6270
15	C	-0.1548	4.9420	0.4380
16	H	-1.0005	5.2839	-0.1689
17	H	-0.4267	5.0980	1.4912
18	H	0.6988	5.5973	0.2277
19	O	-2.0446	-1.6900	0.3965
20	H	-1.1859	-1.5234	-0.1287
21	C	-2.6920	-2.7431	-0.2833
22	H	-2.0030	-3.5688	-0.5182
23	H	-3.4980	-3.1387	0.3515
24	H	-3.1566	-2.4197	-1.2345
25	O	0.5564	-3.9496	-0.5130
26	H	0.3951	-2.9895	-0.8151
27	C	0.5454	-3.9070	0.8975
28	H	-0.2830	-3.2975	1.2877
29	H	0.4318	-4.9292	1.2872
30	H	1.4835	-3.4997	1.3215

SP_obn_rad_MeProp
Energy (POTENTIAL) = -385.5466909 Eh

	Atom	X	Y	Z
1	O	0.0688	-2.9243	-0.8991
2	C	0.1192	-2.5520	0.4300
3	H	-0.7457	-2.9124	1.0100
4	H	1.0528	-2.8609	0.9273
5	C	0.0696	-1.0300	0.2991
6	C	1.2510	-0.2973	0.1273
7	C	-1.1623	-0.3656	0.2426
8	C	1.2008	1.0851	-0.0337
9	H	2.2084	-0.8116	0.1324
10	C	-1.2054	1.0170	0.0814
11	H	-2.0844	-0.9329	0.3375
12	C	-0.0263	1.7659	-0.0556
13	H	2.1252	1.6457	-0.1488
14	H	-2.1667	1.5245	0.0564
15	C	-0.0759	3.2675	-0.1995
16	H	-0.9944	3.5922	-0.6993
17	H	-0.0507	3.7544	0.7845
18	H	0.7788	3.6442	-0.7709

SP_ar3m2cnoac
Energy (POTENTIAL) = -1706.1336105 Eh

	Atom	X	Y	Z
1	C	-1.0507	0.0018	-0.4277
2	C	-0.2634	1.3283	-0.3350
3	C	-0.1546	2.0284	0.8710
4	C	0.6096	3.1910	0.9847
5	C	1.3206	3.7023	-0.1152
6	C	1.2335	2.9837	-1.3253
7	C	0.4659	1.8308	-1.4259
8	H	0.4689	1.3065	-2.3762
9	H	1.7980	3.3128	-2.1917
10	H	0.6279	3.7012	1.9405
11	H	-0.6820	1.6727	1.7493
12	C	-2.1599	-0.0299	0.6494
13	C	-2.2606	-1.0406	1.6082
14	C	-3.3009	-1.0441	2.5426
15	C	-4.2746	-0.0417	2.5536
16	C	-4.1658	0.9810	1.5982
17	C	-3.1270	0.9917	0.6734
18	H	-3.0663	1.8057	-0.0420
19	H	-4.9022	1.7813	1.5825
20	H	-3.3527	-1.8484	3.2727
21	H	-1.5313	-1.8414	1.6299
22	C	-1.8055	-0.0125	-1.8598
23	H	-1.9863	1.0300	-2.1312
24	C	-1.0154	-0.5974	-2.9449
25	N	-0.4061	-1.0378	-3.8295
26	C	-3.1871	-0.6949	-1.8392

27	O	-3.3928	-1.8755	-1.6561
28	O	-4.1424	0.2136	-2.0579
29	C	-5.5024	-0.2632	-1.9381
30	H	-5.6780	-1.0896	-2.6298
31	H	-6.1299	0.5916	-2.1859
32	H	-5.6830	-0.5909	-0.9112
33	C	-0.0792	-1.1898	-0.2886
34	C	1.1944	-1.0322	0.2794
35	C	2.0593	-2.1078	0.4498
36	C	1.6918	-3.4128	0.0677
37	C	0.4113	-3.5763	-0.4930
38	C	-0.4446	-2.4896	-0.6654
39	H	-1.4286	-2.6723	-1.0814
40	H	0.0576	-4.5576	-0.7870
41	H	3.0463	-1.9209	0.8604
42	H	1.5344	-0.0501	0.5859
43	N	2.1311	4.8522	-0.0359
44	C	1.9295	5.8798	-1.0706
45	C	2.3249	5.4619	1.2801
46	C	3.0872	6.8722	-1.0550
47	H	0.9844	6.4216	-0.8921
48	H	1.8696	5.4172	-2.0572
49	C	3.4685	6.4731	1.2166
50	H	1.4086	5.9678	1.6328
51	H	2.5864	4.6857	2.0057
52	H	2.8991	7.6846	-1.7630
53	H	4.0188	6.3580	-1.3425
54	O	3.2347	7.4722	0.2291
55	H	3.5644	6.9926	2.1743
56	H	4.4122	5.9449	1.0037
57	N	2.6018	-4.4786	0.2149
58	C	3.2558	-4.6224	1.5255
59	C	2.2238	-5.7825	-0.3307
60	C	4.4411	-5.5761	1.4183
61	H	2.5399	-5.0143	2.2689
62	H	3.6109	-3.6535	1.8805
63	C	3.4445	-6.7001	-0.3717
64	H	1.4282	-6.2589	0.2694
65	H	1.8506	-5.6554	-1.3513
66	H	4.8761	-5.7518	2.4065
67	H	5.2109	-5.1360	0.7636
68	O	4.0381	-6.8465	0.9147
69	H	3.1510	-7.7015	-0.6996
70	H	4.1822	-6.2959	-1.0838
71	C	-5.3947	-0.0369	3.5662
72	H	-5.4678	-0.9957	4.0889
73	H	-5.2393	0.7433	4.3226
74	H	-6.3606	0.1669	3.0896

SP_obn_an_m2b_MeButa
Energy (POTENTIAL) = -617.2852747 Eh

	Atom	X	Y	Z
1	O	0.0808	-1.4553	-1.0784
2	C	1.1150	-0.7176	-0.5558
3	H	1.5066	-1.1163	0.4126
4	H	2.0133	-0.7199	-1.2205
5	C	0.7829	0.7562	-0.3005
6	C	1.7376	1.6328	0.2354
7	C	-0.4787	1.2707	-0.6063
8	C	1.4396	2.9760	0.4592
9	H	2.7309	1.2555	0.4787
10	C	-0.7794	2.6175	-0.3805
11	H	-1.2210	0.5901	-1.0095
12	C	0.1704	3.4944	0.1559
13	H	2.1999	3.6359	0.8750
14	H	-1.7714	2.9940	-0.6264
15	C	-0.1624	4.9437	0.4307
16	H	-1.0093	5.2828	-0.1761
17	H	-0.4337	5.1015	1.4839
18	H	0.6897	5.6003	0.2182
19	O	-2.0446	-1.6957	0.3971
20	H	-1.1850	-1.5264	-0.1258
21	C	-2.6865	-2.7510	-0.2843
22	H	-1.9936	-3.5735	-0.5191
23	H	-3.4915	-3.1508	0.3494
24	H	-3.1516	-2.4289	-1.2357
25	O	0.5636	-3.9489	-0.5075
26	H	0.4010	-2.9893	-0.8107
27	C	0.5496	-3.9049	0.9027
28	H	-0.2808	-3.2968	1.2908
29	H	0.4376	-4.9270	1.2933
30	H	1.4861	-3.4951	1.3284

ar3m2obn_meoh
Energy (POTENTIAL) = -1846.90233764 Eh

	Atom	X	Y	Z

1	C	-0.0745	0.1728	-0.7600
2	C	-1.3072	-0.6175	-0.3106
3	C	-2.5502	-0.3408	-0.9091
4	C	-1.3029	-1.4934	0.7768
5	C	-3.7202	-0.9337	-0.4593
6	H	-2.6062	0.3793	-1.7189
7	C	-2.4721	-2.1101	1.2281
8	H	-0.3736	-1.7170	1.2891
9	C	-3.7114	-1.8468	0.6186
10	H	-4.6598	-0.6628	-0.9300
11	H	-2.3994	-2.8055	2.0559
12	C	1.2685	-0.3832	-0.2550
13	C	2.2624	0.4686	0.2451
14	C	1.5923	-1.7366	-0.3972
15	C	3.5091	-0.0170	0.6239
16	H	2.0657	1.5319	0.3205
17	C	2.8358	-2.2366	-0.0119
18	H	0.8586	-2.4251	-0.8060
19	C	3.8250	-1.3856	0.5160
20	H	4.2580	0.6825	0.9825
21	H	3.0194	-3.2996	-0.1161
22	C	0.0450	0.2723	-2.2904
23	C	0.6434	1.3940	-2.8793
24	C	-0.3377	-0.7861	-3.1218
25	C	0.8269	1.4621	-4.2596
26	H	0.9664	2.2179	-2.2527
27	C	-0.1455	-0.7159	-4.5024
28	H	-0.7965	-1.6715	-2.6931
29	C	0.4329	0.4109	-5.0993
30	H	1.2893	2.3472	-4.6913
31	H	-0.4555	-1.5521	-5.1251
32	N	5.1077	-1.8416	0.8901
33	C	5.5688	-1.4721	2.2386
34	C	5.4553	-3.2284	0.5799
35	C	7.0648	-1.7359	2.3698
36	H	5.0284	-2.0582	3.0025
37	H	5.3744	-0.4148	2.4277
38	C	6.9573	-3.4360	0.7675
39	H	4.9065	-3.9403	1.2222
40	H	5.1992	-3.4428	-0.4622
41	H	7.3956	-1.5437	3.3945
42	H	7.6184	-1.0715	1.6864
43	O	7.3746	-3.0979	2.0868
44	H	7.2143	-4.4883	0.6157
45	H	7.5055	-2.8269	0.0305
46	N	-4.9190	-2.4104	1.0727
47	C	-5.8035	-3.0098	0.0603
48	C	-4.8818	-3.2231	2.2888
49	C	-7.1904	-3.2453	0.6492
50	H	-5.3885	-3.9715	-0.2881
51	H	-5.8916	-2.3501	-0.8045
52	C	-6.3025	-3.4560	2.8001
53	H	-4.3923	-4.1970	2.1109
54	H	-4.3174	-2.6943	3.0626
55	H	-7.8238	-3.7689	-0.0726
56	H	-7.6564	-2.2772	0.8955
57	O	-7.1254	-4.0670	1.8115
58	H	-6.2861	-4.1325	3.6594
59	H	-6.7404	-2.4946	3.1142
60	O	-0.2284	1.5526	-0.3131
61	C	-0.5657	1.7486	1.0789
62	H	-1.6343	1.5475	1.2173
63	H	-0.0050	1.0438	1.7012
64	C	-0.2195	3.1705	1.4297
65	C	0.9364	3.4712	2.1581
66	C	-1.0343	4.2263	0.9976
67	C	1.2718	4.7953	2.4483
68	H	1.5807	2.6648	2.5005
69	C	-0.6932	5.5468	1.2846
70	H	-1.9258	4.0066	0.4164
71	C	0.4634	5.8549	2.0177
72	H	2.1749	5.0071	3.0162
73	H	-1.3349	6.3533	0.9365
74	C	0.8073	7.2871	2.3537
75	H	0.5394	7.9649	1.5358
76	H	0.2611	7.6241	3.2447
77	H	1.8758	7.4058	2.5609
78	C	0.6065	0.5048	-6.5968
79	H	-0.2201	1.0637	-7.0553
80	H	1.5332	1.0264	-6.8602
81	H	0.6281	-0.4865	-7.0614
82	C	-1.7971	3.9057	-2.5304
83	H	-2.6531	4.4214	-2.9772
84	H	-1.2230	4.6381	-1.9425
85	H	-1.1547	3.5307	-3.3399
86	O	-2.3060	2.8536	-1.7204

87	H	-1.5399	2.4071	-1.3031

ar3no2_cat
Energy (POTENTIAL) = -1181.23526798 Eh

	Atom	X	Y	Z
1	C	-0.0031	0.0008	0.5316
2	C	0.0177	-1.2540	-0.2144
3	C	0.7636	-1.3574	-1.4115
4	C	-0.7211	-2.3709	0.2405
5	C	0.7948	-2.5502	-2.1196
6	H	1.3501	-0.5149	-1.7588
7	C	-0.7153	-3.5583	-0.4784
8	H	-1.3337	-2.2873	1.1304
9	C	0.0494	-3.6275	-1.6415
10	H	1.3804	-2.6515	-3.0238
11	H	-1.2929	-4.4139	-0.1537
12	C	-0.0058	0.0049	1.9551
13	C	-0.5750	1.0920	2.6858
14	C	0.5590	-1.0789	2.6959
15	C	-0.5872	1.0805	4.0653
16	H	-1.0621	1.8994	2.1517
17	C	0.5633	-1.0588	4.0742
18	H	1.0446	-1.8920	2.1691
19	C	-0.0119	0.0149	4.7880
20	H	-1.0566	1.8973	4.6041
21	H	1.0245	-1.8750	4.6216
22	C	-0.0203	1.2520	-0.2206
23	C	0.7165	2.3711	0.2320
24	C	-0.7616	1.3500	-1.4211
25	C	0.7131	3.5554	-0.4922
26	H	1.3259	2.2916	1.1245
27	C	-0.7903	2.5396	-2.1346
28	H	-1.3468	0.5059	-1.7668
29	C	-0.0471	3.6192	-1.6584
30	H	-1.3726	2.6368	-3.0415
31	H	1.2893	4.4126	-0.1690
32	C	0.0162	0.0285	6.2867
33	H	1.0019	0.3709	6.6310
34	H	-0.7349	0.7055	6.7012
35	H	-0.1357	-0.9751	6.6964
36	N	-0.0618	4.8818	-2.4257
37	O	-0.7384	4.9195	-3.4519
38	O	0.6027	5.8218	-1.9933
39	N	0.0667	-4.8934	-2.4032
40	O	0.7471	-4.9358	-3.4267
41	O	-0.5996	-5.8314	-1.9692

SP_cnoac_an_MeProp
Energy (POTENTIAL) = -360.2631038 Eh

	Atom	X	Y	Z
1	C	-0.8577	0.7628	-0.0073
2	H	-0.7552	1.8418	0.0154
3	C	-2.1361	0.2080	-0.0646
4	N	-3.2244	-0.2402	-0.1131
5	C	0.2937	-0.0536	0.0238
6	O	0.3804	-1.2904	0.0050
7	O	1.4587	0.7238	0.0842
8	C	2.6706	-0.0192	0.1084
9	H	2.7168	-0.6960	0.9699
10	H	3.4758	0.7178	0.1756
11	H	2.7984	-0.6214	-0.7995

ar3m2cnoac_MeButa
Energy (POTENTIAL) = -1705.67637101 Eh

	Atom	X	Y	Z
1	C	-1.0510	0.0049	-0.4260
2	C	-0.2607	1.3299	-0.3329
3	C	-0.1649	2.0424	0.8667
4	C	0.6006	3.2047	0.9768
5	C	1.3258	3.7019	-0.1195
6	C	1.2531	2.9699	-1.3223
7	C	0.4843	1.8180	-1.4200
8	H	0.4999	1.2800	-2.3624
9	H	1.8322	3.2874	-2.1835
10	H	0.6090	3.7254	1.9271
11	H	-0.7032	1.6971	1.7424
12	C	-2.1582	-0.0290	0.6531
13	C	-2.2605	-1.0471	1.6039
14	C	-3.2987	-1.0547	2.5399
15	C	-4.2692	-0.0494	2.5608
16	C	-4.1602	0.9795	1.6125
17	C	-3.1233	0.9939	0.6854
18	H	-3.0618	1.8126	-0.0247
19	H	-4.8946	1.7818	1.6044
20	H	-3.3523	-1.8650	3.2632
21	H	-1.5340	-1.8506	1.6158

22	C	-1.8102	-0.0023	-1.8549
23	H	-1.9966	1.0421	-2.1152
24	C	-1.0244	-0.5730	-2.9512
25	N	-0.4213	-0.9988	-3.8471
26	C	-3.1887	-0.6905	-1.8327
27	O	-3.3912	-1.8713	-1.6536
28	O	-4.1496	0.2167	-2.0450
29	C	-5.5048	-0.2691	-1.9201
30	H	-5.6795	-1.0942	-2.6139
31	H	-6.1396	0.5826	-2.1607
32	H	-5.6778	-0.6037	-0.8940
33	C	-0.0807	-1.1876	-0.2904
34	C	1.1816	-1.0346	0.3038
35	C	2.0451	-2.1104	0.4760
36	C	1.6870	-3.4102	0.0695
37	C	0.4184	-3.5691	-0.5169
38	C	-0.4370	-2.4824	-0.6913
39	H	-1.4130	-2.6630	-1.1262
40	H	0.0736	-4.5473	-0.8315
41	H	3.0252	-1.9285	0.9054
42	H	1.5135	-0.0553	0.6281
43	N	2.1373	4.8521	-0.0442
44	C	1.9380	5.8736	-1.0847
45	C	2.3270	5.4688	1.2685
46	C	3.0959	6.8663	-1.0714
47	H	0.9924	6.4169	-0.9120
48	H	1.8800	5.4043	-2.0685
49	C	3.4714	6.4793	1.2023
50	H	1.4103	5.9782	1.6158
51	H	2.5850	4.6955	1.9987
52	H	2.9098	7.6757	-1.7834
53	H	4.0279	6.3503	-1.3546
54	O	3.2401	7.4723	0.2093
55	H	3.5654	7.0048	2.1570
56	H	4.4152	5.9490	0.9950
57	N	2.5976	-4.4760	0.2205
58	C	3.2092	-4.6479	1.5473
59	C	2.2442	-5.7674	-0.3676
60	C	4.4043	-5.5911	1.4540
61	H	2.4733	-5.0638	2.2578
62	H	3.5437	-3.6849	1.9372
63	C	3.4723	-6.6759	-0.3933
64	H	1.4346	-6.2644	0.1965
65	H	1.9001	-5.6157	-1.3952
66	H	4.8095	-5.7901	2.4506
67	H	5.1912	-5.1291	0.8354
68	O	4.0265	-6.8500	0.9065
69	H	3.1973	-7.6713	-0.7541
70	H	4.2283	-6.2488	-1.0723
71	C	-5.3837	-0.0476	3.5797
72	H	-5.4937	-1.0269	4.0562
73	H	-5.1920	0.6868	4.3731
74	H	-6.3429	0.2184	3.1212

SP_ar3no2obn_meoh_PMMA
Energy (POTENTIAL) = -1683.1411791 Eh

	Atom	X	Y	Z
1	C	-0.3248	-0.3949	-0.2731
2	C	-1.7002	-0.2596	0.4011
3	C	-2.6523	0.5771	-0.2079
4	C	-1.9929	-0.8209	1.6503
5	C	-3.8777	0.8226	0.3988
6	H	-2.4220	1.0605	-1.1508
7	C	-3.2194	-0.5895	2.2701
8	H	-1.2648	-1.4422	2.1579
9	C	-4.1484	0.2254	1.6304
10	H	-4.6143	1.4667	-0.0646
11	H	-3.4529	-1.0252	3.2333
12	C	0.5695	-1.5111	0.2977
13	C	1.9186	-1.2637	0.5828
14	C	0.0770	-2.8200	0.4284
15	C	2.7536	-2.2861	1.0249
16	H	2.3222	-0.2682	0.4463
17	C	0.8959	-3.8523	0.8681
18	H	-0.9562	-3.0363	0.1787
19	C	2.2288	-3.5683	1.1684
20	H	3.7957	-2.0999	1.2527
21	H	0.5202	-4.8619	0.9760
22	C	-0.4313	-0.6767	-1.7816
23	C	0.5750	-0.2239	-2.6438
24	C	-1.4617	-1.4616	-2.3101
25	C	0.5305	-0.5256	-4.0034
26	H	1.3932	0.3687	-2.2497
27	C	-1.4957	-1.7654	-3.6708
28	H	-2.2516	-1.8336	-1.6647
29	C	-0.5063	-1.2979	-4.5445

30	H	1.3199	-0.1575	-4.6547
31	H	-2.3099	-2.3728	-4.0586
32	O	0.3498	0.8785	-0.1695
33	C	0.3960	1.5067	1.1387
34	H	-0.5697	1.9874	1.3304
35	H	0.5597	0.7479	1.9111
36	C	1.5187	2.5065	1.1149
37	C	2.7267	2.2484	1.7701
38	C	1.3814	3.7050	0.3998
39	C	3.7770	3.1667	1.7129
40	H	2.8489	1.3257	2.3329
41	C	2.4342	4.6145	0.3406
42	H	0.4506	3.9107	-0.1221
43	C	3.6491	4.3635	0.9979
44	H	4.7081	2.9491	2.2308
45	H	2.3130	5.5377	-0.2218
46	C	4.7724	5.3721	0.9527
47	H	4.8674	5.8194	-0.0431
48	H	4.5921	6.1929	1.6592
49	H	5.7327	4.9170	1.2156
50	C	-0.5664	-1.5929	-6.0239
51	H	-1.0749	-0.7834	-6.5640
52	H	0.4363	-1.6897	-6.4537
53	H	-1.1168	-2.5173	-6.2270
54	C	-0.1580	3.2259	-2.9438
55	H	-0.7247	4.0125	-3.4509
56	H	0.8216	3.6373	-2.6553
57	H	0.0056	2.4010	-3.6518
58	O	-0.9168	2.8170	-1.8125
59	H	-0.4068	2.1235	-1.3494
60	N	3.1007	-4.6498	1.6385
61	O	4.2757	-4.3773	1.8936
62	O	2.6138	-5.7763	1.7562
63	N	-5.4447	0.4734	2.2763
64	O	-5.6601	-0.0619	3.3653
65	O	-6.2491	1.2035	1.6952

ar3m2obn_meoh_B
Energy (POTENTIAL) = -1846.90048388 Eh

	Atom	X	Y	Z
1	C	-0.5788	-0.6515	0.0444
2	C	-1.7880	0.2974	-0.0131
3	C	-2.2451	0.9533	1.1406
4	C	-3.4001	1.7236	1.1340
5	C	-4.1834	1.8658	-0.0341
6	C	-3.7356	1.1849	-1.1835
7	C	-2.5677	0.4218	-1.1650
8	H	-2.2788	-0.1059	-2.0676
9	H	-4.3029	1.2220	-2.1056
10	N	-5.3138	2.7026	-0.0309
11	C	-6.0749	2.9052	-1.2682
12	C	-7.1405	1.8211	-1.4553
13	H	-7.7639	2.0294	-2.3303
14	H	-6.6645	0.8364	-1.5874
15	O	-8.0189	1.7911	-0.3284
16	C	-7.3037	1.5661	0.8866
17	C	-6.2420	2.6472	1.1114
18	H	-5.7070	2.4598	2.0428
19	H	-6.7325	3.6243	1.1993
20	H	-8.0464	1.5752	1.6904
21	H	-6.8188	0.5758	0.8643
22	H	-5.4024	2.9634	-2.1267
23	H	-6.5807	3.8752	-1.1840
24	H	-3.6827	2.2402	2.0451
25	H	-1.6826	0.8663	2.0655
26	C	0.6372	-0.0925	0.7827
27	C	1.6781	-0.9582	1.1434
28	C	2.8748	-0.4953	1.6722
29	C	3.1048	0.8858	1.8712
30	C	2.0555	1.7564	1.5047
31	C	0.8640	1.2750	0.9698
32	H	0.1032	1.9945	0.6866
33	H	2.1563	2.8272	1.6345
34	N	4.2908	1.3700	2.4488
35	C	5.4373	0.4613	2.5968
36	C	6.2024	0.3443	1.2764
37	H	7.0965	-0.2762	1.3887
38	H	5.5563	-0.1010	0.5045
39	O	6.6584	1.6335	0.8513
40	C	5.5802	2.5676	0.7259
41	C	4.8005	2.6900	2.0377
42	H	3.9949	3.4173	1.9379
43	H	5.4668	3.0499	2.8299
44	H	6.0337	3.5233	0.4459
45	H	4.8982	2.2493	-0.0756
46	H	5.1216	-0.5139	2.9702

47	H	6.1005	0.8944	3.3543
48	H	3.6417	-1.2247	1.8987
49	H	1.5646	-2.0258	0.9818
50	C	-1.1161	-1.9589	0.6558
51	C	-1.0435	-2.2082	2.0314
52	C	-1.6235	-3.3509	2.5844
53	C	-2.3002	-4.2813	1.7867
54	C	-2.3810	-4.0213	0.4117
55	C	-1.8111	-2.8774	-0.1437
56	H	-1.9085	-2.6959	-1.2080
57	H	-2.9012	-4.7237	-0.2360
58	C	-2.9487	-5.5061	2.3878
59	H	-2.4703	-5.7911	3.3306
60	H	-4.0107	-5.3255	2.6014
61	H	-2.8977	-6.3617	1.7055
62	H	-1.5427	-3.5217	3.6556
63	H	-0.5227	-1.5111	2.6797
64	O	-0.1578	-1.0159	-1.3076
65	C	0.4086	0.0214	-2.1256
66	H	-0.0114	0.9922	-1.8440
67	H	0.0471	-0.2031	-3.1367
68	C	1.9226	0.1015	-2.1364
69	C	2.7391	-1.0318	-2.0724
70	C	4.1274	-0.9127	-2.1705
71	C	4.7438	0.3324	-2.3492
72	C	3.9171	1.4631	-2.4244
73	C	2.5326	1.3505	-2.3083
74	H	1.9179	2.2470	-2.3529
75	H	4.3650	2.4446	-2.5646
76	C	6.2462	0.4697	-2.4344
77	H	6.5402	1.0991	-3.2828
78	H	6.6495	0.9369	-1.5274
79	H	6.7307	-0.5052	-2.5517
80	H	4.7434	-1.8076	-2.1104
81	H	2.2951	-2.0125	-1.9392
82	C	0.2726	-3.3709	-3.4837
83	H	0.4865	-4.2884	-4.0407
84	H	-0.7934	-3.1294	-3.6131
85	H	0.8667	-2.5555	-3.9254
86	O	0.6088	-3.6038	-2.1224
87	H	0.3581	-2.7891	-1.6428

SP_obn_an_m2c_MeButa
Energy (POTENTIAL) = -617.2837074 Eh

	Atom	X	Y	Z
1	O	0.8106	-1.3018	-1.2765
2	C	1.4961	-0.3587	-0.4904
3	H	1.7083	-0.7576	0.5200
4	H	2.4763	-0.1707	-0.9543
5	C	0.7719	0.9730	-0.3260
6	C	1.4752	2.1810	-0.2940
7	C	-0.6224	1.0106	-0.1697
8	C	0.8121	3.3970	-0.1037
9	H	2.5563	2.1772	-0.4265
10	C	-1.2793	2.2259	0.0179
11	H	-1.1768	0.0735	-0.1929
12	C	-0.5774	3.4407	0.0575
13	H	1.3821	4.3245	-0.0900
14	H	-2.3620	2.2354	0.1355
15	C	-1.3031	4.7474	0.2856
16	H	-2.2247	4.8032	-0.3059
17	H	-1.5920	4.8654	1.3389
18	H	-0.6784	5.6068	0.0192
19	O	-1.1658	-2.1424	0.1427
20	H	0.0170	-1.6554	-0.7161
21	C	-0.8117	-2.1054	1.4824
22	H	-0.5840	-1.0761	1.8376
23	H	-1.6166	-2.4816	2.1531
24	H	0.0877	-2.7132	1.7323
25	O	-0.8146	-4.6416	-0.4740
26	H	-1.0461	-3.6748	-0.2494
27	C	0.5928	-4.7053	-0.4419
28	H	0.9929	-4.8869	0.5757
29	H	0.9383	-5.5378	-1.0743
30	H	1.0593	-3.7796	-0.8108

SP_ar3m2cnoac_MeProp
Energy (POTENTIAL) = -1706.1290541 Eh

	Atom	X	Y	Z
1	C	-1.0508	0.0044	-0.4262
2	C	-0.2607	1.3296	-0.3331
3	C	-0.1635	2.0407	0.8671
4	C	0.6017	3.2031	0.9776
5	C	1.3252	3.7020	-0.1192
6	C	1.2510	2.9714	-1.3227
7	C	0.4824	1.8192	-1.4207

8	H	0.4967	1.2827	-2.3639
9	H	1.8285	3.2901	-2.1846
10	H	0.6112	3.7227	1.9285
11	H	-0.7006	1.6943	1.7432
12	C	-2.1582	-0.0291	0.6527
13	C	-2.2600	-1.0460	1.6048
14	C	-3.2983	-1.0529	2.5408
15	C	-4.2692	-0.0480	2.5604
16	C	-4.1606	0.9798	1.6110
17	C	-3.1236	0.9936	0.6839
18	H	-3.0624	1.8116	-0.0271
19	H	-4.8953	1.7818	1.6018
20	H	-3.3515	-1.8623	3.2652
21	H	-1.5330	-1.8491	1.6180
22	C	-1.8094	-0.0036	-1.8555
23	H	-1.9950	1.0406	-2.1171
24	C	-1.0232	-0.5761	-2.9504
25	N	-0.4195	-1.0036	-3.8451
26	C	-3.1884	-0.6909	-1.8335
27	O	-3.3915	-1.8717	-1.6539
28	O	-4.1485	0.2166	-2.0467
29	C	-5.5043	-0.2680	-1.9225
30	H	-5.6790	-1.0936	-2.6157
31	H	-6.1382	0.5839	-2.1645
32	H	-5.6785	-0.6013	-0.8962
33	C	-0.0804	-1.1881	-0.2902
34	C	1.1834	-1.0344	0.3004
35	C	2.0470	-2.1104	0.4723
36	C	1.6874	-3.4108	0.0692
37	C	0.4172	-3.5703	-0.5136
38	C	-0.4382	-2.4835	-0.6877
39	H	-1.4154	-2.6644	-1.1198
40	H	0.0711	-4.5489	-0.8253
41	H	3.0283	-1.9279	0.8990
42	H	1.5167	-0.0547	0.6221
43	N	2.1364	4.8523	-0.0434
44	C	1.9368	5.8743	-1.0834
45	C	2.3264	5.4683	1.2695
46	C	3.0945	6.8672	-1.0698
47	H	0.9912	6.4172	-0.9103
48	H	1.8789	5.4057	-2.0675
49	C	3.4704	6.4791	1.2037
50	H	1.4096	5.9771	1.6174
51	H	2.5848	4.6948	1.9993
52	H	2.9081	7.6768	-1.7814
53	H	4.0266	6.3516	-1.3533
54	O	3.2388	7.4727	0.2113
55	H	3.5644	7.0041	2.1588
56	H	4.4144	5.9492	0.9959
57	N	2.5979	-4.4767	0.2198
58	C	3.2143	-4.6454	1.5448
59	C	2.2414	-5.7696	-0.3634
60	C	4.4082	-5.5899	1.4500
61	H	2.4805	-5.0585	2.2592
62	H	3.5512	-3.6817	1.9308
63	C	3.4686	-6.6793	-0.3909
64	H	1.4333	-6.2641	0.2049
65	H	1.8939	-5.6207	-1.3902
66	H	4.8168	-5.7863	2.4457
67	H	5.1933	-5.1305	0.8272
68	O	4.0272	-6.8502	0.9075
69	H	3.1913	-7.6754	-0.7479
70	H	4.2225	-6.2548	-1.0738
71	C	-5.3841	-0.0456	3.5790
72	H	-5.4912	-1.0234	4.0594
73	H	-5.1950	0.6926	4.3693
74	H	-6.3440	0.2154	3.1189

SP_ar3m2obn_MeButa
Energy (POTENTIAL) = -1731.6041451 Eh

	Atom	X	Y	Z
1	C	-0.1385	0.2977	-0.8996
2	C	-1.4380	-0.3578	-0.4145
3	C	-2.6220	-0.1377	-1.1390
4	C	-1.5530	-1.0276	0.8053
5	C	-3.8503	-0.5927	-0.6838
6	H	-2.5782	0.4165	-2.0716
7	C	-2.7814	-1.5056	1.2684
8	H	-0.6716	-1.1958	1.4150
9	C	-3.9610	-1.3014	0.5324
10	H	-4.7419	-0.3727	-1.2626
11	H	-2.8023	-2.0478	2.2066
12	C	1.1483	-0.2834	-0.2798
13	C	2.2100	0.5651	0.0645
14	C	1.3613	-1.6600	-0.1569
15	C	3.4171	0.0628	0.5355

16	H	2.0912	1.6363	-0.0557
17	C	2.5651	-2.1755	0.3250
18	H	0.5718	-2.3548	-0.4269
19	C	3.6238	-1.3227	0.6854
20	H	4.2226	0.7548	0.7618
21	H	2.6625	-3.2501	0.4275
22	C	0.0227	0.1883	-2.4269
23	C	0.4960	1.2658	-3.1818
24	C	-0.2287	-1.0229	-3.0857
25	C	0.7035	1.1346	-4.5567
26	H	0.7015	2.2098	-2.6913
27	C	-0.0181	-1.1489	-4.4568
28	H	-0.6008	-1.8740	-2.5230
29	C	0.4502	-0.0706	-5.2211
30	H	1.0712	1.9889	-5.1215
31	H	-0.2230	-2.1007	-4.9425
32	N	4.8727	-1.8011	1.1406
33	C	5.3790	-1.2311	2.3987
34	C	5.1117	-3.2426	1.0865
35	C	6.8541	-1.5774	2.5711
36	H	4.8101	-1.6283	3.2580
37	H	5.2638	-0.1455	2.3940
38	C	6.5974	-3.5255	1.3046
39	H	4.5232	-3.7840	1.8491
40	H	4.8231	-3.6216	0.1011
41	H	7.2160	-1.2293	3.5431
42	H	7.4423	-1.0896	1.7764
43	O	7.0600	-2.9865	2.5384
44	H	6.7742	-4.6042	1.3471
45	H	7.1762	-3.1036	0.4668
46	N	-5.2269	-1.7294	0.9819
47	C	-6.0535	-2.4845	0.0271
48	C	-5.3182	-2.3031	2.3240
49	C	-7.4916	-2.5631	0.5288
50	H	-5.6568	-3.5072	-0.0983
51	H	-6.0417	-1.9985	-0.9500
52	C	-6.7833	-2.3948	2.7486
53	H	-4.8607	-3.3077	2.3690
54	H	-4.7902	-1.6564	3.0315
55	H	-8.0908	-3.1940	-0.1342
56	H	-7.9304	-1.5520	0.5509
57	O	-7.5545	-3.1531	1.8234
58	H	-6.8642	-2.9007	3.7150
59	H	-7.1998	-1.3787	2.8443
60	O	-0.2136	1.7143	-0.6316
61	C	-0.5327	2.0929	0.7141
62	H	-1.6123	1.9796	0.8794
63	H	-0.0166	1.4456	1.4337
64	C	-0.1013	3.5247	0.8891
65	C	1.0299	3.8536	1.6420
66	C	-0.8072	4.5571	0.2574
67	C	1.4413	5.1823	1.7689
68	H	1.5972	3.0638	2.1291
69	C	-0.3927	5.8812	0.3807
70	H	-1.6831	4.3153	-0.3398
71	C	0.7372	6.2177	1.1426
72	H	2.3232	5.4168	2.3609
73	H	-0.9527	6.6688	-0.1189
74	C	1.1610	7.6595	1.2995
75	H	1.0305	8.2186	0.3663
76	H	0.5599	8.1664	2.0661
77	H	2.2107	7.7391	1.6007
78	C	0.6464	-0.2031	-6.7133
79	H	-0.3021	-0.0727	-7.2514
80	H	1.3442	0.5498	-7.0945
81	H	1.0341	-1.1928	-6.9800

SP_obn_an_m2
Energy (POTENTIAL) = -617.300304 Eh

	Atom	X	Y	Z
1	O	0.4556	-1.6727	-1.2485
2	C	1.1971	-0.9304	-0.3554
3	H	1.1822	-1.3442	0.6826
4	H	2.2813	-0.9189	-0.6177
5	C	0.7680	0.5336	-0.2316
6	C	1.5726	1.4697	0.4349
7	C	-0.4438	0.9773	-0.7674
8	C	1.1777	2.8004	0.5636
9	H	2.5269	1.1510	0.8534
10	C	-0.8422	2.3118	-0.6359
11	H	-1.0761	0.2541	-1.2721
12	C	-0.0420	3.2474	0.0299
13	H	1.8249	3.5075	1.0801
14	H	-1.7922	2.6319	-1.0612
15	C	-0.4765	4.6867	0.1916
16	H	-1.2961	4.9375	-0.4902

17	H	-0.8265	4.8872	1.2134
18	H	0.3501	5.3804	-0.0035
19	O	-1.9840	-2.0713	-0.4411
20	H	-1.0239	-1.9146	-0.7589
21	C	-2.0238	-1.7287	0.9259
22	H	-1.6801	-0.6978	1.1145
23	H	-3.0614	-1.8031	1.2800
24	H	-1.4126	-2.4012	1.5530
25	O	1.2273	-4.0985	-0.7917
26	H	0.9537	-3.1445	-1.0352
27	C	0.3787	-4.4700	0.2714
28	H	-0.6765	-4.2266	0.0718
29	H	0.4565	-5.5547	0.4335
30	H	0.6447	-3.9784	1.2271

obn_an_m2_MeButa
Energy (POTENTIAL) = -617.070353670 Eh

	Atom	X	Y	Z
1	O	0.4605	-1.6841	-1.2594
2	C	1.2317	-0.9335	-0.4042
3	H	1.2784	-1.3465	0.6345
4	H	2.3021	-0.9018	-0.7195
5	C	0.7879	0.5244	-0.2555
6	C	1.5827	1.4590	0.4242
7	C	-0.4273	0.9632	-0.7868
8	C	1.1758	2.7840	0.5700
9	H	2.5395	1.1425	0.8396
10	C	-0.8381	2.2918	-0.6376
11	H	-1.0505	0.2388	-1.3010
12	C	-0.0476	3.2260	0.0411
13	H	1.8155	3.4906	1.0971
14	H	-1.7911	2.6085	-1.0590
15	C	-0.4970	4.6584	0.2227
16	H	-1.3145	4.9120	-0.4610
17	H	-0.8566	4.8410	1.2448
18	H	0.3235	5.3639	0.0430
19	O	-1.9834	-2.0534	-0.4440
20	H	-1.0176	-1.9096	-0.7515
21	C	-2.0371	-1.6999	0.9174
22	H	-1.6939	-0.6679	1.1037
23	H	-3.0789	-1.7698	1.2615
24	H	-1.4345	-2.3683	1.5583
25	O	1.2367	-4.1036	-0.7494
26	H	0.9656	-3.1602	-1.0296
27	C	0.3609	-4.4407	0.3009
28	H	-0.6881	-4.1981	0.0700
29	H	0.4293	-5.5213	0.4955
30	H	0.6059	-3.9244	1.2502

SP_ar3no2cnoac_PMMA
Energy (POTENTIAL) = -1541.8777663 Eh

	Atom	X	Y	Z
1	C	-0.3324	0.0155	-0.3431
2	C	0.2547	1.4432	-0.2054
3	C	0.0564	2.1971	0.9613
4	C	0.6262	3.4587	1.1113
5	C	1.4060	3.9706	0.0784
6	C	1.6393	3.2450	-1.0880
7	C	1.0678	1.9839	-1.2166
8	H	1.2816	1.4173	-2.1154
9	H	2.2611	3.6606	-1.8708
10	H	0.4716	4.0393	2.0120
11	H	-0.5459	1.7987	1.7679
12	C	-1.5137	-0.1824	0.6335
13	C	-1.5256	-1.1719	1.6196
14	C	-2.6303	-1.3317	2.4599
15	C	-3.7570	-0.5126	2.3471
16	C	-3.7391	0.4876	1.3632
17	C	-2.6387	0.6552	0.5293
18	H	-2.6570	1.4479	-0.2121
19	H	-4.5964	1.1471	1.2521
20	H	-2.6107	-2.1151	3.2134
21	H	-0.6781	-1.8360	1.7390
22	C	-0.9365	-0.1102	-1.8339
23	H	-1.2648	0.8900	-2.1258
24	C	0.0295	-0.5459	-2.8454
25	N	0.7825	-0.8754	-3.6647
26	C	-2.1847	-1.0149	-1.9288
27	O	-2.2086	-2.2064	-1.7161
28	O	-3.2423	-0.2843	-2.2904
29	C	-4.5065	-0.9877	-2.3197
30	H	-4.4519	-1.8357	-3.0053
31	H	-5.2349	-0.2545	-2.6626
32	H	-4.7501	-1.3380	-1.3136
33	C	0.7884	-1.0156	-0.0796
34	C	1.9153	-0.6654	0.6805

35	C	2.9118	-1.5943	0.9667
36	C	2.7760	-2.8929	0.4851
37	C	1.6659	-3.2803	-0.2619
38	C	0.6774	-2.3410	-0.5343
39	H	-0.1977	-2.6532	-1.0904
40	H	1.5805	-4.2992	-0.6179
41	H	3.7809	-1.3200	1.5510
42	H	2.0308	0.3419	1.0598
43	C	-4.9439	-0.6721	3.2661
44	H	-4.9541	-1.6578	3.7414
45	H	-4.9246	0.0804	4.0651
46	H	-5.8876	-0.5431	2.7245
47	N	2.0053	5.3035	0.2260
48	O	2.6925	5.7334	-0.7017
49	O	1.7887	5.9212	1.2700
50	N	3.8235	-3.8792	0.7791
51	O	3.6770	-5.0240	0.3475
52	O	4.7940	-3.5114	1.4439

ar3m2_cat_MeProp
Energy (POTENTIAL) = -1345.48649531 Eh

	Atom	X	Y	Z
1	C	-0.1700	0.0030	1.1707
2	C	-0.1906	-1.2388	0.4563
3	C	0.3529	-1.3709	-0.8544
4	C	-0.7510	-2.4179	1.0251
5	C	0.3366	-2.5631	-1.5369
6	H	0.8578	-0.5224	-1.3035
7	C	-0.7943	-3.6105	0.3431
8	H	-1.2193	-2.3612	2.0017
9	C	-0.2443	-3.7377	-0.9686
10	H	0.8282	-2.6066	-2.5001
11	H	-1.3022	-4.4458	0.8068
12	C	-0.1226	-0.0150	2.6372
13	C	-0.8855	0.8960	3.3998
14	C	0.6825	-0.9468	3.3270
15	C	-0.8499	0.8641	4.7879
16	H	-1.5350	1.6018	2.8921
17	C	0.7281	-0.9555	4.7156
18	H	1.2951	-1.6421	2.7622
19	C	-0.0377	-0.0546	5.4724
20	H	-1.4657	1.5579	5.3541
21	H	1.3696	-1.6699	5.2245
22	C	-0.1971	1.2626	0.4868
23	C	0.3971	2.4303	1.0464
24	C	-0.8355	1.4262	-0.7750
25	C	0.3873	3.6399	0.3953
26	H	0.9255	2.3528	1.9903
27	C	-0.8729	2.6373	-1.4252
28	H	-1.3750	0.5892	-1.2055
29	C	-0.2450	3.7957	-0.8756
30	H	-1.4423	2.7078	-2.3426
31	C	-0.6896	-6.1867	-1.0207
32	C	-0.1457	-5.0364	-3.0983
33	C	0.1980	-7.3389	-1.5025
34	H	-1.7375	-6.3836	-1.2864
35	H	-0.6086	-6.1286	0.0641
36	C	0.7293	-6.2275	-3.4847
37	H	-1.1561	-5.1848	-3.5051
38	H	0.2599	-4.1254	-3.5328
39	H	-0.1986	-8.2866	-1.1286
40	H	1.2180	-7.2013	-1.1093
41	H	0.7216	-6.3576	-4.5700
42	H	1.7653	-6.0518	-3.1536
43	N	-0.2518	-4.9268	-1.6357
44	O	0.2248	-7.4307	-2.9195
45	N	-0.2410	4.9890	-1.5372
46	C	-0.7700	5.1419	-2.8988
47	C	-0.0669	6.2841	-0.8613
48	C	0.0721	6.1567	-3.6779
49	H	-1.8162	5.4761	-2.8553
50	H	-0.7276	4.1913	-3.4297
51	C	0.7541	7.2375	-1.7271
52	H	-1.0614	6.7195	-0.6879
53	H	0.4182	6.1603	0.1044
54	H	-0.3923	6.3475	-4.6491
55	H	1.0803	5.7434	-3.8420
56	O	0.1557	7.4047	-3.0057
57	H	0.7888	8.2250	-1.2596
58	H	1.7825	6.8559	-1.8306
59	H	0.9121	4.4688	0.8520
60	C	0.0291	-0.0535	6.9781
61	H	0.7834	0.6635	7.3284
62	H	-0.9282	0.2403	7.4205
63	H	0.3061	-1.0373	7.3692

ar3no2obn_meoh
Energy (POTENTIAL) = -1682.68695382 Eh

	Atom	X	Y	Z
1	C	-0.3288	-0.3989	-0.2724
2	C	-1.7039	-0.2619	0.4019
3	C	-2.6582	0.5690	-0.2117
4	C	-1.9929	-0.8137	1.6559
5	C	-3.8826	0.8180	0.3946
6	H	-2.4311	1.0425	-1.1604
7	C	-3.2181	-0.5786	2.2764
8	H	-1.2637	-1.4301	2.1676
9	C	-4.1495	0.2303	1.6321
10	H	-4.6208	1.4562	-0.0741
11	H	-3.4468	-1.0077	3.2436
12	C	0.5643	-1.5164	0.2970
13	C	1.9167	-1.2729	0.5703
14	C	0.0669	-2.8226	0.4365
15	C	2.7513	-2.2964	1.0095
16	H	2.3235	-0.2796	0.4288
17	C	0.8853	-3.8563	0.8734
18	H	-0.9693	-3.0369	0.1981
19	C	2.2222	-3.5764	1.1615
20	H	3.7952	-2.1108	1.2288
21	H	0.5042	-4.8629	0.9884
22	C	-0.4344	-0.6806	-1.7811
23	C	0.5713	-0.2263	-2.6433
24	C	-1.4634	-1.4682	-2.3093
25	C	0.5275	-0.5280	-4.0033
26	H	1.3888	0.3678	-2.2501
27	C	-1.4967	-1.7721	-3.6703
28	H	-2.2518	-1.8433	-1.6640
29	C	-0.5082	-1.3024	-4.5443
30	H	1.3161	-0.1581	-4.6543
31	H	-2.3092	-2.3820	-4.0575
32	O	0.3448	0.8755	-0.1673
33	C	0.3988	1.4956	1.1455
34	H	-0.5681	1.9684	1.3495
35	H	0.5749	0.7331	1.9109
36	C	1.5141	2.5034	1.1173
37	C	2.7340	2.2459	1.7509
38	C	1.3583	3.7098	0.4193
39	C	3.7771	3.1724	1.6885
40	H	2.8716	1.3172	2.3000
41	C	2.4038	4.6279	0.3542
42	H	0.4187	3.9154	-0.0864
43	C	3.6305	4.3772	0.9899
44	H	4.7176	2.9545	2.1888
45	H	2.2678	5.5564	-0.1957
46	C	4.7467	5.3934	0.9390
47	H	4.8230	5.8527	-0.0528
48	H	4.5716	6.2051	1.6573
49	H	5.7133	4.9413	1.1826
50	C	-0.5678	-1.5978	-6.0236
51	H	-1.0789	-0.7896	-6.5633
52	H	0.4351	-1.6909	-6.4537
53	H	-1.1161	-2.5236	-6.2260
54	C	-0.0861	3.2330	-2.9213
55	H	-0.6298	4.0215	-3.4505
56	H	0.8746	3.6459	-2.5785
57	H	0.1183	2.4155	-3.6269
58	O	-0.9006	2.8093	-1.8334
59	H	-0.4065	2.1197	-1.3469
60	N	3.0923	-4.6581	1.6271
61	O	4.2723	-4.3928	1.8708
62	O	2.6043	-5.7840	1.7544
63	N	-5.4427	0.4825	2.2775
64	O	-5.6592	-0.0437	3.3715
65	O	-6.2506	1.2089	1.6945

SP_ar3no2obn
Energy (POTENTIAL) = -1567.3626498 Eh

	Atom	X	Y	Z
1	C	-0.3728	-0.2375	-0.3861
2	C	-1.7316	0.1290	0.2352
3	C	-2.6607	0.8126	-0.5668
4	C	-2.0168	-0.0495	1.5948
5	C	-3.8589	1.2768	-0.0402
6	H	-2.4395	0.9816	-1.6147
7	C	-3.2142	0.4062	2.1419
8	H	-1.3038	-0.5422	2.2451
9	C	-4.1226	1.0591	1.3132
10	H	-4.5795	1.7989	-0.6567
11	H	-3.4395	0.2650	3.1913
12	C	0.4400	-1.2798	0.4095
13	C	1.8232	-1.1104	0.5629
14	C	-0.1553	-2.4574	0.8902

15	C	2.5951	-2.0731	1.2052
16	H	2.2983	-0.2195	0.1710
17	C	0.5999	-3.4311	1.5323
18	H	-1.2200	-2.6201	0.7652
19	C	1.9711	-3.2229	1.6867
20	H	3.6624	-1.9415	1.3311
21	H	0.1425	-4.3371	1.9087
22	C	-0.5220	-0.8185	-1.8040
23	C	0.3982	-0.4869	-2.8041
24	C	-1.5255	-1.7502	-2.0995
25	C	0.3062	-1.0670	-4.0698
26	H	1.1844	0.2281	-2.5920
27	C	-1.6096	-2.3274	-3.3651
28	H	-2.2560	-2.0244	-1.3440
29	C	-0.6974	-1.9949	-4.3760
30	H	1.0312	-0.7928	-4.8327
31	H	-2.3994	-3.0462	-3.5704
32	O	0.3805	0.9723	-0.5404
33	C	0.5198	1.8145	0.6222
34	H	-0.4047	2.3880	0.7632
35	H	0.6819	1.2103	1.5225
36	C	1.6940	2.7199	0.3691
37	C	2.9122	2.5275	1.0283
38	C	1.5975	3.7495	-0.5766
39	C	4.0077	3.3491	0.7535
40	H	3.0073	1.7292	1.7608
41	C	2.6931	4.5642	-0.8526
42	H	0.6584	3.9058	-1.1020
43	C	3.9168	4.3805	-0.1892
44	H	4.9460	3.1840	1.2778
45	H	2.6007	5.3569	-1.5916
46	C	5.0899	5.2903	-0.4688
47	H	5.1467	5.5544	-1.5305
48	H	5.0013	6.2295	0.0932
49	H	6.0376	4.8242	-0.1805
50	C	-0.8120	-2.5957	-5.7568
51	H	-1.4712	-1.9916	-6.3942
52	H	0.1626	-2.6471	-6.2533
53	H	-1.2329	-3.6061	-5.7203
54	N	-5.3870	1.5392	1.8797
55	O	-6.1766	2.1188	1.1303
56	O	-5.6004	1.3409	3.0780
57	N	2.7742	-4.2401	2.3671
58	O	2.2032	-5.2524	2.7817
59	O	3.9847	-4.0379	2.4948

SP_obn_an_m2_MeProp
Energy (POTENTIAL) = -617.2875798 Eh

	Atom	X	Y	Z
1	O	0.4605	-1.6832	-1.2595
2	C	1.2297	-0.9333	-0.4014
3	H	1.2721	-1.3465	0.6372
4	H	2.3011	-0.9029	-0.7131
5	C	0.7868	0.5250	-0.2542
6	C	1.5824	1.4597	0.4245
7	C	-0.4283	0.9640	-0.7855
8	C	1.1761	2.7850	0.5694
9	H	2.5391	1.1432	0.8398
10	C	-0.8384	2.2930	-0.6372
11	H	-1.0522	0.2398	-1.2989
12	C	-0.0472	3.2274	0.0405
13	H	1.8165	3.4917	1.0957
14	H	-1.7914	2.6099	-1.0586
15	C	-0.4956	4.6602	0.2210
16	H	-1.3142	4.9133	-0.4615
17	H	-0.8532	4.8441	1.2435
18	H	0.3249	5.3650	0.0390
19	O	-1.9828	-2.0548	-0.4441
20	H	-1.0170	-1.9100	-0.7514
21	C	-2.0371	-1.7018	0.9176
22	H	-1.6954	-0.6694	1.1040
23	H	-3.0787	-1.7734	1.2616
24	H	-1.4334	-2.3695	1.5581
25	O	1.2360	-4.1030	-0.7519
26	H	0.9646	-3.1589	-1.0298
27	C	0.3620	-4.4425	0.2994
28	H	-0.6875	-4.2001	0.0707
29	H	0.4314	-5.5234	0.4921
30	H	0.6084	-3.9276	1.2491

SP_meoh
Energy (POTENTIAL) = -115.7716205 Eh

	Atom	X	Y	Z
1	C	0.0184	0.0015	-0.6649
2	H	-0.9920	0.0068	-1.0841
3	H	0.5486	0.8915	-1.0355

4	H	0.5403	-0.8929	-1.0370
5	O	-0.1206	0.0010	0.7532
6	H	0.7721	0.0008	1.1315

ar3m2obn_meoh_MeProp
Energy (POTENTIAL) = -1846.89875461 Eh

	Atom	X	Y	Z
1	C	-0.0760	0.1736	-0.7612
2	C	-1.3082	-0.6172	-0.3109
3	C	-2.5525	-0.3349	-0.9040
4	C	-1.3012	-1.4986	0.7719
5	C	-3.7215	-0.9281	-0.4525
6	H	-2.6104	0.3907	-1.7088
7	C	-2.4699	-2.1158	1.2239
8	H	-0.3702	-1.7258	1.2797
9	C	-3.7103	-1.8467	0.6201
10	H	-4.6629	-0.6513	-0.9164
11	H	-2.3963	-2.8158	2.0478
12	C	1.2673	-0.3810	-0.2556
13	C	2.2582	0.4710	0.2497
14	C	1.5940	-1.7330	-0.4024
15	C	3.5049	-0.0137	0.6290
16	H	2.0591	1.5338	0.3268
17	C	2.8373	-2.2321	-0.0156
18	H	0.8624	-2.4205	-0.8168
19	C	3.8233	-1.3810	0.5171
20	H	4.2535	0.6852	0.9896
21	H	3.0240	-3.2943	-0.1236
22	C	0.0434	0.2724	-2.2914
23	C	0.6518	1.3896	-2.8786
24	C	-0.3487	-0.7813	-3.1239
25	C	0.8356	1.4577	-4.2584
26	H	0.9818	2.2094	-2.2502
27	C	-0.1562	-0.7107	-4.5042
28	H	-0.8156	-1.6628	-2.6959
29	C	0.4320	0.4114	-5.0993
30	H	1.3063	2.3390	-4.6892
31	H	-0.4742	-1.5430	-5.1283
32	N	5.1066	-1.8354	0.8932
33	C	5.5588	-1.4782	2.2472
34	C	5.4625	-3.2165	0.5699
35	C	7.0560	-1.7349	2.3819
36	H	5.0189	-2.0758	3.0029
37	H	5.3560	-0.4242	2.4463
38	C	6.9650	-3.4177	0.7616
39	H	4.9156	-3.9386	1.2028
40	H	5.2107	-3.4204	-0.4755
41	H	7.3822	-1.5526	3.4101
42	H	7.6081	-1.0594	1.7080
43	O	7.3745	-3.0911	2.0855
44	H	7.2292	-4.4670	0.6008
45	H	7.5123	-2.7980	0.0327
46	N	-4.9181	-2.4106	1.0751
47	C	-5.8013	-3.0121	0.0636
48	C	-4.8810	-3.2207	2.2921
49	C	-7.1888	-3.2460	0.6525
50	H	-5.3867	-3.9751	-0.2825
51	H	-5.8874	-2.3540	-0.8027
52	C	-6.3022	-3.4520	2.8035
53	H	-4.3924	-4.1957	2.1167
54	H	-4.3157	-2.6899	3.0642
55	H	-7.8224	-3.7718	-0.0678
56	H	-7.6545	-2.2769	0.8957
57	O	-7.1243	-4.0644	1.8163
58	H	-6.2872	-4.1270	3.6641
59	H	-6.7392	-2.4893	3.1155
60	O	-0.2303	1.5533	-0.3156
61	C	-0.5714	1.7507	1.0749
62	H	-1.6413	1.5546	1.2096
63	H	-0.0162	1.0429	1.6991
64	C	-0.2189	3.1706	1.4278
65	C	0.9292	3.4637	2.1709
66	C	-1.0207	4.2317	0.9843
67	C	1.2700	4.7853	2.4653
68	H	1.5633	2.6526	2.5216
69	C	-0.6740	5.5495	1.2758
70	H	-1.9062	4.0175	0.3918
71	C	0.4747	5.8500	2.0239
72	H	2.1670	4.9913	3.0450
73	H	-1.3055	6.3604	0.9192
74	C	0.8236	7.2800	2.3644
75	H	0.5727	7.9593	1.5423
76	H	0.2673	7.6215	3.2473
77	H	1.8900	7.3917	2.5864
78	C	0.6056	0.5062	-6.5968
79	H	-0.2162	1.0730	-7.0544

80	H	1.5371	1.0195	-6.8601
81	H	0.6187	-0.4845	-7.0632
82	C	-1.8120	3.8836	-2.5537
83	H	-2.6713	4.3990	-2.9947
84	H	-1.2196	4.6222	-1.9915
85	H	-1.1887	3.4861	-3.3675
86	O	-2.3158	2.8540	-1.7138
87	H	-1.5461	2.4063	-1.3049

SP_obn_an_m1
Energy (POTENTIAL) = -501.5071087 Eh

	Atom	X	Y	Z
1	O	0.9590	-2.3642	-1.4368
2	C	1.6265	-1.4254	-0.7151
3	H	1.9715	-1.7739	0.2989
4	H	2.5851	-1.1056	-1.2042
5	C	0.8623	-0.1170	-0.4347
6	C	1.5026	1.0028	0.1163
7	C	-0.5008	-0.0138	-0.7223
8	C	0.8044	2.1824	0.3725
9	H	2.5681	0.9508	0.3419
10	C	-1.2040	1.1670	-0.4613
11	H	-0.9991	-0.8872	-1.1307
12	C	-0.5674	2.2862	0.0885
13	H	1.3277	3.0398	0.7940
14	H	-2.2674	1.2215	-0.6916
15	C	-1.3277	3.5578	0.3923
16	H	-2.2744	3.6002	-0.1571
17	H	-1.5670	3.6375	1.4617
18	H	-0.7441	4.4482	0.1295
19	O	-1.0676	-3.2993	-0.2418
20	H	-0.2118	-2.9122	-0.7175
21	C	-0.9350	-3.0199	1.1266
22	H	-0.8187	-1.9411	1.3380
23	H	-1.8355	-3.3606	1.6608
24	H	-0.0721	-3.5315	1.5973

SP_ar3m2obn_meoh_MeProp
Energy (POTENTIAL) = -1847.390683 Eh

	Atom	X	Y	Z
1	C	-0.0760	0.1736	-0.7612
2	C	-1.3082	-0.6172	-0.3109
3	C	-2.5525	-0.3349	-0.9040
4	C	-1.3012	-1.4986	0.7719
5	C	-3.7215	-0.9281	-0.4525
6	H	-2.6104	0.3907	-1.7088
7	C	-2.4699	-2.1158	1.2239
8	H	-0.3702	-1.7258	1.2797
9	C	-3.7103	-1.8467	0.6201
10	H	-4.6629	-0.6513	-0.9164
11	H	-2.3963	-2.8158	2.0478
12	C	1.2673	-0.3810	-0.2556
13	C	2.2582	0.4710	0.2497
14	C	1.5940	-1.7330	-0.4024
15	C	3.5049	-0.0137	0.6290
16	H	2.0591	1.5338	0.3268
17	C	2.8373	-2.2321	-0.0156
18	H	0.8624	-2.4205	-0.8168
19	C	3.8233	-1.3810	0.5171
20	H	4.2535	0.6852	0.9896
21	H	3.0240	-3.2943	-0.1236
22	C	0.0434	0.2724	-2.2914
23	C	0.6518	1.3896	-2.8786
24	C	-0.3487	-0.7813	-3.1239
25	C	0.8356	1.4577	-4.2584
26	H	0.9818	2.2094	-2.2502
27	C	-0.1562	-0.7107	-4.5042
28	H	-0.8156	-1.6628	-2.6959
29	C	0.4320	0.4114	-5.0993
30	H	1.3063	2.3390	-4.6892
31	H	-0.4742	-1.5430	-5.1283
32	N	5.1066	-1.8354	0.8932
33	C	5.5588	-1.4782	2.2472
34	C	5.4625	-3.2165	0.5699
35	C	7.0560	-1.7349	2.3819
36	H	5.0189	-2.0758	3.0029
37	H	5.3560	-0.4242	2.4463
38	C	6.9650	-3.4177	0.7616
39	H	4.9156	-3.9386	1.2028
40	H	5.2107	-3.4204	-0.4755
41	H	7.3822	-1.5526	3.4101
42	H	7.6081	-1.0594	1.7080
43	O	7.3745	-3.0911	2.0855
44	H	7.2292	-4.4670	0.6008
45	H	7.5123	-2.7980	0.0327
46	N	-4.9181	-2.4106	1.0751

47	C	-5.8013	-3.0121	0.0636
48	C	-4.8810	-3.2207	2.2921
49	C	-7.1888	-3.2460	0.6524
50	H	-5.3867	-3.9751	-0.2825
51	H	-5.8874	-2.3540	-0.8027
52	C	-6.3022	-3.4520	2.8035
53	H	-4.3924	-4.1957	2.1167
54	H	-4.3157	-2.6899	3.0642
55	H	-7.8224	-3.7718	-0.0678
56	H	-7.6545	-2.2769	0.8957
57	O	-7.1243	-4.0644	1.8163
58	H	-6.2872	-4.1270	3.6641
59	H	-6.7392	-2.4893	3.1155
60	O	-0.2303	1.5533	-0.3156
61	C	-0.5714	1.7507	1.0749
62	H	-1.6413	1.5546	1.2096
63	H	-0.0162	1.0429	1.6991
64	C	-0.2189	3.1706	1.4278
65	C	0.9292	3.4637	2.1709
66	C	-1.0207	4.2317	0.9843
67	C	1.2700	4.7853	2.4653
68	H	1.5633	2.6526	2.5216
69	C	-0.6740	5.5495	1.2758
70	H	-1.9062	4.0175	0.3918
71	C	0.4747	5.8500	2.0239
72	H	2.1670	4.9913	3.0450
73	H	-1.3055	6.3604	0.9192
74	C	0.8236	7.2800	2.3644
75	H	0.5727	7.9593	1.5423
76	H	0.2673	7.6215	3.2474
77	H	1.8900	7.3917	2.5864
78	C	0.6056	0.5062	-6.5968
79	H	-0.2162	1.0730	-7.0544
80	H	1.5371	1.0195	-6.8601
81	H	0.6187	-0.4845	-7.0632
82	C	-1.8120	3.8836	-2.5537
83	H	-2.6713	4.3990	-2.9947
84	H	-1.2196	4.6222	-1.9915
85	H	-1.1887	3.4861	-3.3675
86	O	-2.3158	2.8540	-1.7138
87	H	-1.5461	2.4063	-1.3049

uvvisfrag1_cat
Energy (POTENTIAL) = -1345.1949416 Eh

	Atom	X	Y	Z
1	C	-0.1734	0.0030	1.1688
2	C	-0.1948	-1.2388	0.4546
3	C	0.3467	-1.3705	-0.8570
4	C	-0.7537	-2.4182	1.0246
5	C	0.3302	-2.5627	-1.5394
6	H	0.8501	-0.5219	-1.3074
7	C	-0.7973	-3.6109	0.3428
8	H	-1.2207	-2.3619	2.0017
9	C	-0.2489	-3.7374	-0.9696
10	H	0.8200	-2.6059	-2.5035
11	H	-1.3038	-4.4467	0.8071
12	C	-0.1241	-0.0150	2.6358
13	C	-0.8860	0.8961	3.3990
14	C	0.6818	-0.9465	3.3247
15	C	-0.8479	0.8654	4.7872
16	H	-1.5359	1.6021	2.8921
17	C	0.7294	-0.9547	4.7134
18	H	1.2929	-1.6426	2.7594
19	C	-0.0347	-0.0530	5.4708
20	H	-1.4622	1.5602	5.3538
21	H	1.3712	-1.6697	5.2214
22	C	-0.2015	1.2624	0.4853
23	C	0.3916	2.4308	1.0452
24	C	-0.8402	1.4256	-0.7766
25	C	0.3798	3.6407	0.3949
26	H	0.9211	2.3536	1.9885
27	C	-0.8797	2.6368	-1.4262
28	H	-1.3782	0.5882	-1.2079
29	C	-0.2544	3.7962	-0.8753
30	H	-1.4485	2.7064	-2.3440
31	C	-0.6849	-6.1878	-1.0190
32	C	-0.1535	-5.0368	-3.0988
33	C	0.2108	-7.3339	-1.4995
34	H	-1.7317	-6.3927	-1.2832
35	H	-0.6034	-6.1281	0.0656
36	C	0.7304	-6.2206	-3.4858
37	H	-1.1639	-5.1943	-3.5022
38	H	0.2428	-4.1232	-3.5361
39	H	-0.1781	-8.2839	-1.1233
40	H	1.2302	-7.1876	-1.1082
41	H	0.7211	-6.3519	-4.5711

42	H	1.7655	-6.0361	-3.1574
43	N	-0.2563	-4.9265	-1.6364
44	O	0.2376	-7.4287	-2.9176
45	N	-0.2551	4.9905	-1.5342
46	C	-0.7746	5.1461	-2.8988
47	C	-0.0761	6.2852	-0.8601
48	C	0.0867	6.1470	-3.6748
49	H	-1.8160	5.4952	-2.8617
50	H	-0.7431	4.1943	-3.4278
51	C	0.7656	7.2261	-1.7193
52	H	-1.0683	6.7313	-0.7017
53	H	0.3942	6.1606	0.1125
54	H	-0.3679	6.3406	-4.6501
55	H	1.0904	5.7201	-3.8296
56	O	0.1825	7.3974	-3.0058
57	H	0.8077	8.2145	-1.2541
58	H	1.7899	6.8313	-1.8114
59	H	0.9045	4.4698	0.8512
60	C	0.0351	-0.0507	6.9766
61	H	0.7930	0.6635	7.3248
62	H	-0.9201	0.2474	7.4205
63	H	0.3091	-1.0354	7.3677

ar3m2_rad_MeButa
Energy (POTENTIAL) = -1345.62211829 Eh

	Atom	X	Y	Z
1	C	-0.0912	-0.0165	1.2406
2	C	-0.0197	-1.2860	0.5256
3	C	0.7063	-1.4233	-0.6854
4	C	-0.6688	-2.4522	0.9923
5	C	0.7777	-2.6250	-1.3684
6	H	1.2523	-0.5684	-1.0719
7	C	-0.6131	-3.6588	0.3040
8	H	-1.2601	-2.3987	1.9012
9	C	0.1137	-3.7802	-0.8974
10	H	1.3894	-2.6839	-2.2634
11	H	-1.1687	-4.5026	0.6972
12	C	-0.1947	0.0008	2.7002
13	C	-0.9354	0.9979	3.3795
14	C	0.4351	-0.9790	3.5050
15	C	-1.0385	1.0088	4.7661
16	H	-1.4529	1.7565	2.8006
17	C	0.3291	-0.9587	4.8911
18	H	1.0262	-1.7540	3.0269
19	C	-0.4076	0.0347	5.5546
20	H	-1.6309	1.7834	5.2489
21	H	0.8353	-1.7255	5.4741
22	C	-0.0654	1.2421	0.4989
23	C	0.5417	2.4073	1.0189
24	C	-0.6486	1.3672	-0.7872
25	C	0.5646	3.6082	0.3181
26	H	1.0120	2.3657	1.9966
27	C	-0.6248	2.5579	-1.4933
28	H	-1.1366	0.5048	-1.2307
29	C	-0.0172	3.7171	-0.9610
30	H	-1.0698	2.5892	-2.4830
31	C	-0.3185	-6.2034	-0.9855
32	C	-0.0324	-4.9672	-3.0460
33	C	0.2151	-7.4380	-1.7114
34	H	-1.4226	-6.2102	-1.0175
35	H	-0.0071	-6.2524	0.0623
36	C	0.4941	-6.2412	-3.6986
37	H	-1.1169	-4.8838	-3.2354
38	H	0.4540	-4.1033	-3.5021
39	H	-0.2567	-8.3414	-1.3139
40	H	1.3043	-7.5096	-1.5577
41	H	0.2233	-6.2644	-4.7583
42	H	1.5925	-6.2743	-3.6111
43	N	0.2304	-4.9948	-1.5988
44	O	-0.0802	-7.4003	-3.1033
45	N	0.0250	4.9024	-1.7199
46	C	-1.2389	5.3727	-2.3078
47	C	0.8189	6.0206	-1.2118
48	C	-0.9590	6.4212	-3.3794
49	H	-1.8822	5.8141	-1.5267
50	H	-1.7816	4.5386	-2.7560
51	C	1.0262	7.0515	-2.3205
52	H	0.3313	6.5064	-0.3478
53	H	1.7978	5.6535	-0.8888
54	H	-1.8974	6.8366	-3.7582
55	H	-0.4117	5.9565	-4.2160
56	O	-0.2119	7.5120	-2.8506
57	H	1.5459	7.9294	-1.9259
58	H	1.6388	6.6073	-3.1222
59	H	1.0368	4.4668	0.7816
60	C	-0.4932	0.0701	7.0613

61	H	0.3436	0.6361	7.4933
62	H	-1.4176	0.5510	7.3994
63	H	-0.4561	-0.9375	7.4896

SP_meoh_PMMA
Energy (POTENTIAL) = -115.7697857 Eh

	Atom	X	Y	Z
1	C	0.0194	0.0016	-0.6647
2	H	-0.9917	0.0065	-1.0821
3	H	0.5486	0.8923	-1.0365
4	H	0.5404	-0.8935	-1.0376
5	O	-0.1209	0.0013	0.7522
6	H	0.7710	0.0003	1.1318

SP_obn_an_m2_PMMA
Energy (POTENTIAL) = -617.2788998 Eh

	Atom	X	Y	Z
1	O	0.4605	-1.6871	-1.2571
2	C	1.2377	-0.9344	-0.4117
3	H	1.2984	-1.3453	0.6276
4	H	2.3048	-0.8980	-0.7386
5	C	0.7912	0.5226	-0.2587
6	C	1.5832	1.4571	0.4243
7	C	-0.4240	0.9603	-0.7909
8	C	1.1739	2.7811	0.5723
9	H	2.5397	1.1409	0.8406
10	C	-0.8370	2.2878	-0.6393
11	H	-1.0445	0.2352	-1.3073
12	C	-0.0493	3.2219	0.0425
13	H	1.8116	3.4879	1.1022
14	H	-1.7902	2.6038	-1.0613
15	C	-0.5020	4.6530	0.2269
16	H	-1.3157	4.9078	-0.4611
17	H	-0.8686	4.8318	1.2472
18	H	0.3182	5.3608	0.0545
19	O	-1.9864	-2.0480	-0.4428
20	H	-1.0205	-1.9087	-0.7520
21	C	-2.0358	-1.6934	0.9174
22	H	-1.6885	-0.6624	1.1032
23	H	-3.0773	-1.7593	1.2640
24	H	-1.4344	-2.3634	1.5583
25	O	1.2406	-4.1059	-0.7408
26	H	0.9704	-3.1651	-1.0289
27	C	0.3567	-4.4359	0.3037
28	H	-0.6900	-4.1900	0.0654
29	H	0.4195	-5.5164	0.5027
30	H	0.5972	-3.9178	1.2538
